# Supplementary material for: Decarboxylative alkylation of alkenes
Source: Nature. 2026 Apr 29;653(8113):104–9. doi: 10.1038/s41586-026-10463-1 (PMC13148981; doi:10.1038/s41586-026-10463-1)
Supplement: Supplementary file 1 — This file contains the following sections: materials and methods, experimental data, spectroscopic data and references. [file 41586_2026_10463_MOESM1_ESM.pdf]

nature portfolio

https://doi.org/10.1038/s41586-026-10463-1

Supplementary information

Decarboxylative alkylation of alkenes

In the format provided by the authors and unedited

SUPPLEMENTARY INFORMATION

Decarboxylative Alkylation of Alkenes

Triptesh Kumar Roy[1,2], Federico Maria Tamborini[1,2], Roland Petzold[1], Jianhan Fu[1], Yiben Tang[1,2], Tobias Ritter[1]*

[1] Max-Planck-Institut für Kohlenforschung, Kaiser-Wilhelm-Platz 1, D-45470 Mülheim an der Ruhr, Germany.

[2] Institute of Organic Chemistry, RWTH Aachen University, Landoltweg 1, D-52074 Aachen, Germany.

*E-Mail: ritter@kofo.mpg.de

TABLE OF CONTENTS

TABLE OF CONTENTS 1

MATERIALS AND METHODS 7

EXPERIMENTAL DATA 8

General procedure for the synthesis of alkenyl thianthrenium salts 8

General procedure A for the synthesis of redox-active esters 8

General procedure B for the synthesis of redox-active esters 8

General procedure for preparing activated zinc for cross-coupling reaction 9

General procedure for the synthesis of alkylated alkenes 9

Procedure for alkylation of alkenes via in-situ generated redox-active esters from carboxylic acid 10

General procedure for the optimization of alkylation of alkenes 11

Reaction optimization for alkylation of alkenes 12

Alkene alkylation using radical mediated pathway 12

Catalyst screening 12

Ligand screening 14

Solvent screening 14

Temperature-dependence 15

Reductant screening 15

Reaction of RAE with Zn prior to addition of alkenyl-TT and catalyst 16

Reaction in the presence of zinc chelator 17

Mechanism study 17

Alkylzinc characterization 17

Alkylzinc quantification 23

Alkylzinc optimization 23

Reaction with alkylzinc in-situ formed from alkyl-iodide 27

Side product determination 28

Cyclic Voltammetry experiment 29

Kinetics study of alkylzinc formation 32

Thianthrenium salt reduction experiment 37

Synthesis of alkenyl thianthrenium salts 38

Methyl pent-4-enoate derived thianthrenium salt **TT-1** 38

4-Phenyl-1-butene derived thianthrenium salt **TT-2** 39

Cycloheptene derived thianthrenium salt **TT-3** 40

Oct-1-en-7-yne derived thianthrenium salt **TT-4** 41
3,4-dihydro-2*H*-pyran derived thianthrenium salt **TT-5** 42
2-Vinylbicyclo[2.2.1]heptane derived thianthrenium salt **TT-6** 42
(+)-3-Carene derived thianthrenium salt **TT-7** 43
(*Z*)-4-Octene derived thianthrenium salt **TT-8** 44
Cyclopentene derived thianthrenium salt **TT-9** 45
Cinchophen derived thianthrenium salt **TT-10** 46
Methyl cyclopent-3-ene-1-carboxylate derived thianthrenium salt **(±)-TT-11** 48
(1*Z*, 9*Z*)-Cyclohexadeca-1,9-diene derived thianthrenium salt **TT-12** 48
(*E*)-4-Octene derived thianthrenium salt **TT-13** 49
Tricyclo[6.2.1.0^{2,7}]undeca-4-ene derived thianthrenium salt **TT-14** 50
1-methylcyclopent-1-ene derived thianthrenium salt **TT-15** 51
(–)-*cis*-Rose-oxide derived thianthrenium salt **TT-16** 52
(*S*)-(+)-Carvone derived thianthrenium salt **TT-17** 53
(*S*)-(–)-*β*-Citronellol derived thianthrenium salt **TT-18** 54
(*S*)-(–)-Perillaldehyde derived thianthrenium salt **TT-19** 55
Citral derived thianthrenium salt **TT-20** 56
Cyclohexene derived thianthrenium salt **TT-21** 57

Synthesis of alkyl redox-active esters 58

3-(3,5-Dimethoxyphenyl)propanoic acid derived redox-active ester **RAE-1** 58
4-((Tert-butoxycarbonyl)amino)butanoic acid derived redox-active ester **RAE-2** 58
Tetrahydropyran-4-carboxylic acid derived redox-active ester **RAE-3** 59
4,4-Difluorocyclohexane-1-carboxylic acid derived redox-active ester **RAE-4** 60
1-Benzoylpiperidine-4-carboxylic acid derived redox-active ester **RAE-5** 60
N-tert-butyloxycarbonyl-azetidine-3-carboxylic acid derived redox-active ester **RAE-6** 61
Levulinic acid derived redox-active ester **RAE-7** 62
4-oxocyclohexane-1-carboxylic acid derived redox-active ester **RAE-8** 62
4-(tert-butoxy)-4-oxobutanoic acid derived redox-active ester **RAE-9** 63
Indometacin derived redox-active ester **RAE-10** 63
2-Methoxyacetic acid derived redox-active ester **RAE-11** 64
2,4-D derived redox-active ester **RAE-12** 65
(−)-Menthyloxyacetic acid derived redox-active ester **RAE-13** 65
2-Methoxypropanoic acid derived redox-active ester **(±)-RAE-14** 66
3,3-Difluorocyclobutane-1-carboxylic acid derived redox-active ester **RAE-15** 67
3-(3,5-dichlorophenyl)propanoic acid derived redox-active ester **RAE-16** 67
Baclofen derived redox-active ester **(±)-RAE-17** 68

Synthesis of alkylated alkenes 69

1-Ethyl-3,5-dimethoxybenzene-derived alkylated alkene **1** 69
γ-Aminobutyric acid-derived alkylated alkene **2** 70
Tetrahydropyran-derived alkylated alkene **3** 71
1-Ethyl-3,5-dimethoxybenzene-derived alkylated alkene **4** 72
1,1-Difluorocyclohexane-derived alkylated alkene **5** 73
1-Benzoylpiperidine-derived alkylated alkene **6** 73
N-*tert*-butyloxycarbonyl-azetidine-derived alkylated alkene **7** 74
Butanone-derived alkylated alkene **8** 75
Cyclohexanone-derived alkylated alkene **9** 76
Tert-butyl-propionate-derived alkylated alkene **10** 77
Indometacin-derived alkylated alkene **11** 77
Methoxymethane-derived alkylated alkene **12** 78
1-Benzoylpiperidine-derived alkylated alkene **(±)-13** 79
N-*tert*-butyloxycarbonyl-azetidine-derived alkylated alkene **14** 80
Tetrahydropyran-derived alkylated alkene **15** 81
1,1-Difluorocyclohexane-derived alkylated alkene **16** 81
2,4-D-derived alkylated alkene **17** 82
Methoxymethane-derived alkylated alkene **18** 83
(−)-Menthyloxyacetic acid derived alkylated alkene **19** 84
Methoxyethane derived alkylated alkene **20** 85
Tert-butyl-propionate-derived alkylated alkene **21** 86
γ-Aminobutyric acid-derived alkylated alkene **22** 86
1,1-Difluorocyclobutane derived alkylated alkene **23** 87
1-Ethyl-3,5-dichlorobenzene derived alkylated alkene **24** 88
Baclofen derived alkylated alkene **(±)-25** 89
N-*tert*-butyloxycarbonyl-azetidine derived alkylated alkene **27** 90

SPECTROSCOPIC DATA 91

^{1}H NMR of methyl pent-4-enoate derived thianthrenium salt **TT-1** 91
^{13}C NMR of methyl pent-4-enoate derived thianthrenium salt **TT-1** 92
^{19}F NMR of methyl pent-4-enoate derived thianthrenium salt **TT-1** 93
^{1}H NMR of oct-1-en-7-yne derived thianthrenium salt **TT-4** 94
^{13}C NMR of oct-1-en-7-yne derived thianthrenium salt **TT-4** 95
^{19}F NMR of oct-1-en-7-yne derived thianthrenium salt **TT-4** 96
^{1}H NMR of (+)-3-carene derived thianthrenium salt **TT-7** 97
^{13}C NMR of (+)-3-carene derived thianthrenium salt **TT-7** 98
^{19}F NMR of (+)-3-carene derived thianthrenium salt **TT-7** 99
^{1}H NMR of methyl cyclopent-3-ene-1-carboxylate derived thianthrenium salt **(±)-TT-11** 100

^{13}C NMR of methyl cyclopent-3-ene-1-carboxylate derived thianthrenium salt **(±)-TT-11** 101
^{19}F NMR of methyl cyclopent-3-ene-1-carboxylate derived thianthrenium salt **(±)-TT-11** 102
^{1}H NMR of 1-methylcyclopent-1-ene derived thianthrenium salt **TT-15** 103
^{13}C NMR of 1-methylcyclopent-1-ene derived thianthrenium salt **TT-15** 104
^{19}F NMR of 1-methylcyclopent-1-ene derived thianthrenium salt **TT-15** 105
^{1}H NMR of (–)-*cis*-rose-oxide derived thianthrenium salt **TT-16** 106
^{13}C NMR of (–)-*cis*-rose-oxide derived thianthrenium salt **TT-16** 107
^{19}F NMR of (–)-*cis*-rose-oxide derived thianthrenium salt **TT-16** 108
^{1}H NMR of (*S*)-(+)-carvone derived thianthrenium salt **TT-17** 109
^{13}C NMR of (*S*)-(+)-carvone derived thianthrenium salt **TT-17** 110
^{19}F NMR of (*S*)-(+)-carvone derived thianthrenium salt **TT-17** 111
^{1}H NMR of (*S*)-(–)-*β*-citronellol derived thianthrenium salt **TT-18** 112
^{13}C NMR of (*S*)-(–)-*β*-citronellol derived thianthrenium salt **TT-18** 113
^{19}F NMR of (*S*)-(–)-*β*-citronellol derived thianthrenium salt **TT-18** 114
^{1}H NMR of (*S*)-(–)-perillaldehyde derived thianthrenium salt **TT-19** 115
^{13}C NMR of (*S*)-(–)-perillaldehyde derived thianthrenium salt **TT-19** 116
^{19}F NMR of (*S*)-(–)-perillaldehyde derived thianthrenium salt **TT-19** 117
^{1}H NMR of citral derived thianthrenium salt **TT-20** 118
^{13}C NMR of citral derived thianthrenium salt **TT-20** 119
^{19}F NMR of citral derived thianthrenium salt **TT-20** 120
^{1}H NMR of 3-(3,5-dichlorophenyl)propanoic acid derived redox-active ester **RAE-16** 121
^{13}C NMR of 3-(3,5-dichlorophenyl)propanoic acid derived redox-active ester **RAE-16** 122
^{1}H NMR of 1-ethyl-3,5-dimethoxybenzene-derived alkylated alkene **1** 123
^{13}C NMR of 1-ethyl-3,5-dimethoxybenzene-derived alkylated alkene **1** 124
^{1}H NMR of γ-aminobutyric acid-derived alkylated alkene **2** 125
^{13}C NMR of γ-aminobutyric acid-derived alkylated alkene **2** 126
^{1}H NMR of tetrahydropyran-derived alkylated alkene **3** 127
^{13}C NMR of tetrahydropyran-derived alkylated alkene **3** 128
^{1}H NMR of 1-ethyl-3,5-dimethoxybenzene-derived alkylated alkene **4** 129
^{13}C NMR of 1-ethyl-3,5-dimethoxybenzene-derived alkylated alkene **4** 130
^{1}H NMR of 1,1-difluorocyclohexane-derived alkylated alkene **5** 131
^{13}C NMR of 1,1-difluorocyclohexane-derived alkylated alkene **5** 132
^{19}F NMR of 1,1-difluorocyclohexane-derived alkylated alkene **5** 133
^{1}H NMR of 1-Benzoylpiperidine-derived alkylated alkene **6** 134
^{13}C NMR of 1-Benzoylpiperidine-derived alkylated alkene **6** 135
^{1}H NMR of *N*-*tert*-butyloxycarbonyl-azetidine-derived alkylated alkene **7** 136
^{13}C NMR of *N*-*tert*-butyloxycarbonyl-azetidine-derived alkylated alkene **7** 137
^{1}H NMR of butanone-derived alkylated alkene **8** 138

^{13}C NMR of butanone-derived alkylated alkene **8** 139
^{1}H NMR of cyclohexanone-derived alkylated alkene **9** 140
^{13}C NMR of cyclohexanone-derived alkylated alkene **9** 141
^{1}H NMR of *tert*-butyl-propionate-derived alkylated alkene **10** 142
^{13}C NMR of *tert*-butyl-propionate-derived alkylated alkene **10** 143
^{1}H NMR of indometacin-derived alkylated alkene **11** 144
^{13}C NMR of indometacin-derived alkylated alkene **11** 145
^{1}H NMR of methoxymethane-derived alkylated alkene **12** 146
^{13}C NMR of methoxymethane-derived alkylated alkene **12** 147
^{1}H NMR of 1-Benzoylpiperidine-derived alkylated alkene **(±)-13** 148
^{13}C NMR of 1-Benzoylpiperidine-derived alkylated alkene **(±)-13** 149
^{1}H NMR of *N*-*tert*-butyloxycarbonyl-azetidine-derived alkylated alkene **14** 150
^{13}C NMR of *N*-*tert*-butyloxycarbonyl-azetidine-derived alkylated alkene **14** 151
^{1}H NMR of tetrahydropyran-derived alkylated alkene **15** 152
^{13}C NMR of tetrahydropyran-derived alkylated alkene **15** 153
^{1}H NMR of 1,1-difluorocyclohexane-derived alkylated alkene **16** 154
^{13}C NMR of 1,1-difluorocyclohexane-derived alkylated alkene **16** 155
^{19}F NMR of 1,1-difluorocyclohexane-derived alkylated alkene **16** 156
^{1}H NMR of 2,4-D-derived alkylated alkene **17** 157
^{13}C NMR of 2,4-D-derived alkylated alkene **17** 158
^{1}H NMR of methoxymethane-derived alkylated alkene **18** 159
^{13}C NMR of methoxymethane-derived alkylated alkene **18** 160
^{1}H NMR of (−)-Menthyloxyacetic acid derived alkylated alkene **19** 161
^{13}C NMR of (−)-Menthyloxyacetic acid derived alkylated alkene **19** 162
^{1}H NMR of methoxyethane derived alkylated alkene **20** 163
^{13}C NMR of methoxyethane derived alkylated alkene **20** 164
^{1}H NMR of *tert*-butyl-propionate-derived alkylated alkene **21** 165
^{13}C NMR *tert*-butyl-propionate-derived of alkylated alkene **21** 166
^{1}H NMR of γ-aminobutyric acid-derived alkylated alkene **22** 167
^{13}C NMR of γ-aminobutyric acid-derived alkylated alkene **22** 168
^{1}H NMR of 1,1-difluorocyclobutane derived alkylated alkene **23** 169
^{13}C NMR of 1,1-difluorocyclobutane derived alkylated alkene **23** 170
^{19}F NMR of 1,1-difluorocyclobutane derived alkylated alkene **23** 171
^{1}H NMR of 1-ethyl-3,5-dichlorobenzene derived alkylated alkene **24** 172
^{13}C NMR of 1-ethyl-3,5-dichlorobenzene derived alkylated alkene **24** 173
^{1}H NMR of baclofen derived alkylated alkene **(±)-25** 174
^{13}C NMR of baclofen derived alkylated alkene **(±)-25** 175
^{1}H NMR of iodide compound **26** 176

^{13}C NMR of iodide compound **26** 177
^{1}H NMR of *N*-*tert*-butyloxycarbonyl-azetidine derived alkylated alkene **27** 178
^{13}C NMR of *N*-*tert*-butyloxycarbonyl-azetidine derived alkylated alkene **27** 179
^{1}H NMR of side product **28** 180
^{13}C NMR of side product **28** 181
^{1}H NMR of side product **29** 182
^{13}C NMR of side product **29** 183
^{1}H NMR of side product **30** 184
^{13}C NMR of side product **30** 185

REFERENCES 186

MATERIALS AND METHODS

All manipulations were performed using oven-dried glassware (110°C for a minimum of 12 hours) and standard Schlenk techniques under an atmosphere of argon, unless otherwise stated. Concentration under reduced pressure was performed by rotary evaporation at 39 – 41°C at a suitable pressure, unless otherwise stated. The purified compounds were further dried under high vacuum (0.01 – 0.005 mbar). Temperature range of 23 – 27°C is defined as 25°C.

Solvents

Anhydrous solvents were obtained from *Phoenix Solvent Drying Systems*. Acetonitrile (MeCN) was purchased from *Fischer Chemical* and used as received. *N*,*N*-Dimethylformamide (DMF, 99.8%, Extra Dry) was ordered from ThermoFischer Scientific Chemicals and stored in a nitrogen-filled Glovebox. All deuterated solvents were purchased from *Euriso-Top*.

Chromatography

Thin layer chromatography (TLC) was performed using Polygram Sil G/UV254 plates from *Macherey-Nagel* pre-coated with 0.20 mm silica and a fluorescence indicator. Plates were visualized by irradiation at 254 nm from a *Herolab* UV-lamp. Flash chromatography was performed using silica gel (40 – 63 µm particle size) purchased from *Geduran* or Biotage® Sfär Silica columns (100 Å pore size, 60 µm particle size) on an automated purification system (Biotage® Isolera One). The detailed solvent composition of the eluents is given for every compound individually.

Spectroscopy and Instruments

NMR spectra were recorded on a *Bruker Ascend*™ 500 spectrometer, a *Bruker AVANCE Neo* 600 MHz equipped with a BBO cryoprobe, or a *Bruker AVANCE III* 600 MHz equipped with a TCI cryoprobe. Chemical shifts are reported in ppm with the solvent residual peak as the internal standard. For ^{1}H NMR: $CDCl_3$, δ 7.26, CD_2Cl_2, δ 5.32, DMSO-d_6, δ 2.50, CD_3CN, δ 1.96. For ^{13}C NMR: CD_3CN, δ 118.26, $CDCl_3$, δ 77.16, CD_2Cl_2, δ 53.84, DMSO-d_6, δ 39.52.[1] ^{19}F NMR spectra were referenced using a unified chemical shift scale based on the ^{1}H resonance of tetramethylsilane (1% (v/v) solution in the respective solvent). Data is reported as follows: s = singlet, d = doublet, t = triplet, q = quartet, p = pentet, h= hexet, hept = heptet, m = multiplet, bs = broad singlet; coupling constants in Hz. Liquid-chromatography-mass spectrometry (LC-MS) was performed with an Agilent Technology 1260 Infinity HPLC system coupled to an Agilent Technologies 6120 Quadrupole mass analyzer.

Starting materials

All substrates were used as received from commercial suppliers, unless otherwise stated. Chemicals were purchased from *Sigma-Aldrich*, *TCI*, *Alfa Aesar, Abcr, or BLDpharm*. Zinc was purchased form *Merck* and the mesh size is < 60 µm.

EXPERIMENTAL DATA

General procedure for the synthesis of alkenyl thianthrenium salts

Under ambient atmosphere, a 20 mL borosilicate vial equipped with a magnetic stir bar was charged with alkene (0.50 mmol, 1.0 equiv.), thianthrene-*S*-oxide (**TTO**) (120 mg, 0.517 mmol, 1.03 equiv.), and MeCN (2.0 mL, *c* = 0.25 M). After cooling to 0°C, trifluoroacetic anhydride (0.21 mL, 0.31 g, 1.5 mmol, 3.0 equiv.) was added dropwise within 30 seconds, followed by dropwise addition of HOTf (52 μL, 88 mg, 0.59 mmol, 1.2 equiv.) or $HBF_4{\cdot}OEt_2$ (87 μL, 97 mg, 0.59 mmol, 1.2 equiv.) within 10 seconds. After stirring the mixture at 0°C (prepared by ice bath) or –40°C (prepared by mixing dry ice and MeCN) for 30 – 60 min followed by stirring at 25°C for 0 – 30 min, the resulting mixture was concentrated under reduced pressure and subsequently dichloromethane (DCM, 10 mL) was added, and the residue was dissolved in DCM. The DCM solution was poured onto a saturated aqueous $NaHCO_3$ solution (ca. 20 mL). The combined mixture was poured into a separatory funnel, and the layers were separated. The DCM layer was collected, and the aqueous layer was further extracted with DCM (2 × ca. 10 mL). The combined DCM phase was washed with aqueous $NaBF_4$ solution (2 × ca. 20 mL, 5% w/w). The DCM layer was dried over Na_2SO_4 (10 g), filtered, and the solvent was removed under reduced pressure. The residue was purified by chromatography on silica gel, eluting with DCM/methanol (50:1, v/v). The product-containing fractions were collected and concentrated under reduced pressure. The residue was further dried in vacuo to afford the alkenyl thianthrenium salt.

General procedure A for the synthesis of redox-active esters

To a 50 mL round bottom flask containing a teflon-coated magnetic stirring bar were added carboxylic acid (6.0 mmol, 1.0 equiv.), 4-dimethylamino-pyridine (DMAP, 73.3 mg, 0.60 mmol, 0.10 equiv.), *N*,*N*'-diisopropylcarbodiimide (DIC, 1.0 mL, 0.83 g, 6.6 mmol, 1.1 equiv.), *N*-hydroxyphthalimide (**NHPI**, 979 mg, 6.0 mmol, 1.0 equiv.) and DCM (30 mL, *c* = 0.20 M). After addition, the mixture was stirred for 16 h at 25°C. After stirring, the reaction mixture was filtered over SiO_2 (10 g), and the SiO_2 was subsequently washed with DCM (10 mL). The solvent was removed under reduced pressure, and the resulting residue was purified by column chromatography on silica gel, eluting with EtOAc/hexanes (1:3, v/v) to afford the redox-active ester.

General procedure B for the synthesis of redox-active esters

A round-bottom flask was charged with *N*-hydroxyphthalimide (**NHPI**, 979 mg, 6.0 mmol, 1.0 equiv.), carboxylic acid (1.38 g, 6.0 mmol, 1.0 equiv.) and 4-dimethylamino-pyridine (DMAP, 73.3 mg, 0.60 mmol, 0.10 equiv.). Dichloromethane was added (30 mL, *c* = 0.20 M) and the mixture was stirred vigorously. Finally, *N*-ethyl-*N'*-(3-dimethylaminopropyl)-carbodiimide hydrochloride (EDC·HCl, 1.27 g, 6.60 mmol, 1.10 equiv.) was added. The mixture was allowed to stir for 16 h at 25°C. After stirring, the mixture was diluted with 10 mL DCM and the DCM solution was poured onto a 1M solution of HCl (ca. 60 mL). The combined mixture was poured into a separatory funnel, and the layers were separated. The DCM layer was collected, and the organic layer was further extracted with 1M solution of HCl (2 × ca. 60 mL). The DCM layer was dried over Na_2SO_4 (10 g), filtered, and the solvent was removed under reduced pressure. The resulting residue was

either purified by column chromatography on silica gel, eluting with EtOAc/hexanes (1:3, v/v) to afford the redox-active ester or directly used for cross-coupling reactions.

General procedure for preparing activated zinc for cross-coupling reaction

Under an ambient atmosphere, zinc powder (1 g) was added to a 20-mL borosilicate vial containing a teflon-coated magnetic stir bar. Subsequently, saturated aqueous NH_4Cl solution (ca. 5 mL) was added to the vial and the mixture was stirred vigorously (1000 rpm) for 1 hour. Following this, the liquid was decanted, and the remaining zinc was washed sequentially with distilled water, acetone, ethanol, DMF and diethyl ether. The vial was then further dried in vacuo and immediately transferred into a nitrogen-filled glovebox for direct use in cross-coupling reactions.

General procedure for the synthesis of alkylated alkenes

General procedure for the synthesis of alkylated alkenes using a Schlenk line

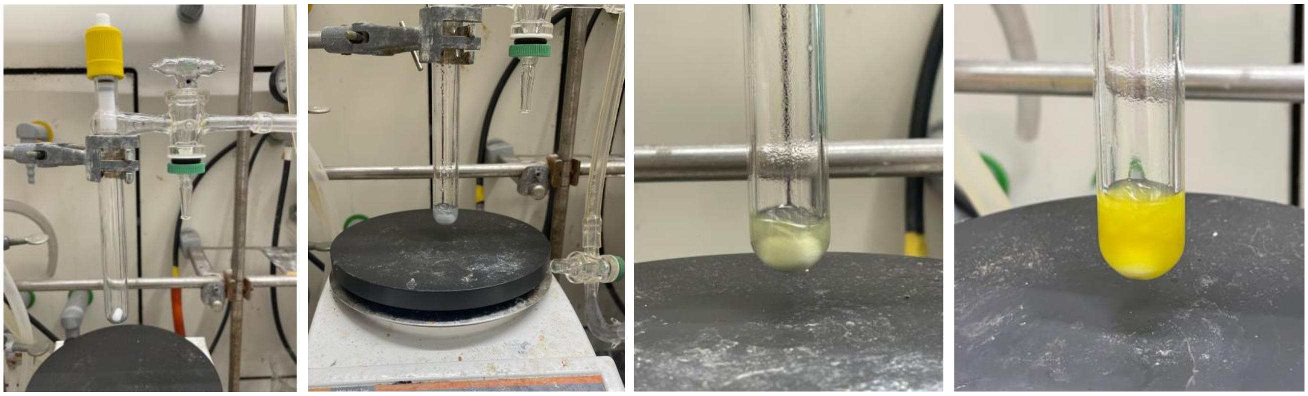

Figure S1. Reaction setup in a Schlenk tube (from left to right): flame-dried Schlenk tube, after redox-active ester and zinc addition, after stirring for 120 minutes, after thianthrenium salt and $PdCl_2(amphos)_2$ addition.

To a flame-dried 10 mL Schlenk tube under argon atmosphere containing a teflon-coated magnetic stir bar were added the redox-active ester (0.44 mmol, 2.2 equiv.) and zinc powder (39.2 mg, 0.60 mmol, 3.0 equiv.). The Schlenk tube was sealed with a septum stopper. The Schlenk tube was evacuated and backfilled with argon. Dry DMF (0.80 mL, c = 0.25 M) was added to the solids. The reaction mixture was stirred (800 rpm) at 25°C on a stirring plate for 2 h. In a separate 4-mL vial, $PdCl_2(amphos)_2$ (5.6 mg, 8.0 μmol, 4.0 mol%) and the alkenyl thianthrenium salt (0.20 mmol, 1.0 equiv.) were added under ambient atmosphere. Dry DMF (0.80 mL, c = 0.25 M) was added and the mixture was stirred at 25°C for 5 min. After that, the obtained yellow suspension was added to the Schlenk finger via a syringe. The reaction mixture was stirred (800 rpm) at 25°C for 16 h. The reaction mixture was diluted with ethyl acetate (2 mL), and transferred to a separatory funnel that contained ethyl acetate (20 mL). The organic layer was washed with brine (1 × 25 mL). The aqueous layer was then extracted with ethyl acetate (3 × 20 mL). The organic layers were combined, dried over $MgSO_4$, filtered, and concentrated under reduced pressure. The resulting residue was purified by column chromatography on silica gel to afford the desired product.

General procedure for the synthesis of alkylated alkenes using a glovebox

Under an ambient atmosphere, the redox-active ester (0.44 mmol, 2.2 equiv.) was added to a 4-mL vial that contained a teflon-coated magnetic stir bar. The vial was transferred to a nitrogen-filled glovebox, where zinc powder (39.2 mg, 0.60 mmol, 3.0 equiv.) and DMF (0.80 mL, *c* = 0.25 M) were added. The reaction mixture was stirred (800 rpm) at 25°C on a stirring plate for 2 h. In a separate 4-mL vial, $PdCl_2(amphos)_2$ (5.6 mg, 8.0 µmol, 4.0 mol%) and the alkenyl thianthrenium salt (0.20 mmol, 1.0 equiv.) were added under ambient atmosphere. The vial was transferred to a nitrogen-filled glovebox and DMF (0.80 mL, *c* = 0.25 M) was added. The mixture was stirred at 25°C for 5 min. After that, the obtained yellow suspension was added to the first vial. The vial was sealed, and the reaction mixture was stirred (800 rpm) at 25°C for 16 h. The reaction mixture was diluted with ethyl acetate (2 mL), and transferred to a separatory funnel that contained ethyl acetate (20 mL). The organic layer was washed with brine (1 × 25 mL). The aqueous layer was then extracted with ethyl acetate (3 × 20 mL). The organic layers were combined, dried over $MgSO_4$, filtered, and concentrated under reduced pressure. The resulting residue was purified by column chromatography on silica gel to afford the desired product.

Note: For convenience and efficiency, the alkylation reactions were conducted in the glovebox unless stated otherwise. No change in yield was found when the alkylation reactions were carried out using a Schlenk line or in the glovebox. All manipulations involving alkylzinc species should be conducted under an inert atmosphere using rigorously dried solvents, as these reagents are highly sensitive to air and moisture.

Procedure for alkylation of alkenes via in-situ generated redox-active esters from carboxylic acid

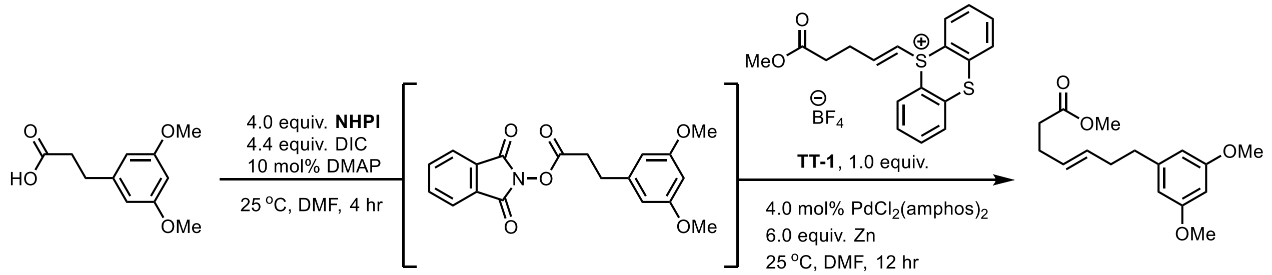

R-3, 4.0 equiv. **RAE-1** In-situ formed, no isolation required **1**, 40%

Under an ambient atmosphere, the carboxylic acid 3-(3,5-dimethoxyphenyl)-propanoic acid (84.1 mg, 0.40 mmol, 4.0 equiv.), *N*-hydroxyphthalimide (**NHPI**, 65.2 mg, 0.40 mmol, 4.0 equiv.) and 4-dimethylamino-pyridine (DMAP, 4.9 mg, 0.04 mmol, 0.4 equiv.) was added to a 4-mL vial that contained a teflon-coated magnetic stir bar. The vial was transferred to a nitrogen-filled glovebox, where *N*,*N*'-diisopropylcarbodiimide (DIC, 68 µL, 55.5 mg, 0.44 mmol, 4.4 equiv.) and DMF (0.80 mL, *c* = 0.25) were added. The reaction mixture was stirred (800 rpm) at 25°C on a stirring plate for 4 h. In a separate 4-mL vial inside the glovebox, zinc powder (39.2 mg, 0.60 mmol, 6.0 equiv.) was added, and the reaction mixture was taken and passed through 0.22 µm syringe filter into the new vial. The mixture was again put on the stirring plate and was stirred (800 rpm) at 25°C for 2 h. In a separate 4-mL vial, $PdCl_2(amphos)_2$ (5.6 mg, 8.0 µmol, 4.0 mol%) and the alkenyl

thianthrenium salt **TT-1** (41.6 mg, 0.10 mmol, 1.0 equiv.) were added under ambient atmosphere. The vial was transferred to a nitrogen-filled glovebox and DMF (0.80 mL, *c* = 0.25) was added. The mixture was stirred at 25°C for 5 min. After that, the obtained yellow suspension was added to the second vial. The vial was sealed, and the reaction mixture was stirred (800 rpm) at 25°C for 16 h outside the glovebox. The reaction mixture was diluted with ethyl acetate (2 mL), and transferred to a separatory funnel that contained ethyl acetate (20 mL). The organic layer was washed with brine (1 × 25 mL). The aqueous layer was then extracted with ethyl acetate (3 × 20 mL). The organic layers were combined, dried over $MgSO_4$, filtered, and concentrated under reduced pressure. The resulting residue was purified by column chromatography on silica gel, eluting with EtOAc/hexanes (3:97, v/v) to afford the desired product **1** (11 mg, 40%) as colorless solid.

General procedure for the optimization of alkylation of alkenes

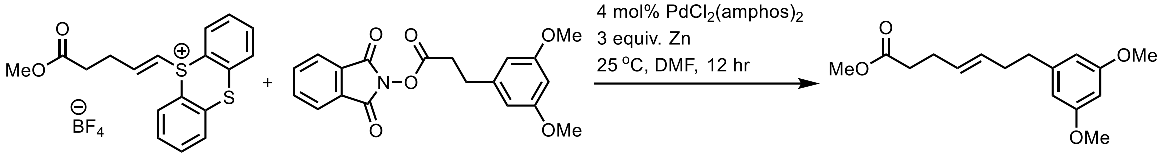

TT-1, 1.0 equiv. **RAE-1**, 2.2 equiv. **1**, yield

Under an ambient atmosphere, the redox-active ester **RAE-1** (78.2 mg, 0.220 mmol, 2.20 equiv.), $PdCl_2(amphos)_2$ (5.6 mg, 8.0 μmol, 4.0 mol%) and the alkenyl thianthrenium salt **TT-1** (41.6 mg, 0.10 mmol, 1.0 equiv.) were added to a 4-mL vial that contained a teflon-coated magnetic stir bar. The vial was transferred to a nitrogen-filled glovebox, where zinc powder (19.6 mg, 0.30 mmol, 3.0 equiv.) and DMF (0.80 mL, *c* = 0.13) were added. The vial was sealed, and the reaction mixture was stirred (800 rpm) at 25°C for 16 h outside the glovebox. The reaction mixture was diluted with ethyl acetate (2 mL), and transferred to a separatory funnel that contained ethyl acetate (20 mL). The organic layer was washed with brine (1 × 25 mL). The aqueous layer was then extracted with ethyl acetate (3 × 20 mL). The organic layers were combined, dried over $MgSO_4$, filtered, and concentrated under reduced pressure. Dibromomethane (CH_2Br_2) (7.0 μL, 17 mg, 0.10 mmol) was added, and the exact weight added was noted. $CDCl_3$ (0.5 mL) was added, and the resulting mixture was vigorously shaken for 10 seconds. Then, an aliquot of the mixture was taken and passed into an NMR tube through a Pasteur pipette that had been fitted with a piece of cotton to remove insoluble solids. More $CDCl_3$ was passed through the same Pasteur pipette into the NMR tube until the NMR tube was filled with a volume of at least 0.5 mL. Then, a ^1H NMR spectrum (NS = 16, D1 = 1 s, SW = 19.9875 ppm, O1P = 6.175 ppm, TD = 65536, O2 = 3089.62 Hz, O2P = 6.175 ppm) was measured, and the yield of **1** was determined by comparing the relative integrals of dibromomethane's proton signal (δ = 4.95 ppm, s, 2H) and two of **1**'s olefinic proton signals (δ = 5.48 ppm, m, 2H).

Reaction optimization for alkylation of alkenes

Alkene alkylation using radical mediated pathway

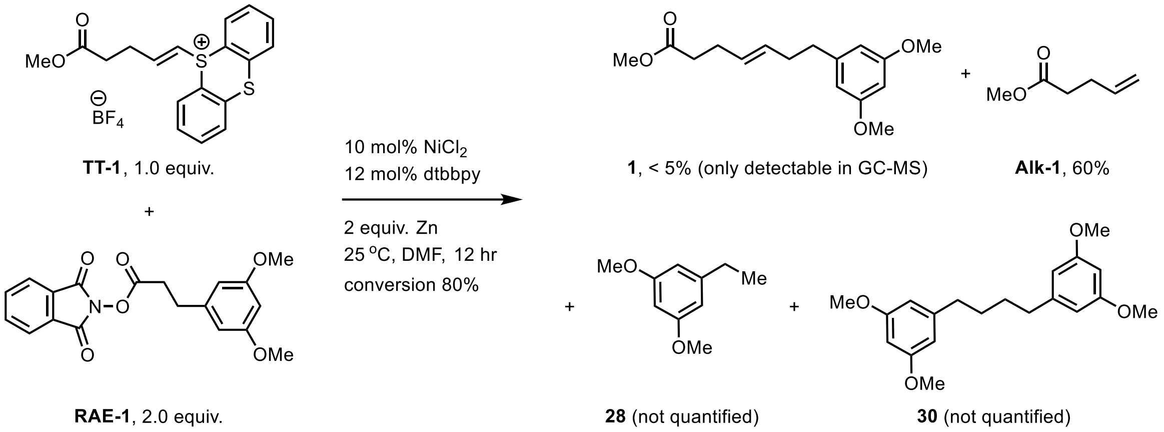

Under an ambient atmosphere, the redox-active ester **RAE-1** (71.1 mg, 0.20 mmol, 2.0 equiv.), $NiCl_2$ (1.3 mg, 10 μmol, 10 mol%), 4,4′-di-*tert*-butyl-2,2′-dipyridine (3.2 mg, 12 μmol, 12 mol%) and the alkenyl thianthrenium salt **TT-1** (41.6 mg, 0.10 mmol, 1.0 equiv.) were added to a 4-mL vial that contained a teflon-coated magnetic stir bar. The vial was transferred to a nitrogen-filled glovebox, where zinc powder (19.6 mg, 0.30 mmol, 3.0 equiv.) and DMF (0.80 mL, *c* = 0.13) were added. The vial was sealed, and the reaction mixture was stirred (800 rpm) at 25°C for 16 h outside the glovebox. The reaction mixture was diluted with ethyl acetate (2 mL), and transferred to a separatory funnel that contained ethyl acetate (20 mL). The organic layer was washed with saturated NH_4Cl solution (1 × 20 mL). The aqueous layer was then extracted with ethyl acetate (3 × 20 mL). The organic layers were combined, dried over $MgSO_4$, filtered, and concentrated under reduced pressure. Dibromomethane (CH_2Br_2) (7.0 μL, 17 mg, 0.10 mmol) was added, and the exact weight added was noted. $CDCl_3$ (0.5 mL) was added, and the resulting mixture was vigorously shaken for 10 seconds. Then, an aliquot of the mixture was taken and passed into an NMR tube through a Pasteur pipette that had been fitted with a piece of cotton to remove insoluble solids. More $CDCl_3$ was passed through the same Pasteur pipette into the NMR tube until the NMR tube was filled with a volume of at least 0.5 mL. Then, a ^{1}H NMR spectrum (NS = 16, D1 = 1 s, SW = 19.9875 ppm, O1P = 6.175 ppm, TD = 65536, O2 = 3089.62 Hz, O2P = 6.175 ppm) was measured, and the yield of **1** was determined by comparing the relative integrals of dibromomethane's proton signal (δ = 4.95 ppm, s, 2H) and two of **1**'s olefinic proton signals (δ = 5.48 ppm, m, 2H).

Catalyst screening

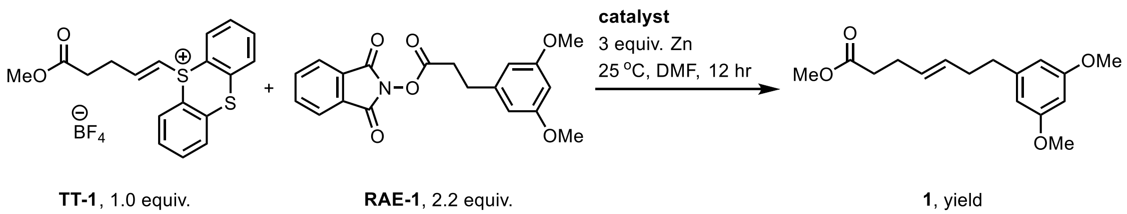

Table S1. Catalyst screening

Entry	catalyst	yield /%
**1**	10 mol% $NiCl_2(DME)$ + 12 mol% bpy	< 5
**2**	10 mol% $NiCl_2$ + 12 mol% dtbbpy	< 5
**3**	10 mol% $NiCl_2(DME)$ + 12 mol% 1-bpp	< 5
**4**	10 mol% $NiCl_2(DME)$ + 12 mol% tpy	< 5
**5**	10 mol% $NiCl_2(PPh_3)_2$	< 5
**6**	10 mol% $NiCl_2(PCy_3)_2$	< 5
**7**	10 mol% $NiCl_2(Py)_4$	< 5
**8**	10 mol% $FeCl_2$ + 24 mol% PPh_3	< 5
**9**	10 mol% $Fe(OTf)_2$	< 5
**10**	10 mol% $Fe(acac)_3$ + 12 mol% dppbz	< 5
**11**	10 mol% $FeCl_3{\cdot}6H_2O$ + 12 mol% dppbz	< 5
**12**	10 mol% CuI + 12 mol% tpy	n.o.
**13**	10 mol% $Cu(OTf)_2$ + 12 mol% bpy	n.o.
**14**	4 mol% $PdCl_2(amphos)_2$	34

n.o. = no product observed

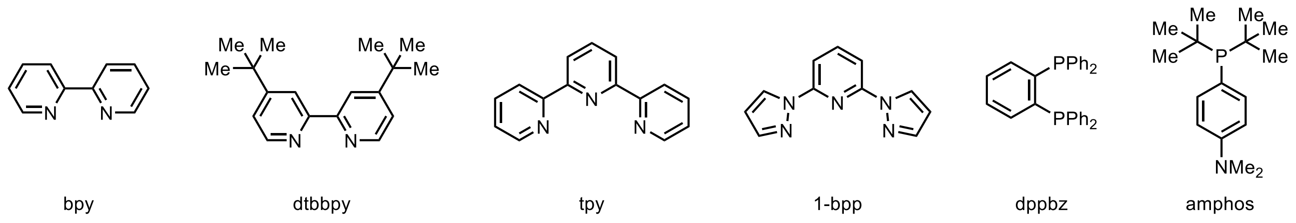

Ligand screening

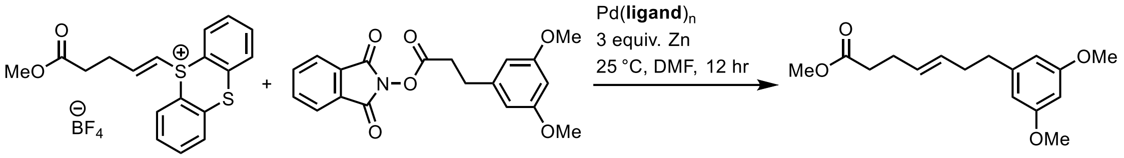

TT-1, 1.0 equiv. **RAE-1**, 2.2 equiv. **1**, yield

Table S2. Catalyst and ligand screening

Entry	catalyst	yield /%
**1**	4 mol% $PdCl_2(amphos)_2$	34
**2**	4 mol% $PdCl_2$ + 8 mol% Xantphos	6
**3**	4 mol% $PdCl_2$ + 8 mol% dppe	< 5
**4**	4 mol% $PdCl_2$ + 8 mol% dppbz	< 5
**5**	4 mol% $Pd(PPh_3)_4$	14
**6**	4 mol% $Pd(P^tBu_3)_2$	17
**7**	4 mol% $Pd(OAc)_2$ + 8 mol% Amphos	20
**8**	4 mol% $Pd_2(dba)_3$ + 8 mol% Amphos	14

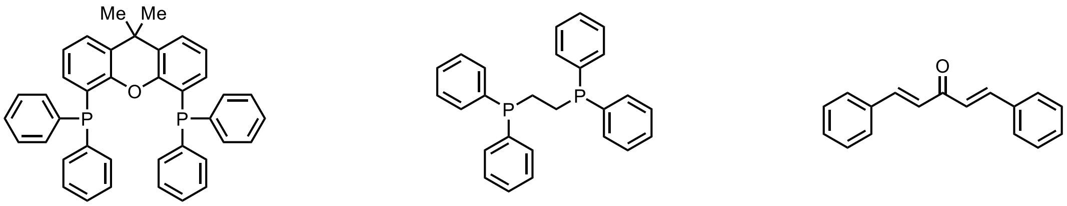

Xantphos dppe dba

Solvent screening

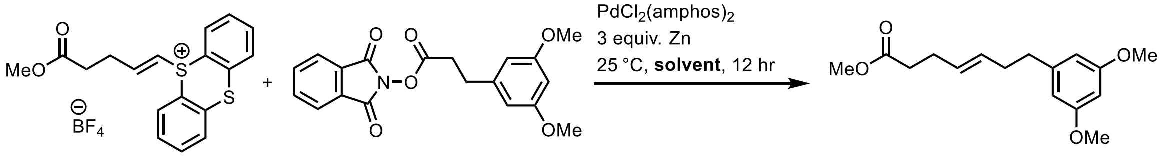

TT-1, 1.0 equiv. **RAE-1**, 2.2 equiv. **1**, yield

Table S3. Solvent screening

Entry	solvent	yield /%
**1**	DMF	34
**2**	DMA	31
**3**	THF	n.o.
**4**	MeCN	n.o.
**5**	Toluene	n.o.
**6**	1,4-dioxane	n.o.

n.o. = no product observed

Temperature-dependence

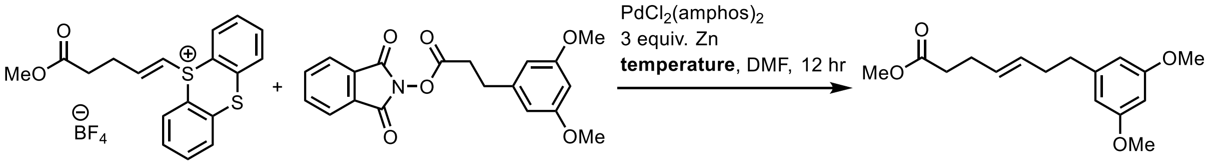

TT-1, 1.0 equiv. **RAE-1**, 2.2 equiv. **1**, yield

Table S4. Reaction temperature dependence investigation

Entry	temperature	yield /%
**1**	25°C	34
**2**	40°C	33
**3**	60°C	28
**4**	80°C	22

Reductant screening

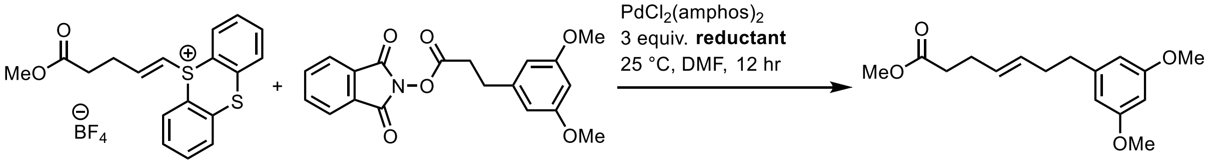

TT-1, 1.0 equiv. **RAE-1**, 2.2 equiv. **1**, yield

Table S5. Screening of reductants

Entry	reductant	yield /%
**1**	No reductant	n.o.
**2**	Zn	34
**3**	Mn	n.o.
**4**	Mg	n.o.
**5**	TDAE	n.o.

n.o. = no product observed

Reaction of RAE with Zn prior to addition of alkenyl-TT and catalyst

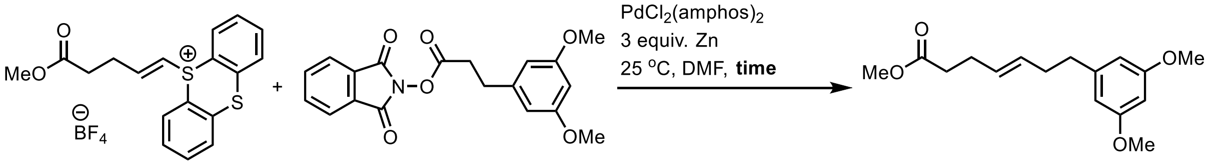

TT-1, 1.0 equiv. **RAE-1**, 2.2 equiv. **1**, yield

Table S6. Time screening for reaction of RAE with Zn prior to addition of alkenyl-TT and catalyst

Entry	time	yield /%
**1**	0 min	34
**2**	15 min	46
**3**	30 min	54
**4**	45 min	60
**5**	60 min	72
**6**	90 min	75
**7**	120 min	75

Reaction in the presence of zinc chelator

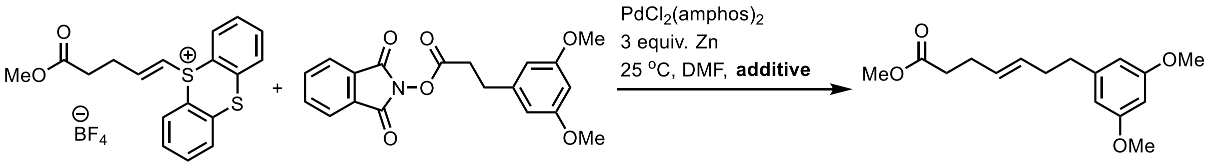

TT-1, 1.0 equiv. **RAE-1**, 2.2 equiv. **1**, yield

Table S7. Screening for zinc chelator

Entry	additive	yield /%
**1**	No additive	75
**2**	12 mol% EDTA	70
**3**	1 equiv. EDTA	< 5

Mechanism study

Alkylzinc characterization

NMR characterization of alkylzinc formation experiment in DMF-d_7

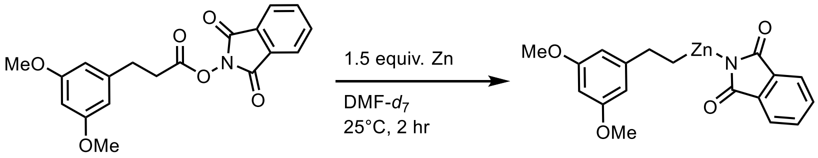

RAE-1, 1.0 equiv. **I-1**

Under an ambient atmosphere, the redox-active ester **RAE-1** (35.5 mg, 0.10 mmol, 1.0 equiv.) was added to a 4-mL vial that contained a teflon-coated magnetic stir bar. The vial was transferred to a nitrogen-filled glovebox, where zinc powder (9.8 mg, 0.15 mmol, 1.5 equiv.) and DMF (0.20 mL, c = 0.25) were added. The reaction mixture was stirred (800 rpm) in the glovebox at 25°C on a stirring plate for 2 hours and then was allowed to stand for 5 minutes. After zinc had completely precipitated, the supernatant was collected, passed through a 0.22 µm syringe filter, and transferred to a J-Young NMR tube. More solvent was passed through the same filter into the tube until the tube was filled with a volume of at least 0.5 mL.

In parallel, a blank sample containing only DMF-d_7 and a reference sample containing the redox-active ester **RAE-1** (35.5 mg, 0.10 mmol, 1.0 equiv.) in DMF-d_7 (0.20 mL, c = 0.25) were prepared under identical conditions. All J-Young tubes were removed from the glovebox and submitted for NMR analysis.

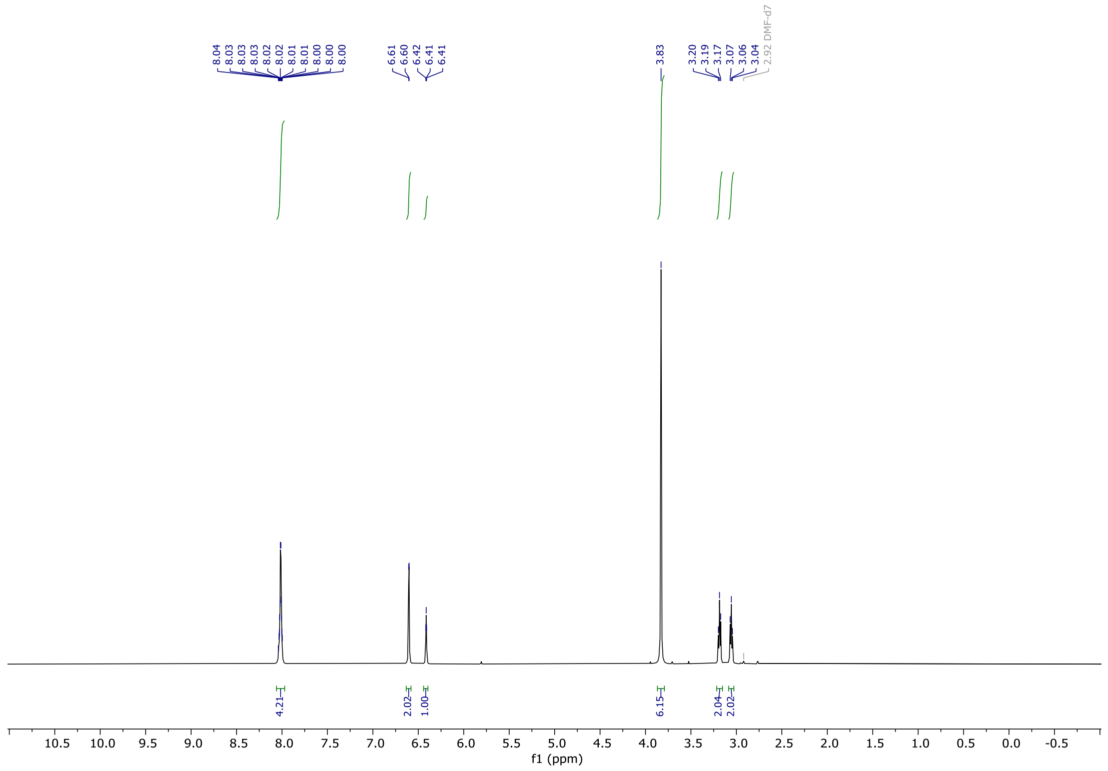

Figure S2. ^{1}H NMR of the reference sample contained only **RAE-1** in DMF-d_7, the chemical shift of solvent residue is confirmed to be 2.92 ppm in the blank experiment.

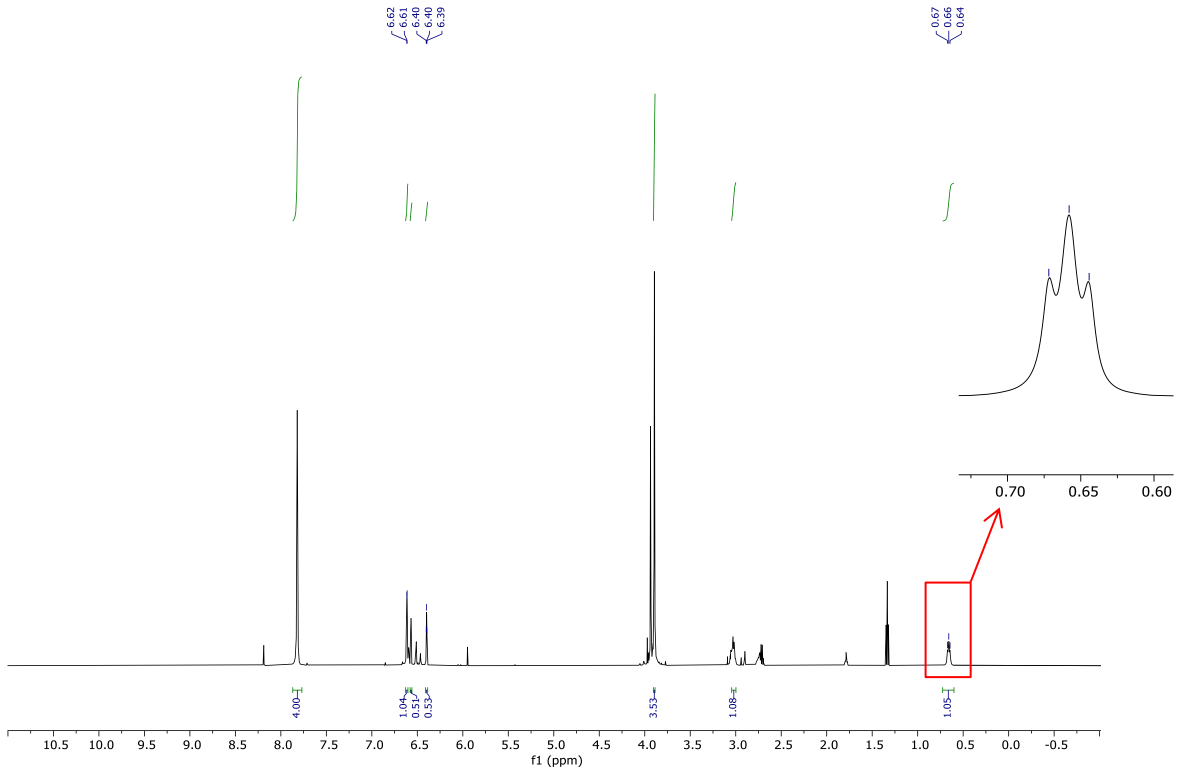

Figure S3. ^{1}H NMR of **RAE-1** after zinc addition 2hr in DMF-d_7, the red box highlights the appearance of new upfield signals consistent with formation of alkyl-zinc[2].

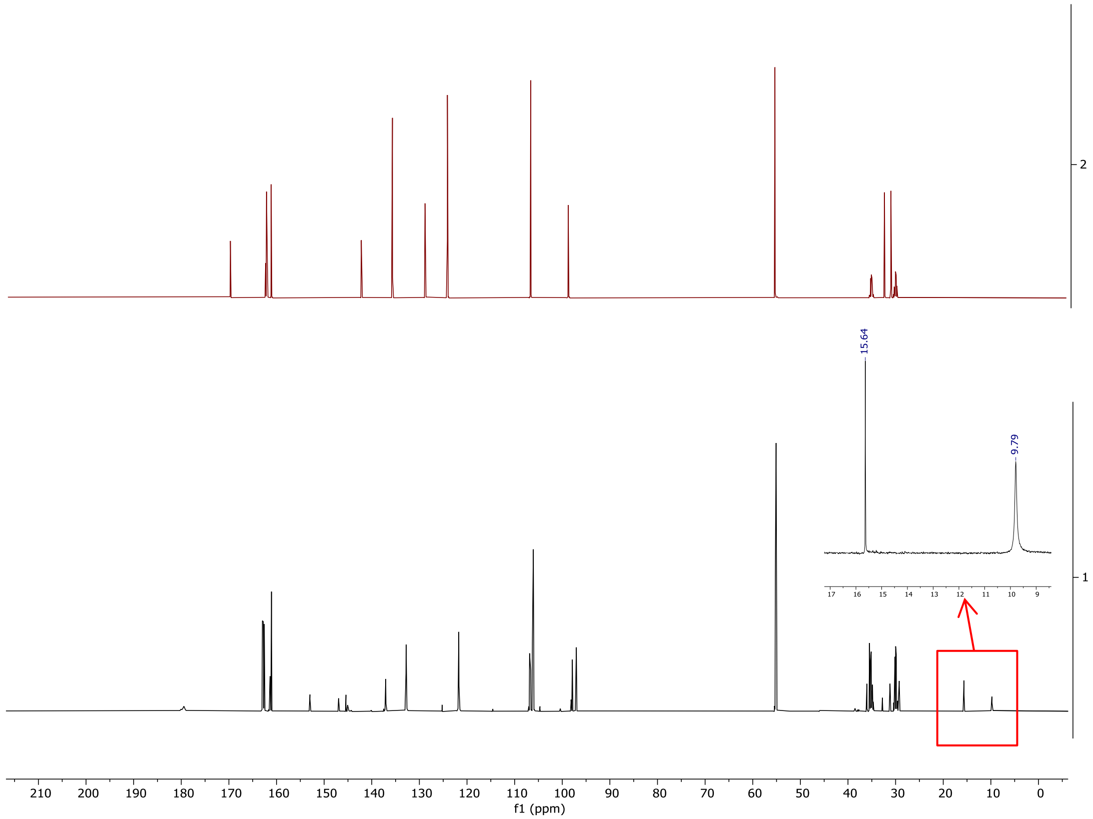

Figure S4. ^{13}C NMR of **RAE-1** before and after zinc addition 2hr in DMF-d_7. The red box highlights the appearance of new upfield signals consistent with formation of alkyl-zinc[2].

NMR characterization of alkylzinc formation experiment in THF-d_8

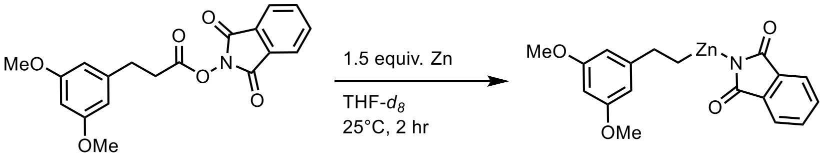

RAE-1, 1.0 equiv. **I-1**

Under an ambient atmosphere, the redox-active ester **RAE-1** (35.5 mg, 0.10 mmol, 1.0 equiv.) was added to a 4-mL vial that contained a teflon-coated magnetic stir bar. The vial was transferred to a nitrogen-filled glovebox, where zinc powder (9.8 mg, 0.15 mmol, 1.5 equiv.) and DMF (0.20 mL, c = 0.25) were added. The reaction mixture was stirred (800 rpm) in the glovebox at 25°C on a stirring plate for 2 hours and then allowed to stand for 5 min. An identical reaction was also carried out at 60°C. After zinc had completely precipitated, the supernatant was collected, passed through a 0.22 µm syringe filter, and transferred to a J-Young NMR tube. More solvent was passed through the same filter into the tube until the tube was filled with a volume of at least 0.5 mL.

In parallel, a blank sample containing only THF-d_8 and a reference sample containing **RAE-1** (35.5 mg, 0.10 mmol, 1.0 equiv.) in THF-d_8 (0.20 mL, c = 0.25 M) were prepared under the same conditions. All J-Young

tubes were removed from the glovebox and submitted for NMR analysis.

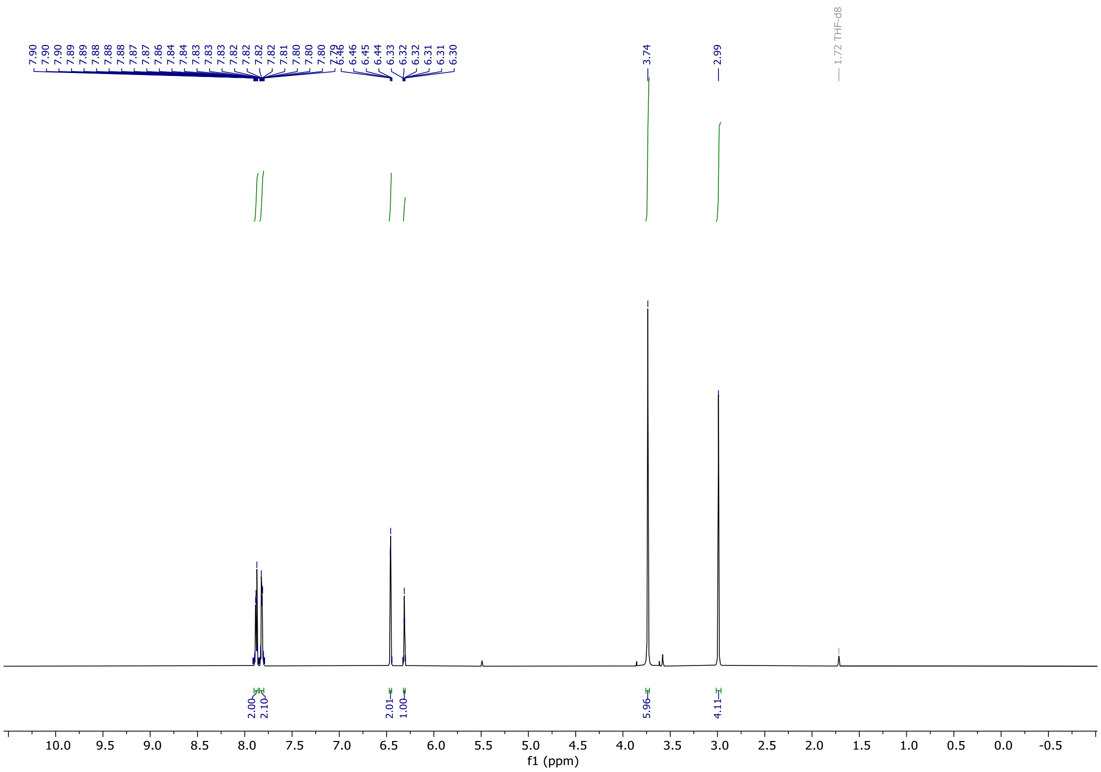

Figure S5. ^{1}H NMR of the reference sample contained only **RAE-1** in THF-d_8, the chemical shift of solvent residue is confirmed to be 1.72 ppm in the blank experiment.

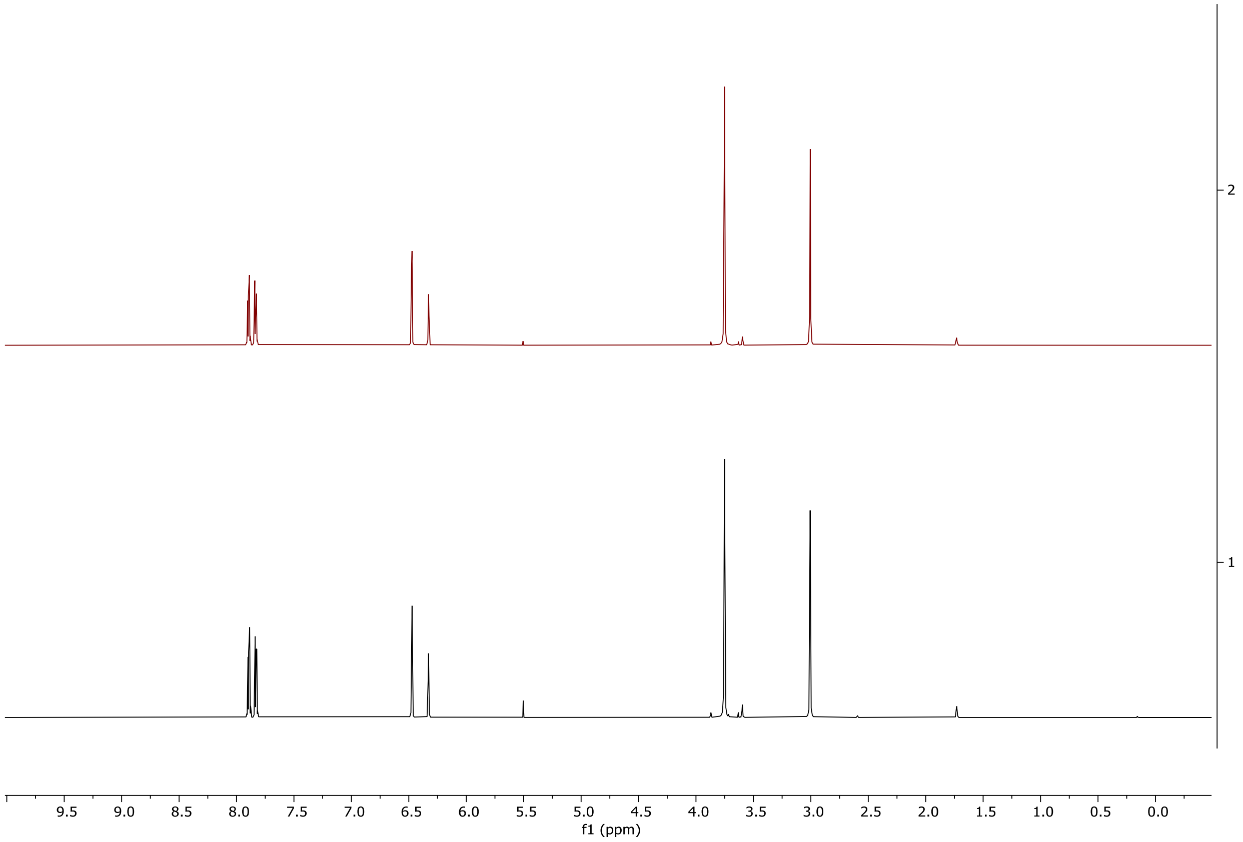

Figure S6. ^{1}H NMR of **RAE-1** after zinc addition 2hr under different temperature in THF-d_8, no trace for alkylzinc formation has been observed.

MS characterization of alkylzinc formation experiment in DMF

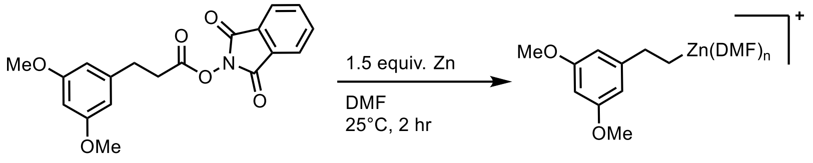

Under an ambient atmosphere, the redox-active ester **RAE-1** (35.5 mg, 0.10 mmol, 1.0 equiv.) was added to a 4-mL vial that contained a teflon-coated magnetic stir bar. The vial was transferred to a nitrogen-filled glovebox, where zinc powder (9.8 mg, 0.15 mmol, 1.5 equiv.) and DMF (0.20 mL, c = 0.25) were added. The reaction mixture was stirred (800 rpm) in the glovebox at 25°C on a stirring plate for 2 hours and then was allowed to stand for 5 minutes. After the zinc was completely precipitated, 20 µL of supernatant fluid was collected and put into a 2-mL GC vial, and the vial was taken out from the glovebox. Using a Discovery Comfort pipette from HTL Lab Solutions, 2 µL of the sample was moved to an argon filled Eppendorf tube containing 1 mL dry THF. A 250 µL Hamilton gastight #1725 syringe was loaded and the sample was measured by direct injection at a flow of 10 µL/min.

HRMS-ESI (m/z) calc'd for $C_{13}H_{20}NO_3Zn^+$ [M+DMF]$^+$, 302.0729; found, 302.0728; deviation: 0.3 ppm.

HRMS-ESI (m/z) calc'd for $C_{16}H_{27}N_2O_4Zn^+$ [M+2*DMF]$^+$, 375.1257; found, 375.1256; deviation: 0.2 ppm.

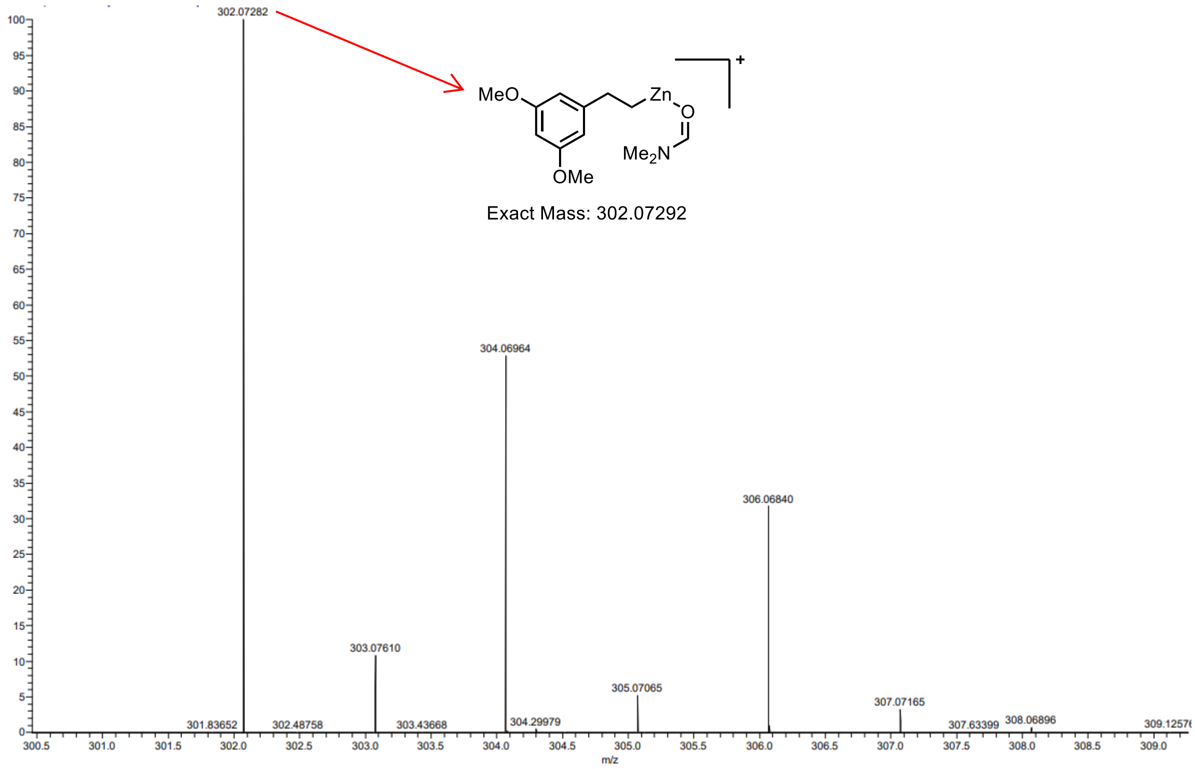

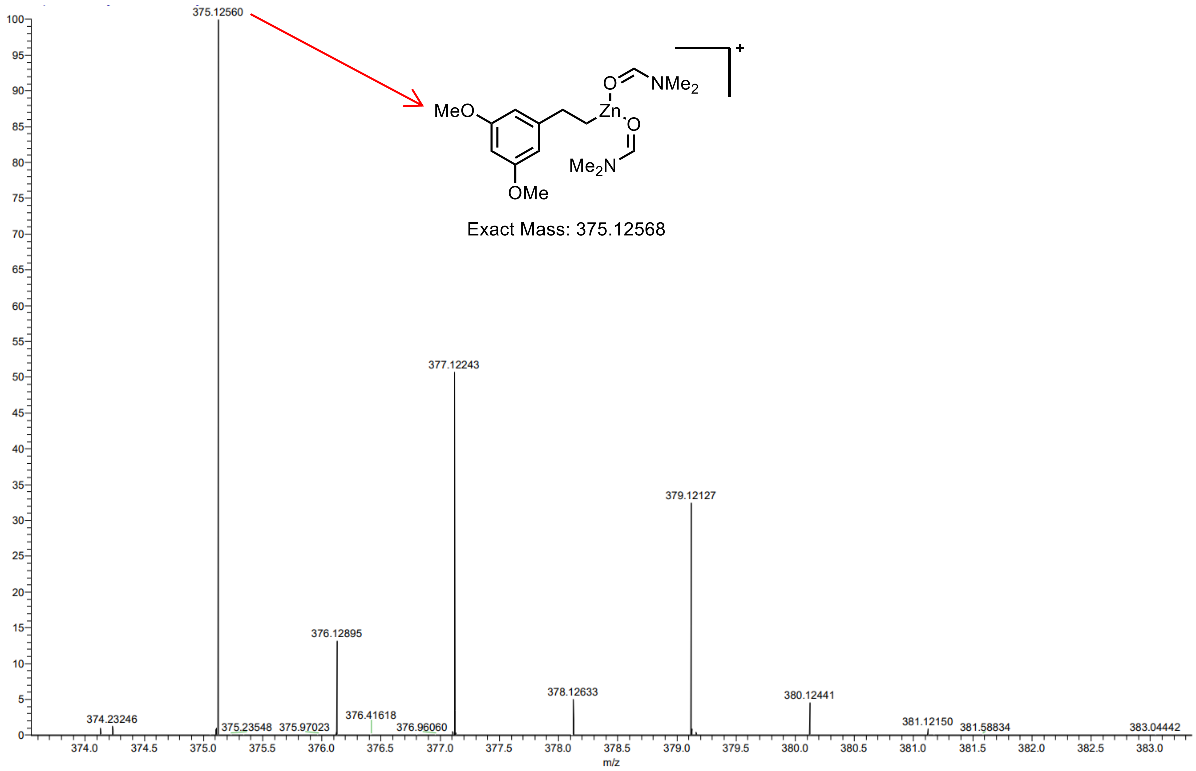

Figure S7. ESI-MS graph of **RAE-1** after zinc addition 2hr in DMF.

Alkylzinc quantification

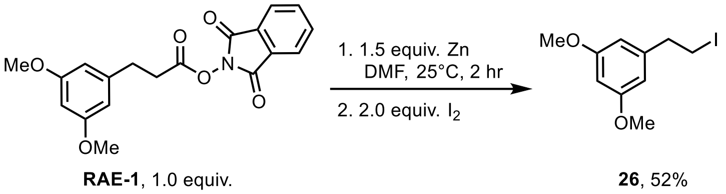

Under an ambient atmosphere, the redox-active ester **RAE-1** (35.5 mg, 0.10 mmol, 1.0 equiv.) was added to a 4-mL vial that contained a teflon-coated magnetic stir bar. The vial was transferred to a nitrogen-filled glovebox, where zinc powder (9.8 mg, 0.15 mmol, 1.5 equiv.) and DMF (0.20 mL, *c* = 0.25) were added. The reaction mixture was stirred (800 rpm) at 25°C on a stirring plate for 2 h. In a separate 4-mL vial, iodine (50.8 mg, 0.20 mmol, 2.0 equiv.) was added and the vial was transferred into the glovebox, then DMF (0.20 mL, *c* = 0.25) were added to dissolve the iodine. The resulting iodine solution was then added to the first vial, then the vial was sealed, and the reaction mixture was stirred (800 rpm) at 25°C for 2 minutes outside the glovebox. The reaction mixture was diluted with ethyl acetate (2 mL), and transferred to a separatory funnel that contained ethyl acetate (20 mL). The organic layer was washed with brine (1 × 25 mL). The aqueous layer was then extracted with ethyl acetate (3 × 20 mL). The organic layers were combined, dried over $MgSO_4$, filtered, and concentrated under reduced pressure. The resulting residue was purified by column chromatography on silica gel, eluting with EtOAc/hexanes (3:97, v/v) to afford the desired product **26** (15 mg, 52%) as colorless oil.

$\mathbf{R}_f$ = 0.35 (EtOAc/pentane, 5:95, v/v).

NMR Spectroscopy:

^{1}H NMR (600 MHz, $CDCl_3$, 23°C, δ): 6.37 (t, *J* = 2.3 Hz, 1H), 6.35 – 6.34 (m, 2H), 3.79 (s, 6H), 3.36 – 3.31 (m, 2H), 3.12 (t, *J* = 8.0 Hz, 2H).

^{13}C NMR (151 MHz, $CDCl_3$, 23°C, δ): 161.0, 143.0, 106.5, 98.8, 55.4, 40.8, 5.3.

HRMS-EI GC (m/z) calc'd for $C_{10}H_{13}O_2I$ $[M]^+$, 291.9955; found, 291.9960; deviation: –1.7 ppm.

Alkylzinc optimization

General procedure for optimization of alkylzinc formation

Under an ambient atmosphere, the redox-active ester **RAE-1** (35.5 mg, 0.10 mmol, 1.0 equiv.) was added to a 4-mL vial that contained a teflon-coated magnetic stir bar. The vial was transferred to a nitrogen-filled glovebox, where zinc powder (9.8 mg, 0.15 mmol, 1.5 equiv.) and DMF (0.20 mL, *c* = 0.25) were added. The reaction mixture was stirred (800 rpm) at 25°C on a stirring plate for 2 h. In a separate 4-mL vial, iodine (50.8 mg, 0.20 mmol, 2.0 equiv.) was added and the vial was transferred into the glovebox, then DMF (0.20 mL, *c* = 0.25) were added to dissolve the iodine. The resulting iodine solution was then added to the first vial, then the vial was sealed, and the reaction mixture was stirred (800 rpm) at 25°C for 2 minutes outside the glovebox. The reaction mixture was diluted with ethyl acetate (2 mL), and transferred to a separatory funnel that

contained ethyl acetate (20 mL). The organic layer was washed with brine (1 × 25 mL). The aqueous layer was then extracted with ethyl acetate (3 × 20 mL). The organic layers were combined, dried over $MgSO_4$, filtered, and concentrated under reduced pressure. Dibromomethane (CH_2Br_2) (7.0 μL, 17 mg, 0.10 mmol) was added, and the exact weight added was noted. $CDCl_3$ (0.5 mL) was added, and the resulting mixture was vigorously shaken for 10 seconds. Then, an aliquot of the mixture was taken and passed into an NMR tube through a Pasteur pipette that had been fitted with a piece of cotton to remove insoluble solids. More $CDCl_3$ was passed through the same Pasteur pipette into the NMR tube until the NMR tube was filled with a volume of at least 0.5 mL. Then, a ^1H NMR spectrum (NS = 16, D1 = 1 s, SW = 19.9875 ppm, O1P = 6.175 ppm, TD = 65536, O2 = 3089.62 Hz, O2P = 6.175 ppm) was measured, and the yield of **26** was determined by comparing the relative integrals of dibromomethane's proton signal (δ = 4.95 ppm, s, 2H) and two of **26**'s proton signals that are at the α-position of iodine (δ = 3.36 – 3.31 ppm, m, 2H).

Additive

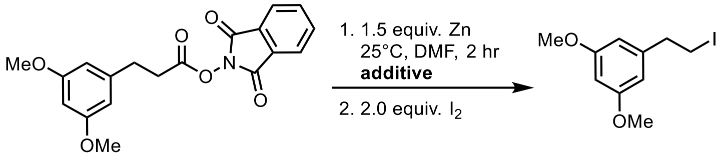

RAE-1, 1.0 equiv. **26**

Table S8. Screening of additives

Entry	additive	yield /%
**1**	none	52
**2**	$ZnCl_2$	52
**3**	ZnI_2	48
**4**	$Zn(OTf)_2$	48
**5**	I_2	60

Time

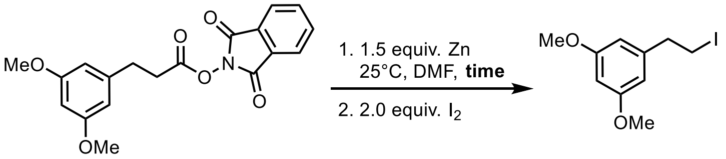

RAE-1, 1.0 equiv. **26**

Table S9. Screening of time

Entry	time	yield /%
**1**	0.5h	46
**2**	1h	49
**3**	1.5h	52
**4**	2h	52
**5**	3h	53
**6**	4h	52
**7**	6h	48

Solvent

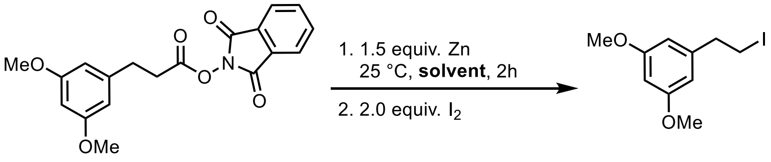

RAE-1, 1.0 equiv. **26**

Table S10. Screening of solvents

Entry	solvent	yield /%
**1**	DMF	52
**2**	DMA	42
**3**	THF	n.o.
**4**	MeCN	n.o.
**5**	1,4-dioxane	n.o.
**6**	Toluene	n.o.

n.o. = no product observed

Temperature

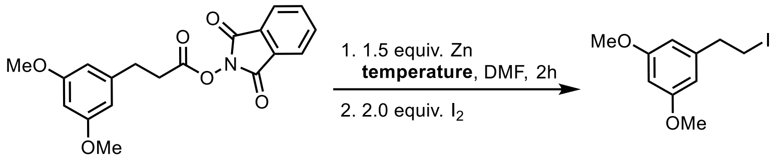

RAE-1, 1.0 equiv. **26**

Table S11. Screening of temperature

Entry	temperature	yield /%
**1**	25°C	52
**2**	40°C	50
**3**	60°C	44

Effect of multidentate ligands

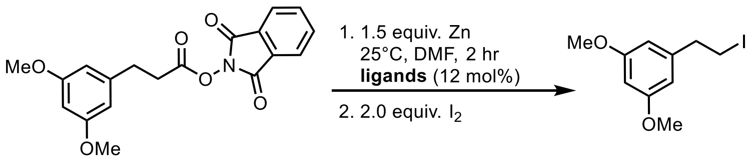

RAE-1, 1.0 equiv. **26**

Table S12. Screening of multidentate ligands additives

Entry	additive	yield /%
**1**	none	52
**2**	bpy	52
**3**	dtbbpy	48
**4**	tpy	43
**5**	dppbz	45

Reaction with alkylzinc in-situ formed from alkyl-iodide

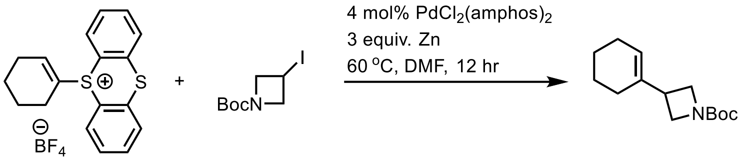

TT-21, 1.0 equiv. **R-1,** 2.2 equiv. **27**, 78%

Under an ambient atmosphere, *tert*-butyl 3-iodoazetidine-1-carboxylate (125 mg, 0.44 mmol, 2.2 equiv.) was added to a 4-mL vial that contained a teflon-coated magnetic stir bar. The vial was transferred to a nitrogen-filled glovebox, where zinc powder (39.2 mg, 0.60 mmol, 3.0 equiv.) and DMF (0.80 mL, *c* = 0.25) were added. The reaction mixture was stirred (800 rpm) at 60°C on a stirring plate for 2 h. In a separate 4-mL vial, $PdCl_2(amphos)_2$ (5.6 mg, 8.0 μmol, 4.0 mol%) and the alkenyl thianthrenium salt **TT-21** (77 mg, 0.20 mmol, 1.0 equiv.) were added under ambient atmosphere. The vial was transferred to a nitrogen-filled glovebox and DMF (0.80 mL, *c* = 0.25) was added. The mixture was stirred at 25°C for 5 min. After that, the obtained yellow suspension was added to the first vial. The vial was sealed, and the reaction mixture was stirred (800 rpm) at 60°C for 16 h. The reaction mixture was diluted with ethyl acetate (2 mL), and transferred to a separatory funnel that contained ethyl acetate (20 mL). The organic layer was washed with brine (1 × 25 mL). The aqueous layer was then extracted with ethyl acetate (3 × 20 mL). The organic layers were combined, dried over $MgSO_4$, filtered, and concentrated under reduced pressure. The resulting residue was purified by column chromatography on silica gel, eluting with EtOAc/hexanes (0:1 to 3:97, v/v) to afford the desired product **27** (37 mg, 78%) as colorless oil.

$\mathbf{R}_f$ = 0.55 (EtOAc/pentane, 1:9, v/v).

NMR Spectroscopy:

^{1}**H NMR** (500 MHz, $CDCl_3$, 23°C, δ): 5.53 – 5.50 (m, 1H), 3.97 (t, *J* = 8.5 Hz, 2H), 3.81 (dd, *J* = 8.4, 6.4 Hz, 2H), 3.14 – 3.05 (m, 1H), 2.04 – 2.00 (m, 2H), 1.96– 1.92 (m, 2H), 1.68 – 1.61 (m, 2H), 1.58 – 1.54 (m, 2H), 1.43 (s, 9H).

^{13}**C NMR** (151 MHz, $CDCl_3$, 23°C, δ): 156.6, 136.5, 122.1, 79.3, 35.2, 28.6, 25.5, 25.2, 22.8, 22.5.

HRMS-ESI (m/z) calc'd for $C_{14}H_{23}NO_2Na$ $[M+Na]^+$, 260.1621; found, 260.1622; deviation: –0.4 ppm.

Temperature screening for this reaction

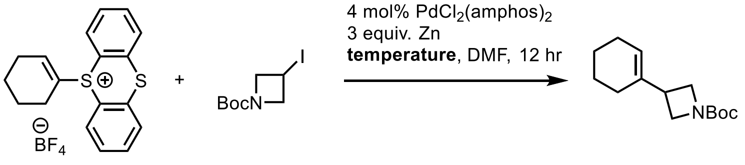

TT-21, 1.0 equiv. **R-1,** 2.2 equiv. **27**, yield

Table S13. Screening of temperature

Entry	Temperature	yield /%
**1**	60°C	78%
**2**	25°C	70%

Side product determination

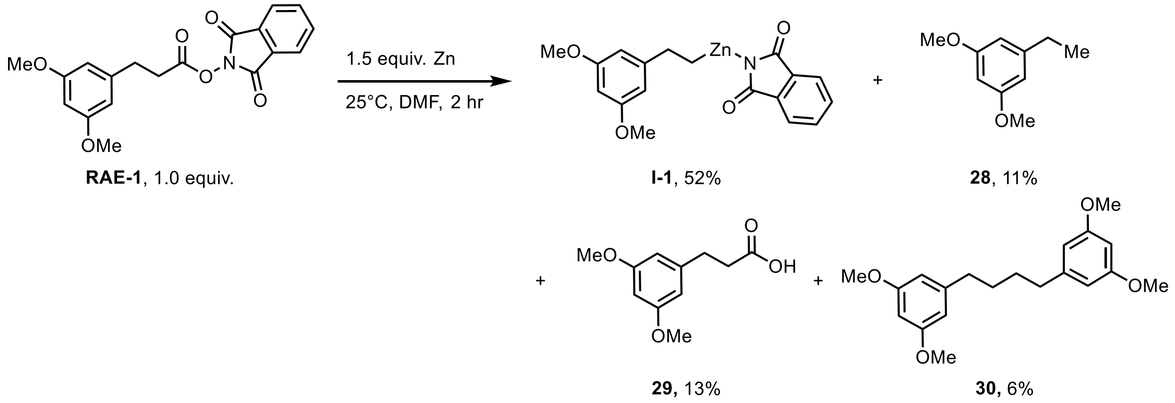

Characterization data of **28**

R*f* = 0.49 (EtOAc/pentane, 5:95, v/v).

NMR Spectroscopy:

^{1}H NMR (500 MHz, $CDCl_3$, 23°C, δ): 6.37 (d, *J* = 2.3 Hz, 2H), 6.30 (t, *J* = 2.4 Hz, 1H), 3.79 (s, 6H), 2.60 (q, *J* = 7.6 Hz, 2H), 1.23 (t, *J* = 7.6 Hz, 3H).

^{13}C NMR (126 MHz, $CDCl_3$, 23°C, δ): 160.9, 146.9, 106.1, 97.7, 55.4, 29.3, 15.6.

HRMS-EI GC (m/z) calc'd for $C_{10}H_{14}O_2$ $[M]^+$, 166.0988; found, 166.0989; deviation: –0.3 ppm.

Characterization data of **29**

R*f* = 0.32 (EtOAc/pentane, 20:80, v/v).

NMR Spectroscopy:

^{1}H NMR (500 MHz, $CDCl_3$, 23°C, δ): 6.39 (d, *J* = 2.2 Hz, 2H), 6.35 (t, *J* = 2.3 Hz, 1H), 3.80 (s, 6H), 2.93 (t, *J* = 7.8 Hz, 2H), 2.70 (t, *J* = 7.8 Hz, 2H).

^{13}C NMR (151 MHz, $CDCl_3$, 23°C, δ): 179.1, 161.0, 142.6, 106.4, 98.4, 55.4, 35.6, 31.0.

HRMS-EI GC (m/z) calc'd for $C_{11}H_{14}O_4Na$ $[M+Na]^+$, 210.0887; found, 210.0888; deviation: –0.9 ppm.

Characterization data of **30**

R*f* = 0.45 (DCM/pentane, 1:1, v/v).

NMR Spectroscopy:

^{1}H NMR (600 MHz, $CDCl_3$, 23°C, δ): 6.33 (dt, *J* = 2.3, 0.5 Hz, 4H), 6.29 (t, *J* = 2.3 Hz, 2H), 3.77 (s, 12H), 2.62 – 2.54 (m, 4H), 1.70 – 1.61 (m, 4H).

^{13}C NMR (151 MHz, $CDCl_3$, 23°C, δ): 160.8, 145.1, 106.6, 97.8, 55.4, 36.2, 30.9.

HRMS-ESI (m/z) calc'd for $C_{20}H_{26}O_4Na$ $[M+Na]^+$, 353.1723; found, 353.1728; deviation: –1.3 ppm.

Cyclic Voltammetry experiment

Cyclic voltammetry was performed using an Autolab PGSTAT204 potentiostat at a sweep rate of 100 mV/s. Solutions were made to contain 10 mM of the analyte and 100 mM Bu_4NBF_4 in DMF. The additives, $ZnCl_2$ or $ZnBr_2$ were also in 10 mM concentration. The sample was prepared in a vial equipped with a glassy carbon disk working electrode (3 mm diameter, purchased from BASi), Pt wire counter electrode (purchased from BASi) and an $AgNO_3$/Ag reference electrode (purchased from BASi). Before data collection, each solution was stirred vigorously and sparged with argon for 10 minutes to remove dissolved oxygen.

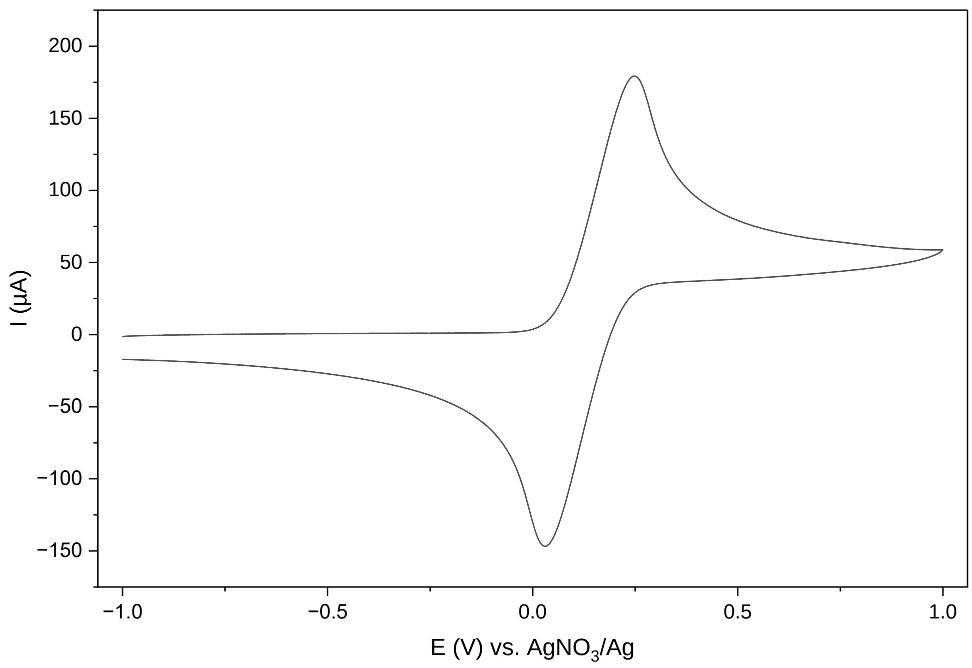

Figure S8. Cyclovoltammogram of ferrocene in DMF, E_p = 0.25 V.

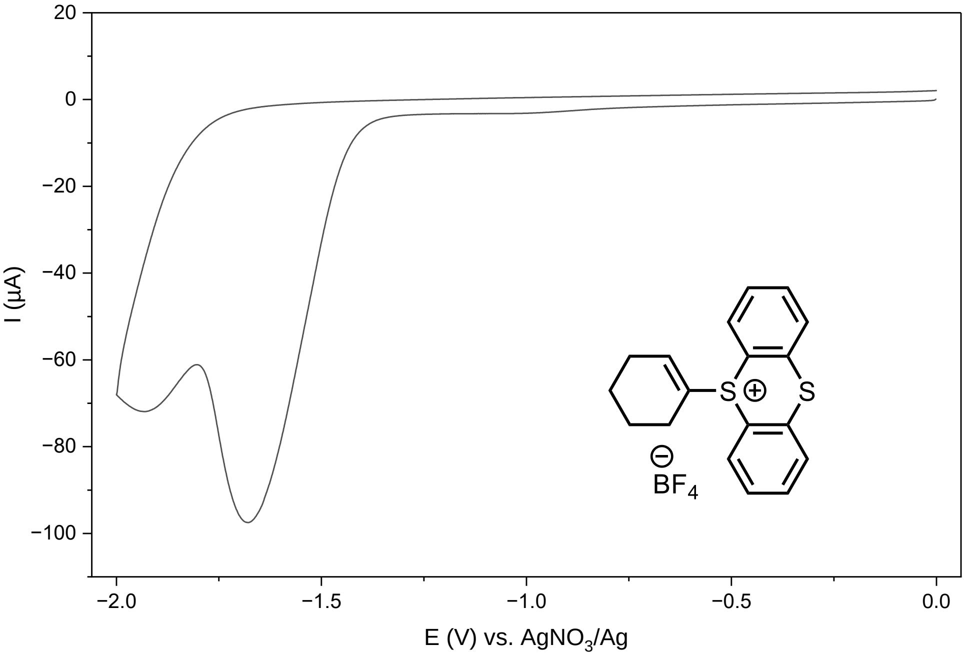

Figure S9. Cyclovoltammogram of thianthrenium salt **TT-21** in DMF, E_p = -1.68 V.

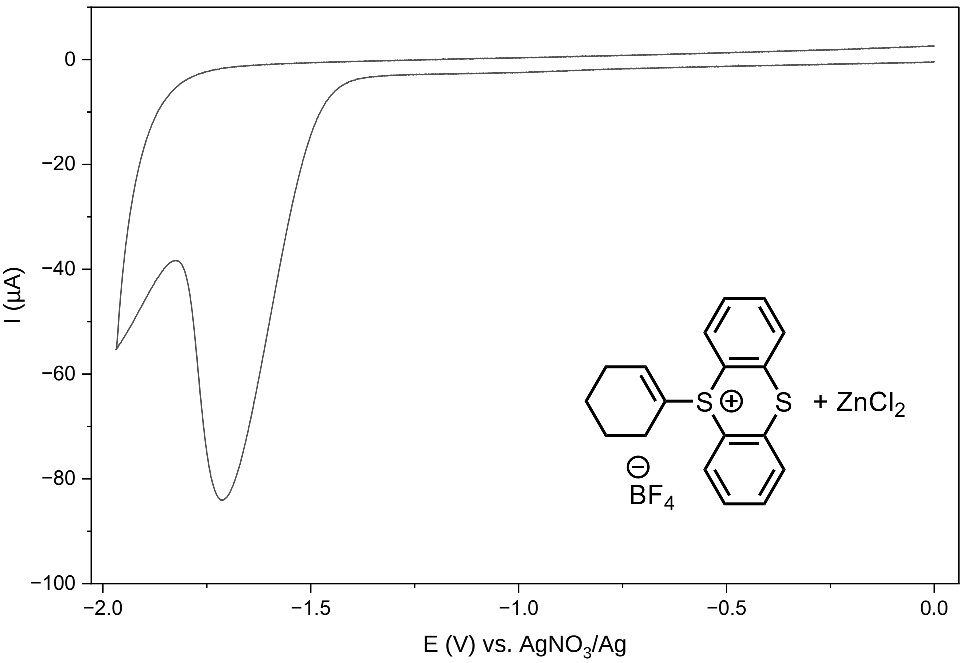

Figure S10. Cyclovoltammogram of thianthrenium salt **TT-21** and $ZnCl_2$ in DMF, E_p = -1.72 V.

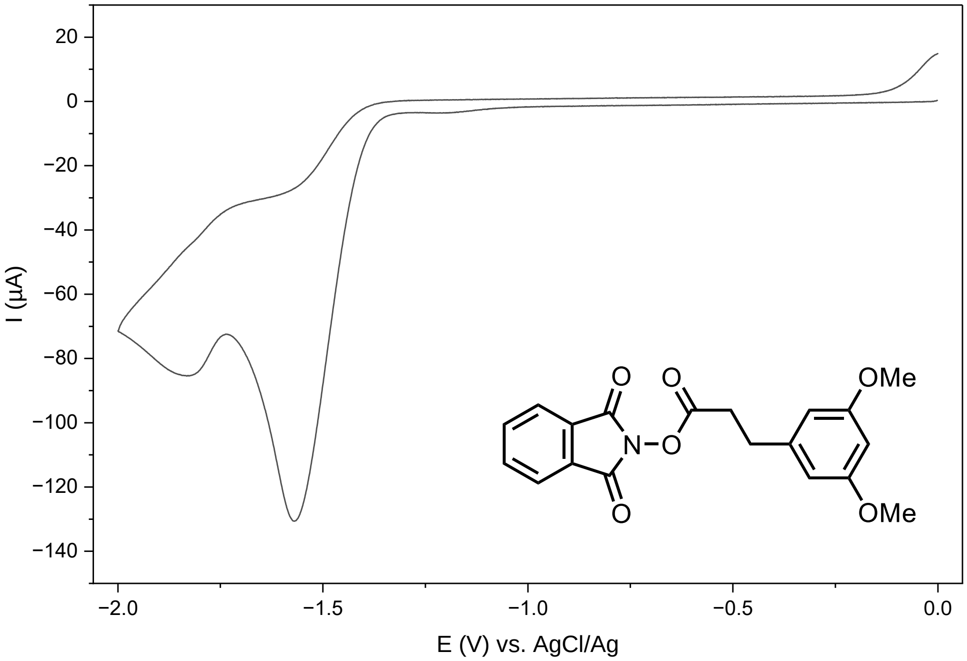

Figure S11. Cyclovoltammogram of redox-active ester **RAE-1** in DMF, E_p = -1.59 V

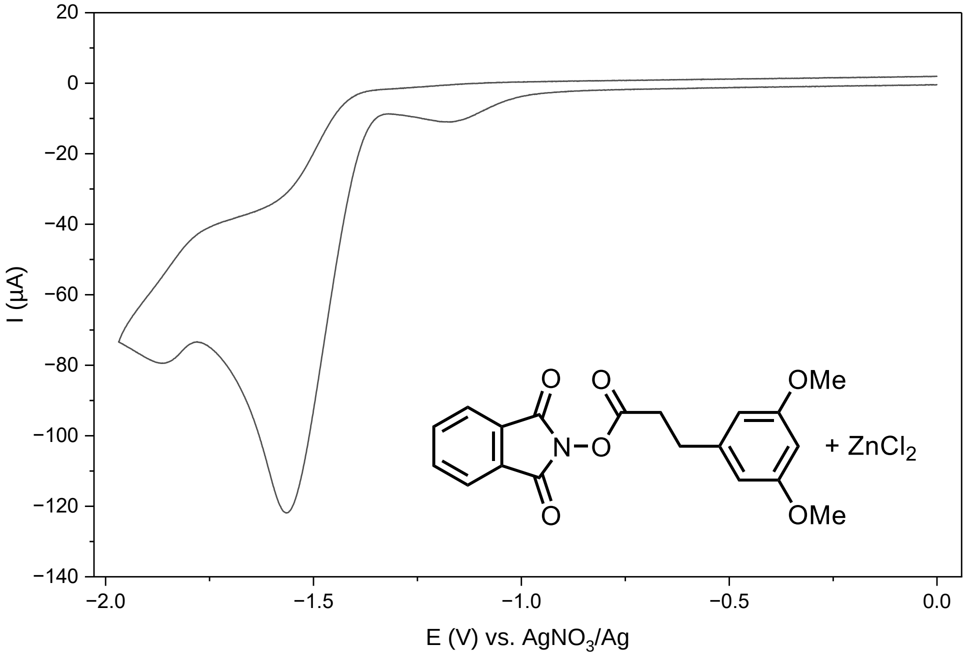

Figure S12. Cyclovoltammogram of redox-active ester **RAE-1** and $ZnCl_2$ in DMF, E_p = -1.56 V

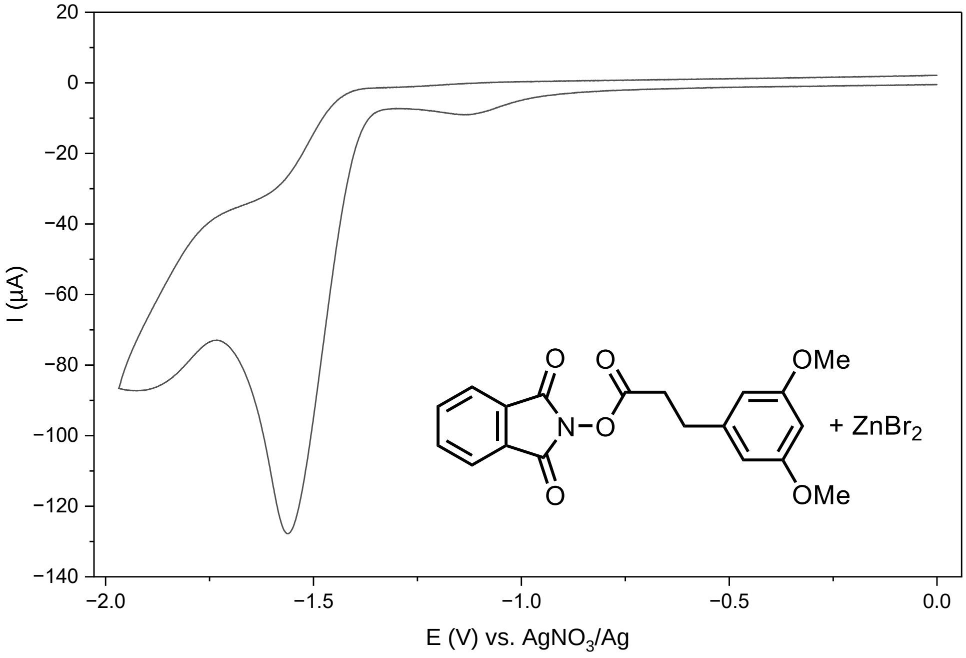

Figure S13. Cyclovoltammogram of redox-active ester **RAE-1** and $ZnBr_2$ in DMF, E_p = -1.56 V

Kinetics study of alkylzinc formation

Kinetics study of alkylzinc formation under standard condition

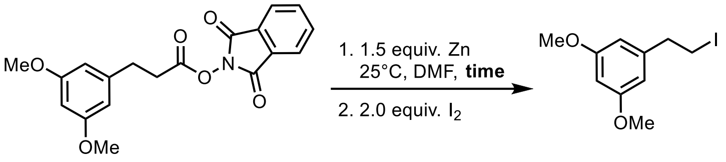

RAE-1, 1.0 equiv. **26**

Under an ambient atmosphere, the redox-active ester **RAE-1** (35.5 mg, 0.10 mmol, 1.0 equiv.) was added to a 4-mL vial that contained a teflon-coated magnetic stir bar. The vial was transferred to a nitrogen-filled glovebox, where zinc powder (9.8 mg, 0.15 mmol, 1.5 equiv.) and DMF (0.20 mL, *c* = 0.25) were added. The reaction mixture was stirred (800 rpm) at 25°C on a stirring plate. In a separate 4-mL vial, iodine (50.8 mg, 0.20 mmol, 2.0 equiv.) was added and the vial was transferred into the glovebox, then DMF (0.20 mL, *c* = 0.25) were added to dissolve the iodine. The resulting iodine solution was then added to the first vial, then the vial was sealed, and the reaction mixture was stirred (800 rpm) at 25°C for 2 minutes outside the glovebox. The reaction mixture was diluted with ethyl acetate (2 mL), and transferred to a separatory funnel that contained ethyl acetate (20 mL). The organic layer was washed with brine (1 × 25 mL). The aqueous layer was then extracted with ethyl acetate (3 × 20 mL). The organic layers were combined, dried over $MgSO_4$, filtered, and concentrated under reduced pressure. Dibromomethane (CH_2Br_2) (7.0 μL, 17 mg, 0.10 mmol) was added, and the exact weight added was noted. $CDCl_3$ (0.5 mL) was added, and the resulting mixture was vigorously shaken for 10 seconds. Then, an aliquot of the mixture was taken and passed into an NMR tube

through a Pasteur pipette that had been fitted with a piece of cotton to remove insoluble solids. More $CDCl_3$ was passed through the same Pasteur pipette into the NMR tube until the NMR tube was filled with a volume of at least 0.5 mL. Then, a ^{1}H NMR spectrum (NS = 16, D1 = 1 s, SW = 19.9875 ppm, O1P = 6.175 ppm, TD = 65536, O2 = 3089.62 Hz, O2P = 6.175 ppm) was measured, and the yield of **26** was determined by comparing the relative integrals of dibromomethane's proton signal (δ = 4.95 ppm, s, 2H) and two of **26**'s proton signals that are at the α-position of iodine (δ = 3.36 – 3.31 ppm, m, 2H).

Table S14. Result of kinetic study under standard condition

Entry	time	yield /%
**1**	0 min	0
**2**	2 min	4
**3**	5 min	6
**4**	10 min	13
**5**	15 min	34
**6**	20 min	37
**7**	25 min	42
**8**	30 min	46
**9**	35 min	49
**10**	40 min	50

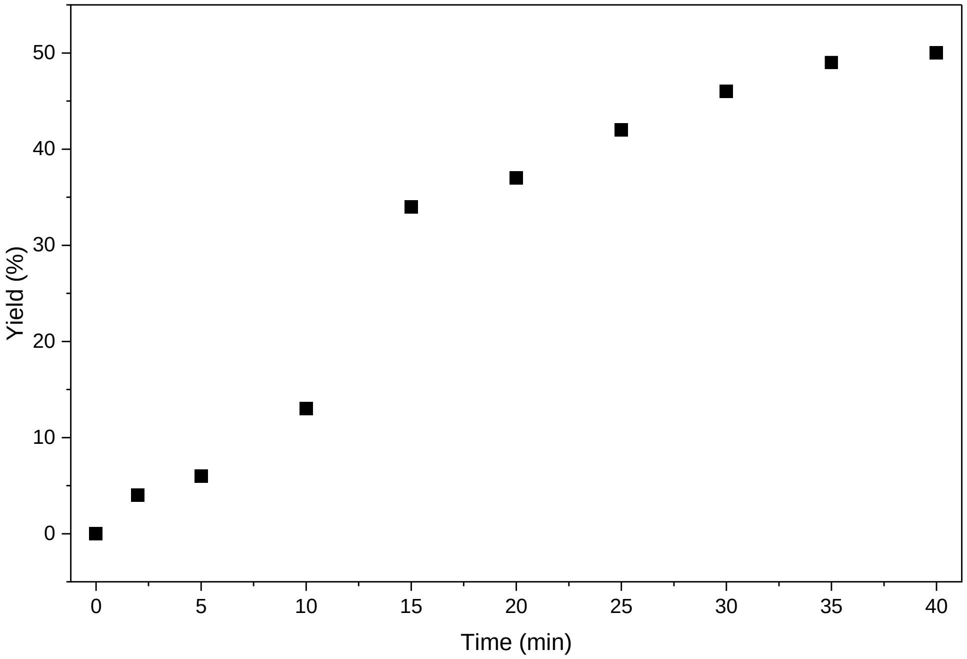

Figure S14. Kinetic reaction profile of alkylzinc formation under standard condition.

Kinetics study of alkylzinc formation in the presence of diethylzinc additive

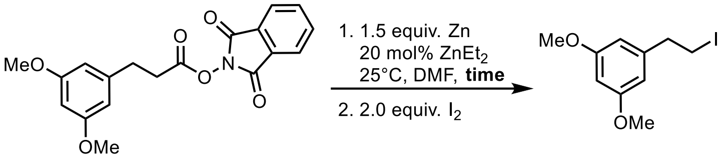

RAE-1, 1.0 equiv. **26**

Under an ambient atmosphere, the redox-active ester **RAE-1** (35.5 mg, 0.10 mmol, 1.0 equiv.) was added to a 4-mL vial that contained a teflon-coated magnetic stir bar. The vial was transferred to a nitrogen-filled glovebox, where zinc powder (9.8 mg, 0.15 mmol, 1.5 equiv.), diethylzinc (2.1 μL, 2.5 mg, 0.020 mmol, 20 mol%) and DMF (0.20 mL, c = 0.25) were added. The reaction mixture was stirred (800 rpm) at 25°C on a stirring plate. In a separate 4-mL vial, iodine (50.8 mg, 0.20 mmol, 2.0 equiv.) was added and the vial was transferred into the glovebox, then DMF (0.20 mL, c = 0.25) were added to dissolve the iodine. The resulting iodine solution was then added to the first vial, then the vial was sealed, and the reaction mixture was stirred (800 rpm) at 25°C for 2 minutes outside the glovebox. The reaction mixture was diluted with ethyl acetate (2 mL), and transferred to a separatory funnel that contained ethyl acetate (20 mL). The organic layer was washed with brine (1 × 25 mL). The aqueous layer was then extracted with ethyl acetate (3 × 20 mL). The organic layers were combined, dried over $MgSO_4$, filtered, and concentrated under reduced pressure. Dibromomethane (CH_2Br_2) (7.0 μL, 17 mg, 0.10 mmol) was added, and the exact weight added was noted. $CDCl_3$ (0.5 mL) was added, and the resulting mixture was vigorously shaken for 10 seconds. Then, an aliquot of the mixture was taken and passed into an NMR tube through a Pasteur pipette that had been fitted with a

piece of cotton to remove insoluble solids. More $CDCl_3$ was passed through the same Pasteur pipette into the NMR tube until the NMR tube was filled with a volume of at least 0.5 mL. Then, a ^{1}H NMR spectrum (NS = 16, D1 = 1 s, SW = 19.9875 ppm, O1P = 6.175 ppm, TD = 65536, O2 = 3089.62 Hz, O2P = 6.175 ppm) was measured, and the yield of **26** was determined by comparing the relative integrals of dibromomethane's proton signal (δ = 4.95 ppm, s, 2H) and two of **26**'s proton signals that are at the α-position of iodine (δ = 3.36 – 3.31 ppm, m, 2H)

Table S15. Result of kinetic study in the presence of diethylzinc additive

Entry	time	yield /%
**1**	0 min	0
**2**	2 min	15
**3**	5 min	35
**4**	8 min	39
**5**	10 min	49
**6**	15 min	51
**7**	20 min	51
**8**	25 min	51
**9**	30 min	52
**10**	35 min	53
**11**	40 min	55

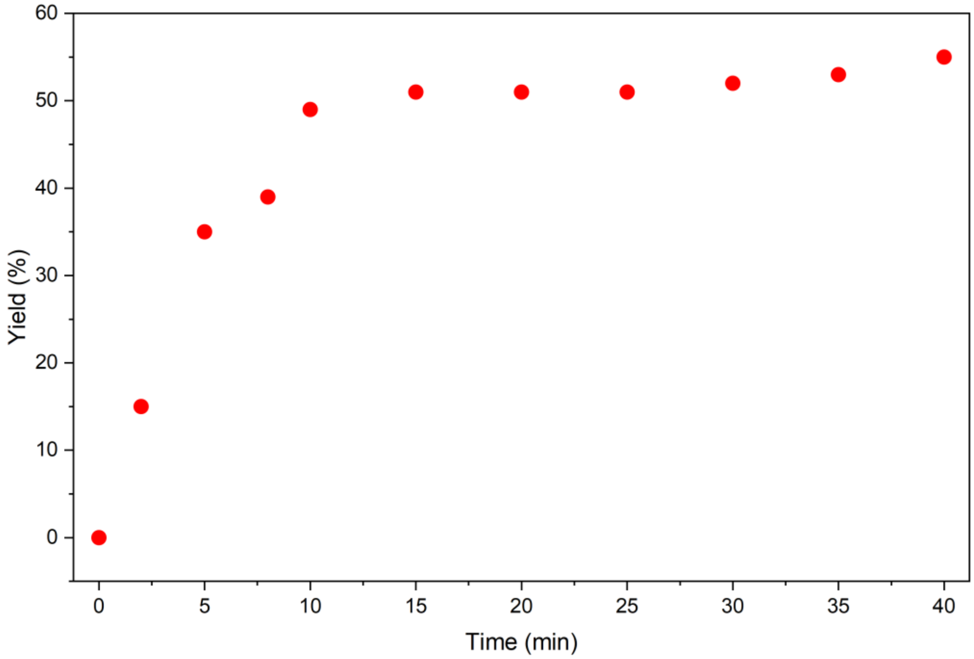

Figure S15. Kinetic reaction profile of alkylzinc formation in the presence of diethylzinc additive.

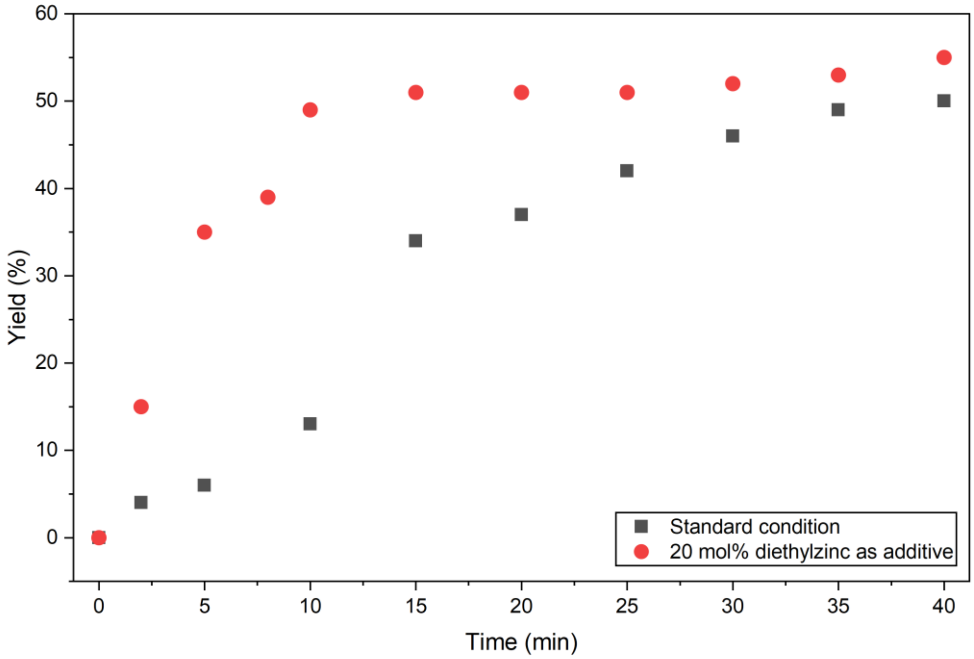

Figure S16. Comparison of kinetic reaction profile of alkylzinc formation with and without diethylzinc as an additive.

Thianthrenium salt reduction experiment

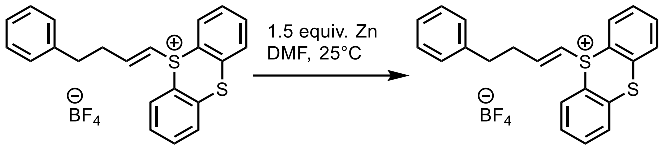

TT-2, 1.00 equiv. **TT-2**, 65% recovered

Under an ambient atmosphere, the thianthrenium salt **TT-2** (43.4 mg, 0.10 mmol, 1.0 equiv.) was added to a 4-mL vial that contained a teflon-coated magnetic stir bar. The vial was transferred to a nitrogen-filled glovebox, where zinc powder (9.8 mg, 0.15 mmol, 1.5 equiv.) and DMF (0.20 mL, *c* = 0.25) were added. The reaction was stirred in a glovebox for 2 hours. Subsequently, the reaction mixture was diluted with ethyl acetate (2 mL), and transferred to a separatory funnel that contained ethyl acetate (20 mL). The organic layer was washed with brine (1 × 25 mL). The aqueous layer was then extracted with ethyl acetate (3 × 20 mL). The organic layers were combined, dried over $MgSO_4$, filtered, and concentrated under reduced pressure organic layers were combined, dried over $MgSO_4$, filtered, and concentrated under reduced pressure. Dibromomethane (CH_2Br_2) (7.0 µL, 17 mg, 0.10 mmol) was added, and the exact weight added was noted. $CDCl_3$ (0.5 mL) was added, and the resulting mixture was vigorously shaken for 10 seconds. Then, an aliquot of the mixture was taken and passed into an NMR tube through a Pasteur pipette that had been fitted with a piece of cotton to remove insoluble solids. More $CDCl_3$ was passed through the same Pasteur pipette into the NMR tube until the NMR tube was filled with a volume of at least 0.5 mL. Then, a ^{1}H NMR spectrum (NS = 16, D1 = 1 s, SW = 19.9875 ppm, O1P = 6.175 ppm, TD = 65536, O2 = 3089.62 Hz, O2P = 6.175 ppm) was measured.

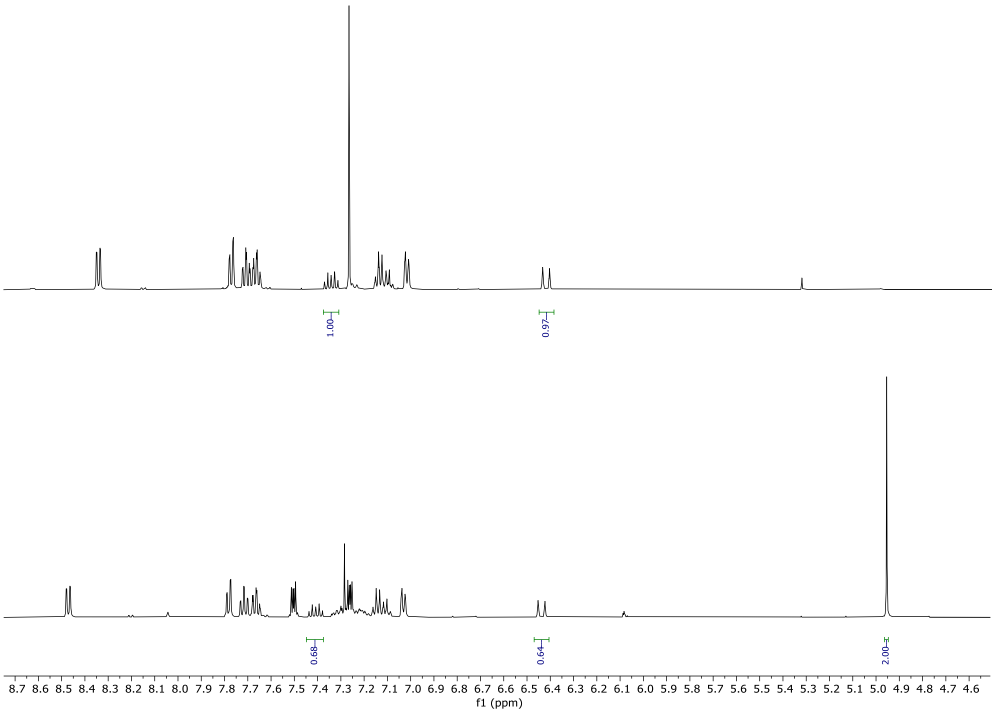

Figure S17. ^{1}H NMR of **TT-2** before and after zinc addition 2hr.

Synthesis of alkenyl thianthrenium salts

Methyl pent-4-enoate derived thianthrenium salt TT-1

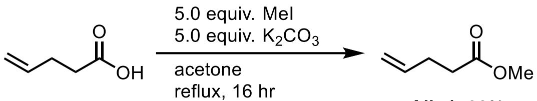

Alk-2, 1.0 equiv. **Alk-1**, 88%

Prepared according to a reported procedure and spectra are in good accordance with literature[3].

Pent-4-enoic acid (2.0 g, 2.0 mL, 20 mmol, 1.0 equiv.) and potassium carbonate (13.8 g, 0.10 mol, 5.0 equiv.) were dissolved in acetone (15.0 g, 19.1 mL, 0.260 mol, 13.0 equiv.). To this suspension, iodomethane (14.2 g, 6.22 mL, 0.10 mol, 5.0 equiv.) was added dropwise at room temperature. This suspension was refluxed at 60°C overnight. The reaction mixture was allowed to cool down to room temperature, diluted with diethyl ether. The organic layer was washed with saturated ammonium chloride solution, dried over Na_2SO_4, concentrated at reduced pressure to afford **Alk-1** (2.0 g, 88%) as a colorless oil.

R*f* = 0.61 (EtOAc/pentane, 1:9, v/v)

NMR Spectroscopy:

^{1}H NMR (600 MHz, $CDCl_3$, 23°C, δ): 5.86 – 5.75 (m, 1H), 5.08 – 4.93 (m, 2H), 3.65 (s, 3H), 2.42 – 2.38 (m, 2H), 2.38 – 2.33 (m, 2H).

^{13}C NMR (151 MHz, $CDCl_3$, 23°C, δ): 173.64, 136.75, 115.59, 51.64, 33.43, 28.94.

HRMS-ESI (m/z) calc'd for $C_6H_{11}O_2$ $[M+H]^+$, 115.0754; found, 115.0754; deviation: –0.2 ppm.

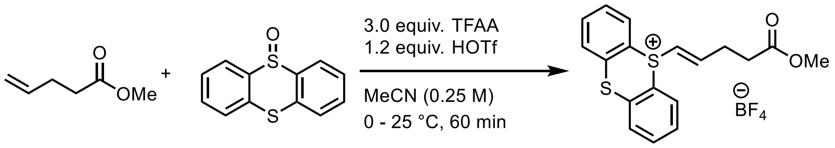

Alk-1, 1.0 equiv. **TTO**, 1.03 equiv. **TT-1**, 73%

Under ambient atmosphere, a 20 mL borosilicate vial equipped with a magnetic stir bar was charged with methyl pent-4-enoate (65 µL, 57 mg, 0.50 mmol, 1.0 equiv.), thianthrene S-oxide (**TTO**) (120 mg, 0.515 mmol, 1.03 equiv.), and MeCN (2.0 mL, c = 0.25 M). After cooling to 0°C, trifluoroacetic anhydride (0.21 mL, 0.32 g, 1.50 mmol, 3.0 equiv.) was added dropwise within 30 seconds, followed by dropwise addition of HOTf (53 µL, 90 mg, 0.60 mmol, 1.2 equiv.) within 10 seconds. After stirring the lilac mixture at 0°C for 60 min followed by stirring at 25°C for 30 min, the resulting yellow mixture was concentrated under reduced pressure and subsequently diluted with DCM (10 mL). The DCM solution was poured onto a saturated aqueous $NaHCO_3$ solution (ca. 20 mL). The combined mixture was poured into a separatory funnel, and the layers were separated. The DCM layer was collected, and the aqueous layer was further extracted with DCM (2 × ca. 10 mL). The combined DCM phase was washed with aqueous $NaBF_4$ solution (2 × ca. 20 mL, 5% w/w). The DCM layer was dried over Na_2SO_4 (10 g), filtered, and the solvent was removed under reduced pressure.

The residue was purified by chromatography on silica gel, eluting with DCM/MeOH (97:3, v/v). The product-containing fractions were collected and concentrated under reduced pressure. The residue was further dried in vacuo to afford **TT-1** (*E:Z* > 20:1, 152 mg, 73%) as a colorless solid.

R*f* = 0.42 (DCM/MeOH, 19:1, v/v)

NMR Spectroscopy:

^{1}H NMR (500 MHz, $CDCl_3$, 23°C, δ): 8.33 (d, *J* = 7.9 Hz, 2H), 7.83 (d, *J* = 7.9 Hz, 2H), 7.74 (t, *J* = 7.6 Hz, 2H), 7.67 (t, *J* = 7.7 Hz, 2H), 7.25 – 7.21 (m, 1H), 6.63 (d, *J* = 14.8 Hz, 1H), 3.58 (s, 3H), 2.60 – 2.55 (m, 2H), 2.50 – 2.47 (m, 2H).

^{13}C NMR (151 MHz, $CDCl_3$, 23°C, δ): 172.4, 155.1, 135.8, 134.8, 134.1, 130.7, 130.4, 120.8, 111.0, 52.2, 31.6, 28.6.

^{19}F NMR (565 MHz, $CDCl_3$, 23°C, δ): –150.60 (bs), –150.66 (bs).

HRMS-ESI (m/z) calc'd for $C_{18}H_{17}O_2S_2$ [M-BF_4]$^+$, 329.0665; found, 329.0664; deviation: 0.3 ppm.

4-Phenyl-1-butene derived thianthrenium salt TT-2

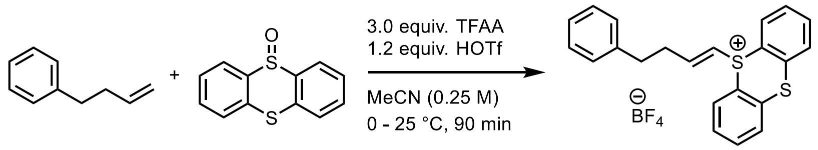

Alk-3, 1.0 equiv. **TTO**, 1.03 equiv. **TT-2**, 75%

Prepared according to a reported procedure and spectra are in good accordance with literature[4].

Under ambient atmosphere, a 20 mL borosilicate vial equipped with a magnetic stir bar was charged with 4-phenyl-1-butene (75 µL, 66 mg, 0.50 mmol, 1.0 equiv.), thianthrene *S*-oxide (**TTO**) (120 mg, 0.517 mmol, 1.03 equiv.), and MeCN (2.0 mL, *c* = 0.25 M). After cooling to 0°C, trifluoroacetic anhydride (0.21 mL, 0.31 g, 1.5 mmol, 3.0 equiv.) was added dropwise within 30 seconds, followed by dropwise addition of HOTf (52 µL, 88 mg, 0.59 mmol, 1.2 equiv.) within 10 seconds. After stirring the lilac mixture at 0°C for 60 min followed by stirring at 25°C for 30 min, the resulting purple mixture was concentrated under reduced pressure and subsequently diluted with DCM (10 mL). The DCM solution was poured onto a saturated aqueous $NaHCO_3$ solution (ca. 20 mL). The combined mixture was poured into a separatory funnel, and the layers were separated. The DCM layer was collected, and the aqueous layer was further extracted with DCM (2 × ca. 10 mL). The combined DCM phase was washed with aqueous $NaBF_4$ solution (2 × ca. 20 mL, 5% w/w). The DCM layer was dried over Na_2SO_4 (10 g), filtered, and the solvent was removed under reduced pressure. The residue was purified by chromatography on silica gel, eluting with DCM/MeOH (98:2, v/v). The product-containing fractions were collected and concentrated under reduced pressure. The residue was further dried in vacuo to afford **TT-2** (*E:Z* = 22:1, 163 mg, 75%) as a colorless solid.

R*f* = 0.42 (DCM/MeOH, 9:1, v/v).

NMR Spectroscopy:

^{1}H NMR (600 MHz, CD_2Cl_2, 23°C, δ): 8.16 (ddd, *J* = 7.9, 1.4, 0.5 Hz, 2H), 7.85 (ddd, *J* = 7.9, 1.4, 0.5 Hz, 2H), 7.76 (ddd, *J* = 7.9, 7.4, 1.4 Hz, 2H), 7.68 (ddd, *J* = 8.0, 7.4, 1.4 Hz, 2H), 7.18 – 7.14 (m, 2H), 7.13 – 7.09 (m, 2H), 7.06 – 7.02 (m, 2H), 6.41 (dt, *J* = 14.8, 1.4 Hz, 1H), 2.76 (t, *J* = 7.4 Hz, 2H), 2.60 (qdd, *J* = 7.3, 1.4, 0.5 Hz, 2H).

^{13}C NMR (151 MHz, CD_2Cl_2, 23°C, δ): 155.9, 139.9, 136.0, 134.8, 133.5, 130.6, 130.6, 128.9, 128.8, 126.7, 120.7, 110.6, 35.1, 33.6.

^{19}F NMR (565 MHz, CD_2Cl_2, 23°C, δ): –151.10 (bs), –151.1 (bs).

HRMS-ESI (m/z) calc'd for $C_{22}H_{19}S_2^+$ $[M\text{-}BF_4]^+$, 347.0923; found, 347.0923; deviation: –0.1 ppm.

Cycloheptene derived thianthrenium salt TT-3

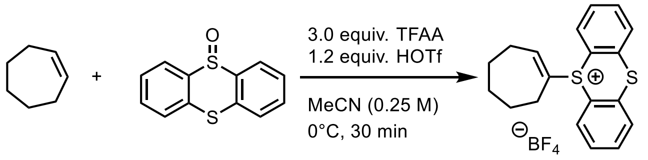

Alk-4, 1.0 equiv. **TTO**, 1.03 equiv. **TT-3**, 75%

Prepared according to a reported procedure and spectra are in good accordance with literature[4].

Under ambient atmosphere, a 20 mL borosilicate vial equipped with a magnetic stir bar was charged with cycloheptene (59 µL, 49 mg, 0.50 mmol, 1.0 equiv.), thianthrene *S*-oxide (**TTO**) (120 mg, 0.517 mmol, 1.03 equiv.), and MeCN (2.0 mL, *c* = 0.25 M). After cooling to 0°C, trifluoroacetic anhydride (0.21 mL, 0.31 g, 1.5 mmol, 3.0 equiv.) was added dropwise within 30 seconds, followed by dropwise addition of HOTf (52 µL, 88 mg, 0.59 mmol, 1.2 equiv.) within 10 seconds. After stirring the lilac mixture at 0°C for 30 min, the resulting pink mixture was concentrated under reduced pressure and subsequently diluted with DCM (10 mL). The DCM solution was poured onto a saturated aqueous $NaHCO_3$ solution (ca. 20 mL). The combined mixture was poured into a separatory funnel, and the layers were separated. The DCM layer was collected, and the aqueous layer was further extracted with DCM (2 × ca. 10 mL). The combined DCM phase was washed with aqueous $NaBF_4$ solution (2 × ca. 20 mL, 5% w/w). The DCM layer was dried over Na_2SO_4 (10 g), filtered, and the solvent was removed under reduced pressure. The residue was purified by chromatography on silica gel, eluting with DCM/MeOH (98:2, v/v). The product-containing fractions were collected and concentrated under reduced pressure. The residue was further dried in vacuo to afford **TT-3** (149 mg, 75%) as a colorless solid.

R_f = 0.46 (DCM/MeOH, 15:1, v/v)

NMR Spectroscopy:

^{1}H NMR (500 MHz, CD_2Cl_2, 23°C, δ): 8.21 (dd, *J* = 7.8, 1.5 Hz, 2H), 7.88 (dd, *J* = 7.9, 1.5 Hz, 2H), 7.82 (td, *J* = 7.7, 1.4 Hz, 2H), 7.75 (td, *J* = 7.7, 1.4 Hz, 2H), 5.92 (t, *J* = 6.6 Hz, 1H), 2.33 – 2.24 (m, 2H), 1.72 – 1.65 (m, 2H), 1.60 – 1.54 (m, 2H), 1.50 (p, *J* = 5.6 Hz, 2H), 1.39 – 1.30 (m, 2H).

^{13}C NMR (126 MHz, CD_2Cl_2, 23°C, δ): 145.0, 137.0, 135.2, 135.2, 130.6, 130.5, 124.5, 117.5, 30.8, 30.5,

29.8, 25.9, 25.3.

^{19}F NMR (471 MHz, CD_2Cl_2, 23°C, δ): –151.88 (bs), –151.94 (bs).

HRMS-ESI (m/z) calc'd for $C_{19}H_{19}S_2^+$ [M-BF_4]$^+$, 311.0923; found, 311.0922; deviation: 0.2 ppm.

Oct-1-en-7-yne derived thianthrenium salt TT-4

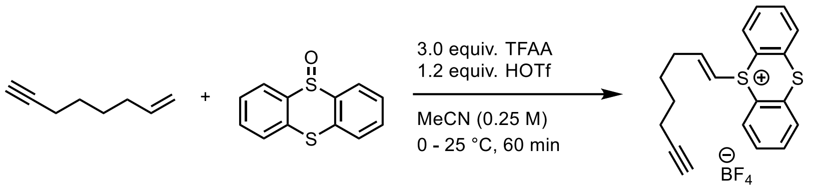

Under ambient atmosphere, a 20 mL borosilicate vial equipped with a magnetic stir bar was charged with Oct-1-en-7-yne (54.1 mg, 0.50 mmol, 1.0 equiv.), thianthrene S-oxide (**TTO**) (120 mg, 0.515 mmol, 1.03 equiv.), and MeCN (2.0 mL, *c* = 0.25 M). After cooling to 0°C, trifluoroacetic anhydride (0.21 mL, 0.32 g, 1.5 mmol, 3.0 equiv.) was added dropwise within 30 seconds, followed by dropwise addition of HOTf (52 µL, 90 mg, 0.60 mmol, 1.2 equiv.) within 10 seconds. After stirring the lilac mixture at 0°C for 60 min followed by stirring at 25°C for 30 min, the resulting yellow mixture was concentrated under reduced pressure and subsequently diluted with DCM (10 mL). The DCM solution was poured onto a saturated aqueous $NaHCO_3$ solution (ca. 20 mL). The combined mixture was poured into a separatory funnel, and the layers were separated. The DCM layer was collected, and the aqueous layer was further extracted with DCM (2 × ca. 10 mL). The combined DCM phase was washed with aqueous $NaBF_4$ solution (2 × ca. 20 mL, 5% w/w). The DCM layer was dried over Na_2SO_4 (10 g), filtered, and the solvent was removed under reduced pressure. The residue was purified by chromatography on silica gel, eluting with DCM/MeOH (98:2, v/v). The product-containing fractions were collected and concentrated under reduced pressure. The residue was further dried in vacuo to afford **TT-4** (*E:Z* = 14:1, 146 mg, 71%) as a colorless oil.

R_f = 0.33 (DCM/MeOH, 9:1, v/v).

NMR Spectroscopy:

^{1}H NMR (600 MHz, CD_3CN, 23°C, δ): 8.18 – 8.07 (m, 2H), 8.01 – 7.90 (m, 2H), 7.86 – 7.77 (m, 2H), 7.77 – 7.68 (m, 2H), 6.83 (dt, *J* = 14.9, 7.1 Hz, 1H), 6.51 (dt, *J* = 14.8, 1.5 Hz, 1H), 2.27 (qd, *J* = 7.2, 1.5 Hz, 2H), 2.14 (s, 1H), 2.14 (s, 2H), 1.51 – 1.45 (m, 2H), 1.45 – 1.39 (m, 2H).

^{13}C NMR (126 MHz, CD_3CN, 23°C, δ): 156.2, 136.6, 135.5, 134.0, 131.3, 130.8, 121.1, 111.1, 84.9, 69.9, 32.9, 28.3, 27.1, 18.3.

^{19}F NMR (471 MHz, CD_3CN, 23°C, δ): –151.40 – –151.93 (m).

HRMS-ESI (m/z) calc'd for $C_{20}H_{19}S_2$ [M-BF_4]$^+$, 323.0923; found, 323.0921; deviation: 0.6 ppm.

3,4-dihydro-2*H*-pyran derived thianthrenium salt TT-5

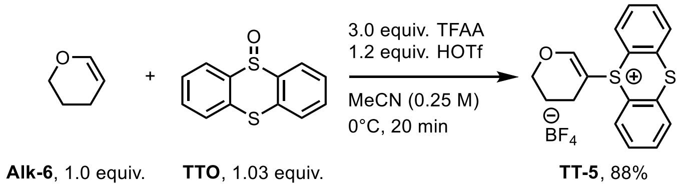

Prepared according to a reported procedure and spectra are in good accordance with literature[4].

Under ambient atmosphere, a 20 mL borosilicate vial equipped with a magnetic stir bar was charged with 3,4-dihydro-2*H*-pyrane (45.7 µL, 42.1 mg, 0.50 mmol, 1.0 equiv.), thianthrene *S*-oxide (**TTO**) (120 mg, 0.517 mmol, 1.03 equiv.), and MeCN (2.0 mL, c = 0.25 M). After cooling to 0°C, trifluoroacetic anhydride (0.21 mL, 0.31 g, 1.5 mmol, 3.0 equiv.) was added dropwise within 30 seconds, followed by dropwise addition of HOTf (52 µL, 88 mg, 0.59 mmol, 1.2 equiv.) within 10 seconds. After stirring the lilac mixture at 0°C for 20 min, the resulting yellow mixture was concentrated under reduced pressure and subsequently diluted with DCM (10 mL). The DCM solution was poured onto a saturated aqueous $NaHCO_3$ solution (ca. 20 mL). The combined mixture was poured into a separatory funnel, and the layers were separated. The DCM layer was collected, and the aqueous layer was further extracted with DCM (2 × ca. 10 mL). The combined DCM phase was washed with aqueous $NaBF_4$ solution (2 × ca. 20 mL, 5% w/w). The DCM layer was dried over Na_2SO_4 (10 g), filtered, and the solvent was removed under reduced pressure. The residue was purified by chromatography on silica gel, eluting with DCM/MeOH (97:3, v/v). The product-containing fractions were collected and concentrated under reduced pressure. The residue was further dried in vacuo to afford **TT-5** (170 mg, 88%) as a colorless solid.

R*f* = 0.46 (DCM/MeOH = 15:1, v/v)

NMR Spectroscopy:

^{1}H NMR (500 MHz, CD_2Cl_2, 23°C, δ): 8.07 (dd, *J* = 7.9, 1.2 Hz, 2H), 7.79 – 7.73 (m, 5H), 7.69 (ddd, *J* = 8.6, 6.6, 2.0 Hz, 2H), 4.18 – 4.14 (t, *J* = 5.9 Hz, 2H), 2.21 (t, *J* = 6.3 Hz, 2H), 1.95 (p, *J* = 6.1 Hz, 2H).

^{13}C NMR (126 MHz, CD_2Cl_2, 23°C, δ): 159.6, 136.2, 134.6, 133.0, 130.6, 130.0, 118.6, 103.0, 68.3, 21.8, 20.2.

^{19}F NMR (471 MHz, CD_2Cl_2, 23°C, δ): –151.78 (bs), –151.83 (bs).

HRMS-ESI (m/z) calc'd for $C_{22}H_{25}OS_2^+$ $[M-BF_4]^+$, 369.1341; found, 369.1343; deviation: –0.5 ppm.

2-Vinylbicyclo[2.2.1]heptane derived thianthrenium salt TT-6

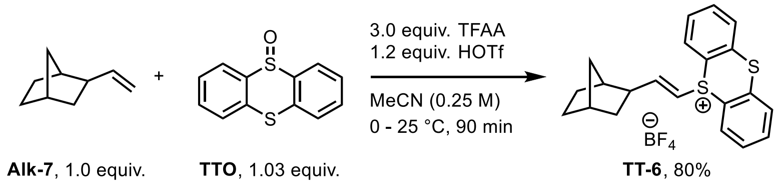

Prepared according to a reported procedure and spectra are in good accordance with literature[4].

Under ambient atmosphere, a 20 mL borosilicate vial equipped with a magnetic stir bar was charged with 2-vinylbicyclo[2.2.1]heptane (60.1 mg, 0.50 mmol, 1.0 equiv.), thianthrene *S*-oxide (**TTO**) (120 mg, 0.517 mmol, 1.03 equiv.), and MeCN (2.0 mL, *c* = 0.25 M). After cooling to 0°C, trifluoroacetic anhydride (0.21 mL, 0.31 g, 1.5 mmol, 3.0 equiv.) was added dropwise within 30 seconds, followed by dropwise addition of HOTf (52 µL, 88 mg, 0.59 mmol, 1.2 equiv.) within 10 seconds. After stirring the lilac mixture at 0°C for 60 min followed by stirring at 25°C for 30 min, the resulting purple mixture was concentrated under reduced pressure and subsequently diluted with DCM (10 mL). The DCM solution was poured onto a saturated aqueous $NaHCO_3$ solution (ca. 20 mL). The combined mixture was poured into a separatory funnel, and the layers were separated. The DCM layer was collected, and the aqueous layer was further extracted with DCM (2 × ca. 10 mL). The combined DCM phase was washed with aqueous $NaBF_4$ solution (2 × ca. 20 mL, 5% w/w). The DCM layer was dried over Na_2SO_4 (10 g), filtered, and the solvent was removed under reduced pressure. The residue was purified by chromatography on silica gel, eluting with DCM/MeOH (98:2, v/v). The product-containing fractions were collected and concentrated under reduced pressure. The residue was further dried in vacuo to afford **TT-6** (*E*:*Z* = 13:1, 170 mg, 80%) as a colorless solid.

R*f* = 0.46 (DCM/MeOH = 15:1, v/v)

NMR Spectroscopy:

^{1}**H NMR** (600 MHz, CD_2Cl_2, 23°C, δ): 8.23 (dtd, *J* = 8.0, 1.4, 0.5 Hz, 2H), 7.88 (dtd, *J* = 7.9, 1.3, 0.5 Hz, 2H), 7.80 – 7.74 (m, 2H), 7.74 – 7.66 (m, 2H), 6.98 (dd, *J* = 14.8, 8.6 Hz, 1H), 6.49 (dd, *J* = 14.7, 1.1 Hz, 1H), 2.40 – 2.22 (m, 2H), 2.18 – 2.09 (m, 1H), 1.53 – 1.42 (m, 3H), 1.34 – 1.26 (m, 2H), 1.21 – 1.09 (m, 3H).

^{13}**C NMR** (151 MHz, CD_2Cl_2, 23°C, δ): 161.0, 136.2 (d, *J* = 3.2 Hz), 134.8, 133.6 (d, *J* = 4.1 Hz), 130.7, 130.6, 121.0 (d, *J* = 4.8 Hz), 107.6, 46.5, 42.2, 37.1, 36.8, 36.1, 29.7, 28.9.

^{19}**F NMR** (565 MHz, CD_2Cl_2, 23°C, δ): –151.13 (bs), –151.18 (bs).

HRMS-ESI (m/z) calc'd for $C_{21}H_{21}S_2^+$ [M-BF_4]$^+$, 337.1079; found, 337.1078; deviation: 0.3 ppm.

(+)-3-Carene derived thianthrenium salt TT-7

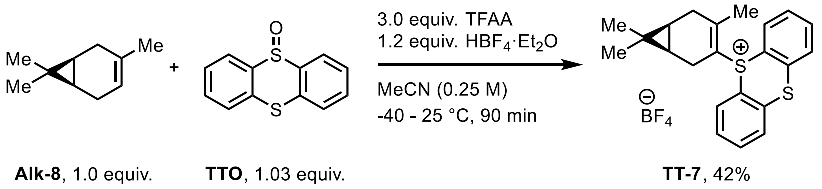

Under ambient atmosphere, a 20 mL borosilicate vial equipped with a magnetic stir bar was charged with (+)-3-carene (79 µL, 68 mg, 0.50 mmol, 1.0 equiv.), thianthrene *S*-oxide (**TTO**) (120 mg, 0.517 mmol, 1.03 equiv.), and MeCN (2.0 mL, *c* = 0.25 M). After cooling to –40°C, trifluoroacetic anhydride (0.21 mL, 0.31 g, 1.5 mmol, 3.0 equiv.) was added dropwise within 30 seconds, followed by dropwise addition of $HBF_4{\cdot}OEt_2$ (87 µL, 97 mg, 0.59 mmol, 1.2 equiv.) within 10 seconds. After stirring the mixture at –40°C for 60 min followed by stirring at 25°C for 30 min, the resulting mixture was diluted with DCM (10 mL). The DCM solution was

poured onto a saturated aqueous $NaHCO_3$ solution (ca. 20 mL). The combined mixture was poured into a separatory funnel, and the layers were separated. The DCM layer was collected, and the aqueous layer was further extracted with DCM (2 × ca. 10 mL). The combined DCM phase was washed with aqueous $NaBF_4$ solution (2 × ca. 20 mL, 5% w/w). The DCM layer was dried over Na_2SO_4 (10 g), filtered, and the solvent was removed under reduced pressure. The residue was purified by chromatography on silica gel, eluting with EtOAc/pentane (1:3, v/v) → EtOAc (100%) → DCM/MeOH (98:2, v/v). The product-containing fractions were concentrated under reduced pressure and the residue was further dried in vacuo to afford **TT-7** (92 mg, 42%) as a colorless solid.

R*f* = 0.44 (DCM/MeOH = 19:1, v/v)

NMR Spectroscopy:

^{1}H NMR (500 MHz, $CDCl_3$, 23°C, δ): 8.02 (dd, *J* = 8.0, 1.4 Hz, 1H), 7.97 (dd, *J* = 8.1, 1.3 Hz, 1H), 7.73 – 7.69 (m, 2H), 7.68 – 7.56 (m, 4H), 2.74 – 2.63 (m, 1H), 2.48 (s, 3H), 2.46 – 2.39 (m, 1H), 2.26 (ddd, *J* = 20.8, 4.1, 2.1 Hz, 1H), 2.10 – 2.03 (m, 1H), 0.89 (s, 3H), 0.75 – 0.68 (m, 2H), 0.42 (s, 3H).

^{13}C NMR (126 MHz, $CDCl_3$, 23°C, δ): 155.7, 135.7, 135.4, 134.5, 134.4, 133.7, 133.3, 130.2, 131.0, 129.2, 129.1, 124.0, 115.7, 115.6, 31.2, 27.5, 22.3, 21.2, 18.0, 17.8, 17.2, 13.1.

^{19}F NMR (471 MHz, $CDCl_3$, 23°C, δ): –152.38, –152.43.

HRMS-ESI (m/z) calc'd for $C_{22}H_{23}S_2^+$ $[M-BF_4]^+$, 351.1236; found, 351.1234; deviation: 0.4 ppm.

(*Z*)-4-Octene derived thianthrenium salt TT-8

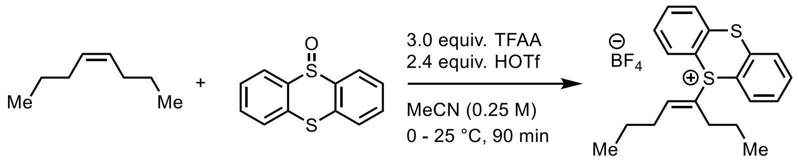

Alk-9, 1.0 equiv. **TTO**, 1.03 equiv. **TT-8**, 98%

Prepared according to a reported procedure and spectra are in good accordance with literature[4].

Under ambient atmosphere, a 20 mL borosilicate vial equipped with a magnetic stir bar was charged with (*Z*)-4-octene (78 µL, 56 mg, 0.50 mmol, 1.0 equiv.), thianthrene *S*-oxide (**TTO**) (120 mg, 0.517 mmol, 1.03 equiv.), and MeCN (2.0 mL, *c* = 0.25 M). After cooling to 0°C, trifluoroacetic anhydride (0.21 mL, 0.31 g, 1.5 mmol, 3.0 equiv.) was added dropwise within 30 seconds, followed by dropwise addition of HOTf (104 µL, 1.77 g, 1.18 mmol, 2.40 equiv.) within 10 seconds. After stirring the lilac mixture at 0°C for 60 min followed by stirring at 25°C for 30 min, the resulting purple mixture was concentrated under reduced pressure and subsequently diluted with DCM (10 mL). The DCM solution was poured onto a saturated aqueous $NaHCO_3$ solution (ca. 20 mL). The combined mixture was poured into a separatory funnel, and the layers were separated. The DCM layer was collected, and the aqueous layer was further extracted with DCM (2 × ca. 10 mL). The combined DCM phase was washed with aqueous $NaBF_4$ solution (2 × ca. 20 mL, 5% w/w). The DCM layer was dried over Na_2SO_4 (10 g), filtered, and the solvent was removed under reduced pressure. The

residue was purified by chromatography on silica gel, eluting with DCM/MeOH (98:2, v/v). The product-containing fractions were collected and concentrated under reduced pressure. The residue was further dried in vacuo to afford **TT-8** (*E:Z* > 49:1, 203 mg, 98%) as a colorless solid.

R*f* = 0.46 (DCM/MeOH = 15:1, v/v)

NMR Spectroscopy:

^{1}H NMR (500 MHz, CD_2Cl_2, 23°C, δ): 8.26 (dd, *J* = 7.9, 1.4 Hz, 2H), 7.94 (dd, *J* = 7.9, 1.5 Hz, 2H), 7.88 (td, *J* = 7.6, 1.4 Hz, 2H), 7.80 (td, *J* = 7.6, 1.5 Hz, 2H), 5.50 (t, *J* = 7.7 Hz, 1H), 2.18 (dt, *J* = 14.8, 7.3 Hz, 4H), 1.36 (hept, *J* = 7.4 Hz, 4H), 0.86 (dt, *J* = 17.1, 7.4 Hz, 6H).

^{13}C NMR (126 MHz, CD_2Cl_2, 23°C, δ): 145.0, 137.0, 135.2, 135.2, 130.6, 130.5, 124.5, 117.5, 30.9, 30.5, 29.8, 25.9, 25.3.

^{19}F NMR (471 MHz, CD_2Cl_2, 23°C, δ): –152.11 (bs), –152.16 (bs).

HRMS-ESI (m/z) calc'd for $C_{20}H_{23}S_2^+$ $[M\text{-}BF_4]^+$, 327.1236; found, 327.1235; deviation: 0.2 ppm.

Cyclopentene derived thianthrenium salt TT-9

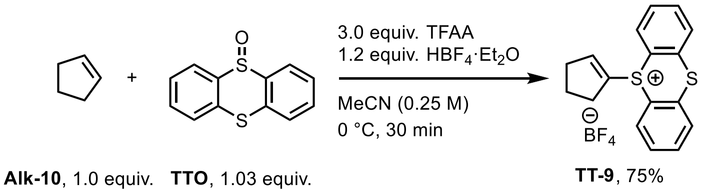

Prepared according to a reported procedure and spectra are in good accordance with literature[4].

Under ambient atmosphere, a 20 mL borosilicate vial equipped with a magnetic stir bar was charged cyclopentene (46 µL, 34 mg, 0.50 mmol, 1.0 equiv.), thianthrene *S*-oxide (**TTO**) (120 mg, 0.517 mmol, 1.03 equiv.), and MeCN (2.0 mL, *c* = 0.25 M). After cooling to 0°C, trifluoroacetic anhydride (0.21 mL, 0.31 g, 1.5 mmol, 3.0 equiv.) was added dropwise within 30 seconds, followed by dropwise addition of $HBF_4·OEt_2$ (87 µL, 97 mg, 0.59 mmol, 1.2 equiv.) within 10 seconds. After stirring the lilac mixture at 0°C for 30 min, the resulting light purple mixture was concentrated under reduced pressure and subsequently diluted with DCM (10 mL). The DCM solution was poured onto a saturated aqueous $NaHCO_3$ solution (ca. 20 mL). The combined mixture was poured into a separatory funnel, and the layers were separated. The DCM layer was collected, and the aqueous layer was further extracted with DCM (2 × ca. 10 mL). The combined DCM phase was washed with aqueous $NaBF_4$ solution (2 × ca. 20 mL, 5% w/w). The DCM layer was dried over Na_2SO_4 (10 g), filtered, and the solvent was removed under reduced pressure. The residue was purified by chromatography on silica gel, eluting with DCM/MeOH (98:2, v/v). The product-containing fractions were collected and concentrated under reduced pressure. The residue was further dried in vacuo to afford **TT-9** (139 mg, 75%) as a colorless solid.

R*f* = 0.46 (DCM/MeOH = 15:1, v/v)

NMR Spectroscopy:

^{1}H NMR** (500 MHz, CD_2Cl_2, 23°C, δ): 8.23 (dd, *J* = 7.9, 1.4 Hz, 2H), 7.87 (dd, *J* = 7.9, 1.5 Hz, 2H), 7.81 (td, *J* = 7.6, 1.4 Hz, 2H), 7.73 (td, *J* = 7.6, 1.4 Hz, 2H), 6.28 (p, *J* = 2.2 Hz, 1H), 2.58 – 2.48 (m, 2H), 2.39 – 2.33 (m, 2H), 2.05 (p, *J* = 7.7 Hz, 2H).

^{13}C NMR (151 MHz, CD_2Cl_2, 23°C, δ): 148.5, 136.8, 135.2, 134.4, 130.5, 130.4, 124.6, 117.8, 33.9, 32.9, 23.8.

^{19}F NMR (471 MHz, CD_2Cl_2, 23°C, δ): –149.08 – –154.48 (m).

HRMS-ESI (m/z) calc'd for $C_{17}H_{15}S_2^+$ [M-BF_4]$^+$, 283.0610; found, 283.0608; deviation: 0.5 ppm.

Cinchophen derived thianthrenium salt TT-10

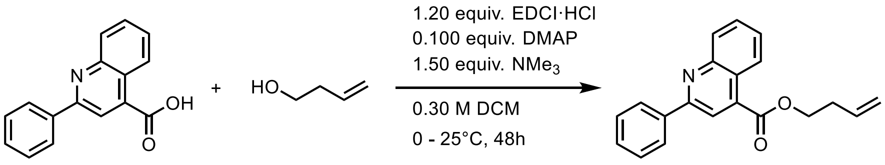

R-2, 1.00 equiv. **Alk-11**, 1.0 equiv. **Alk-12**, 66%

Prepared according to a reported procedure and spectra are in good accordance with literature[4].

Under ambient atmosphere, a 25 mL round-bottom flask equipped with a magnetic stir bar was charged with 3-buten-1-ol (1.0 mL, 0.87 g, 10 mmol, 1.0 equiv.), EDC·HCl (2.30 g, 12.0 mmol, 1.20 equiv.), trimethylamine (2.1 mL, 1.5 g, 15 mmol, 1.5 equiv.), and 4-dimethylamino-pyridine (DMAP, 122 mg, 1.0 mmol, 0.10 equiv.) in anhydrous DCM (33 mL, *c* = 0.30 M). After the reaction mixture was cooled to 0°C, cinchophen (2.94 g, 10.0 mmol, 1.0 equiv.) was added at 0°C in 5 portions within 2 min. The reaction mixture was stirred at 0°C for 30 min and then at 25°C for 48 h further. The resulting mixture was subsequently diluted with 20 mL DCM, washed by 1 M HCl (2 × ca. 20 mL), saturated aqueous $NaHCO_3$ (2 × ca. 20 mL), and brine (1 × ca. 20 mL). The organic layer was dried over Na_2SO_4, filtered, and the solvent was removed under reduced pressure. The residue was purified by chromatography on silica gel, eluting with EtOAc/hexanes (1:20, v/v). The product-containing fractions were collected and concentrated under reduced pressure. The residue was further dried in vacuo to afford the desired product **Alk-12** (2.0 g, 66%).

R*f* = 0.50 (EtOAc/pentane, 1:10, v/v)

NMR Spectroscopy:

^{1}H NMR (600 MHz, $CDCl_3$, 23°C, δ): 8.76 (m, 1H), 8.40 (s, 1H), 8.30 – 8.18 (m, 3H), 7.76 (ddd, *J* = 8.4, 6.8, 1.5 Hz, 1H), 7.61 (ddd, *J* = 8.2, 6.8, 1.2 Hz, 1H), 7.58 – 7.52 (m, 2H), 7.53 – 7.45 (m, 1H), 5.94 (ddt, *J* = 17.0, 10.2, 6.7 Hz, 1H), 5.26 (dq, *J* = 17.1, 1.6 Hz, 1H), 5.20 (dd, *J* = 10.2, 1.8 Hz, 1H), 4.54 (t, *J* = 6.7 Hz, 2H), 2.63 (qt, *J* = 6.7, 1.5 Hz, 2H).

^{13}C NMR (151 MHz, $CDCl_3$, 23°C, δ): 166.3, 156.6, 149.3, 138.8, 135.9, 133.9, 130.4, 129.9, 129.7, 128.9, 127.7, 127.5, 125.5, 124.0, 120.3, 117.8, 64.8, 33.2.

HRMS-EI GC (m/z) calc'd for $C_{20}H_{17}NO_2$ [M]$^+$, 303.1254; found, 303.1255; deviation: –0.4 ppm.

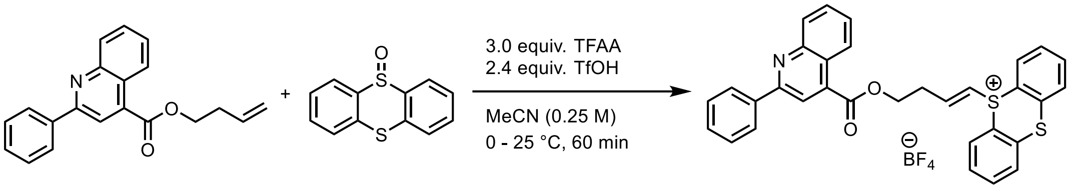

Alk-13, 1.0 equiv. **TTO**, 1.03 equiv. **TT-10**, 71%

Prepared according to a reported procedure and spectra are in good accordance with literature[4].

Under ambient atmosphere, a 20 mL borosilicate vial equipped with a magnetic stir bar was charged with **Alk-12** (152 mg, 0.50 mmol, 1.0 equiv.), thianthrene *S*-oxide (**TTO**) (120 mg, 0.517 mmol, 1.03 equiv.), and MeCN (2.0 mL, *c* = 0.25 M). After cooling to 0°C, HOTf (52 µL, 88 mg, 0.59 mmol, 1.2 equiv.) was added in one portion. Subsequently, trifluoroacetic anhydride (0.21 mL, 0.31 g, 1.5 mmol, 3.0 equiv.) was added dropwise within 30 seconds, followed by dropwise addition of HOTf (52 µL, 88 mg, 0.59 mmol, 1.2 equiv.) within 10 seconds. After stirring the lilac mixture at 0°C for 60 min followed by stirring at 25°C for 30 min, the resulting purple mixture was concentrated under reduced pressure and subsequently diluted with DCM (30 mL). The DCM solution was poured onto a saturated aqueous $NaHCO_3$ solution (ca. 60 mL). The combined mixture was poured into a separatory funnel, and the layers were separated. The DCM layer was collected, and the aqueous layer was further extracted with DCM (2 × ca. 30 mL). The combined DCM solution was washed with aqueous $NaBF_4$ solution (5 × ca. 60 mL, 5% w/w). The DCM layer was dried over Na_2SO_4, filtered, and the solvent was removed under reduced pressure. The residue was purified by chromatography on silica gel, eluting with DCM/MeOH (97:3, v/v). The product-containing fractions were collected and concentrated under reduced pressure. The residue was further dried in vacuo to afford **TT-10** (*E:Z* > 50:1, 215 mg, 71%) as a colorless solid.

$\mathbf{R}_f$ = 0.41 (DCM/MeOH, 15:1, v/v)

NMR Spectroscopy:

^{1}H NMR (600 MHz, CD_2Cl_2, 23°C, δ): 8.62 (ddd, *J* = 8.5, 1.5, 0.7 Hz, 1H), 8.29 – 8.21 (m, 4H), 8.16 – 8.10 (m, 2H), 7.82 (ddd, *J* = 8.4, 6.8, 1.4 Hz, 1H), 7.65 – 7.59 (m, 3H), 7.58 – 7.54 (m, 1H), 7.52 – 7.46 (m, 4H), 7.43 (ddd, *J* = 7.9, 6.9, 2.0 Hz, 2H), 7.16 (dt, *J* = 14.8, 7.1 Hz, 1H), 6.71 (dt, *J* = 14.8, 1.4 Hz, 1H), 4.59 (t, *J* = 6.0 Hz, 2H), 2.99 – 2.71 (m, 2H).

^{13}C NMR (151 MHz, CD_2Cl_2, 23°C, δ): 166.1, 156.8, 151.7, 149.6, 138.8, 135.9, 135.3, 134.8, 133.7, 130.6, 130.5, 130.4, 130.4, 130.3, 129.5, 128.4, 127.8, 125.6, 124.2, 120.2, 119.9, 112.6, 62.6, 33.0.

^{19}F NMR (471 MHz, CD_2Cl_2, 23°C, δ): –150.62 (bs), –150.67 (bs).

HRMS-ESI (m/z) calc'd for $C_{32}H_{24}NO_2S_2^+$ $[M-BF_4]^+$, 518.1243; found, 518.1248; deviation: –0.9 ppm.

Methyl cyclopent-3-ene-1-carboxylate derived thianthrenium salt (±)-TT-11

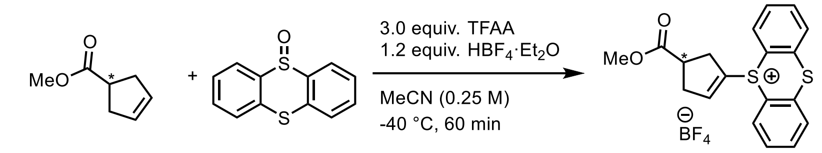

(±)-Alk-13, 1.0 equiv. **TTO**, 1.03 equiv. **(±)-TT-11**, 70%

Under ambient atmosphere, a 20 mL borosilicate vial equipped with a magnetic stir bar was charged with methyl cyclopent-3-ene-1-carboxylate (63.1 mg, 0.50 mmol, 1.0 equiv.), thianthrene *S*-oxide (**TTO**) (120 mg, 0.517 mmol, 1.03 equiv.), and MeCN (2.0 mL, *c* = 0.25 M). After cooling to –40°C, trifluoroacetic anhydride (0.21 mL, 0.31 g, 1.5 mmol, 3.0 equiv.) was added dropwise within 30 seconds, followed by dropwise addition of $HBF_4·OEt_2$ (87 µL, 97 mg, 0.59 mmol, 1.2 equiv.) within 10 seconds. After stirring the mixture at –40°C for 60 min followed by stirring at 25°C for 30 min, the resulting mixture was diluted with DCM (10 mL). The DCM solution was poured onto a saturated aqueous $NaHCO_3$ solution (ca. 20 mL). The combined mixture was poured into a separatory funnel, and the layers were separated. The DCM layer was collected, and the aqueous layer was further extracted with DCM (2 × ca. 10 mL). The combined DCM phase was washed with aqueous $NaBF_4$ solution (2 × ca. 20 mL, 5% w/w). The DCM layer was dried over Na_2SO_4 (10 g), filtered, and the solvent was removed under reduced pressure. The residue was purified by chromatography on silica gel, eluting with EtOAc/pentane (1:3, v/v) → EtOAc (100%) → DCM/MeOH (98:2, v/v). The product-containing fractions were concentrated under reduced pressure and the residue was further dried in vacuo to afford **(±)-TT-11** (150 mg, 70%) as a colorless solid.

R*f* = 0.42 (DCM/MeOH, 19:1, v/v)

NMR Spectroscopy:

^{1}H NMR (500 MHz, $CDCl_3$, 23°C, δ): 8.30 – 8.22 (m, 2H), 7.84 – 7.74 (m, 4H), 7.69 – 7.62 (m, 2H), 6.21 (p, *J* = 2.5 Hz, 1H), 3.7 (s, 3H), 3.33 – 3.22 (m, 1H), 2.87 – 2.71 (m, 2H), 2.71 – 2.52 (m, 2H).

^{13}C NMR (126 MHz, $CDCl_3$, 23°C, δ): 173.4, 145.7, 136.1, 136.1, 135.0, 134.9, 134.2, 134.2, 130.2, 130.1, 130.0, 123.0, 117.1, 116.7, 52.3, 41.6, 36.8, 35.2.

^{19}F NMR (471 MHz, $CDCl_3$, 23°C, δ): –151.11 (bs), –151.16 (bs).

HRMS-ESI (m/z) calc'd for $C_{19}H_{17}O_2S_2^+$ $[M-BF_4]^+$, 341.0664; found 341.0662; deviation: 0.8 ppm.

(1*Z*, 9*Z*)-Cyclohexadeca-1,9-diene derived thianthrenium salt TT-12

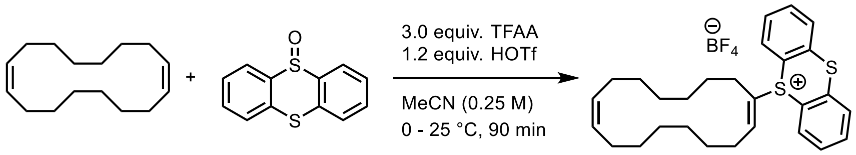

Alk-14, 1.0 equiv. **TTO**, 1.03 equiv. **TT-12**, 61%

Prepared according to a reported procedure and spectra are in good accordance with literature[4].

Under ambient atmosphere, a 20 mL borosilicate vial equipped with a magnetic stir bar was charged with (1*Z*, 9*Z*)-cyclohexadeca-1,9-diene (110 mg, 0.50 mmol, 1.0 equiv.), thianthrene *S*-oxide (**TTO**) (120 mg, 0.517 mmol, 1.03 equiv.), and MeCN (2.0 mL, *c* = 0.25 M). After cooling to 0°C, trifluoroacetic anhydride (0.21 mL, 0.31 g, 1.5 mmol, 3.0 equiv.) was added dropwise within 30 seconds, followed by dropwise addition of HOTf (52 µL, 88 mg, 0.59 mmol, 1.2 equiv.) within 10 seconds. After stirring the lilac mixture at 0°C for 60 min followed by stirring at 25°C for 30 min, the resulting purple mixture was concentrated under reduced pressure and subsequently diluted with DCM (10 mL). The DCM solution was poured onto a saturated aqueous $NaHCO_3$ solution (ca. 20 mL). The combined mixture was poured into a separatory funnel, and the layers were separated. The DCM layer was collected, and the aqueous layer was further extracted with DCM (2 × ca. 10 mL). The combined DCM phase was washed with aqueous $NaBF_4$ solution (2 × ca. 20 mL, 5% w/w). The DCM layer was dried over Na_2SO_4 (10 g), filtered, and the solvent was removed under reduced pressure. The residue was purified by chromatography on silica gel, eluting with DCM/MeOH (98:2, v/v). The product-containing fractions were collected and concentrated under reduced pressure. The residue was further dried in vacuo to afford **TT-12** (159 mg, 61%) as a colorless solid.

R_f = 0.46 (DCM/MeOH, 15:1, v/v)

NMR Spectroscopy:

^{1}H NMR (600 MHz, CD_2Cl_2, 23°C, δ): 8.21 (ddd, *J* = 7.9, 1.4, 0.5 Hz, 2H), 7.89 (ddd, *J* = 8.0, 1.4, 0.5 Hz, 2H), 7.83 (ddd, *J* = 8.0, 7.4, 1.4 Hz, 2H), 7.75 (ddd, *J* = 7.9, 7.4, 1.4 Hz, 2H), 5.52 (t, *J* = 8.4 Hz, 1H), 5.32 (d, *J* = 1.1 Hz, 2H), 2.25 – 2.18 (m, 2H), 2.15 – 2.08 (m, 2H), 2.04 – 1.97 (m, 3H), 1.36 – 1.08 (m, 16H).

^{13}C NMR (151 MHz, CD_2Cl_2, 23°C, δ): 142.4, 137.5, 135.4, 135.4, 135.3, 130.8, 130.6, 130.6, 130.4, 130.2, 123.6, 117.1, 29.4, 29.2, 29.0, 28.6, 28.5, 28.4, 28.2, 28.1, 28.1, 28.0, 27.8, 27.1, 26.9.

^{19}F NMR (565 MHz, CD_2Cl_2, 23°C, δ): –152.09 (bs), –152.14 (bs).

HRMS-ESI (m/z) calc'd for $C_{28}H_{35}S_2^+$ $[M-BF_4]^+$, 435.2175; found, 435.2175; deviation: –0.1 ppm.

(*E*)-4-Octene derived thianthrenium salt TT-13

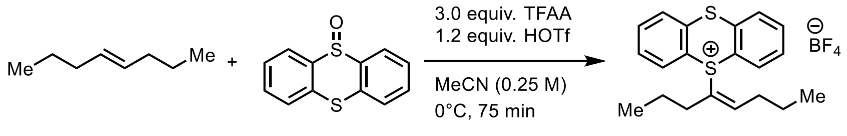

Alk-15, 1.0 equiv. **TTO**, 1.03 equiv. **TT-13**, 95%

Prepared according to a reported procedure and spectra are in good accordance with literature[4].

Under ambient atmosphere, a 20 mL borosilicate vial equipped with a magnetic stir bar was charged with (*E*)-4-octene (78 µL, 56 mg, 0.50 mmol, 1.0 equiv.), thianthrene *S*-oxide (**TTO**) (120 mg, 0.517 mmol, 1.03 equiv.), and MeCN (2.0 mL, *c* = 0.25 M). After cooling to 0°C, trifluoroacetic anhydride (0.21 mL, 0.31 g, 1.5 mmol, 3.0 equiv.) was added dropwise within 30 seconds, followed by dropwise addition of HOTf (52 µL, 88 mg, 0.59 mmol, 1.2 equiv.) within 10 seconds. After stirring the lilac mixture at 0°C for 75 min, the resulting

light pink mixture was concentrated under reduced pressure and subsequently diluted with DCM (10 mL). The DCM solution was poured onto a saturated aqueous $NaHCO_3$ solution (ca. 20 mL). The combined mixture was poured into a separatory funnel, and the layers were separated. The DCM layer was collected, and the aqueous layer was further extracted with DCM (2 × ca. 10 mL). The combined DCM phase was washed with aqueous $NaBF_4$ solution (2 × ca. 20 mL, 5% w/w). The DCM layer was dried over Na_2SO_4 (10 g), filtered, and the solvent was removed under reduced pressure. The residue was purified by chromatography on silica gel, eluting with DCM/MeOH (100:1, v/v). The product-containing fractions were collected and concentrated under reduced pressure. The residue was further dried in vacuo to afford **TT-13** (*E*:*Z* < 50:1, 197 mg, 95%) as a colorless solid.

R*f* = 0.46 (DCM/MeOH, 15:1, v/v)

NMR Spectroscopy:

^{1}H NMR (500 MHz, CD_2Cl_2, 23°C, δ): 8.05 (dd, *J* = 8.0, 1.4 Hz, 2H), 7.88 – 7.80 (m, 4H), 7.74 (ddd, *J* = 8.7, 7.2, 1.8 Hz, 2H), 6.46 (tt, *J* = 7.8, 1.5 Hz, 1H), 2.78 – 2.64 (m, 2H), 2.32 – 2.14 (m, 2H), 1.59 (h, *J* = 7.3 Hz, 2H), 1.39 (h, *J* = 7.4 Hz, 2H), 1.06 (t, *J* = 7.4 Hz, 3H), 0.80 (t, *J* = 7.3 Hz, 3H).

^{13}C NMR (126 MHz, CD_2Cl_2, 23°C, δ): 145.0, 137.0, 135.2, 135.2, 130.6, 130.4, 124.4, 117.5, 30.8, 30.5, 29.8, 25.9, 25.3.

^{19}F NMR (471 MHz, CD_2Cl_2, 23°C, δ): –152.29 (bs), –152.34 (bs).

HRMS-ESI (m/z) calc'd for $C_{20}H_{23}S_2^+$ [M-BF_4]$^+$, 327.1236; found, 327.1237; deviation: –0.5 ppm.

Tricyclo[6.2.1.0^{2,7}]undeca-4-ene derived thianthrenium salt TT-14

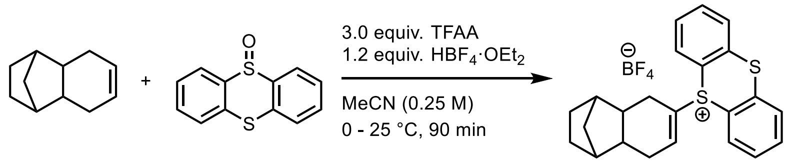

Alk-16, 1.0 equiv. **TTO**, 1.03 equiv. **TT-14**, 53%

Prepared according to a reported procedure and spectra are in good accordance with literature[4].

Under ambient atmosphere, a 20 mL borosilicate vial equipped with a magnetic stir bar was charged with tricyclo[6.2.1.0^{2,7}]undeca-4-ene (61 μL, 54 mg, 0.50 mmol, 1.0 equiv.), thianthrene *S*-oxide (**TTO**) (120 mg, 0.517 mmol, 1.03 equiv.), and MeCN (2.0 mL, *c* = 0.25 M). After cooling to 0°C, trifluoroacetic anhydride (0.21 mL, 0.31 g, 1.5 mmol, 3.0 equiv.) was added dropwise within 30 seconds, followed by dropwise addition of $HBF_4·OEt_2$ (87 μL, 88 mg, 0.59 mmol, 1.2 equiv.) within 10 seconds. After stirring the lilac mixture at 0°C for 45 min, the resulting yellow mixture was concentrated under reduced pressure and subsequently diluted with DCM (10 mL). The DCM solution was poured onto a saturated aqueous $NaHCO_3$ solution (ca. 20 mL). The combined mixture was poured into a separatory funnel, and the layers were separated. The DCM layer was collected, and the aqueous layer was further extracted with DCM (2 × ca. 10 mL). The combined DCM phase was washed with aqueous $NaBF_4$ solution (2 × ca. 20 mL, 5% w/w). The DCM layer was dried over

Na_2SO_4 (10 g), filtered, and the solvent was removed under reduced pressure. The residue was purified by chromatography on silica gel, eluting with DCM/MeOH (98:2, v/v). The product-containing fractions were collected and concentrated under reduced pressure. The residue was further dried in vacuo to afford **TT-14** (119 mg, 53%) as a colorless solid.

R*f* = 0.46 (DCM/MeOH, 15:1, v/v)

NMR Spectroscopy:

^{1}H NMR (500 MHz, CD_2Cl_2, 23°C, δ): 8.19 (dd, *J* = 8.0, 1.4 Hz, 2H), 7.87 (ddd, *J* = 8.0, 3.8, 1.4 Hz, 2H), 7.81 (tdd, *J* = 7.8, 3.1, 1.4 Hz, 2H), 7.73 (tdd, *J* = 7.5, 3.0, 1.4 Hz, 2H), 6.01 (dd, *J* = 7.6, 2.4 Hz, 1H), 2.49 – 2.42 (m, 1H), 2.25 – 2.17 (m, 1H), 1.95 – 1.90 (m, 1H), 1.89 – 1.83 (m, 1H), 1.66 – 1.54 (m, 4H), 1.51 – 1.40 (m, 3H), 1.18 – 1.06 (m, 2H), 1.05 – 0.98 (m, 1H).

^{13}C NMR (126 MHz, CD_2Cl_2, 23°C, δ): 142.9, 136.9, 135.4, 135.3, 135.2, 134.9, 130.8, 130.7, 130.5, 121.3, 117.5, 117.3, 44.4, 43.6, 43.3, 42.6, 33.6, 31.1, 29.7, 29.6, 29.5.

^{19}F NMR (471 MHz, CD_2Cl_2, 23°C, δ): –151.72 (bs), –151.78 (bs).

HRMS-ESI (m/z) calc'd for $C_{23}H_{23}S_2^+$ [M-BF_4]$^+$, 363.1236; found, 363.1235; deviation: 0.1 ppm

1-methylcyclopent-1-ene derived thianthrenium salt TT-15

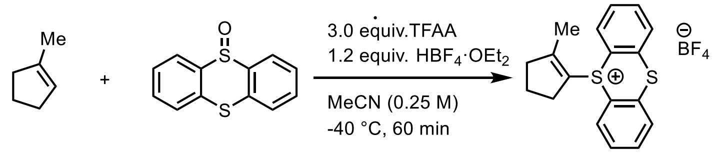

Alk-17, 1.0 equiv. **TTO**, 1.03 equiv. **TT-15**, 38%

Under ambient atmosphere, a 20 mL borosilicate vial equipped with a magnetic stir bar was charged with 1-methylcyclopent-1-ene (55 µL, 41 mg, 0.50 mmol, 1.0 equiv.), thianthrene *S*-oxide (**TTO**) (120 mg, 0.517 mmol, 1.03 equiv.), and MeCN (2.0 mL, *c* = 0.25 M). After cooling to –40°C, trifluoroacetic anhydride (0.21 mL, 0.31 g, 1.5 mmol, 3.0 equiv.) was added dropwise within 30 seconds, followed by dropwise addition of $HBF_4{\cdot}OEt_2$ (87 µL, 97 mg, 0.59 mmol, 1.2 equiv.) within 10 seconds. After stirring the mixture at –40°C for 60 min, the resulting mixture was diluted with DCM (10 mL). The DCM solution was poured onto a saturated aqueous $NaHCO_3$ solution (ca. 20 mL). The combined mixture was poured into a separatory funnel, and the layers were separated. The DCM layer was collected, and the aqueous layer was further extracted with DCM (2 × ca. 10 mL). The combined DCM phase was washed with aqueous $NaBF_4$ solution (2 × ca. 20 mL, 10% w/w). The DCM layer was dried over Na_2SO_4 (10 g), filtered, and the solvent was removed under reduced pressure. The residue was purified with chromatography on silica gel, eluting with EtOAc/pentane (1:3, v/v) → EtOAc (100%) → DCM/MeOH (98:2, v/v). The product-containing fractions were concentrated under reduced pressure and the residue was further dried in vacuo to afford **TT-15** (73 mg, 38%) as a colorless solid.

R*f* = 0.52 (DCM/MeOH, 19:1, v/v)

NMR Spectroscopy:

^{1}H NMR (500 MHz, $CDCl_3$, 23°C, δ): 8.19 (dd, *J* = 7.9, 1.3 Hz, 2H), 7.78 – 7.72 (m, 4H), 7.68 (m, 2H), 2.65 – 2.59 (m, 2H), 2.52 (m, 2H), 2.39 – 2.33 (m, 3H), 1.94 (p, *J* = 7.6 Hz, 2H).

^{13}C NMR (126 MHz, $CDCl_3$, 23°C, δ): 166.6, 135.7, 134.3, 133.4, 130.3, 129.5, 119.8, 118.3, 39.7, 33.7, 21.5, 16.3.

^{19}F NMR (471 MHz, $CDCl_3$, 23°C, δ): –152.07 (bs), –152.12 (bs).

HRMS-ESI (m/z) calc'd for $C_{18}H_{17}S_2^+$ $[M-BF_4]^+$, 297.0766; found, 297.0764; deviation: 0.7 ppm.

(–)-*cis*-Rose-oxide derived thianthrenium salt TT-16

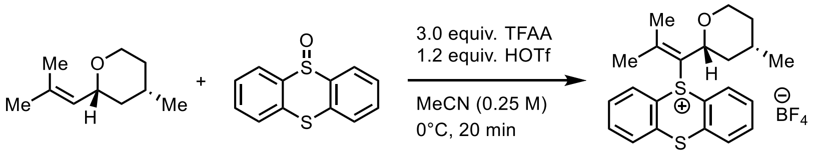

Alk-18, 1.0 equiv. **TTO**, 1.03 equiv. **TT-16**, 72%

Under ambient atmosphere, a 20 mL borosilicate vial equipped with a magnetic stir bar was charged with (–)-*cis*-rose-oxide (89 µL, 77 mg, 0.50 mmol, 1.0 equiv.), thianthrene *S*-oxide (**TTO**) (120 mg, 0.517 mmol, 1.03 equiv.), and MeCN (2.0 mL, *c* = 0.25 M). After cooling to 0°C, trifluoroacetic anhydride (0.21 mL, 0.31 g, 1.5 mmol, 3.0 equiv.) was added dropwise within 30 seconds, followed by dropwise addition of HOTf (52 µL, 88 mg, 0.59 mmol, 1.2 equiv.) within 10 seconds. After stirring the lilac mixture at 0°C for 20 min, the resulting yellow mixture was concentrated under reduced pressure and subsequently diluted with DCM (10 mL). The DCM solution was poured onto a saturated aqueous $NaHCO_3$ solution (ca. 20 mL). The combined mixture was poured into a separatory funnel, and the layers were separated. The DCM layer was collected, and the aqueous layer was further extracted with DCM (2 × ca. 10 mL). The combined DCM phase was washed with aqueous $NaBF_4$ solution (2 × ca. 20 mL, 5% w/w). The DCM layer was dried over Na_2SO_4 (10 g), filtered, and the solvent was removed under reduced pressure. The residue was purified by chromatography on silica gel, eluting with DCM/MeOH (97:3, v/v). The product-containing fractions were collected and concentrated under reduced pressure. The residue was further dried in vacuo to afford **TT-16** (164 mg, 72%) as a colorless solid.

R_f = 0.46 (DCM/MeOH, 15:1, v/v)

NMR Spectroscopy:

^{1}H NMR (500 MHz, $CDCl_3$, 23°C, δ): 7.92 (dd, *J* = 8.2, 1.2 Hz, 1H), 7.86 – 7.77 (m, 2H), 7.77 – 7.70 (m, 2H), 7.70 – 7.64 (m, 3H), 4.84 (dd, *J* = 11.6, 2.5 Hz, 1H), 3.98 (ddd, *J* = 11.6, 4.6, 1.5 Hz, 1H), 3.55 (td, *J* = 12.1, 2.3 Hz, 1H), 2.43 (s, 3H), 2.10 – 2.02 (m, 1H), 1.76 (s, 3H), 1.56 – 1.52 (m, 1H), 1.12 (tdd, *J* = 16.7, 14.1, 9.5 Hz, 1H), 1.03 – 0.95 (m, 1H), 0.92 (d, *J* = 6.5 Hz, 3H).

^{13}C NMR (126 MHz, $CDCl_3$, 23°C, δ): 164.4, 133.3, 133.2, 131.4, 131.3, 130.4, 129.7, 129.7, 128.9, 128.8, 124.0, 123.7, 119.2, 75.2, 68.6, 38.9, 33.6, 29.8, 26.7, 24.6, 22.0

^{19}F NMR (471 MHz, $CDCl_3$, 23°C, δ): –152.90 (bs), –152.96 (bs).

HRMS-ESI (m/z) calc'd for $C_{22}H_{25}S_2^+$ [M-BF4]+, 369.1341; found, 369.1340; deviation: 0.2 ppm.

(*S*)-(+)-Carvone derived thianthrenium salt TT-17

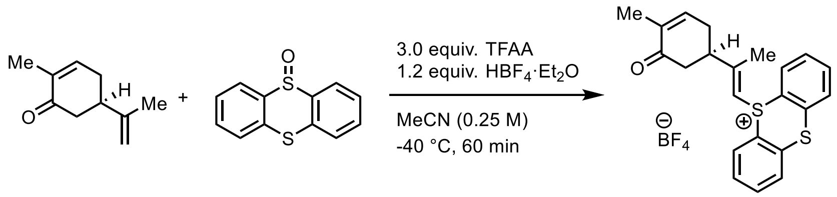

Alk-19, 1.0 equiv. **TTO**, 1.03 equiv. **TT-17**, 41%

Under ambient atmosphere, a 20 mL borosilicate vial equipped with a magnetic stir bar was charged with (*S*)-(+)-carvone (78 µL, 75 mg, 0.50 mmol, 1.0 equiv.), thianthrene *S*-oxide (**TTO**) (120 mg, 0.517 mmol, 1.03 equiv.), and MeCN (2.0 mL, *c* = 0.25 M). After cooling to –40°C, trifluoroacetic anhydride (0.21 mL, 0.31 g, 1.5 mmol, 3.0 equiv.) was added dropwise within 30 seconds, followed by dropwise addition of $HBF_4{\cdot}OEt_2$ (87 µL, 97 mg, 0.59 mmol, 1.2 equiv.) within 10 seconds. After stirring the mixture at –40°C for 60 min, the resulting mixture was diluted with DCM (10 mL). The DCM solution was poured onto a saturated aqueous $NaHCO_3$ solution (ca. 20 mL). The combined mixture was poured into a separatory funnel, and the layers were separated. The DCM layer was collected, and the aqueous layer was further extracted with DCM (2 × ca. 10 mL). The combined DCM phase was washed with aqueous $NaBF_4$ solution (2 × ca. 20 mL, 10% w/w). The DCM layer was dried over Na_2SO_4 (10 g), filtered, and the solvent was removed under reduced pressure. The residue was purified by chromatography on silica gel, eluting with EtOAc/pentane (1:3, v/v) → EtOAc (100%) → DCM/MeOH (97:3, v/v). The product-containing fractions were concentrated under reduced pressure and the residue was further dried in vacuo to afford **TT-17** (*E:Z* > 20:1, 93 mg, 41%) as a colorless solid.

$\mathbf{R}_f$ = 0.41 (DCM/MeOH, 19:1, v/v)

NMR Spectroscopy:

^{1}H NMR (500 MHz, $CDCl_3$, 23°C, δ): 8.38 (d, *J* = 7.0 Hz, 2H), 7.84 (dd, *J* = 7.8, 1.4 Hz, 2H), 7.72 (td, *J* = 7.6, 1.5 Hz, 2H), 7.67 (td, *J* = 7.6, 1.4 Hz, 2H), 6.68 – 6.66 (m, 1H), 6.43 (m, 1H), 2.97 – 2.87 (m, 1H), 2.47 – 2.43 (m, 2H), 2.42 (s, 3H), 2.37 – 2.31 (m, 2H), 1.71 (s, 3H).

^{13}C NMR (126 MHz, $CDCl_3$, 23°C, δ): 197.0, 166.3, 143.2, 135.9, 135.6, 134.3, 133.8, 130.5, 130.4, 121.7, 107.0, 44.6, 41.6, 30.0, 18.3, 15.7.

^{19}F NMR (471 MHz, $CDCl_3$, 23°C, δ): –151.01 (bs), –151.06 (bs).

HRMS-ESI (m/z) calc'd for $C_{22}H_{21}OS_2^+$ [M-BF4]+, 365.1028; found, 365.1023; deviation: 1.5 ppm.

(*S*)-(–)-*β*-Citronellol derived thianthrenium salt TT-18

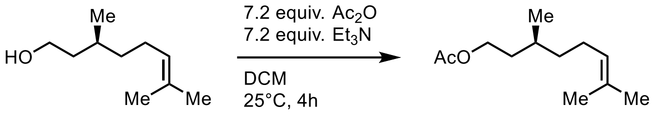

Alk-20, 1.0 equiv. **Alk-21,** 85%

Prepared according to a reported procedure and spectra are in good accordance with literature[5].

To a solution of (*S*)-(–)-*β*-citronellol (1.8 mL, 10 mmol, 1.0 equiv.) in anhydrous DCM (30 mL) was added Et_3N (10 mL, 72 mmol, 7.2 equiv.) and Ac_2O (6.8 mL, 72 mmol, 7.2 equiv.). The reaction solution was stirred at 25°C for 4 hours, and the mixture was quenched by pouring the reaction solution into ice water. The organic layer was washed with 1M solution of HCl (2 × ca. 60 mL), distilled H_2O (ca. 60 mL), saturated aqueous $NaHCO_3$ (ca. 60 mL) and brine (ca. 60 mL). The organic layer was dried over anhydrous Na_2SO_4 and the solvent was removed under reduced pressure. The residue was purified by chromatography on silica gel, eluting with DCM (100%) to afford **Alk-21** (1.7 g, 85%) as a colorless oil.

R_f = 0.50 (Et_2O/pentane, 19:1, v/v)

NMR Spectroscopy:

> **^{1}H NMR** (500 MHz, $CDCl_3$, 23°C, δ): 5.11 – 5.03 (m, 1H), 4.16 – 4.02 (m, 2H), 2.02 (s, 3H), 2.01 – 1.86 (m, 2H), 1.70 – 1.61 (m, 4H), 1.58 (s, 1H), 1.55 – 1.48 (m, 1H), 1.42 (dtd, *J* = 13.6, 7.5, 6.0 Hz, 1H), 1.38 – 1.27 (m, 1H), 1.21 – 1.12 (m, 1H), 0.89 (dd, *J* = 6.6, 1.4 Hz, 3H).
>
> **^{13}C NMR** (101 MHz, $CDCl_3$, 23°C, δ): 171.4, 131.5, 124.7, 63.2, 37.1, 35.6, 29.6, 25.9, 25.5, 21.2, 19.6, 17.8.

HRMS-ESI (m/z) calc'd for $C_{12}H_{26}NO_2$ $[M+NH_4]^+$, 216.1958, found 216.1961; deviation: –1.4 ppm.

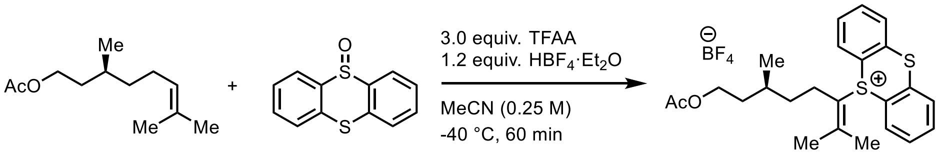

Alk-21, 1.0 equiv. **TTO**, 1.03 equiv. **TT-18**, 54%

Under ambient atmosphere, a 20 mL borosilicate vial equipped with a magnetic stir bar was charged with **Alk-21** (99.2 mg, 0.50 mmol, 1.0 equiv.), thianthrene *S*-oxide (**TTO**) (120 mg, 0.517 mmol, 1.03 equiv.), and MeCN (2.0 mL, *c* = 0.25 M). After cooling to –40°C, trifluoroacetic anhydride (0.21 mL, 0.31 g, 1.5 mmol, 3.0 equiv.) was added dropwise within 30 seconds, followed by dropwise addition of $HBF_4 \cdot OEt_2$ (87 μL, 97 mg, 0.59 mmol, 1.2 equiv.) within 10 seconds. After stirring the mixture at –40°C for 60 min, the resulting mixture was diluted with DCM (10 mL). The DCM solution was poured onto a saturated aqueous $NaHCO_3$ solution (ca. 20 mL). The combined mixture was poured into a separatory funnel, and the layers were separated. The DCM layer was collected, and the aqueous layer was further extracted with DCM (2 × ca. 10 mL). The

combined DCM phase was washed with aqueous $NaBF_4$ solution (2 × ca. 20 mL, 10% w/w). The DCM layer was dried over Na_2SO_4 (10 g), filtered, and the solvent was removed under reduced pressure. The residue was purified by chromatography on silica gel, eluting with EtOAc/pentane (1:3, v/v) → EtOAc (100%) → DCM/MeOH (98:2, v/v). The product-containing fractions were concentrated under reduced pressure and the residue was further dried in vacuo to afford **TT-18** (135 mg, 54%) as a colorless solid.

R*f* = 0.48 (DCM/MeOH, 19:1, v/v)

NMR Spectroscopy:

^{1}H NMR (500 MHz, $CDCl_3$, 23°C, δ): 8.18 – 8.08 (m, 2H), 7.75 – 7.69 (m, 2H), 7.68 – 7.62 (m, 4H), 3.92 – 3.80 (m, 2H), 2.59 (s, 3H), 2.49 – 2.39 (m, 1H), 2.37 – 2.27 (m, 1H), 2.01 (s, 3H), 1.99 (s, 3H), 1.38 – 1.16 (m, 3H), 0.93 (tt, *J* = 13.3, 4.8 Hz, 1H), 0.80 – 0.69 (m, 1H), 0.66 (d, *J* = 6.1 Hz, 3H).

^{13}C NMR (126 MHz, $CDCl_3$, 23°C, δ): 171.2, 157.8, 135.2, 135.1, 134.5, 134.4, 130.4, 129.2, 126.4, 117.5, 117.3, 62.4, 36.5, 35.0, 30.2, 29.5, 24.5, 23.5, 21.1, 18.8.

^{19}F NMR (471 MHz, $CDCl_3$, 23°C, δ): –152.36 (bs), –152.41 (bs).

HRMS-ESI (m/z) calc'd for $C_{24}H_{29}O_2S_2^+$ $[M-BF_4]^+$, 413.1604; found, 413.1607; deviation: –0.8 ppm.

(*S*)-(–)-Perillaldehyde derived thianthrenium salt TT-19

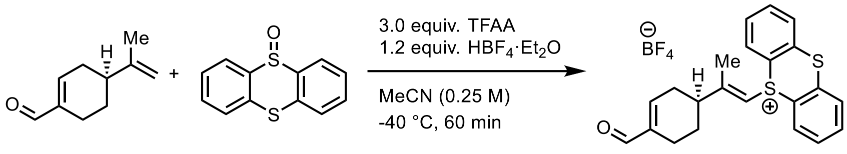

Alk-22, 1.0 equiv. **TTO**, 1.03 equiv. **TT-19**, 40%

Under ambient atmosphere, a 20 mL borosilicate vial equipped with a magnetic stir bar was charged with (*S*)-(–)-perillaldehyde (77 μL, 75 mg, 0.50 mmol, 1.0 equiv.), thianthrene *S*-oxide (**TTO**) (120 mg, 0.517 mmol, 1.03 equiv.), and MeCN (2.0 mL, *c* = 0.25 M). After cooling to –40°C, trifluoroacetic anhydride (0.21 mL, 0.31 g, 1.5 mmol, 3.0 equiv.) was added dropwise within 30 seconds, followed by dropwise addition of $HBF_4 \cdot OEt_2$ (87 μL, 97 mg, 0.59 mmol, 1.2 equiv.) within 10 seconds. After stirring the mixture at –40°C for 60 min, the resulting mixture was diluted with DCM (10 mL). The DCM solution was poured onto a saturated aqueous $NaHCO_3$ solution (ca. 20 mL). The combined mixture was poured into a separatory funnel, and the layers were separated. The DCM layer was collected, and the aqueous layer was further extracted with DCM (2 × ca. 10 mL). The combined DCM phase was washed with aqueous $NaBF_4$ solution (2 × ca. 20 mL, 10% w/w). The DCM layer was dried over Na_2SO_4 (10 g), filtered, and the solvent was removed under reduced pressure. The residue was purified by chromatography on silica gel, eluting with EtOAc/pentane (1:3, v/v) → EtOAc (100%) → DCM/MeOH (97:3, v/v). The product-containing fractions were concentrated under reduced pressure and the residue was further dried in vacuo to afford **TT-19** (*E:Z* = 10:1, 90 mg, 40%) as a colorless solid.

R*f* = 0.41 (DCM/MeOH, 19:1, v/v)

NMR Spectroscopy:

^{1}H NMR (500 MHz, $CDCl_3$, 23°C, δ): 9.37 (s, 1H), 8.33 (ddd, *J* = 10.6, 7.8, 1.4 Hz, 2H), 7.83 (td, *J* = 7.4, 1.4 Hz, 2H), 7.76 – 7.70 (m, 2H), 7.68 – 7.64 (m, 2H), 6.74 (dt, *J* = 5.4, 2.0 Hz, 1H), 6.45 – 6.37 (m, 1H), 2.56 – 2.44 (m, 2H), 2.41 (s, 3H), 2.34 – 2.20 (m, 2H), 2.07 – 2.01 (m, 1H), 1.85 – 1.80 (m, 1H), 1.47 (dtd, *J* = 12.9, 10.9, 5.3 Hz, 1H).

^{13}C NMR (126 MHz, $CDCl_3$, 23°C, δ): 193.4, 169.0, 148.2, 140.9, 135.6, 135.5, 134.4, 134.3, 133.5, 133.3, 130.5, 130.3, 130.4, 122.0, 121.6, 105.9, 43.5, 30.6, 25.6, 21.0, 18.3.

^{19}F NMR (565 MHz, $CDCl_3$, 23°C, δ): –151.11(bs), –151.17 (bs).

HRMS-ESI (m/z) calc'd for $C_{22}H_{21}OS_2^+$ [M-BF_4]$^+$, 365.1028; found, 365.1031; deviation: –0.8 ppm.

Citral derived thianthrenium salt TT-20

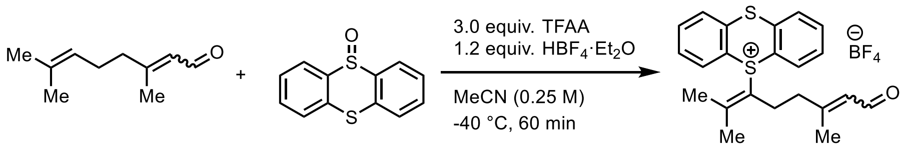

Alk-23, 1.0 equiv. **TTO**, 1.03 equiv. **TT-20**, 56%

Under ambient atmosphere, a 20 mL borosilicate vial equipped with a magnetic stir bar was charged with citral (76.1 mg, 0.50 mmol, 1.0 equiv.), thianthrene *S*-oxide (**TTO**) (120 mg, 0.517 mmol, 1.03 equiv.), and MeCN (2.0 mL, *c* = 0.25 M). After cooling to –40°C, trifluoroacetic anhydride (0.21 mL, 0.31 g, 1.5 mmol, 3.0 equiv.) was added dropwise within 30 seconds, followed by dropwise addition of $HBF_4·OEt_2$ (87 µL, 97 mg, 0.59 mmol, 1.2 equiv.) within 10 seconds. After stirring the mixture at –40°C for 60 min, the resulting mixture was diluted with DCM (10 mL). The DCM solution was poured onto a saturated aqueous $NaHCO_3$ solution (ca. 20 mL). The combined mixture was poured into a separatory funnel, and the layers were separated. The DCM layer was collected, and the aqueous layer was further extracted with DCM (2 × ca. 10 mL). The combined DCM phase was washed with aqueous $NaBF_4$ solution (2 × ca. 20 mL, 5% w/w). The DCM layer was dried over Na_2SO_4 (10 g), filtered, and the solvent was removed under reduced pressure. The residue was purified by chromatography on silica gel, eluting with EtOAc/pentane (1:3, v/v) → EtOAc (100%) → DCM/MeOH (98:2, v/v). The product-containing fractions were concentrated under reduced pressure and the residue was further dried in vacuo to afford **TT-20** (127 mg, 56%) as a colorless solid.

R*f* = 0.41 (DCM/MeOH, 19:1, v/v)

NMR Spectroscopy:

^{1}H NMR (500 MHz, CD_3CN, 23°C, δ): 9.84 (d, *J* = 7.9 Hz, 1H), 9.39 (d, *J* = 7.7 Hz, 1H), 8.02 – 7.96 (m, 4H), 7.81 – 7.77 (m, 8H), 7.69 – 7.64 (m, 4H), 5.71 – 5.67 (m, 1H), 5.48 – 5.44 (m, 1H), 2.67 – 2.58 (m, 4H), 2.53 (s, 3H), 2.52 (s, 3H), 2.27 – 2.20 (m, 2H), 2.06 (s, 3H), 2.01 (s, 3H), 1.92 (d, *J* = 1.3 Hz, 3H), 1.81 – 1.75 (m, 2H), 1.71 (d, *J* = 1.4 Hz, 3H).

^{13}C NMR (126 MHz, $CDCl_3$, 23°C, δ): 191.3, 190.5, 163.8, 163.6, 145.6, 145.5, 134.8, 129.3, 129.2,

129.0, 128.7, 128.1, 127.4, 127.0, 126.2, 126.2, 39.7, 34.6, 34.5, 34.2, 34.1, 31.8, 31.7, 30.5, 25.4, 20.6, 20.5, 20.4, 20.3, 17.9.

^{19}F NMR (471 MHz, CD_3CN, 23°C, δ): –151.60 (bs), –151.65 (bs).

HRMS-ESI (m/z) calc'd for $C_{22}H_{23}OS_2^+$ [M-BF_4]$^+$, 367.1185; found, 367.1186; deviation: –0.2 ppm.

Cyclohexene derived thianthrenium salt TT-21

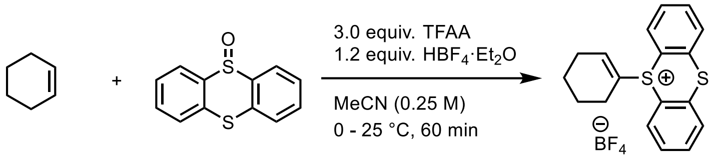

Alk-24, 1.0 equiv. **TTO**, 1.03 equiv. **TT-21**, 72%

Prepared according to a reported procedure and spectra are in good accordance with literature[4].

Under ambient atmosphere, a 20 mL borosilicate vial equipped with a magnetic stir bar was charged cyclohexene (50.6 µL, 41.1 mg, 0.50 mmol, 1.0 equiv.), thianthrene *S*-oxide (**TTO**) (120 mg, 0.517 mmol, 1.03 equiv.), and MeCN (2.0 mL, *c* = 0.25 M). After cooling to 0°C, trifluoroacetic anhydride (0.21 mL, 0.31 g, 1.5 mmol, 3.0 equiv.) was added dropwise within 30 seconds, followed by dropwise addition of $HBF_4·OEt_2$ (87 µL, 97 mg, 0.59 mmol, 1.2 equiv.) within 10 seconds. After stirring the lilac mixture at 0°C for 45 min followed by stirring at 25°C for 15 min, the resulting purple mixture was concentrated under reduced pressure and subsequently diluted with DCM (10 mL). The DCM solution was poured onto a saturated aqueous $NaHCO_3$ solution (ca. 20 mL). The combined mixture was poured into a separatory funnel, and the layers were separated. The DCM layer was collected, and the aqueous layer was further extracted with DCM (2 × ca. 10 mL). The combined DCM phase was washed with aqueous $NaBF_4$ solution (2 × ca. 20 mL, 5% w/w). The DCM layer was dried over Na_2SO_4 (10 g), filtered, and the solvent was removed under reduced pressure. The residue was purified by chromatography on silica gel, eluting with DCM/MeOH (98:2, v/v). The product-containing fractions were collected and concentrated under reduced pressure. The residue was further dried in vacuo to afford **TT-21** (138 mg, 72%) as a colorless solid.

R_f = 0.46 (DCM/MeOH, 15:1, v/v)

NMR Spectroscopy:

^{1}H NMR (500 MHz, CD_2Cl_2, 23°C, δ): 8.19 (d, *J* = 7.9 Hz, 2H), 7.88 (d, *J* = 7.9 Hz, 2H), 7.82 (t, *J* = 7.7 Hz, 2H), 7.75 – 7.70 (m, 2H), 6.05 (d, *J* = 4.1 Hz, 1H), 2.23 – 2.17 (m, 2H), 1.99 – 1.93 (m, 2H), 1.73 – 1.66 (m, 2H), 1.59 – 1.52 (m, 2H).

^{13}C NMR (126 MHz, CD_2Cl_2, 23°C, δ): 142.0, 137.1, 135.4, 135.2, 130.7, 130.5, 123.8, 117.1, 27.7, 25.9, 23.0, 20.8.

^{19}F NMR (471 MHz, CD_2Cl_2, 23°C, δ): –149.84 (bs), –149.92 (bs).

HRMS-ESI (m/z) calc'd for $C_{18}H_{17}S_2^+$ [M-BF_4]$^+$, 297.0766; found, 297.0765; deviation: 0.5 ppm.

Synthesis of alkyl redox-active esters

3-(3,5-Dimethoxyphenyl)propanoic acid derived redox-active ester RAE-1

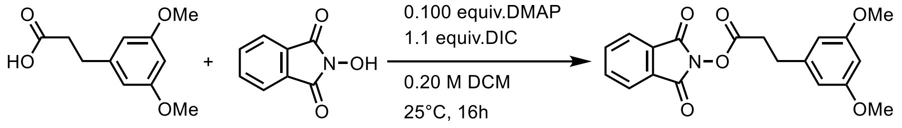

R-3, 1.00 equiv. **NHPI**, 1.00 equiv. **RAE-1**, 58%

Prepared according to a reported procedure and spectra are in good accordance with literature[6].

To a 50 mL round bottom flask containing a teflon-coated magnetic stirring bar were added 3-(3,5-dimethoxyphenyl)-propanoic acid (1.26 g, 6.0 mmol, 1.0 equiv.), 4-dimethylamino-pyridine (DMAP, 73.3 mg, 0.60 mmol, 0.10 equiv.), *N*,*N*'-diisopropylcarbodiimide (DIC, 1.0 mL, 0.83 g, 6.6 mmol, 1.1 equiv.), *N*-hydroxyphthalimide (**NHPI**, 979 mg, 6.0 mmol, 1.0 equiv.) and DCM (30 mL, *c* = 0.20 M). After addition, the mixture was stirred for 16 h at 25°C. After stirring, the reaction mixture was filtered over SiO_2 (10 g), and the SiO_2 was subsequently washed with DCM (10 mL). The solvent was removed under reduced pressure, and the resulting residue was purified by column chromatography on silica gel, eluting with EtOAc/hexanes (1:3, v/v) to afford **RAE-1** (1.2 g, 58%) as a colorless solid.

NMR Spectroscopy:

^{1}H NMR (500 MHz, $CDCl_3$, 23°C, δ): 7.90 (dd, *J* = 5.5, 3.1 Hz, 2H), 7.80 (dd, *J* = 5.5, 3.2 Hz, 2H), 6.41 (d, *J* = 2.3 Hz, 2H), 6.35 (t, *J* = 2.3 Hz, 1H), 3.80 (s, 6H), 3.11 – 3.02 (m, 2H), 3.01 – 2.93 (m, 2H).

^{13}C NMR (126 MHz, $CDCl_3$, 23°C, δ): 169.0, 162.0, 161.2, 141.7, 134.9, 129.1, 124.1, 106.4, 99.0, 55.5, 32.8, 31.0.

HRMS-ESI (m/z) calc'd for $C_{19}H_{17}NO_6Na$ $[M+Na]^+$, 378.0948; found, 378.0948; deviation: 0.0 ppm.

4-((Tert-butoxycarbonyl)amino)butanoic acid derived redox-active ester RAE-2

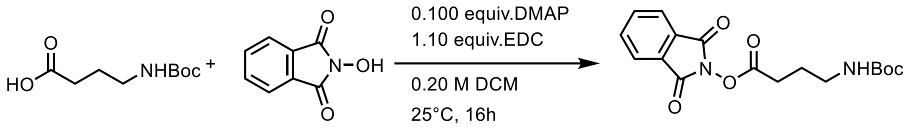

R-4, 1.00 equiv. **NHPI**, 1.00 equiv. **RAE-2**, 73%

Prepared according to a reported procedure and spectra are in good accordance with literature[7].

A round-bottom flask was charged with N-hydroxyphthalimide (**NHPI**, 979 mg, 6.0 mmol, 1.0 equiv.), 4-((tert-butoxycarbonyl)amino)butanoic acid (1.22 g, 6.0 mmol, 1.0 equiv.), and 4-dimethylamino-pyridine (DMAP, 73.3 mg, 0.60 mmol 0.10 equiv.). Dichloromethane was added (30 mL, *c* = 0.20 M) and the mixture was stirred vigorously. Finally, *N*-ethyl-*N*'-(3-dimethylaminopropyl)-carbodiimide hydrochloride (EDC·HCl, 1.27 g, 6.60 mmol, 1.10 equiv.) was added, and the mixture was allowed to stir for 16 h at 25°C. After stirring, the mixture was diluted with 10 mL DCM and poured onto a 1M solution of HCl (ca. 60 mL). The combined

mixture was poured into a separatory funnel, and the layers were separated. The DCM layer was collected and further extracted with 1M solution of HCl (2 × ca. 60 mL). Subsequently, the DCM layer was dried over Na_2SO_4 (10 g), filtered, and the solvent was removed under reduced pressure. The resulting residue was purified by column chromatography on silica gel, eluting with EtOAc/hexanes (1:3, v/v) to afford **RAE-2** (1.5 g, 73%) as a colorless solid.

NMR Spectroscopy:

^{1}**H NMR** (500 MHz, $CDCl_3$, 23°C, δ): 7.89 (dd, *J* = 5.4, 3.1 Hz, 2H), 7.79 (dd, *J* = 5.5, 3.1 Hz, 2H), 4.75 (s, 1H), 3.27 (q, *J* = 6.5 Hz, 2H), 2.72 (t, *J* = 7.4 Hz, 2H), 1.99 (p, *J* = 7.1 Hz, 2H), 1.44 (s, 9H).

^{13}**C NMR** (126 MHz, $CDCl_3$, 23°C, δ): 169.4, 162.0, 156.1, 134.9, 129.0, 124.1, 79.6, 39.5, 28.5, 25.3.

HRMS-ESI (m/z) calc'd for $C_{17}H_{20}N_2O_6Na$ $[M+Na]^+$, 371.1214; found, 371.1210; deviation: 0.9 ppm.

Tetrahydropyran-4-carboxylic acid derived redox-active ester RAE-3

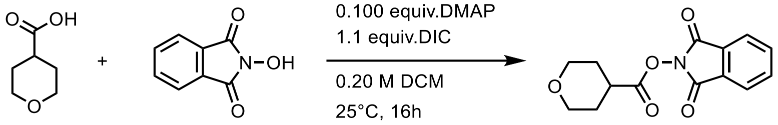

R-5, 1.00 equiv. **NHPI**, 1.00 equiv. **RAE-3**, 57%

Prepared according to a reported procedure and spectra are in good accordance with literature[7].

To a 50 mL round bottom flask containing a teflon-coated magnetic stirring bar were added Tetrahydropyran-4-carboxylic acid (781 mg, 6.0 mmol, 1.0 equiv.), 4-dimethylamino-pyridine (DMAP, 73.3 mg, 0.60 mmol, 0.10 equiv.), *N*,*N*'-diisopropylcarbodiimide (DIC, 1.0 mL, 0.83 g, 6.6 mmol, 1.1 equiv.), *N*-hydroxyphthalimide (**NHPI**, 979 mg, 6.0 mmol, 1.0 equiv.) and DCM (30 mL, *c* = 0.20 M). After addition, the mixture was stirred for 16 h at 25°C. After stirring, the reaction mixture was filtered over SiO_2 (10 g), and the SiO_2 was subsequently washed with DCM (10 mL). The solvent was removed under reduced pressure, and the resulting residue was purified by column chromatography on silica gel, eluting with EtOAc/hexanes (1:3, v/v) to afford **RAE-3** (0.94 g, 57%) as a colorless solid.

NMR Spectroscopy:

^{1}**H NMR** (500 MHz, $CDCl_3$, 23°C, δ): 7.89 (dd, *J* = 5.5, 3.1 Hz, 2H), 7.80 (dd, *J* = 5.5, 3.1 Hz, 2H), 4.02 (dt, *J* = 11.9, 3.9 Hz, 2H), 3.71 – 3.39 (m, 2H), 3.13 – 2.88 (m, 1H), 2.16 – 1.86 (m, 4H).

^{13}**C NMR** (126 MHz, $CDCl_3$, 23°C, δ): 170.7, 162.1, 134.9, 129.1, 124.1, 66.7, 37.8, 28.4.

HRMS-ESI (m/z) calc'd for $C_{14}H_{13}NO_5Na$ $[M+Na]^+$, 298.0686; found, 298.0687; deviation: –0.5 ppm.

4,4-Difluorocyclohexane-1-carboxylic acid derived redox-active ester RAE-4

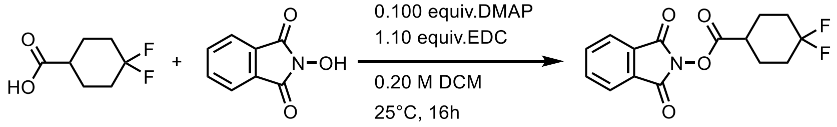

R-6, 1.00 equiv. **NHPI**, 1.00 equiv. **RAE-4**, 92%

Prepared according to a reported procedure and spectra are in good accordance with literature[8].

A round-bottom flask was charged with N-hydroxyphthalimide (**NHPI**, 979 mg, 6.0 mmol, 1.0 equiv.), 4,4-difluorocyclohexane-1-carboxylic acid (985 mg, 6.0 mmol, 1.0 equiv.), and 4-dimethylamino-pyridine (DMAP, 73.3 mg, 0.60 mmol, 0.10 equiv.). Dichloromethane was added (30 mL, *c* = 0.20 M) and the mixture was stirred vigorously. Finally, *N*-ethyl-*N*'-(3-dimethylaminopropyl)-carbodiimide hydrochloride (EDC·HCl, 1.27 g, 6.60 mmol, 1.10 equiv.) was added, and the mixture was allowed to stir for 16 h at 25°C. After stirring, the mixture was diluted with 10 mL DCM and poured onto a 1M solution of HCl (ca. 60 mL). The combined mixture was poured into a separatory funnel, and the layers were separated. The DCM layer was collected and further extracted with 1M solution of HCl (2 × ca. 60 mL). Subsequently, the DCM layer was dried over Na_2SO_4 (10 g), filtered, and the solvent was removed under reduced pressure. The resulting residue was purified by column chromatography on silica gel, eluting with EtOAc/hexanes (1:3, v/v) to afford **RAE-4** (1.7g, 92%) as a colorless solid.

NMR Spectroscopy:

^{1}H NMR (500 MHz, $CDCl_3$, 23°C, δ): 7.89 (dd, *J* = 5.4, 3.1 Hz, 2H), 7.80 (dd, *J* = 5.5, 3.1 Hz, 2H), 2.94 – 2.77 (m, 1H), 2.27 – 2.13 (m, 4H), 2.12 – 1.99 (m, 2H), 1.98 – 1.79 (m, 2H).

^{13}C NMR (126 MHz, $CDCl_3$, 23°C, δ): 170.5, 162.0, 135.0, 129.0, 124.1, 122.3, 37.9, 32.1 (t, *J* = 24.80 Hz), 25.1 (t, *J* = 5.25 Hz).

^{19}F NMR (565 MHz, $CDCl_3$, 23°C, δ): –95.65 (d, *J* = 233.22 Hz), –98.45 (d, *J* = 218.05 Hz).

HRMS-CI GC (m/z) calc'd for $C_{15}H_{17}N_2O_4F_2$ $[M+NH_4]^+$, 327.1151; found, 327.1154; deviation: –1.1 ppm.

1-Benzoylpiperidine-4-carboxylic acid derived redox-active ester RAE-5

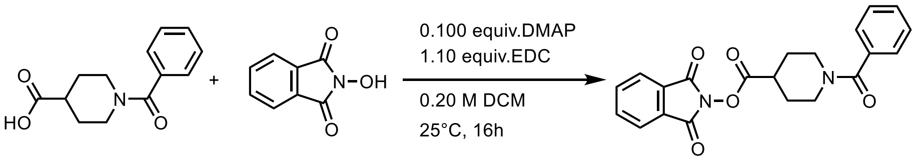

R-7, 1.00 equiv. **NHPI**, 1.00 equiv. **RAE-5**, 66%

Prepared according to a reported procedure and spectra are in good accordance with literature[9].

A round-bottom flask was charged with N-hydroxyphthalimide (**NHPI**, 979 mg, 6.0 mmol, 1.0 equiv.), 1-benzoylpiperidin-4-carboxylic acid (1.40 g, 6.0 mmol, 1.0 equiv.), and 4-dimethylamino-pyridine (DMAP, 73.3 mg, 0.60 mmol, 0.10 equiv.). Dichloromethane was added (30 mL, *c* = 0.20 M) and the mixture was stirred

vigorously. Finally, *N*-ethyl-*N*'-(3-dimethylaminopropyl)-carbodiimide hydrochloride (EDC·HCl, 1.27 g, 6.60 mmol, 1.10 equiv.) was added, and the mixture was allowed to stir for 16 h at 25°C. After stirring, the mixture was diluted with 10 mL DCM and poured onto a 1M solution of HCl (ca. 60 mL). The combined mixture was poured into a separatory funnel, and the layers were separated. The DCM layer was collected and further extracted with 1M solution of HCl (2 × ca. 60 mL). Subsequently, the DCM layer was dried over Na_2SO_4 (10 g), filtered, and the solvent was removed under reduced pressure. The resulting residue was purified by column chromatography on silica gel, eluting with EtOAc/hexanes (1:3, v/v) to afford **RAE-5** (1.5 g, 66%) as a colorless solid.

NMR Spectroscopy:

^{1}H NMR (500 MHz, $CDCl_3$, 23°C, δ): 7.85 (dd, *J* = 5.6, 3.0 Hz, 2H), 7.76 (dd, *J* = 5.4, 3.1 Hz, 2H), 7.43 – 7.33 (m, 5H), 4.45 (s, 1H), 3.77 (s, 1H), 3.21 (ddd, *J* = 13.7, 10.4, 3.3 Hz, 2H), 3.02 (tt, *J* = 10.0, 4.2 Hz, 1H), 2.02 (m, 4H).

^{13}C NMR (126 MHz, $CDCl_3$, 23°C, δ): 170.5, 170.4, 161.9, 135.7, 134.9, 129.8, 128.8, 128.6, 126.9, 124.0, 46.5, 40.9, 38.4, 28.4, 27.6.

HRMS-ESI (m/z) calc'd for $C_{21}H_{18}N_2O_5Na$ $[M+Na]^+$, 401.1108; found, 401.1111; deviation: –0.9 ppm.

N-tert-butyloxycarbonyl-azetidine-3-carboxylic acid derived redox-active ester RAE-6

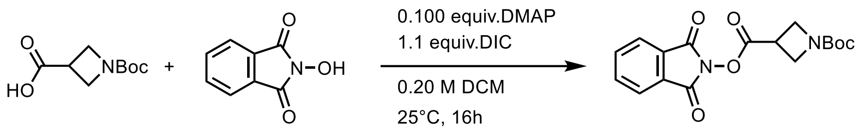

R-8, 1.00 equiv. **NHPI**, 1.00 equiv. **RAE-6**, 53%

Prepared according to a reported procedure and spectra are in good accordance with literature[10].

To a 50 mL round bottom flask containing a teflon-coated magnetic stirring bar were added *N-tert*-butyloxycarbonyl-azetidin-3-carboxylic acid (1.21 g, 6.0 mmol, 1.0 equiv.), 4-dimethylamino-pyridine (DMAP, 73.3 mg, 0.60 mmol, 0.10 equiv.), *N*,*N*'-diisopropylcarbodiimide (DIC, 1.0 mL, 0.83 g, 6.6 mmol, 1.1 equiv.), *N*-hydroxyphthalimide (**NHPI**, 979 mg, 6.0 mmol, 1.0 equiv.) and DCM (30 mL, *c* = 0.20 M). After addition, the mixture was stirred for 16 h at 25°C. After stirring, the reaction mixture was filtered over SiO_2 (10 g), and the SiO_2 was subsequently washed with DCM (10 mL). The solvent was removed under reduced pressure, and the resulting residue was purified by column chromatography on silica gel, eluting with EtOAc/hexanes (1:3, v/v) to afford **RAE-6** (1.1 g, 53%) as a colorless solid.

NMR Spectroscopy:

^{1}H NMR (600 MHz, $CDCl_3$, 23°C, δ): 7.90 (dd, *J* = 5.5, 3.0 Hz, 2H), 7.81 (dd, *J* = 5.5, 3.0 Hz, 2H), 4.32 – 4.23 (m, 4H), 3.72 (tt, *J* = 8.9, 6.2 Hz, 1H), 1.45 (s, 9H).

^{13}C NMR (126 MHz, $CDCl_3$, 23°C, δ): 169.2, 161.8, 155.9, 135.1, 129.0, 124.3, 80.4, 29.8, 28.5.

HRMS-ESI (m/z) calc'd for $C_{17}H_{18}N_2O_6Na$ $[M+Na]^+$, 369.1057; found, 369.1057; deviation: 0.0 ppm.

Levulinic acid derived redox-active ester RAE-7

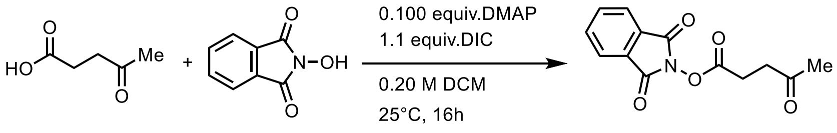

R-9, 1.00 equiv. **NHPI**, 1.00 equiv. **RAE-7**, 50%

Prepared according to a reported procedure and spectra are in good accordance with literature[8].

To a 50 mL round bottom flask containing a teflon-coated magnetic stirring bar were added levulinic acid (697 mg, 6.0 mmol, 1.0 equiv.), 4-dimethylamino-pyridine (DMAP, 73.3 mg, 0.60 mmol, 0.10 equiv.), *N*,*N*'-diisopropylcarbodiimide (DIC, 1.0 mL, 0.83 g, 6.6 mmol, 1.1 equiv.), *N*-hydroxyphthalimide (**NHPI**, 979 mg, 6.0 mmol, 1.0 equiv.) and DCM (30 mL, *c* = 0.20 M). After addition, the mixture was stirred for 16 h at 25°C. After stirring, the reaction mixture was filtered over SiO_2 (10 g), and the SiO_2 was subsequently washed with DCM (10 mL). The solvent was removed under reduced pressure, and the resulting residue was purified by column chromatography on silica gel, eluting with EtOAc/hexanes (1:3, v/v) to afford **RAE-7** (0.78 g, 50%) as a colorless solid.

NMR Spectroscopy:

^{1}H NMR (600 MHz, $CDCl_3$, 23°C, δ): 7.86 (dd, *J* = 5.5, 3.1 Hz, 2H), 7.77 (dd, *J* = 5.5, 3.0 Hz, 2H), 2.97 – 2.91 (m, 2H), 2.91 – 2.85 (m, 2H), 2.20 (s, 3H).

^{13}C NMR (151 MHz, $CDCl_3$, 23°C, δ): 205.0, 169.2, 161.8, 134.9, 128.9, 124.1, 37.7, 29.8, 25.2.

HRMS-ESI (m/z) calc'd for $C_{13}H_{11}NO_5Na$ $[M+Na]^+$, 284.0529; found, 284.0530; deviation: –0.2 ppm.

4-oxocyclohexane-1-carboxylic acid derived redox-active ester RAE-8

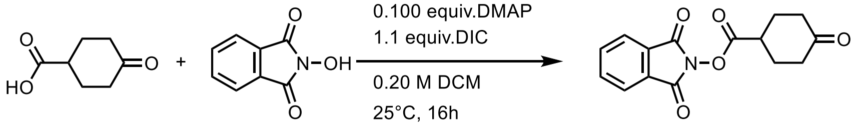

R-10, 1.00 equiv. **NHPI**, 1.00 equiv. **RAE-8**, 64%

Prepared according to a reported procedure and spectra are in good accordance with literature[10].

To a 50 mL round bottom flask containing a teflon-coated magnetic stirring bar were added 4-oxocyclohexane-1-carboxylic acid (852 mg, 6.0 mmol, 1.0 equiv.), 4-dimethylamino-pyridine (DMAP, 73.3 mg, 0.60 mmol, 0.10 equiv.), *N*,*N*'-diisopropylcarbodiimide (DIC, 1.0 mL, 0.83 g, 6.6 mmol, 1.1 equiv.), *N*-hydroxyphthalimide (**NHPI**, 979 mg, 6.0 mmol, 1.0 equiv.) and DCM (30 mL, *c* = 0.20 M). After addition, the mixture was stirred for 16 h at 25°C. After stirring, the reaction mixture was filtered over SiO_2 (10 g), and the SiO_2 was subsequently washed with DCM (10 mL). The solvent was removed under reduced pressure, and the resulting residue was purified by column chromatography on silica gel, eluting with EtOAc/hexanes (1:3, v/v) to afford **RAE-8** (1.1 g, 64%) as a colorless solid.

NMR Spectroscopy:

^{1}H NMR (500 MHz, $CDCl_3$, 23°C, δ): 7.90 (dd, J = 5.5, 3.1 Hz, 2H), 7.81 (dd, J = 5.5, 3.1 Hz, 2H), 3.20 (tt, J = 8.4, 4.2 Hz, 1H), 2.67 – 2.58 (m, 2H), 2.48 – 2.41 (m, 2H), 2.40 – 2.33 (m, 2H), 2.32 – 2.24 (m, 2H).

^{13}C NMR (126 MHz, $CDCl_3$, 23°C, δ): 209.0, 170.6, 162.0, 135.0, 129.0, 124.2, 39.3, 38.1, 28.4.

HRMS-ESI (m/z) calc'd for $C_{15}H_{13}NO_5Na$ $[M+Na]^+$, 310.0686; found, 310.0684; deviation: –0.3 ppm.

4-(tert-butoxy)-4-oxobutanoic acid derived redox-active ester RAE-9

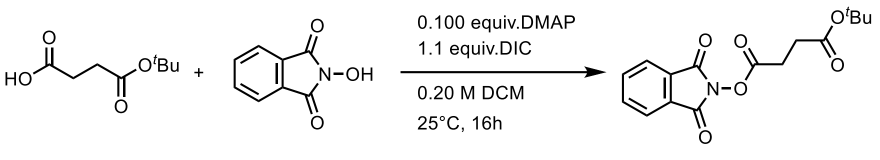

R-11, 1.00 equiv. **NHPI**, 1.00 equiv. **RAE-9**, 64%

Prepared according to a reported procedure and spectra are in good accordance with literature[11].

To a 50 mL round bottom flask containing a teflon-coated magnetic stirring bar were added 4-(tert-butoxy)-4-oxobutanoic acid (1.05 g, 6.0 mmol, 1.0 equiv.), 4-dimethylamino-pyridine (DMAP, 73.3 mg, 0.60 mmol, 0.10 equiv.), *N*,*N*'-diisopropylcarbodiimide (DIC, 1.0 mL, 0.83 g, 6.6 mmol, 1.1 equiv.), *N*-hydroxyphthalimide (**NHPI**, 979 mg, 6.0 mmol, 1.0 equiv.) and DCM (30 mL, c = 0.20 M). After addition, the mixture was stirred for 16 h at 25°C. After stirring, the reaction mixture was filtered over SiO_2 (10 g), and the SiO_2 was subsequently washed with DCM (10 mL). The solvent was removed under reduced pressure, and the resulting residue was purified by column chromatography on silica gel, eluting with EtOAc/hexanes (1:3, v/v) to afford **RAE-9** (1.2 g, 64%) as a colorless solid.

NMR Spectroscopy:

^{1}H NMR (500 MHz, $CDCl_3$, 23°C, δ): 7.89 (dd, J = 5.5, 3.1 Hz, 2H), 7.79 (dd, J = 5.5, 3.2 Hz, 2H), 2.97 (t, J = 7.0 Hz, 2H), 2.70 (t, J = 7.1 Hz, 2H), 1.47 (s, 9H).

^{13}C NMR (126 MHz, $CDCl_3$, 23°C, δ): 170.2, 168.9, 161.8, 134.9, 129.0, 124.1, 81.6, 30.1, 28.1, 26.6.

HRMS-ESI (m/z) calc'd for $C_{16}H_{17}NO_6Na$ $[M+Na]^+$, 342.0948; found, 342.0949; deviation: –0.3 ppm.

Indometacin derived redox-active ester RAE-10

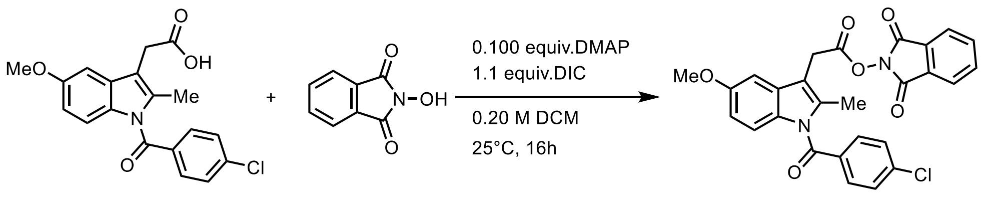

R-12, 1.00 equiv. **NHPI**, 1.00 equiv. **RAE-10**, 61%

Prepared according to a reported procedure and spectra are in good accordance with literature[12].

To a 50 mL round bottom flask containing a teflon-coated magnetic stirring bar were added Indomethacin

(2.15 g, 6.0 mmol, 1.0 equiv.), 4-dimethylamino-pyridine (DMAP, 73.3 mg, 0.60 mmol, 0.10 equiv.), *N*,*N'*-diisopropylcarbodiimide (DIC, 1.0 mL, 0.83 g, 6.6 mmol, 1.1 equiv.), *N*-hydroxyphthalimide (**NHPI**, 979 mg, 6.0 mmol, 1.0 equiv.) and DCM (30 mL, *c* = 0.20 M). After addition, the mixture was stirred for 16 h at 25°C. After stirring, the reaction mixture was filtered over SiO_2 (10 g), and the SiO_2 was subsequently washed with DCM (10 mL). The solvent was removed under reduced pressure, and the resulting residue was purified by column chromatography on silica gel, eluting with EtOAc/hexanes (1:3, v/v) to afford **RAE-10** (1.8 g, 61%) as a colorless solid.

NMR Spectroscopy:

^{1}H NMR (600 MHz, $CDCl_3$, 23°C, δ): 7.88 (dd, *J* = 5.5, 3.0 Hz, 2H), 7.83 – 7.75 (m, 2H), 7.72 – 7.64 (m, 2H), 7.53 – 7.44 (m, 2H), 7.06 – 6.99 (m, 1H), 6.93 (dd, *J* = 9.0, 0.5 Hz, 1H), 6.71 (dd, *J* = 9.0, 2.5 Hz, 1H), 4.05 – 4.00 (m, 2H), 3.89 (s, 3H), 2.42 (s, 3H).

^{13}C NMR (126 MHz, $CDCl_3$, 23°C, δ): 168.4, 167.2, 161.9, 156.4, 139.6, 136.6, 135.0, 133.8, 131.5, 130.9, 130.1, 129.3, 129.0, 124.2, 115.2, 112.6, 110.3, 100.8, 55.9, 27.3, 13.6.

HRMS-ESI (m/z) calc'd for $C_{27}H_{19}N_2O_6ClNa$ $[M+Na]^+$, 525.0824; found, 525.0830; deviation: –1.2 ppm.

2-Methoxyacetic acid derived redox-active ester RAE-11

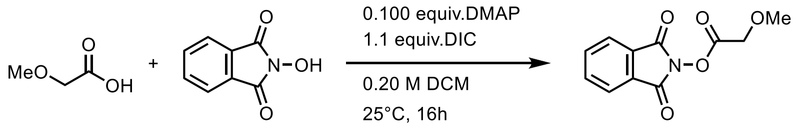

R-13, 1.00 equiv. **NHPI**, 1.00 equiv. **RAE-11**, 55%

Prepared according to a reported procedure and spectra are in good accordance with literature[13].

To a 50 mL round bottom flask containing a teflon-coated magnetic stirring bar were added 2-methoxy acetic acid (0.54 g, 0.46 mL, 6.0 mmol, 1.0 equiv.), 4-dimethylamino-pyridine (DMAP, 73.3 mg, 0.60 mmol, 0.10 equiv.), *N*,*N'*-diisopropylcarbodiimide (DIC, 1.0 mL, 0.83 g, 6.6 mmol, 1.1 equiv.), *N*-hydroxyphthalimide (**NHPI**, 979 mg, 6.0 mmol, 1.0 equiv.) and DCM (30 mL, *c* = 0.20 M). After addition, the mixture was stirred for 16 h at 25°C. After stirring, the reaction mixture was filtered over SiO_2 (10 g), and the SiO_2 was subsequently washed with DCM (10 mL). The solvent was removed under reduced pressure, and the resulting residue was purified by column chromatography on silica gel, eluting with EtOAc/hexanes (1:3, v/v) to afford **RAE-11** (0.78 g, 55%) as a colorless solid.

NMR Spectroscopy:

^{1}H NMR (500 MHz, $CDCl_3$, 23°C, δ): 7.91 (dd, *J* = 5.5, 3.1 Hz, 2H), 7.81 (dd, *J* = 5.5, 3.1 Hz, 2H), 4.45 (s, 2H), 3.55 (s, 3H).

^{13}C NMR (126 MHz, $CDCl_3$, 23°C, δ): 167.0, 162.0, 135.2, 129.2, 124.5, 68.0, 60.2.

HRMS-ESI (m/z) calc'd for $C_{11}H_9NO_5Na$ $[M+Na]^+$, 258.0373; found, 258.0374; deviation: –0.2 ppm.

2,4-D derived redox-active ester RAE-12

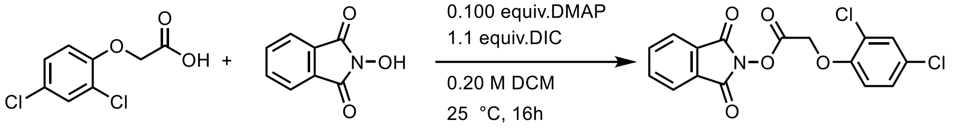

R-14, 1.00 equiv. **NHPI**, 1.00 equiv. **RAE-12**, 71%

Prepared according to a reported procedure and spectra are in good accordance with literature[14].

To a 50 mL round bottom flask containing a teflon-coated magnetic stirring bar were added 2,4-D (1.33 g, 6.0 mmol, 1.0 equiv.), 4-dimethylamino-pyridine (DMAP, 73.3 mg, 0.60 mmol, 0.10 equiv.), *N*,*N*'-diisopropylcarbodiimide (DIC, 1.0 mL, 0.83 g, 6.6 mmol, 1.1 equiv.), *N*-hydroxyphthalimide (**NHPI**, 979 mg, 6.0 mmol, 1.0 equiv.) and DCM (30 mL, *c* = 0.20 M). After addition, the mixture was stirred for 16 h at 25°C. After stirring, the reaction mixture was filtered over SiO_2 (10 g), and the SiO_2 was subsequently washed with DCM (10 mL). The solvent was removed under reduced pressure, and the resulting residue was purified by column chromatography on silica gel, eluting with EtOAc/hexanes (1:3, v/v) to afford **RAE-12** (1.6 g, 71%) as a colorless solid.

NMR Spectroscopy:

^{1}H NMR (500 MHz, $CDCl_3$, 23°C, δ): 7.91 (dd, *J* = 5.5, 3.1 Hz, 2H), 7.82 (dd, *J* = 5.5, 3.1 Hz, 2H), 7.41 (d, *J* = 2.5 Hz, 1H), 7.25 (dd, *J* = 8.8, 2.6 Hz, 1H), 6.96 (d, *J* = 8.8 Hz, 1H), 5.10 (s, 2H).

^{13}C NMR (126 MHz, $CDCl_3$, 23°C, δ): 165.1, 161.6, 152.0, 135.2, 130.7, 128.9, 128.3, 128.0, 124.8, 124.3, 115.7, 64.8.

HRMS-EI GC (m/z) calc'd for $C_{16}H_9NO_5Cl_2$ $[M]^+$, 364.9852; found, 364.9853; deviation: –0.2 ppm.

(−)-Menthyloxyacetic acid derived redox-active ester RAE-13

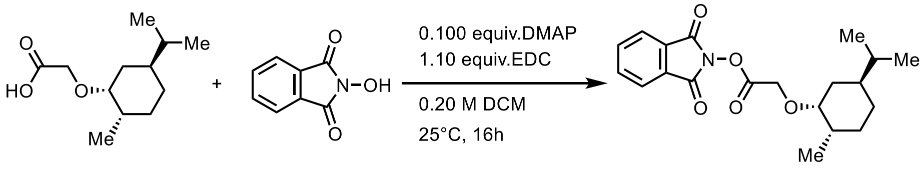

R-15, 1.00 equiv. **NHPI**, 1.00 equiv. **RAE-13**, 56%

Prepared according to a reported procedure and spectra are in good accordance with literature[15].

A round-bottom flask was charged with N-hydroxyphthalimide (**NHPI**, 979 mg, 6.0 mmol, 1.0 equiv.), (–)-menthyloxyacetic acid (1.29 g, 6.0 mmol, 1.0 equiv.), and 4-dimethylamino-pyridine (DMAP, 73.3 mg, 0.60 mmol, 0.10 equiv.). Dichloromethane was added (30 mL, *c* = 0.20 M) and the mixture was stirred vigorously. Finally, *N*-ethyl-*N*'-(3-dimethylaminopropyl)-carbodiimide hydrochloride (EDC·HCl, 1.27 g, 6.60 mmol, 1.10 equiv.) was added, and the mixture was allowed to stir for 16 h at 25°C. After stirring, the mixture was diluted with 10 mL DCM and poured onto a 1M solution of HCl (ca. 60 mL). The combined mixture was poured into a separatory funnel, and the layers were separated. The DCM layer was collected and further extracted with

1M solution of HCl (2 × ca. 60 mL). Subsequently, the DCM layer was dried over Na_2SO_4 (10 g), filtered, and the solvent was removed under reduced pressure. The resulting residue was purified by column chromatography on silica gel, eluting with EtOAc/hexanes (1:3, v/v) to afford **RAE-13** (1.2 g, 56%) as a colorless solid.

NMR Spectroscopy:

^{1}H NMR (500 MHz, $CDCl_3$, 23°C, δ): 7.88 (dd, *J* = 5.5, 3.0 Hz, 2H), 7.79 (dd, *J* = 5.5, 3.0 Hz, 2H), 4.52 (s, 2H), 3.28 (td, *J* = 10.7, 4.3 Hz, 1H), 2.29 (pd, *J* = 7.0, 2.6 Hz, 1H), 2.15 – 2.11 (m, 1H), 1.68 – 1.61 (m, 2H), 1.42 – 1.34 (m, 1H), 1.33 – 1.27 (m, 1H), 0.95 – 0.93 (m, 3H), 0.93 – 0.91 (m, 1H), 0.91 – 0.88 (m, 3H), 0.80 – 0.77 (m, 3H).

^{13}C NMR (126 MHz, $CDCl_3$, 23°C, δ): 167.2, 161.6, 134.7, 128.8, 123.9, 80.8, 63.4, 48.1, 39.7, 34.2, 31.4, 25.3, 23.1, 22.1, 20.9, 16.1.

HRMS-ESI (m/z) calc'd for $C_{20}H_{25}NO_5Na$ $[M+Na]^+$, 382.1625; found, 382.1622; deviation: 0.8 ppm.

2-Methoxypropanoic acid derived redox-active ester (±)-RAE-14

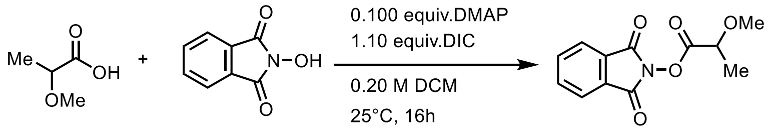

(±)-R-16, 1.00 equiv. **NHPI**, 1.00 equiv. **(±)-RAE-14**, 84%

Prepared according to a reported procedure and spectra are in good accordance with literature[10].

To a 50 mL round bottom flask containing a teflon-coated magnetic stirring bar were added 2-methoxypropanoic acid (0.62 g, 0.58 mL, 6.0 mmol, 1.0 equiv.), 4-dimethylamino-pyridine (DMAP, 73.3 mg, 0.60 mmol, 0.10 equiv.), *N*,*N*'-diisopropylcarbodiimide (DIC, 1.0 mL, 0.83 g, 6.6 mmol, 1.1 equiv.), *N*-hydroxyphthalimide (**NHPI**, 979 mg, 6.0 mmol, 1.0 equiv.) and DCM (30 mL, *c* = 0.20 M). After addition, the mixture was stirred for 16 h at 25°C. After stirring, the reaction mixture was filtered over SiO_2 (10 g), and the SiO_2 was subsequently washed with DCM (10 mL). The solvent was removed under reduced pressure, and the resulting residue was purified by column chromatography on silica gel, eluting with EtOAc/hexanes (1:3, v/v) to afford **(±)-RAE-14** (1.3 g, 84%) as a colorless solid.

NMR Spectroscopy:

^{1}H NMR (500 MHz, $CDCl_3$, 23°C, δ): 7.90 (dd, *J* = 5.5, 3.1 Hz, 2H), 7.80 (dd, *J* = 5.5, 3.1 Hz, 2H), 4.28 (q, *J* = 6.9 Hz, 1H), 3.53 (s, 3H), 1.64 (d, *J* = 6.9 Hz, 3H).

^{13}C NMR (126 MHz, $CDCl_3$, 23°C, δ): 169.6, 161.8, 135.0, 129.0, 124.2, 75.0, 58.4, 18.9.

HRMS-ESI (m/z) calc'd for $C_{12}H_{11}NO_5Na$ $[M+Na]^+$, 272.0529; found, 272.0529; deviation: 0.3 ppm.

3,3-Difluorocyclobutane-1-carboxylic acid derived redox-active ester RAE-15

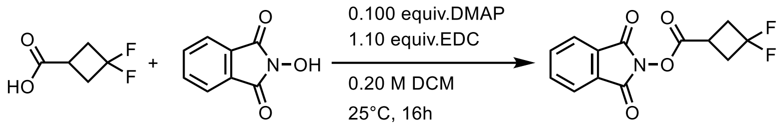

R-17, 1.00 equiv. **NHPI**, 1.00 equiv. **RAE-15**, 77%

Prepared according to a reported procedure and spectra are in good accordance with literature[16].

A round-bottom flask was charged with N-hydroxyphthalimide (**NHPI**, 979 mg, 6.0 mmol, 1.0 equiv.), 3,3-difluorocyclobutane-1-carboxylic acid (817 mg, 6.0 mmol, 1.0 equiv.), and 4-dimethylamino-pyridine (DMAP, 73.3 mg, 0.60 mmol, 0.10 equiv.). Dichloromethane was added (30 mL, *c* = 0.20 M) and the mixture was stirred vigorously. Finally, *N*-ethyl-*N*'-(3-dimethylaminopropyl)-carbodiimide hydrochloride (EDC·HCl, 1.27 g, 6.60 mmol, 1.10 equiv.) was added, and the mixture was allowed to stir for 16 h at 25°C. After stirring, the mixture was diluted with 10 mL DCM and poured onto a 1M solution of HCl (ca. 60 mL). The combined mixture was poured into a separatory funnel, and the layers were separated. The DCM layer was collected and further extracted with 1M solution of HCl (2 × ca. 60 mL). Subsequently, the DCM layer was dried over Na_2SO_4 (10 g), filtered, and the solvent was removed under reduced pressure. The resulting residue was purified by column chromatography on silica gel, eluting with EtOAc/hexanes (1:3, v/v) to afford **RAE-15** (1.3 g, 77%) as a colorless solid.

NMR Spectroscopy:

^{1}**H NMR** (500 MHz, $CDCl_3$, 23°C, δ): 7.90 (dd, *J* = 5.4, 3.1 Hz, 2H), 7.81 (dd, *J* = 5.6, 3.1 Hz, 2H), 3.39 – 3.28 (m, 1H), 3.14 – 2.95 (m, 4H).

^{13}**C NMR** (126 MHz, $CDCl_3$, 23°C, δ): 170.0 (t, *J* = 3.0 Hz), 161.8, 135.1, 128.9, 124.2, 118.2 (dd, *J* = 284.0, 270.9 Hz), 39.2 (t, *J* = 25.3 Hz), 24.2 (dd, *J* = 14.6, 5.7 Hz).

^{19}**F NMR** (471MHz, $CDCl_3$, 23°C, δ): –83.20 (d, *J* = 195.2 Hz), –96.31 (d, *J* = 195.2 Hz).

HRMS-ESI (m/z) calc'd for $C_{13}H_{10}NO_4F_2$ $[M+H]^+$, 282.0572; found, 282.0577.

3-(3,5-dichlorophenyl)propanoic acid derived redox-active ester RAE-16

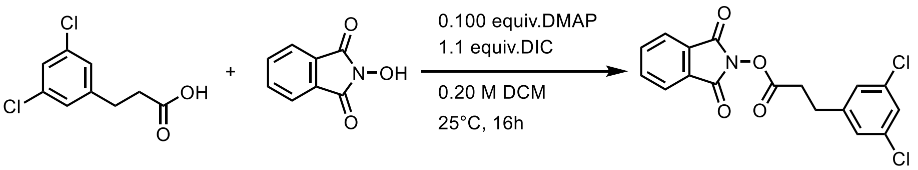

R-18, 1.00 equiv. **NHPI**, 1.00 equiv. **RAE-16**, 53%

To a 50 mL round bottom flask containing a teflon-coated magnetic stirring bar were added 3-(3,5-dichlorphenyl)propanoic acid (1.31 g, 6.0 mmol, 1.0 equiv.), 4-dimethylamino-pyridine (DMAP, 73.3 mg, 0.60 mmol, 0.10 equiv.), *N*,*N*'-diisopropylcarbodiimide (DIC, 1.0 mL, 0.83 g, 6.6 mmol, 1.1 equiv.), *N*-hydroxyphthalimide (**NHPI**, 979 mg, 6.0 mmol, 1.0 equiv.) and DCM (30 mL, *c* = 0.20 M). After addition, the

mixture was stirred for 16 h at 25°C. After stirring, the reaction mixture was filtered over SiO_2 (10 g), and the SiO_2 was subsequently washed with DCM (10 mL). The solvent was removed under reduced pressure, and the resulting residue was purified by column chromatography on silica gel, eluting with EtOAc/hexanes (1:3, v/v) to afford **RAE-16** (1.2 g, 53%) as a colorless solid.

R*f* = 0.28 (EtOAc/pentane, 2:8, v/v).

NMR Spectroscopy:

^{1}H NMR (500 MHz, $CDCl_3$, 23°C, δ): 7.93 – 7.87 (m, 2H), 7.85 – 7.77 (m, 2H), 7.30 – 7.26 (m, 1H), 7.20 (dt, *J* = 1.9, 0.6 Hz, 2H), 3.09 – 3.03 (m, 2H), 3.03 – 2.96 (m, 2H).

^{13}C NMR (126 MHz, $CDCl_3$, 23°C, δ): 169.0, 162.2, 143.2, 135.4, 135.3, 129.2, 127.5, 127.3, 124.3, 32.4, 30.3.

HRMS-CI GC (m/z) calc'd for $C_{17}H_{12}NO_4Cl_2$ $[M+H]^+$, 364.0138; found, 364.0140; deviation: –0.7 ppm.

Baclofen derived redox-active ester (±)-RAE-17

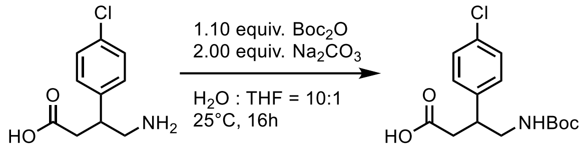

(±)-R-19, 1.00 equiv. **(±)-R-20**, 90%

Prepared according to a reported procedure and spectra are in good accordance with literature[17].

To a 25 mL round bottom flask containing a teflon-coated magnetic stirring bar were added (±)-baclofen (1.78 g, 8.34 mmol, 1.0 equiv.), THF (1.0 mL, 0.89 mg, 12 mmol, 1.5 equiv.), water (10 mL, 10 g, 0.56 mol, 67 equiv.), sodium carbonate (1.76 g, 16.6 mmol, 2.0 equiv.) and di-*tert*-butyl dicarbonate (2.00 mL, 2.00 g, 9.17 mmol, 1.10 equiv.). The mixture was stirred for 16 h at 25°C. After stirring, the mixture was acidified with HCl (10%, v/v) to pH = 2. The aqueous phase was extracted with EtOAc (3 × ca. 20 mL). The combined organic phases were washed with 150 mL brine and dried over Na_2SO_4. The solvent was removed under reduced pressure and the residue **(±)-R-20** (2.4 g, 90%) was used directly for the next step without further purification.

NMR Spectroscopy:

^{1}H NMR (500 MHz, $CDCl_3$, 23°C, δ): 7.89 (dd, *J* = 5.5, 3.1 Hz, 2H), 7.80 (dd, *J* = 5.5, 3.1 Hz, 2H), 5.21 (d, *J* = 6.7 Hz, 1H), 4.46 – 4.39 (m, 1H), 3.78 (s, 3H), 2.89 – 2.68 (m, 2H), 2.41 – 2.27 (m, 1H), 2.12 (m, 1H), 1.46 (s, 9H).

^{13}C NMR (126 MHz, $CDCl_3$, 23°C, δ): 175.2, 156.1, 139.5, 133.0, 129.0, 79.9, 45.4, 41.7, 37.6, 28.3.

HRMS-ESI (m/z) calc'd for $C_{15}H_{19}NO_4Cl$ $[M-H]^-$, 312.1008; found, 312.1011; deviation: –1.0 ppm.

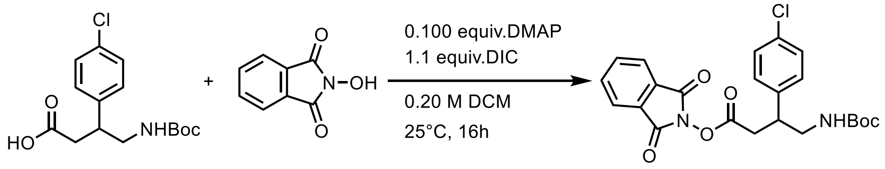

(±)-R-20, 1.00 equiv. **NHPI**, 1.00 equiv. **(±)-RAE-17**, 51%

Prepared according to a reported procedure and spectra are in good accordance with literature[18].

To a 50 mL round bottom flask containing a teflon-coated magnetic stirring bar were added **(±)-R-20** (1.88 g, 6.0 mmol, 1.0 equiv.), 4-dimethylamino-pyridine (DMAP, 73.3 mg, 0.60 mmol, 0.10 equiv.), *N*,*N*'-diisopropylcarbodiimide (DIC, 1.0 mL, 0.83 g, 6.6 mmol, 1.1 equiv.), *N*-hydroxyphthalimide (**NHPI**, 979 mg, 6.0 mmol, 1.0 equiv.) and DCM (30 mL, *c* = 0.20 M). After addition, the mixture was stirred for 16 h at 25°C. After stirring, the reaction mixture was filtered over SiO_2 (10 g), and the SiO_2 was subsequently washed with DCM (10 mL). The solvent was removed under reduced pressure, and the resulting residue was purified by column chromatography on silica gel, eluting with EtOAc/hexanes (1:3, v/v) to afford **(±)-RAE-17** (1.4 g, 51%) as a colorless solid.

NMR Spectroscopy:

^{1}H NMR (500 MHz, $CDCl_3$, 23°C, δ): 7.89 (dd, *J* = 5.6, 3.1 Hz, 2H), 7.80 (dd, *J* = 5.5, 3.1 Hz, 2H), 7.41 – 7.33 (m, 2H), 7.25 (d, *J* = 8.7 Hz, 2H), 4.72 (s, 1H), 3.66 – 3.29 (m, 3H), 3.08 (dd, *J* = 15.9, 6.1 Hz, 1H), 2.97 (dd, *J* = 15.9, 8.2 Hz, 1H), 1.44 (s, 9H).

^{13}C NMR (126 MHz, $CDCl_3$, 23°C, δ): 167.9, 161.8, 156.0, 138.7, 134.9, 133.5, 129.2, 129.1, 129.0, 124.1, 79.8, 45.6, 42.0, 35.2, 28.5, 23.7.

HRMS-ESI (m/z) calc'd for $C_{23}H_{23}N_2O_6ClNa$ $[M+Na]^+$, 481.1137; found, 481.1137; deviation: 0.0 ppm.

Synthesis of alkylated alkenes

1-Ethyl-3,5-dimethoxybenzene-derived alkylated alkene 1

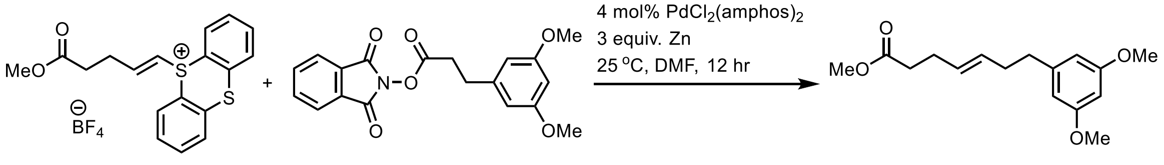

TT-1, 1.0 equiv. **RAE-1**, 2.2 equiv. **1**, 75%

Under an ambient atmosphere, the redox-active ester **RAE-1** (156 mg, 0.44 mmol, 2.2 equiv.) was added to a 4-mL vial that contained a teflon-coated magnetic stir bar. The vial was transferred to a nitrogen-filled glovebox, where zinc powder (39.2 mg, 0.60 mmol, 3.0 equiv.) and DMF (0.80 mL, *c* = 0.25) were added. The reaction mixture was stirred (800 rpm) at 25°C on a stirring plate for 2 h. In a separate 4-mL vial, $PdCl_2(amphos)_2$ (5.6 mg, 8.0 μmol, 4.0 mol%) and the alkenyl thianthrenium salt **TT-1** (83.2 mg, 0.20 mmol, 1.0 equiv.) were added under ambient atmosphere. The vial was transferred to a nitrogen-filled glovebox and DMF (0.80 mL, *c* = 0.25) was added. The mixture was stirred at 25°C for 5 min. After that, the obtained yellow

suspension was added to the first vial. The vial was sealed, and the reaction mixture was stirred (800 rpm) at 25°C for 16 h outside the glovebox. The reaction mixture was diluted with ethyl acetate (2 mL), and transferred to a separatory funnel that contained ethyl acetate (20 mL). The organic layer was washed with brine (1 × 25 mL). The aqueous layer was then extracted with ethyl acetate (3 × 20 mL). The organic layers were combined, dried over $MgSO_4$, filtered, and concentrated under reduced pressure. The resulting residue was purified by column chromatography on silica gel, eluting with EtOAc/hexanes (3:97, v/v) to afford the desired product **1** (*E*:*Z* > 20:1, 42 mg, 75%) as colorless solid.

R*f* = 0.29 (EtOAc/pentane, 1:9, v/v).

NMR Spectroscopy:

^{1}H NMR (500 MHz, $CDCl_3$, 23°C, δ): 6.33 (d, *J* = 2.3 Hz, 2H), 6.30 (t, *J* = 2.3 Hz, 1H), 5.48 (qt, *J* = 15.3, 6.1 Hz, 2H), 3.78 (s, 6H), 3.67 (s, 3H), 2.59 (dd, *J* = 8.9, 6.7 Hz, 2H), 2.40 – 2.34 (m, 2H), 2.34 – 2.26 (m, 4H).

^{13}C NMR (126 MHz, $CDCl_3$, 23°C, δ): 173.8, 160.8, 144.5, 130.9, 128.8, 106.6, 97.9, 55.4, 51.6, 36.4, 34.2, 34.2, 28.0.

HRMS-EI GC (m/z) calc'd for $C_{16}H_{22}O_4$ $[M]^+$, 278.1513; found, 278.1512; deviation: 0.2 ppm.

γ-Aminobutyric acid-derived alkylated alkene 2

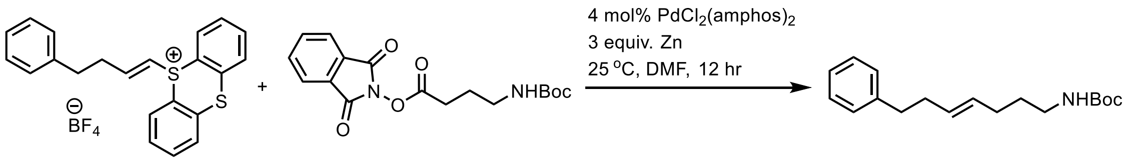

TT-2, 1.0 equiv. **RAE-2**, 2.2 equiv. **2**, 75%

Under an ambient atmosphere, the redox-active ester **RAE-2** (153 mg, 0.44 mmol, 2.2 equiv.) was added to a 4-mL vial that contained a teflon-coated magnetic stir bar. The vial was transferred to a nitrogen-filled glovebox, where zinc powder (39.2 mg, 0.60 mmol, 3.0 equiv.) and DMF (0.80 mL, *c* = 0.25) were added. The reaction mixture was stirred (800 rpm) at 25°C on a stirring plate for 2 h. In a separate 4-mL vial, $PdCl_2(amphos)_2$ (5.6 mg, 8.0 µmol, 4.0 mol%) and the alkenyl thianthrenium salt **TT-2** (86.8 mg, 0.20 mmol, 1.0 equiv.) were added under ambient atmosphere. The vial was transferred to a nitrogen-filled glovebox and DMF (0.80 mL, *c* = 0.25) was added. The mixture was stirred at 25°C for 5 min. After that, the obtained yellow suspension was added to the first vial. The vial was sealed, and the reaction mixture was stirred (800 rpm) at 25°C for 16 h. The reaction mixture was diluted with ethyl acetate (2 mL), and transferred to a separatory funnel that contained ethyl acetate (20 mL). The organic layer was washed with brine (1 × 25 mL). The aqueous layer was then extracted with ethyl acetate (3 × 20 mL). The organic layers were combined, dried over $MgSO_4$, filtered, and concentrated under reduced pressure. The resulting residue was purified by column chromatography on silica gel, eluting with EtOAc/hexanes (0:1 to 1:13, v/v) to afford the desired product **2** (*E*:*Z* > 20:1, 43 mg, 75%) as yellow oil.

R*f* = 0.28 (EtOAc/pentane, 5:95, v/v).

NMR Spectroscopy:

^{1}H NMR (500 MHz, $CDCl_3$, 23°C, δ): 7.29 – 7.24 (m, 2H), 7.21 – 7.14 (m, 3H), 5.44 (m, 2H), 4.49 (bs, 1H), 3.08 (q, *J* = 6.8 Hz, 2H), 2.66 (t, *J* = 7.8 Hz, 2H), 2.38 – 2.26 (m, 2H), 2.01 (q, *J* = 7.0 Hz, 2H), 1.52 (p, *J* = 7.3 Hz, 2H), 1.45 (s, 9H).

^{13}C NMR (126 MHz, $CDCl_3$, 23°C, δ): 156.0, 142.2, 130.4, 130.0, 128.7, 128.6, 128.4, 125.8, 79.2, 40.2, 36.1, 34.5, 29.9, 28.6.

HRMS-ESI (m/z) calc'd for $C_{18}H_{27}NO_2Na$ $[M+Na]^+$, 312.1934; found, 312.1935; deviation: –0.3 ppm.

Tetrahydropyran-derived alkylated alkene 3

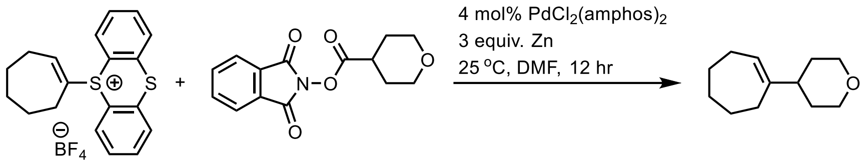

TT-3, 1.0 equiv. **RAE-3**, 2.2 equiv. **3**, 74%

Under an ambient atmosphere, the redox-active ester **RAE-3** (121 mg, 0.44 mmol, 2.2 equiv.) was added to a 4-mL vial that contained a teflon-coated magnetic stir bar. The vial was transferred to a nitrogen-filled glovebox, where zinc powder (39.2 mg, 0.60 mmol, 3.0 equiv.) and DMF (0.80 mL, *c* = 0.25) were added. The reaction mixture was stirred (800 rpm) at 25°C on a stirring plate for 2 h. In a separate 4-mL vial, $PdCl_2(amphos)_2$ (5.6 mg, 8.0 µmol, 4.0 mol%) and the alkenyl thianthrenium salt **TT-3** (79.6 mg, 0.20 mmol, 1.0 equiv.) were added under ambient atmosphere. The vial was transferred to a nitrogen-filled glovebox and DMF (0.80 mL, *c* = 0.25) was added. The mixture was stirred at 25°C for 5 min. After that, the obtained yellow suspension was added to the first vial. The vial was sealed, and the reaction mixture was stirred (800 rpm) at 25°C for 16 h. The reaction mixture was diluted with ethyl acetate (2 mL), and transferred to a separatory funnel that contained ethyl acetate (20 mL). The organic layer was washed with brine (1 × 25 mL). The aqueous layer was then extracted with ethyl acetate (3 × 20 mL). The organic layers were combined, dried over $MgSO_4$, filtered, and concentrated under reduced pressure. The resulting residue was purified by column chromatography on silica gel, eluting with EtOAc/hexanes (0:1 to 1:19, v/v) to afford the desired product **3** (27 mg, 74%) as a clear oil. Product may be volatile if left under vacuum over an extended period of time.

R*f* = 0.42 (EtOAc/pentane, 5:95, v/v).

NMR Spectroscopy:

^{1}H NMR (500 MHz, $CDCl_3$, 23°C, δ): 5.55 (t, *J* = 6.5 Hz, 1H), 4.02 – 3.95 (m, 2H), 3.39 (td, *J* = 11.4, 3.0 Hz, 2H), 2.15 – 2.05 (m, 4H), 2.02 (m, 1H), 1.73 (m, 2H), 1.57 – 1.47 (m, 4H), 1.47 – 1.36 (m, 4H).

^{13}C NMR (126 MHz, $CDCl_3$, 23°C, δ): 148.4, 124.8, 68.4, 44.7, 33.0, 31.6, 31.1, 28.3, 27.4, 27.3.

HRMS-EI GC (m/z) calc'd for $C_{12}H_{20}O$ [M]$^+$, 180.1509; found, 180.1509; deviation: 0.0 ppm.

1-Ethyl-3,5-dimethoxybenzene-derived alkylated alkene 4

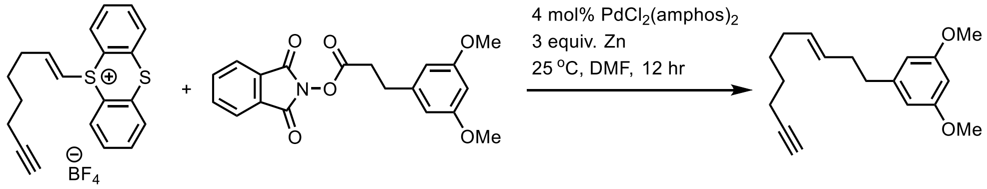

TT-4, 1.0 equiv. **RAE-1**, 2.2 equiv. **4**, 73%

Under an ambient atmosphere, the redox-active ester **RAE-1** (156 mg, 0.44 mmol, 2.2 equiv.) was added to a 4-mL vial that contained a teflon-coated magnetic stir bar. The vial was transferred to a nitrogen-filled glovebox, where zinc powder (39.2 mg, 0.60 mmol, 3.0 equiv.) and DMF (0.80 mL, *c* = 0.25) were added. The reaction mixture was stirred (800 rpm) at 25°C on a stirring plate for 2 h. In a separate 4-mL vial, $PdCl_2(amphos)_2$ (5.6 mg, 8.0 μmol, 4.0 mol%) and the alkenyl thianthrenium salt **TT-4** (82.9 mg, 0.20 mmol, 1.0 equiv.) were added under ambient atmosphere. The vial was transferred to a nitrogen-filled glovebox and DMF (0.80 mL, *c* = 0.25) was added. The mixture was stirred at 25°C for 5 min. After that, the obtained yellow suspension was added to the first vial. The vial was sealed, and the reaction mixture was stirred (800 rpm) at 25°C for 16 h outside the glovebox. The reaction mixture was diluted with ethyl acetate (2 mL), and transferred to a separatory funnel that contained ethyl acetate (20 mL). The organic layer was washed with brine (1 × 25 mL). The aqueous layer was then extracted with ethyl acetate (3 × 20 mL). The organic layers were combined, dried over $MgSO_4$, filtered, and concentrated under reduced pressure. The resulting residue was purified by preparative TLC using Et_2O/pentane (2:98, v/v) as developing solvent to afford the desired product **4** (*E*:*Z* > 20:1, 40 mg, 73%) as a yellow oil.

R_f = 0.26 (Et_2O/pentane, 4:96, v/v).

NMR Spectroscopy:

^{1}H NMR (600 MHz, $CDCl_3$, 23°C, δ): 6.35 (d, *J* = 2.3 Hz, 2H), 6.30 (t, *J* = 2.3 Hz, 1H), 5.50 – 5.39 (m, 2H), 3.78 (s, 6H), 2.64 – 2.58 (m, 2H), 2.34 – 2.26 (m, 2H), 2.18 (td, *J* = 6.9, 2.6 Hz, 2H), 2.02 – 1.98 (m, 2H), 1.94 (t, *J* = 2.6 Hz, 1H), 1.55 – 1.48 (m, 2H), 1.48 – 1.42 (m, 2H).

^{13}C NMR (126 MHz, $CDCl_3$, 23°C, δ): 160.8, 144.7, 130.7, 129.9, 106.6, 97.8, 84.7, 68.3, 55.3, 36.5, 34.3, 32.1, 28.7, 28.0, 18.3.

HRMS-EI GC (m/z) calc'd for $C_{18}H_{24}O_2$ [M]$^+$, 272.1771; found, 272.1772; deviation: –0.4 ppm.

1,1-Difluorocyclohexane-derived alkylated alkene 5

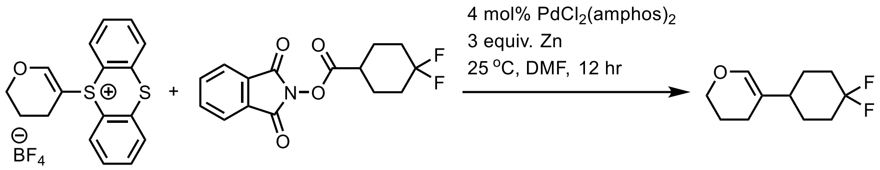

TT-5, 1.0 equiv. **RAE-4**, 2.2 equiv. **5**, 75%

Under an ambient atmosphere, the redox-active ester **RAE-4** (136 mg, 0.44 mmol, 2.2 equiv.) was added to a 4-mL vial that contained a teflon-coated magnetic stirring bar. The vial was transferred to a nitrogen-filled glovebox, where zinc powder (39.2 mg, 0,60 mmol, 3.0 equiv.) and DMF (0.80 mL, *c* = 0.25) were added. The reaction mixture was stirred (800 rpm) at 23°C for 2 h. In a separate 4-mL vial, $PdCl_2(amphos)_2$ (5.6 mg, 8.0 μmol, 4.0 mol%) and the alkenyl thianthrenium salt **TT-5** (77.2 mg, 0.20 mmol, 1.0 equiv.) were added under ambient atmosphere. The vial was transferred to a nitrogen-filled glovebox and DMF (0.80 mL, *c* = 0.25) was added. The mixture was stirred at 25°C for 5 min. After that, the obtained yellow suspension was added to the first vial. The vial was sealed, and the reaction mixture was stirred (600 rpm) at 23°C for 16 h outside the glovebox. The reaction mixture was diluted with DCM (2 mL) and transferred to a separatory funnel that contained DCM (15 mL) and brine (15 mL). The organic phase was washed with brine (2 × 15 mL), dried over $MgSO_4$, filtered, and concentrated under reduced pressure. The resulting residue was purified by column chromatography on silica gel, eluting with Et_2O/pentane (0:1 to 2:98, v/v) to afford the desired alkylation compound **5** (30 mg, 75%) as pale yellow oil.

R_f = 0.27 (Et_2O/pentane, 1:100, v/v)

NMR Spectroscopy:

^{1}H NMR (500 MHz, $CDCl_3$, 23ºC, δ): 6.27 (s, *J* = 1.4 Hz, 1H), 3.89 (t, *J* = 5.8, 4.5 Hz, 2H), 2.10 (m, 2H), 1.98 – 1.91 (m, 2H), 1.90 – 1.80 (m, 3H), 1.80 – 1.64 (m, 4H), 1.61 – 1.49 (m, 2H).

^{13}C NMR (126 MHz, $CDCl_3$, 23ºC, δ): 138.9, 125.4, 123.5 (dd, *J* = 242.1, 239.4 Hz), 121.5, 115.5 (d, *J* = 2.3 Hz), 65.6, 40.0, 33.9 (dd, *J* = 25.6, 22.5 Hz), 27.9 (d, *J* = 9.8 Hz), 22.7, 21.2.

^{19}F NMR (471 MHz, $CDCl_3$, 23ºC, δ): –91.40 (d, *J* = 234.8 Hz), –101.82 – –102.82 (m).

HRMS-EI GC (m/z) calc'd for $C_{11}H_{16}OF_2$ $[M]^+$, 202.1164; found, 202.1166; deviation: –1.2 ppm.

1-Benzoylpiperidine-derived alkylated alkene 6

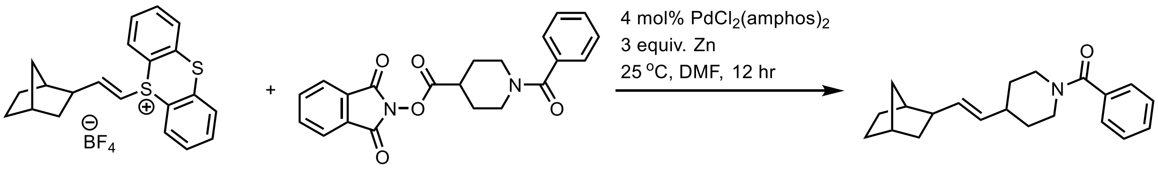

TT-6, 1.0 equiv. **RAE-5**, 2.2 equiv. **6**, 73%

Under an ambient atmosphere, the redox-active ester **RAE-5** (166 mg, 0.44 mmol, 2.2 equiv.) was added to a 4-mL vial that contained a teflon-coated magnetic stir bar. The vial was transferred to a nitrogen-filled

glovebox, where zinc powder (39.2 mg, 0.60 mmol, 3.0 equiv.) and DMF (0.80 mL, *c* = 0.25) were added. The reaction mixture was stirred (800 rpm) at 25°C on a stirring plate for 2 h. In a separate 4-mL vial, $PdCl_2(amphos)_2$ (5.6 mg, 8.0 µmol, 4.0 mol%) and the alkenyl thianthrenium salt **TT-6** (84.8 mg, 0.20 mmol, 1.0 equiv.) were added under ambient atmosphere. The vial was transferred to a nitrogen-filled glovebox and DMF (0.80 mL, *c* = 0.25) was added. The mixture was stirred at 25°C for 5 minutes. After that, the obtained yellow suspension was added to the first vial. The vial was sealed, and the reaction mixture was stirred (800 rpm) at 25°C for 16 h. The reaction mixture was diluted with ethyl acetate (2 mL), and transferred to a separatory funnel that contained ethyl acetate (20 mL). The organic layer was washed with brine (1 × 25 mL). The aqueous layer was then extracted with ethyl acetate (3 × 20 mL). The organic layers were combined, dried over $MgSO_4$, filtered, and concentrated under reduced pressure. The resulting residue was purified by column chromatography on silica gel, eluting with EtOAc/hexanes (0:1 to 1:6, v/v) to afford the desired product **6** (*E*:*Z* > 20:1, 45 mg, 73%) as colorless solid.

$\mathbf{R}_f$ = 0.38 (EtOAc/pentane, 1:9, v/v).

NMR Spectroscopy:

^{1}H NMR (500 MHz, $CDCl_3$, 23°C, δ): 7.39 (s, 5H), 5.42 – 5.21 (m, 2H), 4.66 (bs, 1H), 3.72 (bs, 1H), 3.00 (bs, 1H), 2.82 (bs, 1H), 2.21 (s, 1H), 2.16 (m, 1H), 2.03 (m, 1H), 2.00 – 1.96 (m, 1H), 1.79 (bs, 1H), 1.55 – 1.43 (m, 3H), 1.38 (d, *J* = 9.5 Hz, 1H), 1.32 (m, 2H), 1.24 – 1.17 (m, 3H), 1.15 – 1.04 (m, 2H).

^{13}C NMR (151 MHz, $CDCl_3$, 23°C, δ): 170.4, 136.5, 135.3, 131.1, 129.5, 128.5, 127.0, 47.9, 45.0, 42.8, 42.4, 39.0, 38.1, 36.7, 35.7, 33.0, 32.1, 29.8, 29.1.

HRMS-EI GC (m/z) calc'd for $C_{21}H_{27}NO$ $[M]^+$, 309.2087; found, 309.2089; deviation: –0.6 ppm.

N-tert-butyloxycarbonyl-azetidine-derived alkylated alkene 7

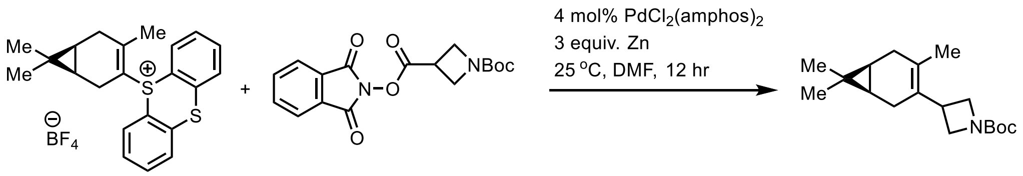

TT-7, 1.0 equiv. **RAE-6**, 2.2 equiv. **7**, 82%

Under an ambient atmosphere, the redox-active ester **RAE-6** (152 mg, 0.44 mmol, 2.2 equiv.) was added to a 4-mL vial that contained a teflon-coated magnetic stir bar. The vial was transferred to a nitrogen-filled glovebox, where zinc powder (39.2 mg, 0.60 mmol, 3.0 equiv.) and DMF (0.80 mL, *c* = 0.25) were added. The reaction mixture was stirred (800 rpm) at 25°C on a stirring plate for 2 h. In a separate 4-mL vial, $PdCl_2(amphos)_2$ (5.6 mg, 8.0 µmol, 4.0 mol%) and the alkenyl thianthrenium salt **TT-7** (87.7 mg, 0.20 mmol, 1.0 equiv.) were added under ambient atmosphere. The vial was transferred to a nitrogen-filled glovebox and DMF (0.80 mL, *c* = 0.25) was added. The mixture was stirred at 25°C for 5 min. After that, the obtained yellow suspension was added to the first vial. The vial was sealed, and the reaction mixture was stirred (800 rpm) at 25°C for 16 h. The reaction mixture was diluted with ethyl acetate (2 mL), and transferred to a separatory

funnel that contained ethyl acetate (20 mL). The organic layer was washed with brine (1 × 25 mL). The aqueous layer was then extracted with ethyl acetate (3 × 20 mL). The organic layers were combined, dried over $MgSO_4$, filtered, and concentrated under reduced pressure. The resulting residue was purified by column chromatography on silica gel, eluting with EtOAc/hexanes (0:1 to 1:9, v/v) to afford the desired product **7** (48 mg, 82%) as clear oil.

R*f* = 0.65 (EtOAc/pentane, 3:7, v/v).

NMR Spectroscopy:

^{1}H NMR (500 MHz, $CDCl_3$, 23°C, δ): 4.00 – 3.93 (m, 2H), 3.88 (dd, *J* = 8.5, 6.5 Hz, 1H), 3.80 (dd, *J* = 8.4, 6.6 Hz, 1H), 3.73 – 3.65 (m, 1H), 2.49 – 2.38 (m, 1H), 2.32 – 2.23 (m, 1H), 2.13 – 2.05 (m, 1H), 1.91 – 1.84 (m, 1H), 1.52 (s, 3H), 1.44 (s, 9H), 1.01 (s, 3H), 0.76 (m, 1H), 0.72 (s, 3H), 0.67 (m, 1H).

^{13}C NMR (126 MHz, $CDCl_3$, 23°C, δ): 156.5, 127.3, 125.5, 79.3, 53.0, 30.7, 28.6, 28.3, 28.0, 20.3, 19.3, 18.0, 17.0, 13.5.

HRMS-ESI (m/z) calc'd for $C_{18}H_{29}NO_2Na$ $[M+Na]^+$, 314.2090; found, 314.2091; deviation: –0.1 ppm.

Butanone-derived alkylated alkene 8

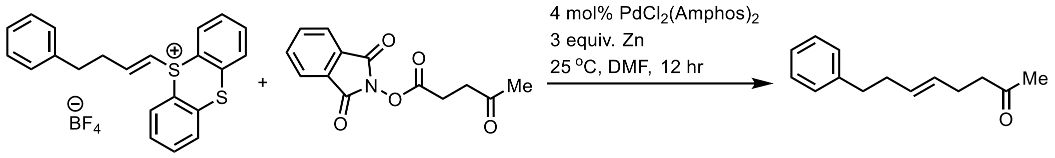

TT-2, 1.0 equiv. **RAE-7**, 2.2 equiv. **8**, 62%

Under an ambient atmosphere, the redox-active ester **RAE-7** (95.1 mg, 0.44 mmol, 2.2 equiv.) was added to a 4-mL vial that contained a teflon-coated magnetic stir bar. The vial was transferred to a nitrogen-filled glovebox, where zinc powder (39.2 mg, 0.60 mmol, 3.0 equiv.) and DMF (0.80 mL, *c* = 0.25) were added. The reaction mixture was stirred (800 rpm) at 25°C on a stirring plate for 2 h. In a separate 4-mL vial, $PdCl_2(amphos)_2$ (5.6 mg, 8.0 µmol, 4.0 mol%) and the alkenyl thianthrenium salt **TT-2** (86.8 mg, 0.20 mmol, 1.0 equiv.) were added under ambient atmosphere. The vial was transferred to a nitrogen-filled glovebox and DMF (0.80 mL, *c* = 0.25) was added. The mixture was stirred at 25°C for 5 min. After that, the obtained yellow suspension was added to the first vial. The vial was sealed, and the reaction mixture was stirred (800 rpm) at 25°C for 16 h. The reaction mixture was diluted with ethyl acetate (2 mL), and transferred to a separatory funnel that contained ethyl acetate (20 mL). The organic layer was washed with brine (1 × 25 mL). The aqueous layer was then extracted with ethyl acetate (3 × 20 mL). The organic layers were combined, dried over $MgSO_4$, filtered, and concentrated under reduced pressure. The resulting residue was purified by column chromatography on silica gel, eluting with EtOAc/hexanes (0:1 to 8:92, v/v) to afford the desired product **8** (*E*:*Z* > 20:1, 25 mg, 62%) as clear oil.

R*f* = 0.31 (EtOAc/pentane, 1:9, v/v).

NMR Spectroscopy:

^{1}H NMR (500 MHz, $CDCl_3$, 23°C, δ): 7.30 – 7.27 (m, 2H), 7.20 – 7.15 (m, 3H), 5.53 – 5.36 (m, 2H), 2.66 (t, J = 7.8 Hz, 2H), 2.46 (t, J = 7.3 Hz, 2H), 2.34 – 2.21 (m, 4H), 2.12 (s, 3H).

^{13}C NMR (126 MHz, $CDCl_3$, 23°C, δ): 208.6, 142.1, 130.6, 129.2, 128.6, 128.4, 125.9, 43.6, 36.0, 34.4, 30.1, 26.9.

HRMS-EI GC (m/z) calc'd for $C_{14}H_{18}O$ $[M]^+$, 202.1352; found, 202.1352; deviation: 0.1 ppm.

Cyclohexanone-derived alkylated alkene 9

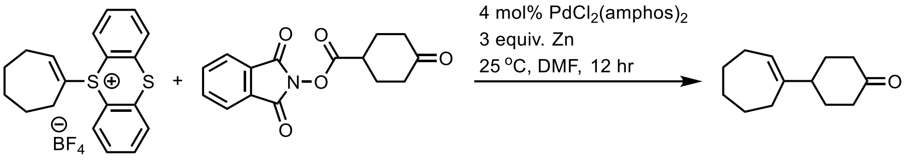

TT-3, 1.0 equiv. **RAE-8**, 2.2 equiv. **9**, 42%

Under an ambient atmosphere, the redox-active ester **RAE-8** (126 mg, 0.44 mmol, 2.2 equiv.) was added to a 4-mL vial that contained a teflon-coated magnetic stir bar. The vial was transferred to a nitrogen-filled glovebox, where zinc powder (39.2 mg, 0.60 mmol, 3.0 equiv.) and DMF (0.80 mL, c = 0.25) were added. The reaction mixture was stirred (800 rpm) at 25°C on a stirring plate for 2 h. In a separate 4-mL vial, $PdCl_2(amphos)_2$ (5.6 mg, 8.0 µmol, 4.0 mol%) and the alkenyl thianthrenium salt **TT-3** (79.6 mg, 0.20 mmol, 1.0 equiv.) were added under ambient atmosphere. The vial was transferred to a nitrogen-filled glovebox and DMF (0.80 mL, c = 0.25) was added. The mixture was stirred at 25°C for 5 min. After that, the obtained yellow suspension was added to the first vial. The vial was sealed, and the reaction mixture was stirred (800 rpm) at 25°C for 16 h. The reaction mixture was diluted with ethyl acetate (2 mL), and transferred to a separatory funnel that contained ethyl acetate (20 mL). The organic layer was washed with brine (1 × 25 mL). The aqueous layer was then extracted with ethyl acetate (3 × 20 mL). The organic layers were combined, dried over $MgSO_4$, filtered, and concentrated under reduced pressure. The resulting residue was purified by column chromatography on silica gel, eluting with EtOAc/hexanes (0:1 to 8:92, v/v) to afford the desired product **9** (16 mg, 42%) as a clear oil.

R_f = 0.31 (EtOAc/pentane, 1:9, v/v).

NMR Spectroscopy:

^{1}H NMR (500 MHz, $CDCl_3$, 23°C, δ): 5.63 (t, J = 1.1 Hz, 1H), 2.51 – 2.35 (m, 4H), 2.35 – 2.26 (m, 1H), 2.20 – 2.06 (m, 4H), 2.05 – 1.96 (m, 2H), 1.80 – 1.69 (m, 2H), 1.68 – 1.55 (m, 2H), 1.51 – 1.39 (m, 4H).

^{13}C NMR (126 MHz, $CDCl_3$, 23°C, δ): 212.2, 147.4, 125.5, 45.9, 41.3, 33.0, 31.5, 31.4, 28.3, 27.3, 27.3.

HRMS-ESI (m/z) calc'd for $C_{13}H_{20}ONa$ $[M+Na]^+$, 215.1406; found, 215.1408; deviation: –0.7 ppm.

Tert-butyl-propionate-derived alkylated alkene 10

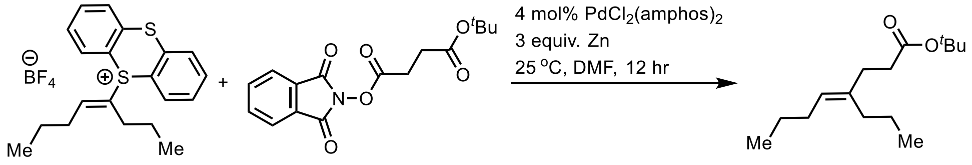

TT-8, 1.0 equiv. **RAE-9**, 2.2 equiv. **10**, 81%

Under an ambient atmosphere, the redox-active ester **RAE-9** (140 mg, 0.44 mmol, 2.2 equiv.) was added to a 4-mL vial that contained a teflon-coated magnetic stir bar. The vial was transferred to a nitrogen-filled glovebox, where zinc powder (39.2 mg, 0.60 mmol, 3.0 equiv.) and DMF (0.80 mL, *c* = 0.25) were added. The reaction mixture was stirred (800 rpm) at 25°C on a stirring plate for 2 h. In a separate 4-mL vial, $PdCl_2(amphos)_2$ (5.6 mg, 8.0 μmol, 4.0 mol%) and the alkenyl thianthrenium salt **TT-8** (82.9 mg, 0.20 mmol, 1.0 equiv.) were added under ambient atmosphere. The vial was transferred to a nitrogen-filled glovebox and DMF (0.80 mL, *c* = 0.25) was added. The mixture was stirred at 25°C for 5 min. After that, the obtained yellow suspension was added to the first vial. The vial was sealed, and the reaction mixture was stirred (800 rpm) at 25°C for 16 h. The reaction mixture was diluted with ethyl acetate (2 mL), and transferred to a separatory funnel that contained ethyl acetate (20 mL). The organic layer was washed with brine (1 × 25 mL). The aqueous layer was then extracted with ethyl acetate (3 × 20 mL). The organic layers were combined, dried over $MgSO_4$, filtered, and concentrated under reduced pressure. The resulting residue was purified by column chromatography on silica gel, eluting with EtOAc/hexanes (0:1 to 5:95, v/v) to afford the desired product **10** (*E*:*Z* > 20:1, 39 mg, 81%) as a clear oil. Product may be volatile if left under vacuum over an extended period of time

$\mathbf{R}_f$ = 0.38 (EtOAc/pentane, 5:95, v/v).

NMR Spectroscopy:

^{1}**H NMR** (500 MHz, $CDCl_3$, 23°C, δ): 5.14 (t, *J* = 7.2 Hz, 1H), 2.37 – 2.28 (m, 2H), 2.28 – 2.22 (m, 2H), 2.02 – 1.92 (m, 4H), 1.43 (s, 9H), 1.42 – 1.36 (m, 2H), 1.36 – 1.30 (m, 2H), 0.89 (td, *J* = 7.3, 2.6 Hz, 6H).

^{13}**C NMR** (126 MHz, $CDCl_3$, 23°C, δ): 173.1, 137.9, 125.5, 80.1, 34.7, 32.4, 32.1, 30.0, 28.3, 23.3, 21.7, 14.3, 14.0.

HRMS-ESI (m/z) calc'd for $C_{15}H_{28}O_2Na$ $[M+Na]^+$, 263.1982; found, 263.1982; deviation: 0.0 ppm.

Indometacin-derived alkylated alkene 11

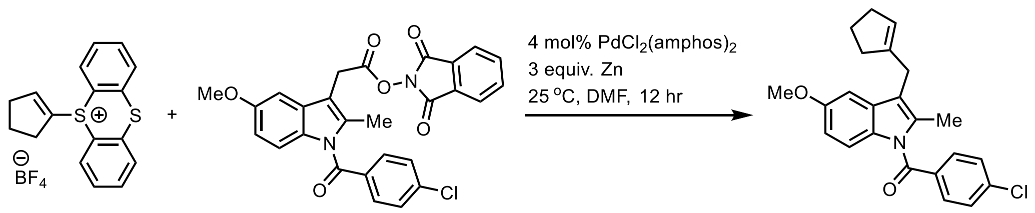

TT-9, 1.0 equiv. **RAE-10**, 2.2 equiv. **11**, 44%

Under an ambient atmosphere, the redox-active ester **RAE-10** (221 mg, 0.44 mmol, 2.2 equiv.) was added to a 4-mL vial that contained a teflon-coated magnetic stir bar. The vial was transferred to a nitrogen-filled glovebox, where zinc powder (39.2 mg, 0,60 mmol, 3.0 equiv.) and DMF (0.80 mL, *c* = 0.25) were added. The reaction mixture was stirred (800 rpm) at 25°C on a stirring plate for 2 h. In a separate 4-mL vial, $PdCl_2(amphos)_2$ (5.6 mg, 8.0 μmol, 4.0 mol%) and the alkenyl thianthrenium salt **TT-9** (74.0 mg, 0.20 mmol, 1.0 equiv.) were added under ambient atmosphere. The vial was transferred to a nitrogen-filled glovebox and DMF (0.80 mL, *c* = 0.25) was added. The mixture was stirred at 25°C for 5 min. After that, the obtained yellow suspension was added to the first vial. The vial was sealed, and the reaction mixture was stirred (800 rpm) at 25°C for 16 h outside the glovebox. The reaction mixture was diluted with ethyl acetate (2 mL), and transferred to a separatory funnel that contained ethyl acetate (20 mL). The organic layer was washed with brine (1 × 25 mL). The aqueous layer was then extracted with ethyl acetate (3 × 20 mL). The organic layers were combined, dried over $MgSO_4$, filtered, and concentrated under reduced pressure. The resulting residue was purified by column chromatography on silica gel, eluting with EtOAc/hexanes (3:97, v/v) to afford the desired product **11** (33 mg, 44%) as colorless solid.

R_f = 0.30 (DCM, 100 v/v).

NMR Spectroscopy:

^{1}H NMR (500 MHz, $CDCl_3$, 23°C, δ): 7.67 – 7.62 (m, 2H), 7.51 – 7.44 (m, 2H), 6.93 – 6.86 (m, 2H), 6.65 (dd, *J* = 9.0, 2.5 Hz, 1H), 5.35 (p, *J* = 1.8 Hz, 1H), 3.82 (s, 3H), 3.42 – 3.38 (m, 2H), 2.31 (s, 3H), 2.31 – 2.24 (m, 1H), 1.93 – 1.82 (m, 2H).

^{13}C NMR (126 MHz, $CDCl_3$, 23°C, δ): 168.5, 156.0, 142.1, 139.1, 134.6, 134.5, 131.6, 131.2, 131.1, 129.2, 125.3, 117.9, 115.0, 111.0, 102.1, 55.8, 35.3, 32.5, 26.2, 23.6, 13.5.

HRMS-ESI (m/z) calc'd for $C_{23}H_{23}NO_2Cl$ $[M+H]^+$, 380.1412; found, 380.1407; deviation: 1.3 ppm.

Methoxymethane-derived alkylated alkene 12

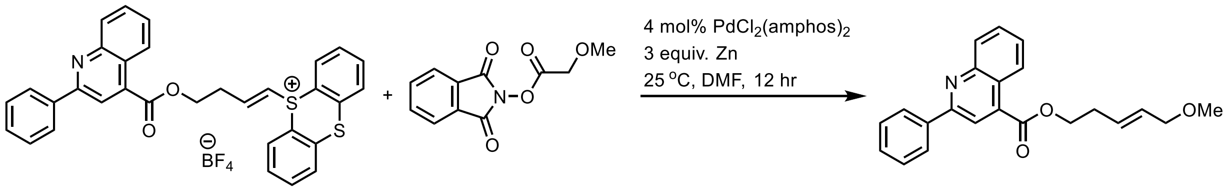

TT-10, 1.0 equiv. **RAE-11**, 2.2 equiv. **12**, 68%

Under an ambient atmosphere, the redox-active ester **RAE-11** (103 mg, 0.44 mmol, 2.2 equiv.) was added to a 4-mL vial that contained a teflon-coated magnetic stir bar. The vial was transferred to a nitrogen-filled glovebox, where zinc powder (39.2 mg, 0.60 mmol, 3.0 equiv.) and DMF (0.80 mL, *c* = 0.25) were added. The reaction mixture was stirred (800 rpm) at 25°C on a stirring plate for 2 h. In a separate 4-mL vial, $PdCl_2(amphos)_2$ (5.6 mg, 8.0 μmol, 4.0 mol%) and the alkenyl thianthrenium salt **TT-10** (121 mg, 0.20 mmol, 1.0 equiv.) were added under ambient atmosphere. The vial was transferred to a nitrogen-filled glovebox and DMF (0.80 mL, *c* = 0.25) was added. The mixture was stirred at 25°C for 5 min. After that, the obtained yellow

suspension was added to the first vial. The vial was sealed, and the reaction mixture was stirred (800 rpm) at 25°C for 16 h outside the glovebox. The reaction mixture was diluted with ethyl acetate (2 mL), and transferred to a separatory funnel that contained ethyl acetate (20 mL). The organic layer was washed with brine (1 × 25 mL). The aqueous layer was then extracted with ethyl acetate (3 × 20 mL). The organic layers were combined, dried over $MgSO_4$, filtered, and concentrated under reduced pressure. The resulting residue was purified by column chromatography on silica gel, eluting with EtOAc/hexanes (3:97, v/v) to afford the desired product **12** (*E*:*Z* > 20:1, 47 mg, 68%) as yellow oil.

R_f = 0.35 (EtOAc/pentane, 5:95, v/v).

NMR Spectroscopy:

^{1}H NMR (500 MHz, $CDCl_3$, 23°C, δ): 8.73 (d, *J* = 8.7 Hz, 1H), 8.39 (s, 1H), 8.26 – 8.18 (m, 3H), 7.84 – 7.73 (m, 1H), 7.66 – 7.59 (m, 1H), 7.58 – 7.53 (m, 2H), 7.52 – 7.47 (m, 1H), 5.87 – 5.74 (m, 2H), 4.54 (t, *J* = 6.7 Hz, 2H), 3.92 (d, *J* = 4.9 Hz, 3H), 3.32 (s, 4H), 2.64 (q, *J* = 6.6 Hz, 2H).

^{13}C NMR (126 MHz, $CDCl_3$, 23°C, δ): 166.5, 156.9, 149.4, 139.0, 136.1, 130.5, 130.0, 129.9, 129.8, 129.1, 129.0, 127.9, 127.6, 125.6, 124.1, 120.4, 73.0, 65.0, 58.1, 31.9.

HRMS-EI GC (m/z) calc'd for $C_{22}H_{21}NO_3$ $[M]^+$, 347.1520; found, 347.1516; deviation: –1.2 ppm.

1-Benzoylpiperidine-derived alkylated alkene (±)-13

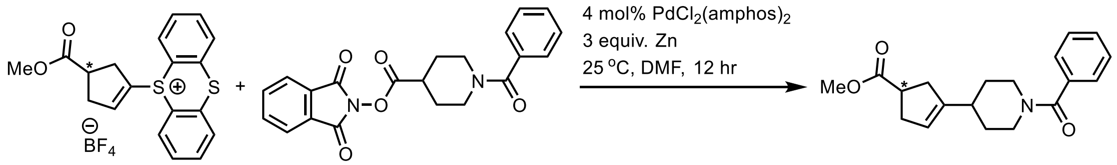

(±)-TT-11, 1.0 equiv. **RAE-5**, 2.2 equiv. **(±)-13**, 54%

Under an ambient atmosphere, the redox-active ester **RAE-5** (166 mg, 0.44 mmol, 2.2 equiv.) was added to a 4-mL vial that contained a teflon-coated magnetic stirring bar. The vial was transferred to a nitrogen-filled glovebox, where zinc powder (39.2 mg, 0.60 mmol, 3.0 equiv.) and DMF (0.80 mL, *c* = 0.25) were added. The reaction mixture was stirred (800 rpm) at 23°C for 2 h. In a separate 4-mL vial, $PdCl_2(amphos)_2$ (5.6 mg, 8.0 μmol, 4.0 mol%) and the alkenyl thianthrenium salt **(±)-TT-11** (85.6 mg, 0.20 mmol, 1.0 equiv.) were added under ambient atmosphere. The vial was transferred to a nitrogen-filled glovebox and DMF (0.80 mL, *c* = 0.25) was added. The mixture was stirred at 25°C for 5 min. After that, the obtained yellow suspension was added to the first vial. The vial was sealed, and the reaction mixture was stirred (600 rpm) at 23°C for 16 h. The reaction mixture was diluted with DCM (2 mL) and transferred to a separatory funnel using DCM (15 mL) and brine (15 mL). The organic phase was washed with brine (2 × 15 mL), dried over $MgSO_4$, filtered, and concentrated by rotary evaporation. The resulting residue was purified by column chromatography on silica gel, eluting with acetone/pentane (0:1 to 3:97, v/v) to afford the desired product **(±)-13** (34 mg, 54%) as a pale yellow oil.

R_f = 0.15 (acetone/pentane, 1:20, v/v)

NMR Spectroscopy:

^{1}H NMR (600 MHz, $CDCl_3$, 23°C, δ): 7.47 – 7.35 (m, 5H), 5.27 (s, 1H), 4.82 – 4.61 (m, 1H), 3.82 – 3.75 (m, 1H), 3.72 – 3.63 (m, 3H), 3.19 – 3.10 (m, 1H), 3.06 – 2.97 (m, 1H), 2.77 (t, *J* = 12.8 Hz, 1H), 2.69 – 2.48 (m, 4H), 2.24 (q, *J* = 13.5 Hz, 1H), 1.91 (t, *J* = 12.8 Hz, 1H), 1.73 (t, *J* = 14.1 Hz, 1H), 1.50 – 1.41 (m, 1H), 1.38 – 1.28 (m, 1H).

^{13}C NMR (151 MHz, $CDCl_3$, 23°C, δ): 177.1, 170.4, 145.6, 135.9, 129.6, 128.6, 126.7, 120.9, 52.3, 48.0, 42.3, 41.5, 37.6, 36.8, 36.0, 31.2, 30.3.

HRMS-ESI (m/z) calc'd for $C_{19}H_{23}NO_3$ $[M]^+$, 313.1672; found, 313.1674; deviation: –0.6 ppm.

N-tert-butyloxycarbonyl-azetidine-derived alkylated alkene 14

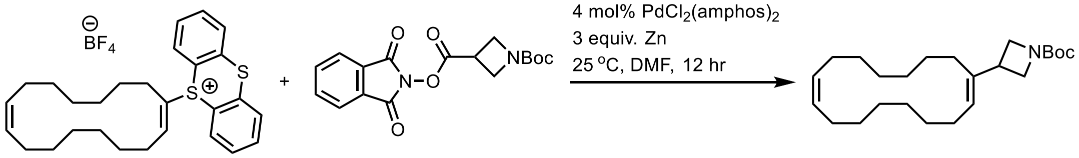

TT-12, 1.0 equiv. **RAE-6**, 2.2 equiv. **14**, 45%

Under an ambient atmosphere, the redox-active ester **RAE-6** (152 mg, 0.44 mmol, 2.2 equiv.) was added to a 4-mL vial that contained a teflon-coated magnetic stirring bar. The vial was transferred to a nitrogen-filled glovebox, where zinc powder (40 mg, 0.60 mmol, 3.0 equiv.) and DMF (0.80 mL, *c* = 0.25) were added. The reaction mixture was stirred (800 rpm) at 23°C for 2 h. In a separate 4-mL vial, $PdCl_2(amphos)_2$ (5.6 mg, 8.0 µmol, 4.0 mol%) and the alkenyl thianthrenium salt **TT-12** (110 mg, 0.20 mmol, 1.0 equiv.) were added under ambient atmosphere. The vial was transferred to a nitrogen-filled glovebox and DMF (0.80 mL, *c* = 0.25) was added. The mixture was stirred at 25°C for 5 min. After that, the obtained yellow suspension was added to the first vial. The vial was sealed, and the reaction mixture was stirred (600 rpm) at 23°C for 16 h. The reaction mixture was diluted with DCM (2 mL) and transferred to a separatory funnel using DCM (15 mL) and brine (15 mL). The organic phase was washed with brine (2 x 15 mL), dried over $MgSO_4$, filtered, and concentrated by rotary evaporation. The resulting residue was purified by column chromatography on silica gel, eluting with EtOAc/hexanes (0:1 to 1:4, v/v) to afford the desired product **14** (34 mg, 45%) as colorless oil.

R*f* = 0.31 (EtOAc/pentane, 1:20, v/v)

NMR Spectroscopy:

^{1}H NMR (500 MHz, $CDCl_3$, 23°C, δ): 5.39 – 5.34 (m, 2H), 5.31 – 5.26 (m, 1H), 3.98 (t, *J* = 8.5 Hz, 2H), 3.78 (t, *J* = 7.5 Hz, 2H), 3.24 – 3.17 (m, 1H), 2.10 – 1.95 (m, 8H), 1.43 (s, 9H), 1.38 – 1.27 (m, 16H).

^{13}C NMR (126 MHz, $CDCl_3$, 23°C, δ): 156.7, 138.7, 130.2, 130.1, 125.3, 79.4, 34.3, 29.5, 29.1, 29.0, 28.9, 28.8, 28.6, 28.3, 28.2, 27.4, 27.1, 26.8.

HRMS-ESI (m/z) calc'd for $C_{24}H_{41}NO_2Na$ $[M+Na]^+$, 398.3029; found 398.3027; deviation: 0.7 ppm.

Tetrahydropyran-derived alkylated alkene 15

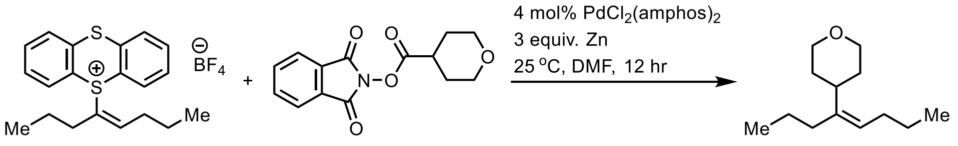

TT-13, 1.0 equiv. **RAE-3**, 2.2 equiv. **15**, 80%

Under an ambient atmosphere, the redox-active ester **RAE-3** (121 mg, 0.44 mmol, 2.2 equiv.) was added to a 4-mL vial that contained a teflon-coated magnetic stir bar. The vial was transferred to a nitrogen-filled glovebox, where zinc powder (39.2 mg, 0.60 mmol, 3.0 equiv.) and DMF (0.80 mL, *c* = 0.25) were added. The reaction mixture was stirred (800 rpm) at 25°C on a stirring plate for 2 h. In a separate 4-mL vial, $PdCl_2(amphos)_2$ (5.6 mg, 8.0 μmol, 4.0 mol%) and the alkenyl thianthrenium salt **TT-13** (82.9 mg, 0.20 mmol, 1.0 equiv.) were added under ambient atmosphere. The vial was transferred to a nitrogen-filled glovebox and DMF (0.80 mL, *c* = 0.25) was added. The mixture was stirred at 25°C for 5 min. After that, the obtained yellow suspension was added to the first vial. The vial was sealed, and the reaction mixture was stirred (800 rpm) at 25°C for 16 h. The reaction mixture was diluted with ethyl acetate (2 mL), and transferred to a separatory funnel that contained ethyl acetate (20 mL). The organic layer was washed with brine (1 × 25 mL). The aqueous layer was then extracted with ethyl acetate (3 × 20 mL). The organic layers were combined, dried over $MgSO_4$, filtered, and concentrated under reduced pressure. The resulting residue was purified by column chromatography on silica gel, eluting with EtOAc/hexanes (0:1 to 1:19, v/v) to afford the desired product **15** (*Z*:*E* > 20:1, 31 mg, 80%) as colorless oil. Product may be volatile if left under vacuum over an extended period of time.

$\mathbf{R_f}$ = 0.40 (EtOAc/pentane, 5:95, v/v).

NMR Spectroscopy:

^{1}H NMR (500 MHz, $CDCl_3$, 23°C, δ): 5.10 (t, *J* = 7.1 Hz, 1H), 4.02 – 3.96 (m, 2H), 3.46 – 3.40 (m, 2H), 2.65 (tt, *J* = 12.2, 3.7 Hz, 1H), 2.06 – 1.98 (m, 2H), 1.94 – 1.87 (m, 2H), 1.79 – 1.67 (m, 2H), 1.47 – 1.39 (m, 2H), 1.39 – 1.32 (m, 4H), 0.95 – 0.86 (m, 6H).

^{13}C NMR (126 MHz, $CDCl_3$, 23°C, δ): 142.0, 124.9, 68.6, 37.5, 35.1, 31.2, 29.5, 23.5, 22.6, 14.3, 14.0.

HRMS-EI GC (m/z) calc’d for $C_{13}H_{24}O$ $[M]^+$, 196.1822; found, 196.1821; deviation: 0.2 ppm.

1,1-Difluorocyclohexane-derived alkylated alkene 16

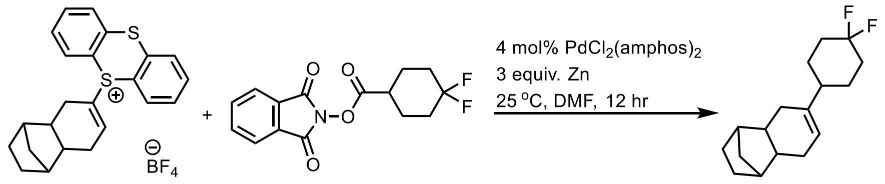

TT-14, 1.0 equiv. **RAE-4**, 2.2 equiv. **16**, 71%

Under an ambient atmosphere, the redox-active ester **RAE-4** (136 mg, 0.44 mmol, 2.2 equiv.) was added to a

4-mL vial that contained a teflon-coated magnetic stir bar. The vial was transferred to a nitrogen-filled glovebox, where zinc powder (39.2 mg, 0.30 mmol, 3.0 equiv.) and DMF (0.80 mL, *c* = 0.25) were added. The reaction mixture was stirred (800 rpm) at 25°C on a stirring plate for 2 h. In a separate 4-mL vial, $PdCl_2(amphos)_2$ (5.6 mg, 8.0 µmol, 4.0 mol%) and the alkenyl thianthrenium salt **TT-14** (90.1 mg, 0.20 mmol, 1.0 equiv.) were added under ambient atmosphere. The vial was transferred to a nitrogen-filled glovebox and DMF (0.80 mL, *c* = 0.25) was added. The mixture was stirred at 25°C for 5 min. After that, the obtained yellow suspension was added to the first vial. The vial was sealed, and the reaction mixture was stirred (800 rpm) at 25°C for 16 h. The reaction mixture was diluted with ethyl acetate (2 mL), and transferred to a separatory funnel that contained ethyl acetate (20 mL). The organic layer was washed with brine (1 × 25 mL). The aqueous layer was then extracted with ethyl acetate (3 × 20 mL). The organic layers were combined, dried over $MgSO_4$, filtered, and concentrated under reduced pressure. The resulting residue was purified by column chromatography on neutral alumina with pentane to afford the desired product **16** (38 mg, 71%) as colorless solid.

R*f* = 0.48 (EtOAc/pentane, 5:95, v/v).

NMR Spectroscopy:

^{1}H NMR (500 MHz, $CDCl_3$, 23°C, δ): 5.57 – 5.52 (m, 1H), 2.25 – 2.06 (m, 4H), 2.00 – 1.90 (m, 3H), 1.81 – 1.69 (m, 4H), 1.69 – 1.62 (m, 1H), 1.60 – 1.42 (m, 8H), 1.23 – 1.17 (m, 2H), 1.07 – 0.98 (m, 1H).

^{13}C NMR (126 MHz, $CDCl_3$, 23°C, δ): 143.7 (d, *J* = 2.4 Hz), 123.7 (dd, *J* = 242.1, 239.3 Hz), 120.5, 44.3, 43.7, 43.3, 43.2, 43.1, 33.8 (dd, *J* = 25.5, 22.2 Hz), 33.4, 30.7, 29.9, 29.8, 28.2, 27.5 (dd, *J* = 16.6, 9.6 Hz).

^{19}F NMR (471 MHz, $CDCl_3$, 23°C, δ): –91.28 (d, *J* = 234.2 Hz), –101.12 – –102.88 (m).

HRMS-EI GC (m/z) calc'd for $C_{17}H_{24}F_2$ $[M]^+$, 266.1841; found, 266.1842; deviation: –0.7 ppm.

2,4-D-derived alkylated alkene 17

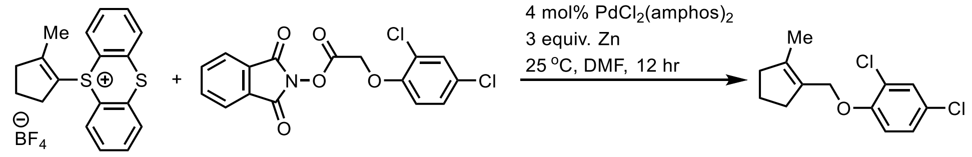

TT-15, 1.0 equiv. **RAE-12**, 2.2 equiv. **17**, 71%

Under an ambient atmosphere, the redox-active ester **RAE-12** (161 mg, 0.44 mmol, 2.2 equiv.) was added to a 4-mL vial that contained a teflon-coated magnetic stir bar. The vial was transferred to a nitrogen-filled glovebox, where zinc powder (39.2 mg, 0.60 mmol, 3.0 equiv.) and DMF (0.80 mL, *c* = 0.25) were added. The reaction mixture was stirred (800 rpm) at 25°C on a stirring plate for 2 h. In a separate 4-mL vial, $PdCl_2(amphos)_2$ (5.6 mg, 8.0 µmol, 4.0 mol%) and the alkenyl thianthrenium salt **TT-15** (76.9 mg, 0.20 mmol, 1.0 equiv.) were added under ambient atmosphere. The vial was transferred to a nitrogen-filled glovebox and DMF (0.80 mL, *c* = 0.25) was added. The mixture was stirred at 25°C for 5 min. After that, the obtained yellow

suspension was added to the first vial. The vial was sealed, and the reaction mixture was stirred (800 rpm) at 25°C for 16 h. The reaction mixture was diluted with ethyl acetate (2 mL), and transferred to a separatory funnel that contained ethyl acetate (20 mL). The organic layer was washed with brine (1 × 25 mL). The aqueous layer was then extracted with ethyl acetate (3 × 20 mL). The organic layers were combined, dried over $MgSO_4$, filtered, and concentrated under reduced pressure. The resulting residue was purified by column chromatography on silica gel, eluting with EtOAc/hexanes (0:1 to 1:19, v/v) to afford the desired product **17** (37 mg, 71%) as pale yellow oil.

R_f = 0.52 (EtOAc/pentane, 1:9, v/v).

NMR Spectroscopy:

^{1}H NMR (500 MHz, $CDCl_3$, 23°C, δ): 7.35 (d, *J* = 2.5 Hz, 1H), 7.15 (dd, *J* = 8.8, 2.6 Hz, 1H), 6.85 (d, *J* = 8.8 Hz, 1H), 4.62 (s, 2H), 2.51 – 2.45 (m, 2H), 2.40 – 2.32 (m, 2H), 1.80-1.86 (m, 2H), 1.74 (s, 3H).
^{13}C NMR (126 MHz, $CDCl_3$, 23°C, δ): 153.5, 138.3, 130.2, 130.1, 127.5, 125.7, 124.2, 114.8, 66.6, 39.0, 34.5, 21.6, 14.3.

HRMS-CI GC (m/z) calc'd for $C_{13}H_{13}OCl_2$ $[M-H]^+$, 255.0338; found, 255.0337; deviation: 0.4 ppm.

Methoxymethane-derived alkylated alkene 18

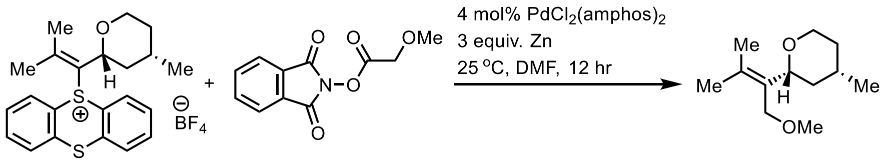

TT-16, 1.0 equiv. **RAE-11**, 2.2 equiv. **18**, 46%

Under an ambient atmosphere, the redox-active ester **RAE-11** (103 mg, 0.44 mmol, 2.2 equiv.) was added to a 4-mL vial that contained a teflon-coated magnetic stir bar. The vial was transferred to a nitrogen-filled glovebox, where zinc powder (39.2 mg, 0.60 mmol, 3.0 equiv.) and DMF (0.80 mL, *c* = 0.25) were added. The reaction mixture was stirred (800 rpm) at 25°C on a stirring plate for 2 h. In a separate 4-mL vial, $PdCl_2(amphos)_2$ (5.6 mg, 8.0 µmol, 4.0 mol%) and the alkenyl thianthrenium salt **TT-16** (91.3 mg, 0.20 mmol, 1.0 equiv.) were added under ambient atmosphere. The vial was transferred to a nitrogen-filled glovebox and DMF (0.80 mL, *c* = 0.25) was added. The mixture was stirred at 25°C for 5 min. After that, the obtained yellow suspension was added to the first vial. The vial was sealed, and the reaction mixture was stirred (800 rpm) at 25°C for 16 h. The reaction mixture was diluted with ethyl acetate (2 mL), and transferred to a separatory funnel that contained ethyl acetate (20 mL). The organic layer was washed with brine (1 × 25 mL). The aqueous layer was then extracted with ethyl acetate (3 × 20 mL). The organic layers were combined, dried over $MgSO_4$, filtered, and concentrated under reduced pressure. The resulting residue was purified by column chromatography on silica gel, eluting with EtOAc/hexanes (0:1 to 1:10, v/v) to afford the desired product **18** (18 mg, 46%) as colorless oil. Product may be volatile if left under vacuum over an extended period of time.

R$_f$ = 0.47 (EtOAc/pentane, 1:9, v/v).

NMR Spectroscopy:

^{1}H NMR (500 MHz, $CDCl_3$, 23°C, δ): 4.19 (dd, *J* = 11.4, 2.2 Hz, 1H), 4.05 – 3.95 (m, 1H), 3.95 (s, 2H), 3.51 – 3.41 (m, 1H), 3.33 (s, 3H), 1.79 (s, 3H), 1.77 (s, 3H), 1.69 – 1.58 (m, 1H), 1.56 – 1.47 (m, 2H), 1.28 – 1.12 (m, 2H), 0.94 (d, *J* = 6.5 Hz, 3H).

^{13}C NMR (126 MHz, $CDCl_3$, 23°C, δ): 136.0, 130.9, 77.1, 68.3, 68.1, 58.2, 38.8, 34.7, 30.8, 22.5, 21.2, 20.4.

HRMS-ESI (m/z) calc'd for $C_{12}H_{22}O_2Na$ $[M+Na]^+$, 221.1512; found, 221.1515; deviation: –1.2 ppm.

(−)-Menthyloxyacetic acid derived alkylated alkene 19

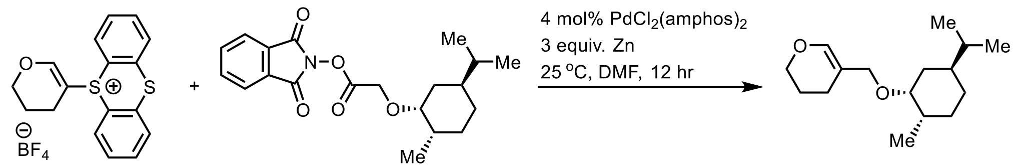

TT-5, 1.0 equiv. **RAE-13**, 2.2 equiv. **19**, 60%

Under an ambient atmosphere, the redox-active ester **RAE-13** (158 mg, 0.44 mmol, 2.2 equiv.) was added to a 4-mL vial that contained a teflon-coated magnetic stirring bar. The vial was transferred to a nitrogen-filled glovebox, where zinc powder (39.2 mg, 0.60 mmol, 3.0 equiv.) and DMF (0.80 mL, *c* = 0.25) were added. The reaction mixture was stirred (800 rpm) at 23°C for 2 h. In a separate 4-mL vial, $PdCl_2(amphos)_2$ (5.6 mg, 8.0 µmol, 4.0 mol%) and the alkenyl thianthrenium salt **TT-5** (77.2 mg, 0.20 mmol, 1.0 equiv.) were added under ambient atmosphere. The vial was transferred to a nitrogen-filled glovebox and DMF (0.80 mL, *c* = 0.25) was added. The mixture was stirred at 25°C for 5 min. After that, the obtained yellow suspension was added to the first vial. The vial was sealed, and the reaction mixture was stirred (600 rpm) at 23°C for 16 h. The reaction mixture was diluted with DCM (2 mL) and transferred to a separatory funnel using DCM (15 mL) and brine (15 mL). The organic phase was washed with brine (2 × 15 mL), dried over $MgSO_4$, filtered, and concentrated by rotary evaporation. The resulting residue was purified by column chromatography on silica gel, eluting with Et_2O/pentane (0:1 to 3:97, v/v) to afford the desired product **19** (30 mg, 60%) as colorless oil.

R$_f$ = 0.18 (Et_2O/pentane, 1:50, v/v)

NMR Spectroscopy:

^{1}H NMR (500 MHz, $CDCl_3$, 23°C, δ): 6.44 (s, 1H), 3.96 – 3.89 (m, 3H), 3.69 (d, *J* = 11.0 Hz, 1H), 3.04 (td, *J* = 10.6, 4.2 Hz, 1H), 2.25 – 2.19 (m, 1H), 2.17 – 2.01 (m, 3H), 1.92 – 1.83 (m, 2H), 1.69 – 1.58 (m, 2H), 1.39 – 1.27 (m, 1H), 1.27 – 1.19 (m, 1H), 1.01 – 0.94 (m, 1H), 0.91 (d, *J* = 6.6 Hz, 3H), 0.89 (d, *J* = 7.1 Hz, 3H), 0.87 – 0.78 (m, 2H), 0.76 (d, *J* = 7.0 Hz, 3H).

^{13}C NMR (126 MHz, $CDCl_3$, 23°C, δ): 142.6, 110.9, 77.9, 70.3, 65.8, 48.5, 40.6, 34.8, 31.7, 25.6, 23.4, 22.5, 22.3, 21.9, 21.2, 16.2.

HRMS-ESI (m/z) calc'd for $C_{16}H_{28}O_2Na$ $[M+Na]^+$, 275.1982; found, 275.1984; deviation: -0.9 ppm.

Methoxyethane derived alkylated alkene 20

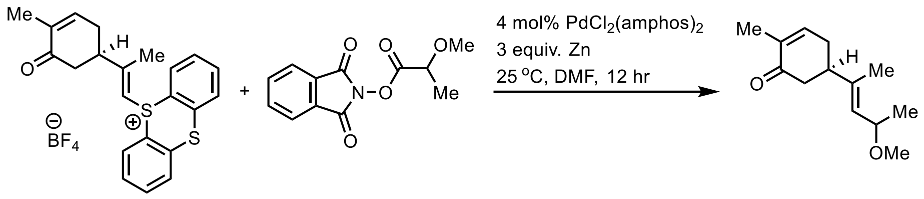

TT-17, 1.0 equiv. **RAE-14**, 2.2 equiv. **20**, 54%

Under an ambient atmosphere, the redox-active ester **(±)-RAE-14** (110 mg, 0.44 mmol, 2.2 equiv.) was added to a 4-mL vial that contained a teflon-coated magnetic stir bar. The vial was transferred to a nitrogen-filled glovebox, where zinc powder (39.2 mg, 0.60 mmol, 3.0 equiv.) and DMF (0.80 mL, *c* = 0.25) were added. The reaction mixture was stirred (800 rpm) at 25°C on a stirring plate for 2 h. In a separate 4-mL vial, $PdCl_2(amphos)_2$ (5.6 mg, 8.0 μmol, 4.0 mol%) and the alkenyl thianthrenium salt **TT-17** (90.5 mg, 0.20 mmol, 1.0 equiv.) were added under ambient atmosphere. The vial was transferred to a nitrogen-filled glovebox and DMF (0.80 mL, *c* = 0.25) was added. The mixture was stirred at 25°C for 5 min. After that, the obtained yellow suspension was added to the first vial. The vial was sealed, and the reaction mixture was stirred (800 rpm) at 25°C for 16 h. The reaction mixture was diluted with ethyl acetate (2 mL), and transferred to a separatory funnel that contained ethyl acetate (20 mL). The organic layer was washed with brine (1 × 25 mL). The aqueous layer was then extracted with ethyl acetate (3 × 20 mL). The organic layers were combined, dried over $MgSO_4$, filtered, and concentrated under reduced pressure. The resulting residue was purified by column chromatography on silica gel, eluting with EtOAc/hexanes (0:1 to 1:9, v/v) to afford the desired product **20** (*E*:*Z* > 20:1, *dr* = 1:1, 22 mg, 54%) as colorless oil.

$\mathbf{R}_f$ = 0.22 (EtOAc/pentane, 1:9, v/v).

NMR Spectroscopy:

> **^{1}H NMR** (500 MHz, $CDCl_3$, 23°C, δ): 6.77 – 6.71 (m, 1H), 5.15 – 5.12 (m, 1H), 4.11 – 4.01 (m, 1H), 3.22 (s, 3H), 2.72 – 2.62 (m, 1H), 2.58 – 2.50 (m, 1H), 2.45 – 2.35 (m, 2H), 2.34 – 2.26 (m, 1H), 1.80 – 1.76 (m, 3H), 1.69 (s, 3H), 1.18 (d, *J* = 6.3 Hz, 3H).

> **^{13}C NMR** (126 MHz, $CDCl_3$, 23°C, δ): 199.9, 144.7, 139.4, 135.6, 127.8, 73.2, 55.8, 44.3, 43.2, 31.4, 21.1, 15.8, 14.7.

HRMS-EI GC (m/z) calc'd for $C_{13}H_{20}O_2$ $[M]^+$, 208.1458; found, 208.1459; deviation: –0.8 ppm.

Tert-butyl-propionate-derived alkylated alkene 21

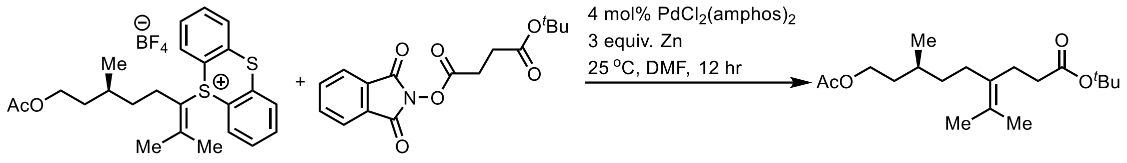

TT-18, 1.0 equiv. **RAE-9**, 2.2 equiv. **21**, 61%

Under an ambient atmosphere, the redox-active ester **RAE-9** (140 mg, 0.44 mmol, 2.2 equiv.) was added to a 4-mL vial that contained a teflon-coated magnetic stir bar. The vial was transferred to a nitrogen-filled glovebox, where zinc powder (39.2 mg, 0.60 mmol, 3.0 equiv.) and DMF (0.80 mL, *c* = 0.25) were added. The reaction mixture was stirred (800 rpm) at 25°C on a stirring plate for 2 h. In a separate 4-mL vial $PdCl_2(amphos)_2$ (5.6 mg, 8.0 μmol, 4.0 mol%), and the alkenyl thianthrenium salt **TT-18** (100 mg, 0.20 mmol, 1.0 equiv.) were added under ambient atmosphere. The vial was transferred to a nitrogen-filled glovebox and DMF (0.80 mL, *c* = 0.25) was added. The mixture was stirred at 25°C for 5 min. After that, the obtained yellow suspension was added to the first vial. The vial was sealed, and the reaction mixture was stirred (800 rpm) at 25°C for 16 h. The reaction mixture was diluted with ethyl acetate (2 mL), and transferred to a separatory funnel that contained ethyl acetate (20 mL). The organic layer was washed with brine (1 × 25 mL). The aqueous layer was then extracted with ethyl acetate (3 × 20 mL). The organic layers were combined, dried over $MgSO_4$, filtered, and concentrated under reduced pressure. The resulting residue was purified by column chromatography on silica gel with eluting with EtOAc/hexanes (0:1 to 1:9, v/v) to afford the desired product **21** (40 mg, 61%) as colorless oil.

R_f = 0.44 (EtOAc/pentane, 1:9, v/v).

NMR Spectroscopy:

^{1}H NMR (500 MHz, $CDCl_3$, 23°C, δ): 4.15 – 4.03 (m, 2H), 2.32 – 2.25 (m, 2H), 2.25 – 2.18 (m, 2H), 2.04 (s, 3H), 1.89 – 2.01 (m, 2H), 1.71 – 1.66 (m, 1H), 1.64 (s, 3H), 1.62 (s, 3H), 1.53 – 1.48 (m, 1H), 1.47 – 1.42 (m, 10H), 1.39 – 1.29 (m, 1H), 1.26 – 1.13 (m, 1H), 0.93 (d, *J* = 6.6 Hz, 3H).

^{13}C NMR (126 MHz, $CDCl_3$, 23°C, δ): 173.2, 171.4, 131.0, 125.9, 80.2, 63.2, 36.0, 35.6, 35.4, 34.9, 30.3, 29.6, 28.3, 28.0, 24.7, 21.2, 20.4, 20.4, 19.5.

HRMS-ESI (m/z) calc'd for $C_{19}H_{34}O_4Na$ $[M+Na]^+$, 349.2349; found, 349.2352; deviation: –0.9 ppm.

γ-Aminobutyric acid-derived alkylated alkene 22

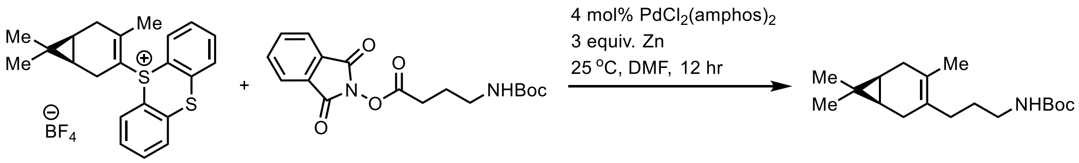

TT-7, 1.0 equiv. **RAE-2**, 2.2 equiv. **22**, 67%

Under an ambient atmosphere, the redox-active ester **RAE-2** (153 mg, 0.44 mmol, 2.2 equiv.) was added to a

4-mL vial that contained a teflon-coated magnetic stir bar. The vial was transferred to a nitrogen-filled glovebox, where zinc powder (39.2 mg, 0.60 mmol, 3.0 equiv.) and DMF (0.80 mL, *c* = 0.25) were added. The reaction mixture was stirred (800 rpm) at 25°C on a stirring plate for 2 h. In a separate 4-mL vial, $PdCl_2(amphos)_2$ (5.6 mg, 8.0 µmol, 4.0 mol%) and the alkenyl thianthrenium salt **TT-7** (87.7 mg, 0.20 mmol, 1.0 equiv.) were added under ambient atmosphere. The vial was transferred to a nitrogen-filled glovebox and DMF (0.80 mL, *c* = 0.25) was added. The mixture was stirred at 25°C for 5 min. After that, the obtained yellow suspension was added to the first vial. The vial was sealed, and the reaction mixture was stirred (800 rpm) at 25°C for 16 h. The reaction mixture was diluted with ethyl acetate (2 mL), and transferred to a separatory funnel that contained ethyl acetate (20 mL). The organic layer was washed with brine (1 × 25 mL). The aqueous layer was then extracted with ethyl acetate (3 × 20 mL). The organic layers were combined, dried over $MgSO_4$, filtered, and concentrated under reduced pressure. The resulting residue was purified by column chromatography on silica gel with a solvent mixture EtOAc/hexanes (0:1 to 1:9, v/v) to afford the desired product **22** (39 mg, 67%) as clear oil.

R*f* = 0.55 (EtOAc/pentane, 1:9, v/v).

NMR Spectroscopy:

^{1}H NMR (500 MHz, $CDCl_3$, 23°C, δ): 4.51 (bs, 1H), 3.07 (q, *J* = 6.8 Hz, 2H), 2.30 – 2.19 (m, 2H), 2.01 – 1.93 (m, 2H), 1.90 – 1.79 (m, 2H), 1.55 (s, 3H), 1.54 – 1.47 (m, 2H), 1.43 (s, 9H), 1.00 (s, 3H), 0.72 (s, 3H), 0.68 – 0.64 (m, 2H).

^{13}C NMR (126 MHz, $CDCl_3$, 23°C, δ): 156.1, 127.0, 124.7, 79.2, 40.9, 30.7, 28.8, 28.6, 28.3, 27.4, 25.0, 19.0, 18.3, 18.2, 16.7, 13.4.

HRMS-ESI (m/z) calc'd for $C_{18}H_{31}NO_2Na$ $[M+Na]^+$, 316.2247; found, 316.2249; deviation: –0.7 ppm.

1,1-Difluorocyclobutane derived alkylated alkene 23

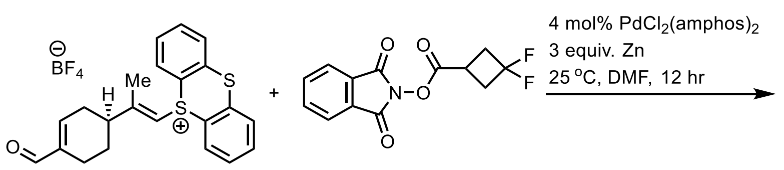

TT-19, 1.0 equiv. **RAE-15**, 2.2 equiv. **23**, 52%

Under an ambient atmosphere, the redox-active ester **RAE-15** (124 mg, 0.44 mmol, 2.2 equiv.) was added to a 4-mL vial that contained a teflon-coated magnetic stir bar. The vial was transferred to a nitrogen-filled glovebox, where zinc powder (39.2 mg, 0.60 mmol, 3.0 equiv.) and DMF (0.80 mL, *c* = 0.25) were added. The reaction mixture was stirred (800 rpm) at 25°C on a stirring plate for 2 h. In a separate 4-mL vial $PdCl_2(amphos)_2$ (5.6 mg, 8.0 µmol, 4.0 mol%), and the alkenyl thianthrenium salt **TT-19** (90.5 mg, 0.20 mmol, 1.0 equiv.) were added under ambient atmosphere. The vial was transferred to a nitrogen-filled glovebox and DMF (0.80 mL, *c* = 0.25) was added. The mixture was stirred at 25°C for 5 min. After that, the obtained yellow suspension was added to the first vial. The vial was sealed, and the reaction mixture was stirred (800 rpm) at 25°C for 16 h. The reaction mixture was diluted with ethyl acetate (2 mL), and transferred to a separatory

funnel that contained ethyl acetate (20 mL). The organic layer was washed with brine (1 × 25 mL). The aqueous layer was then extracted with ethyl acetate (3 × 20 mL). The organic layers were combined, dried over $MgSO_4$, filtered, and concentrated under reduced pressure. The resulting residue was purified by column chromatography on silica gel with EtOAc/hexanes (0:1 to 1:8, v/v) to afford the desired product **23** (*E*:*Z* ~ 10:1, 25 mg, 52%) as yellow oil. Product may be volatile if left under vacuum over an extended period of time.

R*f* = 0.34 (EtOAc/pentane, 1:9, v/v).

NMR Spectroscopy:

^{1}H NMR (500 MHz, $CDCl_3$, 23°C, δ): 9.42 (s, 1H), 6.85 – 6.76 (m, 1H), 5.24 (d, *J* = 8.3 Hz, 1H), 3.03 – 2.88 (m, 1H), 2.86 – 2.69 (m, 2H), 2.53 – 2.33 (m, 2H), 2.31 – 2.22 (m, 3H), 2.22 – 2.17 (m, 1H), 2.13 – 2.05 (m, 1H), 1.87 – 1.78 (m, 1H), 1.61 (s, 3H), 1.50 – 1.36 (m, 1H).

^{13}C NMR (126 MHz, $CDCl_3$, 23°C, δ): 194.0, 150.7, 141.3, 139.9, 127.3, 120.2 (dd, *J* = 284.6, 272.3 Hz), 42.3 (dd, *J* = 23.1, 21.1 Hz), 31.7, 26.5, 21.9, 21.6 (dd, *J* = 14.2, 4.9 Hz), 14.5.

^{19}F NMR (471 MHz, $CDCl_3$, 23°C, δ): –82.07 – –82.71 (m), –97.40 – –97.99 (m).

HRMS-ESI (m/z) calc'd for $C_{14}H_{18}OF_2Na$ $[M+Na]^+$, 263.1218; found, 263.1220; deviation: –0.8 ppm.

1-Ethyl-3,5-dichlorobenzene derived alkylated alkene 24

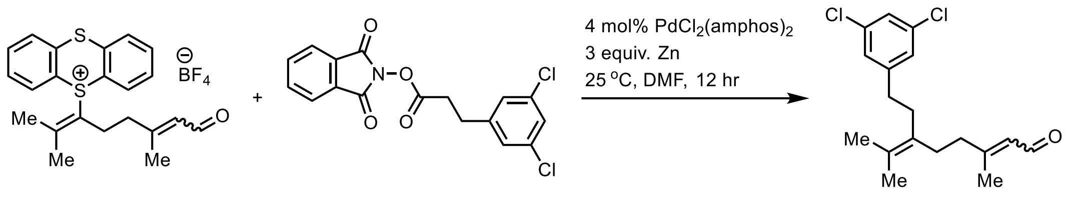

TT-20, 1.0 equiv. **RAE-16**, 2.2 equiv. **24**, 68%

Under an ambient atmosphere, the redox-active ester **RAE-16** (160 mg, 0.44 mmol, 2.2 equiv.) was added to a 4-mL vial that contained a teflon-coated magnetic stir bar. The vial was transferred to a nitrogen-filled glovebox, where zinc powder (39.2 mg, 0.60 mmol, 3.0 equiv.) and DMF (0.80 mL, *c* = 0.25) were added. The reaction mixture was stirred (800 rpm) at 25°C on a stirring plate for 2 h. In a separate 4-mL vial, $PdCl_2(amphos)_2$ (5.6 mg, 8.0 μmol, 4.0 mol%) and the alkenyl thianthrenium salt **TT-20** (90.9 mg, 0.20 mmol, 1.0 equiv.) were added under ambient atmosphere. The vial was transferred to a nitrogen-filled glovebox and DMF (0.80 mL, *c* = 0.25) was added. The mixture was stirred at 25°C for 5 min. After that, the obtained yellow suspension was added to the first vial. The vial was sealed, and the reaction mixture was stirred (800 rpm) at 25°C for 16 h. The reaction mixture was diluted with ethyl acetate (2 mL), and transferred to a separatory funnel that contained ethyl acetate (20 mL). The organic layer was washed with brine (1 × 25 mL). The aqueous layer was then extracted with ethyl acetate (3 × 20 mL). The organic layers were combined, dried over $MgSO_4$, filtered, and concentrated under reduced pressure. The resulting residue was purified by column chromatography on silica gel, eluting with EtOAc/hexanes (0:1 to 1:8, v/v) to afford the desired

product **24** (44 mg, 68%) as colorless oil.

R*f* = 0.31 (EtOAc/pentane, 1:9, v/v).

NMR Spectroscopy:

^{1}H NMR (500 MHz, $CDCl_3$, 23°C, δ): 10.00 (d, *J* = 8.1 Hz, 1H), 9.81 (d, *J* = 8.0 Hz, 1H), 7.22 – 7.17 (m, 2H), 7.04 (m, 4H), 5.88 (t, *J* = 7.8 Hz, 2H), 2.62 – 2.55 (m, 6H), 2.31 – 2.22 (m, 7H), 2.22 – 2.20 (m, 5H), 2.19 (s, 3H), 2.01 (s, 2H), 1.66 (s, 3H), 1.64 (s, 3H), 1.59 (s, 3H), 1.57 (s, 4H).

^{13}C NMR (126 MHz, $CDCl_3$, 23°C, δ): 191.4, 190.5, 163.8, 163.6, 145.6, 145.5, 134.8, 129.3, 129.2, 129.0, 128.7, 128.1, 127.4, 127.0, 126.2, 126.1, 39.7, 34.6, 34.5, 34.2, 34.1, 31.8, 31.7, 30.5, 25.4, 20.6, 20.4, 20.3, 17.9.

HRMS-ESI (m/z) calc'd for $C_{18}H_{22}OCl_2Na$ $[M+Na]^+$, 347.0940; found, 347.0940; deviation: 0.1 ppm.

Baclofen derived alkylated alkene (±)-25

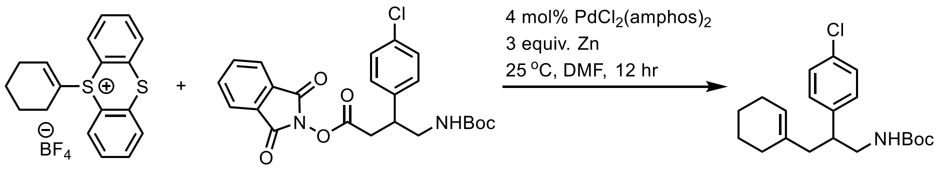

TT-21, 1.0 equiv. **(±)-RAE-17**, 2.2 equiv. **(±)-25**, 55%

Under an ambient atmosphere, the redox-active ester **(±)-RAE-17** (202 mg, 0.44 mmol, 2.2 equiv.) was added to a 4-mL vial that contained a teflon-coated magnetic stir bar. The vial was transferred to a nitrogen-filled glovebox, where zinc powder (39.2 mg, 0.60 mmol, 3.0 equiv.) and DMF (0.80 mL, *c* = 0.25) were added. The reaction mixture was stirred (800 rpm) at 25°C on a stirring plate for 2 h. In a separate 4-mL vial, $PdCl_2(amphos)_2$ (5.6 mg, 8.0 μmol, 4.0 mol%) and the alkenyl thianthrenium salt **TT-21** (76.8 mg, 0.20 mmol, 1.0 equiv.) were added under ambient atmosphere. The vial was transferred to a nitrogen-filled glovebox and DMF (0.80 mL, *c* = 0.25) was added. The mixture was stirred at 25°C for 5 min. After that, the obtained yellow suspension was added to the first vial. The vial was sealed, and the reaction mixture was stirred (800 rpm) at 25°C for 16 h. The reaction mixture was diluted with ethyl acetate (2 mL), and transferred to a separatory funnel that contained ethyl acetate (20 mL). The organic layer was washed with brine (1 × 25 mL). The aqueous layer was then extracted with ethyl acetate (3 × 20 mL). The organic layers were combined, dried over $MgSO_4$, filtered, and concentrated under reduced pressure. The resulting residue was purified by column chromatography on silica gel with a solvent mixture of EtOAc/hexanes (0:1 to 1:9, v/v) to afford the desired product **(±)-25** (38 mg, 55%).

R*f* = 0.48 (EtOAc/pentane, 1:9, v/v).

NMR Spectroscopy:

^{1}**H NMR** (500 MHz, $CDCl_3$, 23°C, δ): 7.30 – 7.22 (m, 2H), 7.11 – 7.08 (m, 2H), 5.32 (bs, 1H), 4.35 (bs, 1H), 3.49 – 3.44 (m, 1H), 3.14 – 3.05 (m, 1H), 2.94 (p, *J* = 7.5 Hz, 1H), 2.21 (ddd, *J* = 51.8, 13.9, 7.6 Hz, 2H), 1.96 – 1.77 (m, 4H), 1.62 – 1.42 (m, 4H), 1.39 (s, 9H).

^{13}**C NMR** (151 MHz, $CDCl_3$, 23°C, δ): 155.9, 141.6, 134.9, 132.3, 129.3, 128.7, 124.0, 79.3, 45.9, 43.5, 43.1, 28.5, 28.4, 25.3, 23.0, 22.4.

HRMS-ESI (m/z) calc'd for $C_{20}H_{28}NO_2ClNa$ $[M+Na]^+$, 372.1701; found, 372.1704; deviation: –0.8 ppm.

N-tert-butyloxycarbonyl-azetidine derived alkylated alkene 27

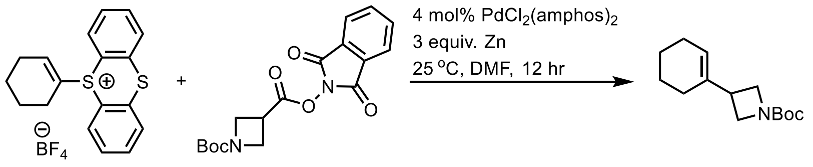

TT-21, 1.0 equiv. **RAE-6,** 2.2 equiv. **27**, 80%

Under an ambient atmosphere, the redox-active ester **RAE-6** (152 mg, 0.44 mmol, 2.2 equiv.) was added to a 4-mL vial that contained a teflon-coated magnetic stir bar. The vial was transferred to a nitrogen-filled glovebox, where zinc powder (39.2 mg, 0.60 mmol, 3.0 equiv.) and DMF (0.80 mL, *c* = 0.25) were added. The reaction mixture was stirred (800 rpm) at 60°C on a stirring plate for 2 h. In a separate 4-mL vial, $PdCl_2(amphos)_2$ (5.6 mg, 8.0 μmol, 4.0 mol%) and the alkenyl thianthrenium salt **TT-21** (77 mg, 0.20 mmol, 1.0 equiv.) were added under ambient atmosphere. The vial was transferred to a nitrogen-filled glovebox and DMF (0.80 mL, *c* = 0.25) was added. The mixture was stirred at 25°C for 5 min. After that, the obtained yellow suspension was added to the first vial. The vial was sealed, and the reaction mixture was stirred (800 rpm) at 60°C for 16 h. The reaction mixture was diluted with ethyl acetate (2 mL), and transferred to a separatory funnel that contained ethyl acetate (20 mL). The organic layer was washed with brine (1 × 25 mL). The aqueous layer was then extracted with ethyl acetate (3 × 20 mL). The organic layers were combined, dried over $MgSO_4$, filtered, and concentrated under reduced pressure. The resulting residue was purified by column chromatography on silica gel, eluting with EtOAc/hexanes (0:1 to 3:97, v/v) to afford the desired product **27** (38 mg, 80%) as colorless oil.

R_f = 0.55 (EtOAc/pentane, 1:9, v/v).

NMR Spectroscopy:

^{1}**H NMR** (500 MHz, $CDCl_3$, 23°C, δ): 5.53 – 5.50 (m, 1H), 3.97 (t, *J* = 8.5 Hz, 2H), 3.81 (dd, *J* = 8.4, 6.4 Hz, 2H), 3.14 – 3.05 (m, 1H), 2.04 – 2.00 (m, 2H), 1.96– 1.92 (m, 2H), 1.68 – 1.61 (m, 2H), 1.58 – 1.54 (m, 2H), 1.43 (s, 9H).

^{13}**C NMR** (151 MHz, $CDCl_3$, 23°C, δ): 156.6, 136.5, 122.1, 79.3, 35.2, 28.6, 25.5, 25.2, 22.8, 22.5.

HRMS-ESI (m/z) calc'd for $C_{14}H_{23}NO_2Na$ $[M+Na]^+$, 260.1621; found, 260.1622; deviation: –0.4 ppm.

SPECTROSCOPIC DATA

^{1}H NMR of methyl pent-4-enoate derived thianthrenium salt TT-1

CDCl$_3$, 23°C

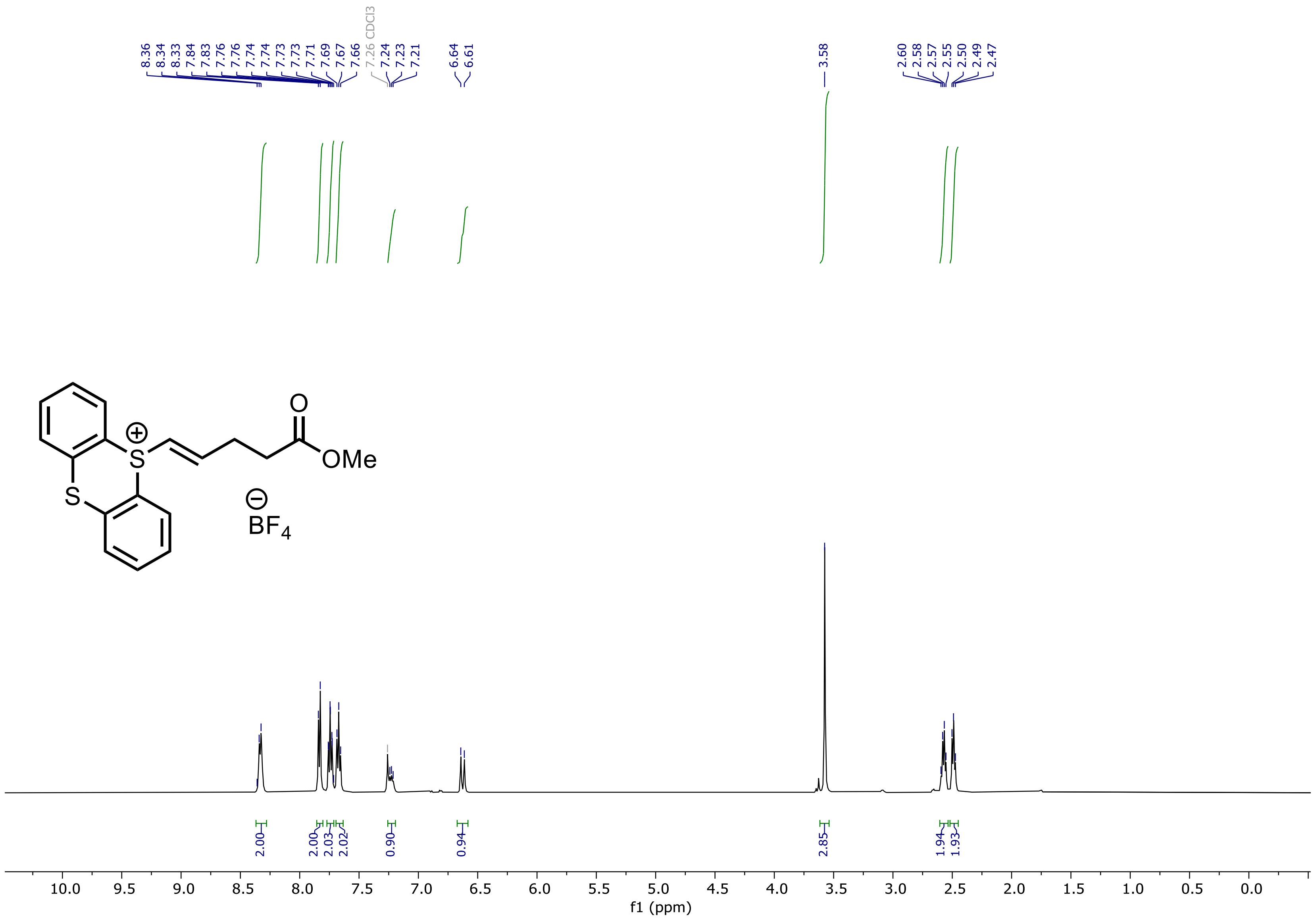

^{13}C NMR of methyl pent-4-enoate derived thianthrenium salt TT-1

$CDCl_3$, 23°C

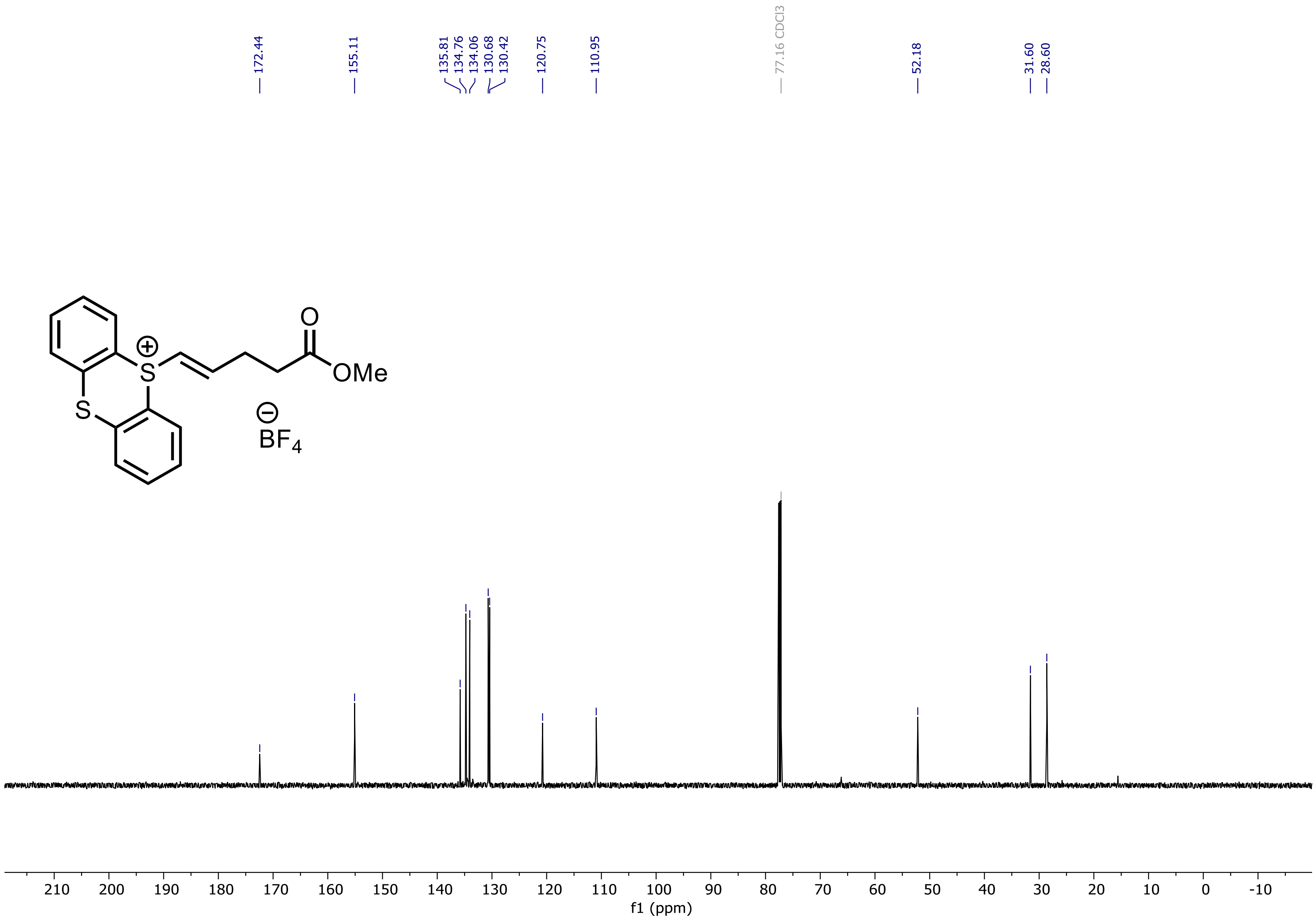

^{19}F NMR of methyl pent-4-enoate derived thianthrenium salt TT-1

$CDCl_3$, 23°C

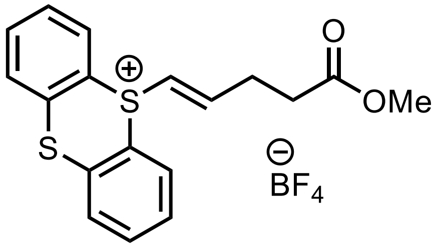

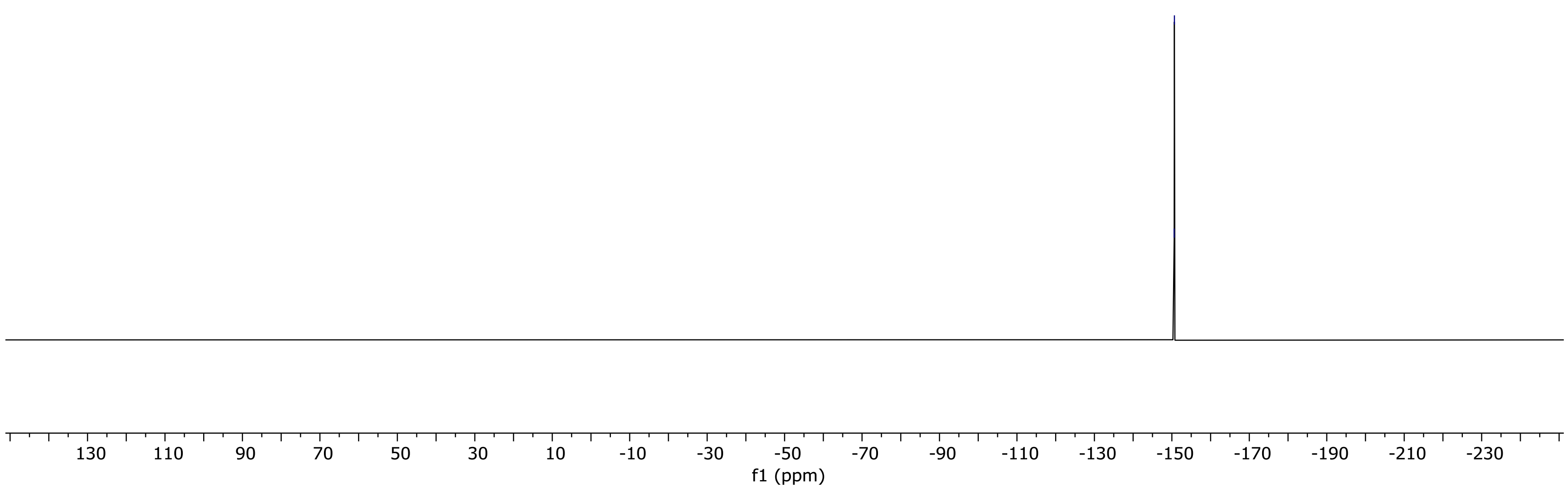

^{1}H NMR of oct-1-en-7-yne derived thianthrenium salt TT-4

CD_3CN, 23°C

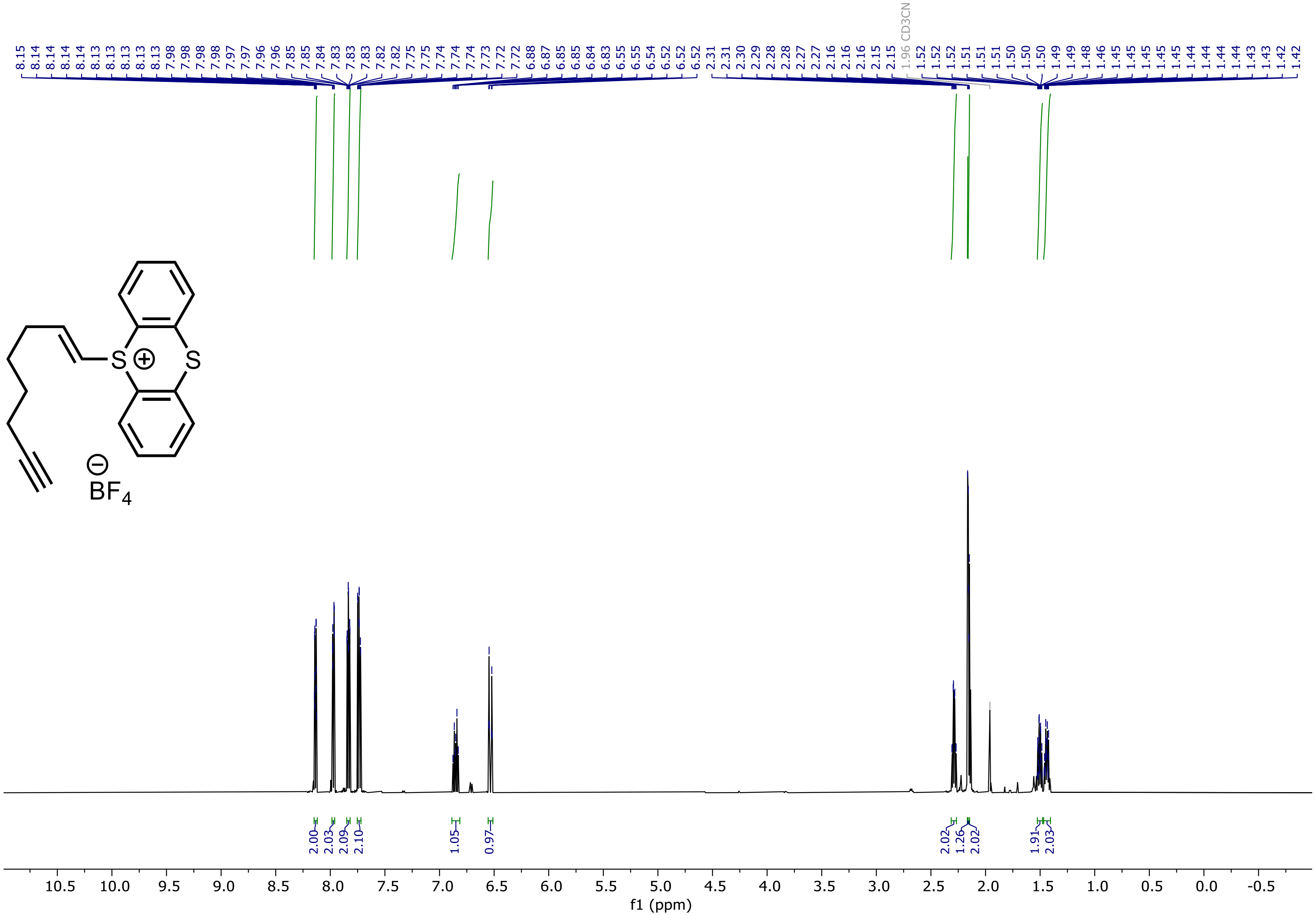

^{13}C NMR of oct-1-en-7-yne derived thianthrenium salt TT-4

CD_3CN, 23°C

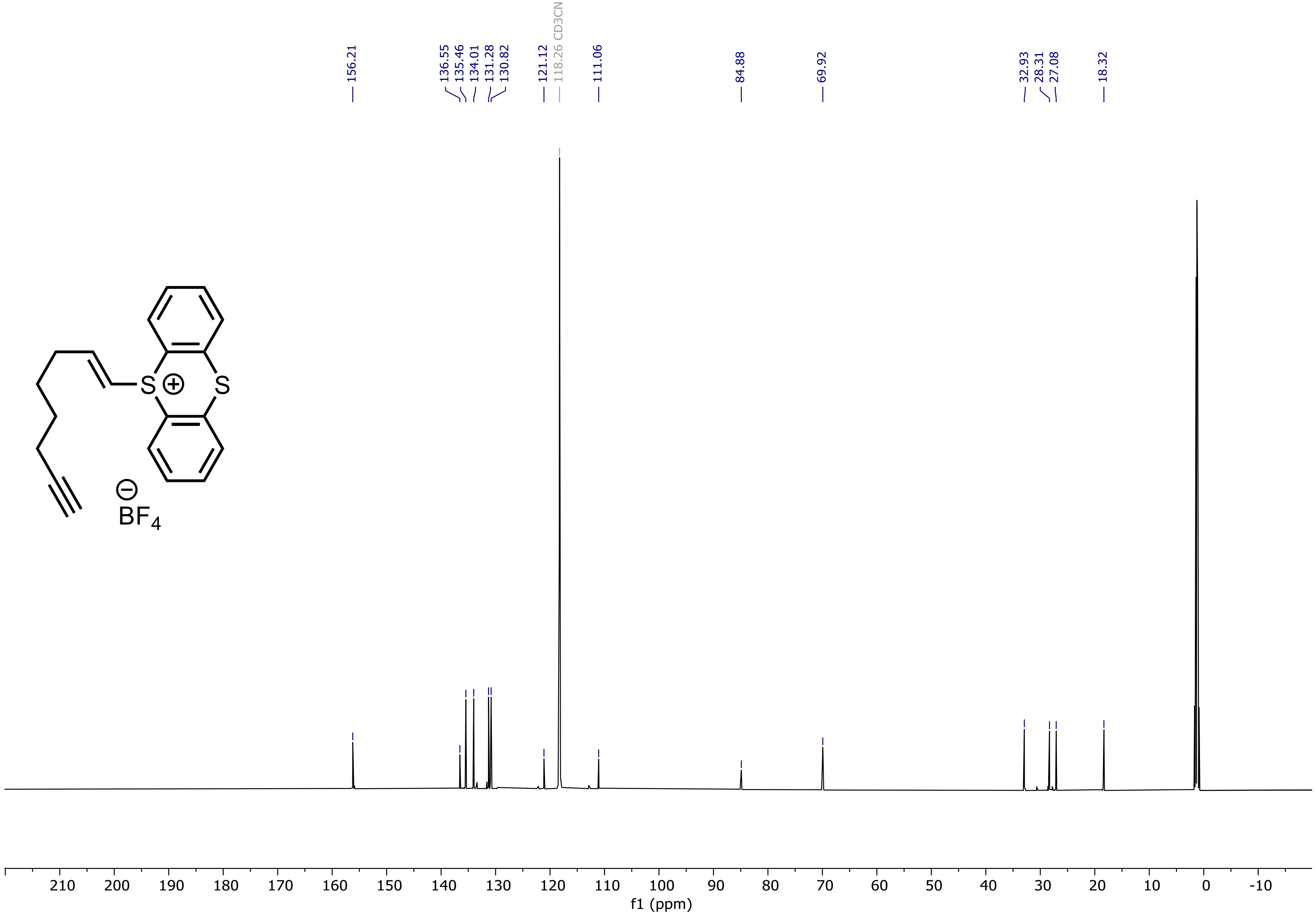

^{19}F NMR of oct-1-en-7-yne derived thianthrenium salt TT-4

CD_3CN, 23°C

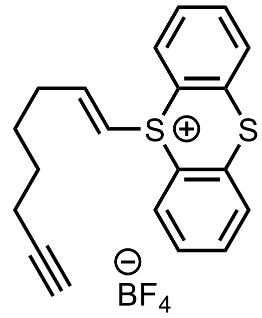

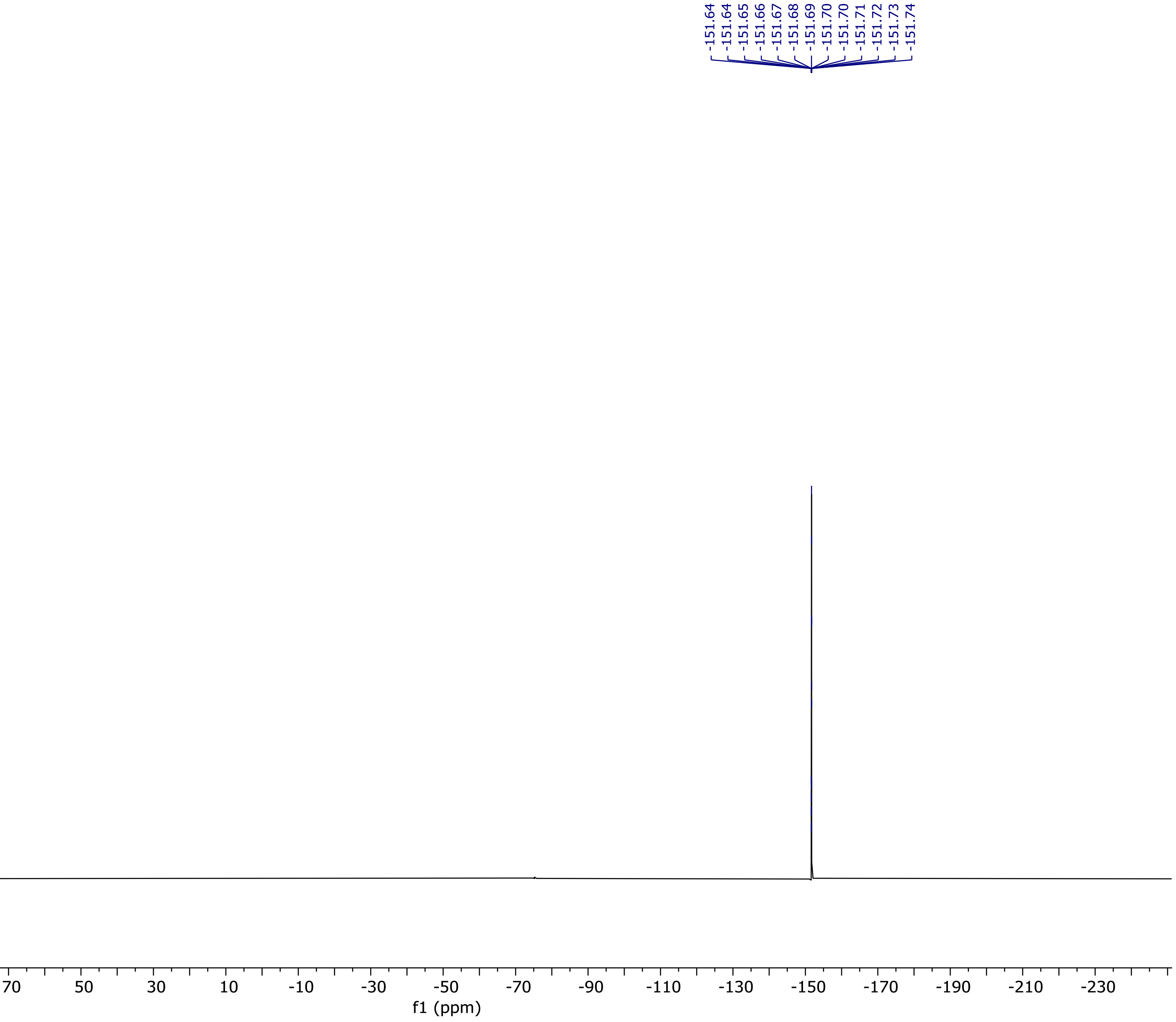

^{1}H NMR of (+)-3-carene derived thianthrenium salt TT-7

$CDCl_3$, 23°C

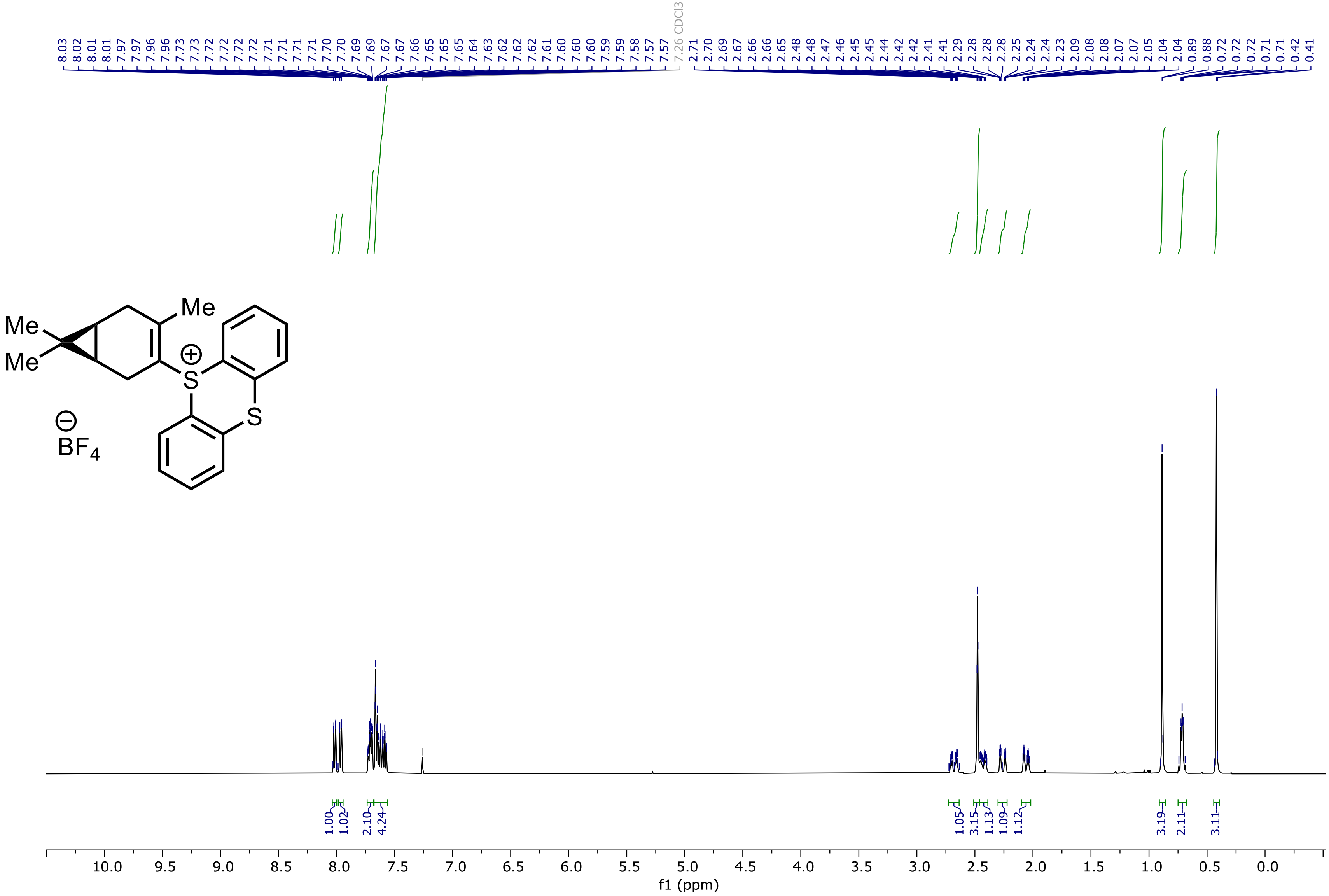

^{13}C NMR of (+)-3-carene derived thianthrenium salt TT-7

$CDCl_3$, 25°C

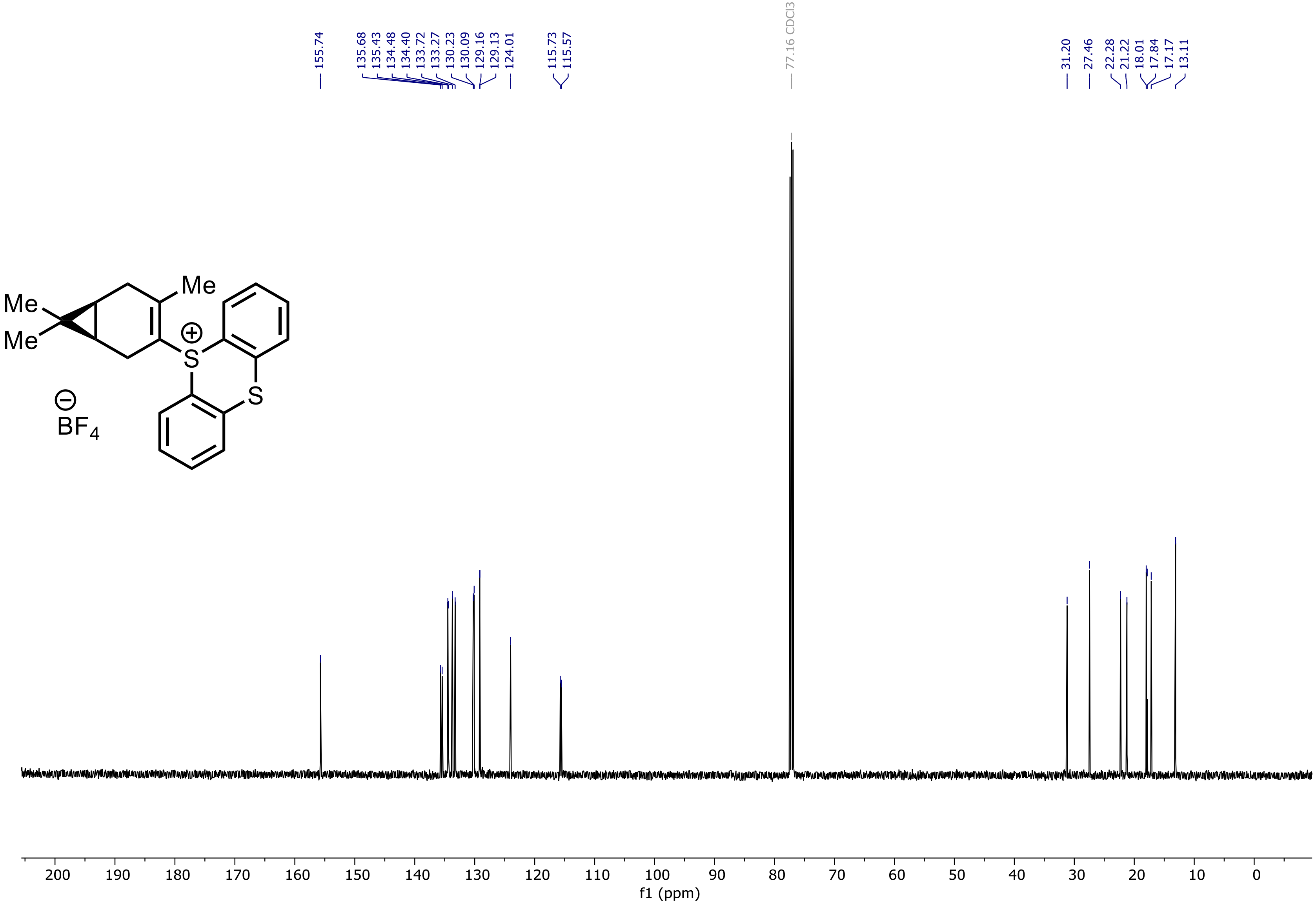

^{19}F NMR of (+)-3-carene derived thianthrenium salt TT-7

$CDCl_3$, 23°C

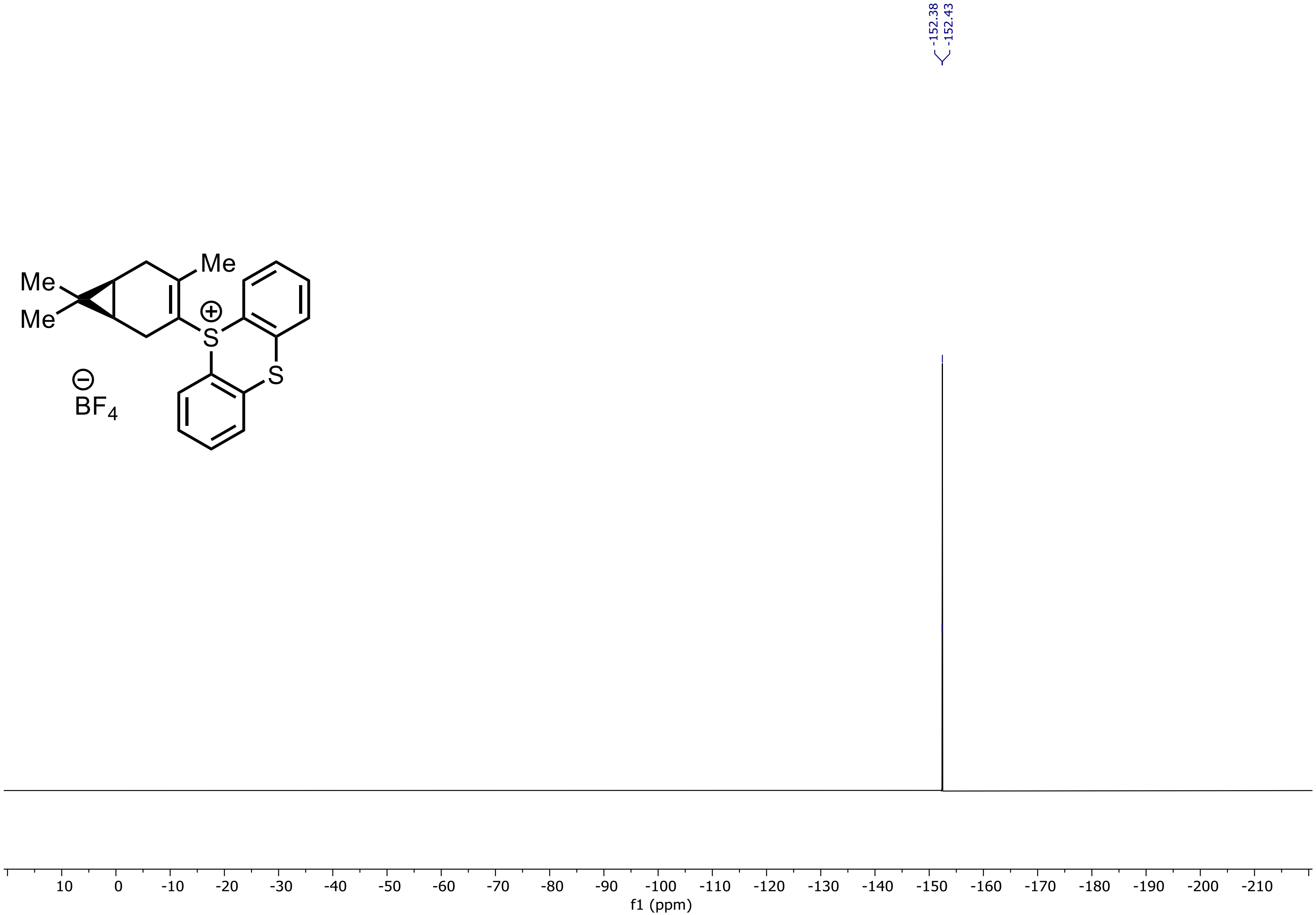

^{1}H NMR of methyl cyclopent-3-ene-1-carboxylate derived thianthrenium salt (±)-TT-11

$CDCl_3$, 23°C

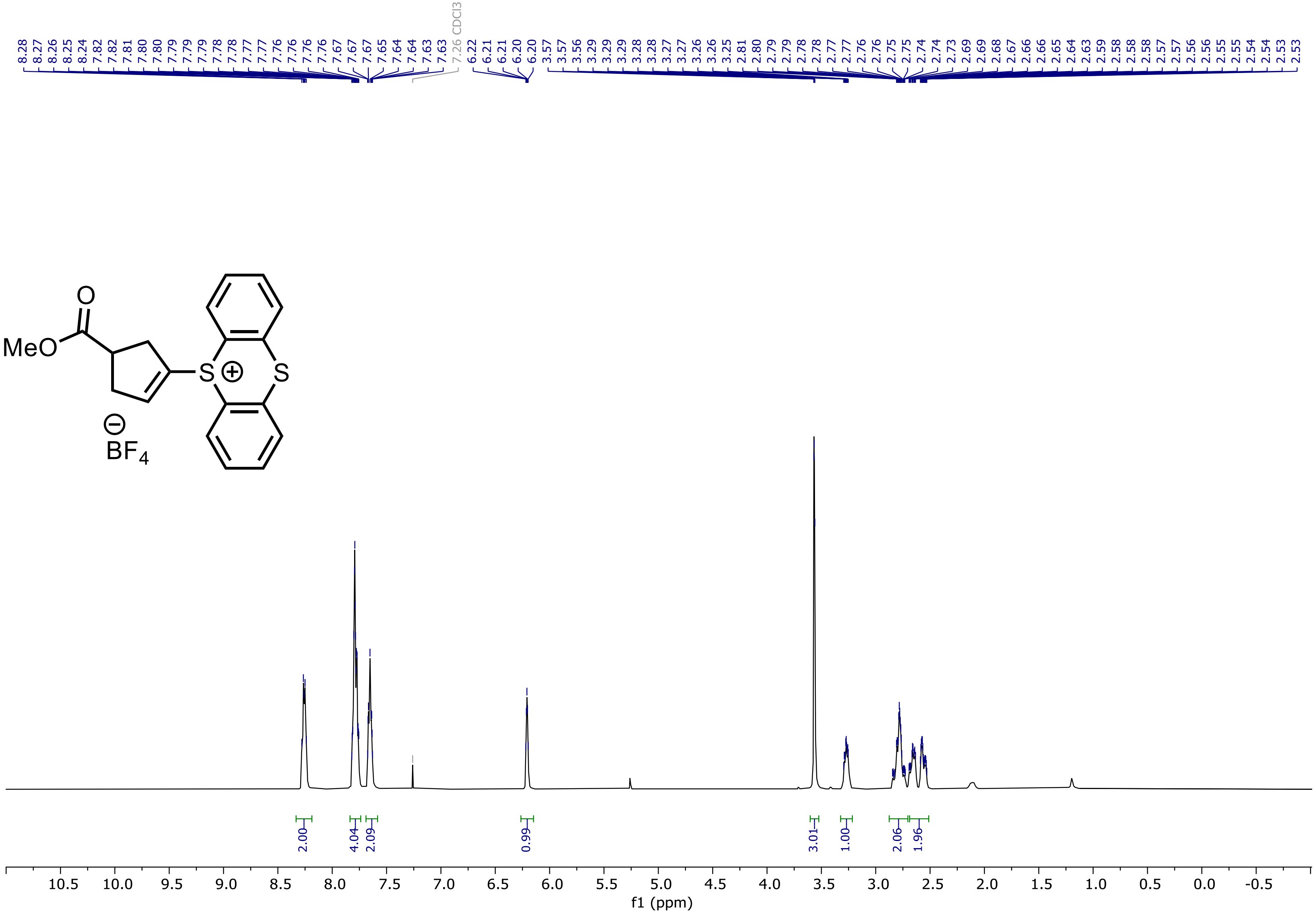

^{13}C NMR of methyl cyclopent-3-ene-1-carboxylate derived thianthrenium salt (±)-TT-11

$CDCl_3$, 25°C

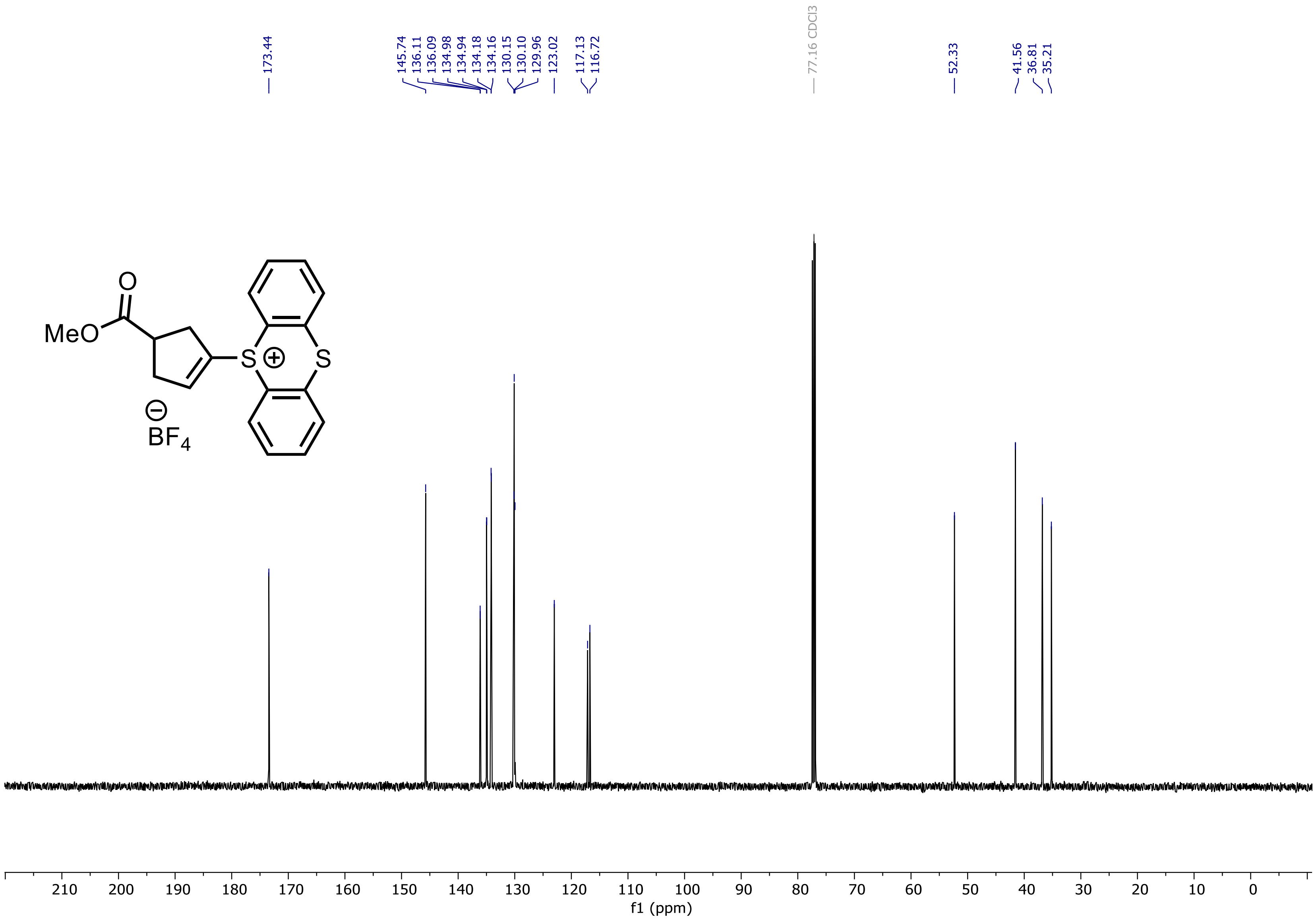

^{19}F NMR of methyl cyclopent-3-ene-1-carboxylate derived thianthrenium salt (±)-TT-11

$CDCl_3$, 23°C

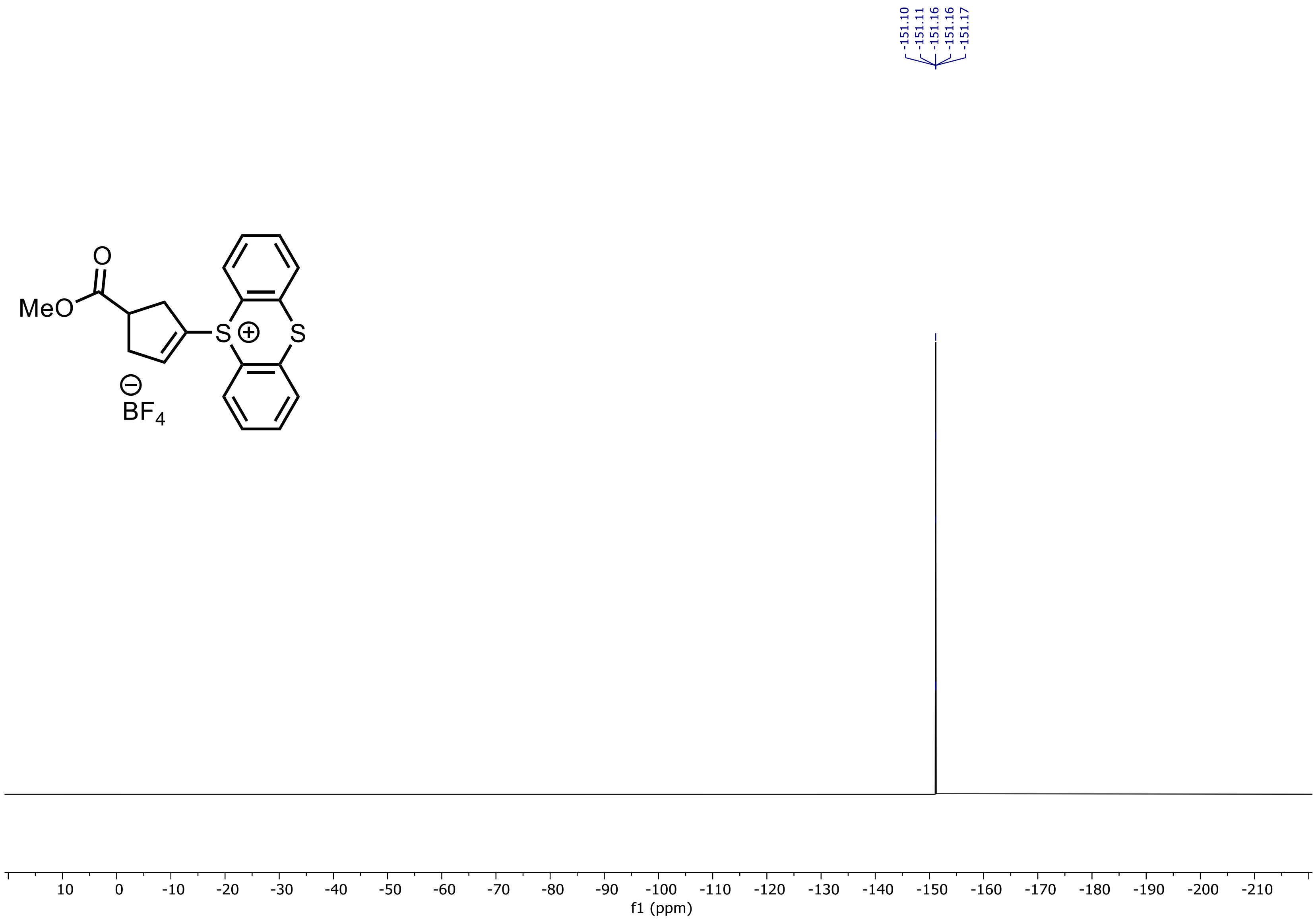

^{1}H NMR of 1-methylcyclopent-1-ene derived thianthrenium salt TT-15

$CDCl_3$, 23°C

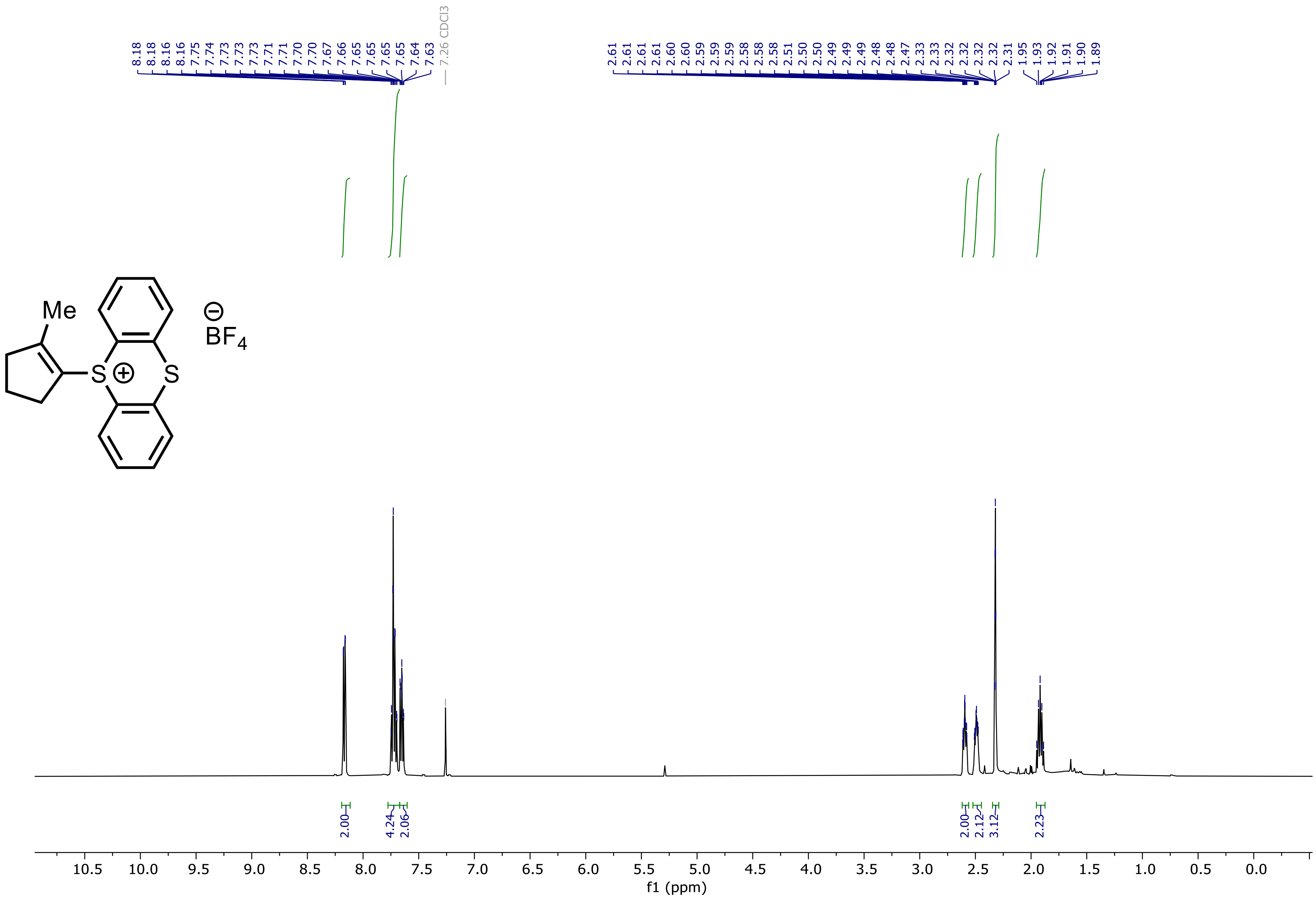

^{13}C NMR of 1-methylcyclopent-1-ene derived thianthrenium salt TT-15

$CDCl_3$, 25°C

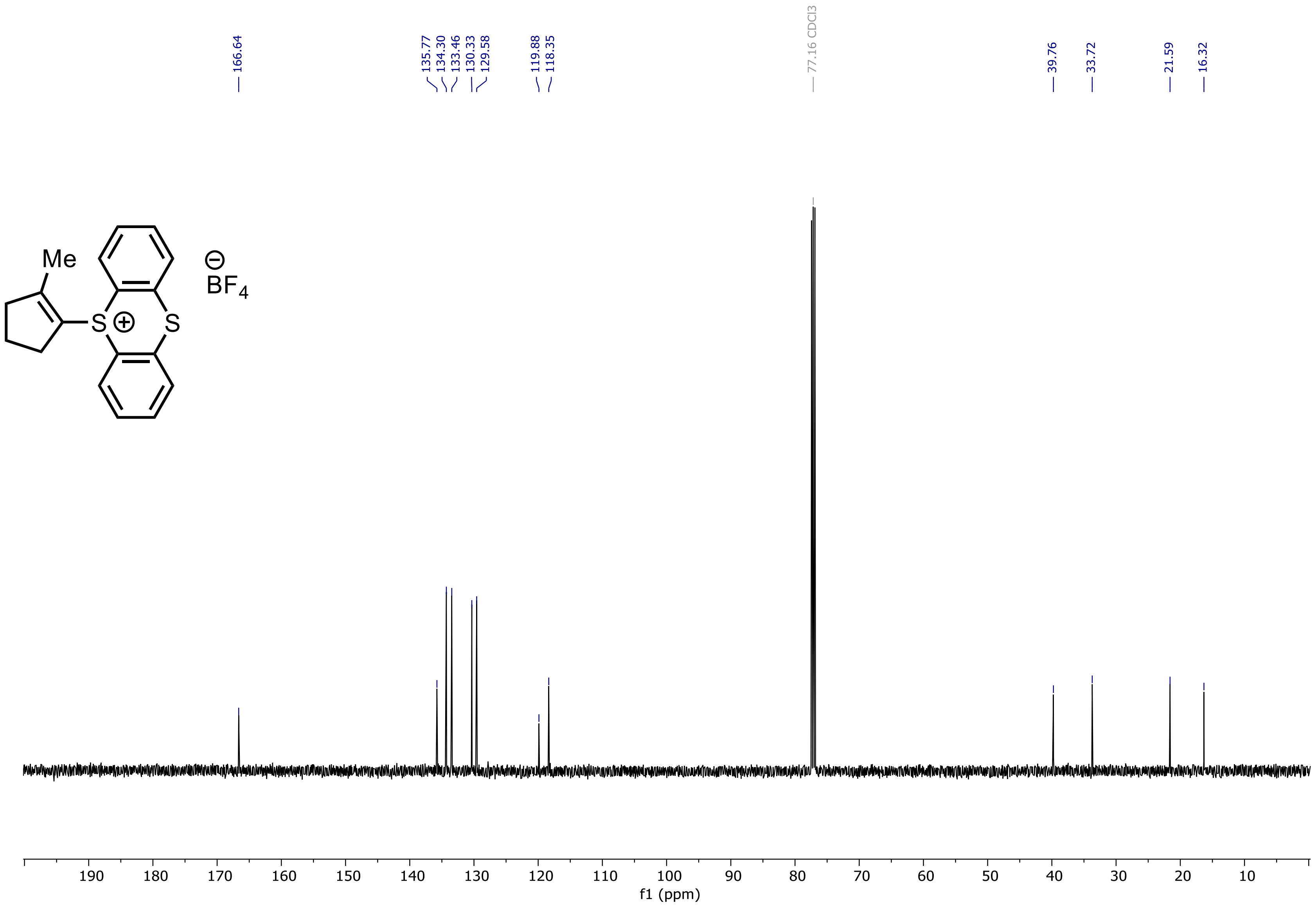

^{19}F NMR of 1-methylcyclopent-1-ene derived thianthrenium salt TT-15

$CDCl_3$, 23°C

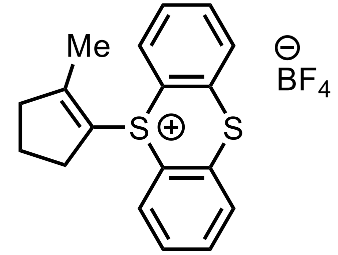

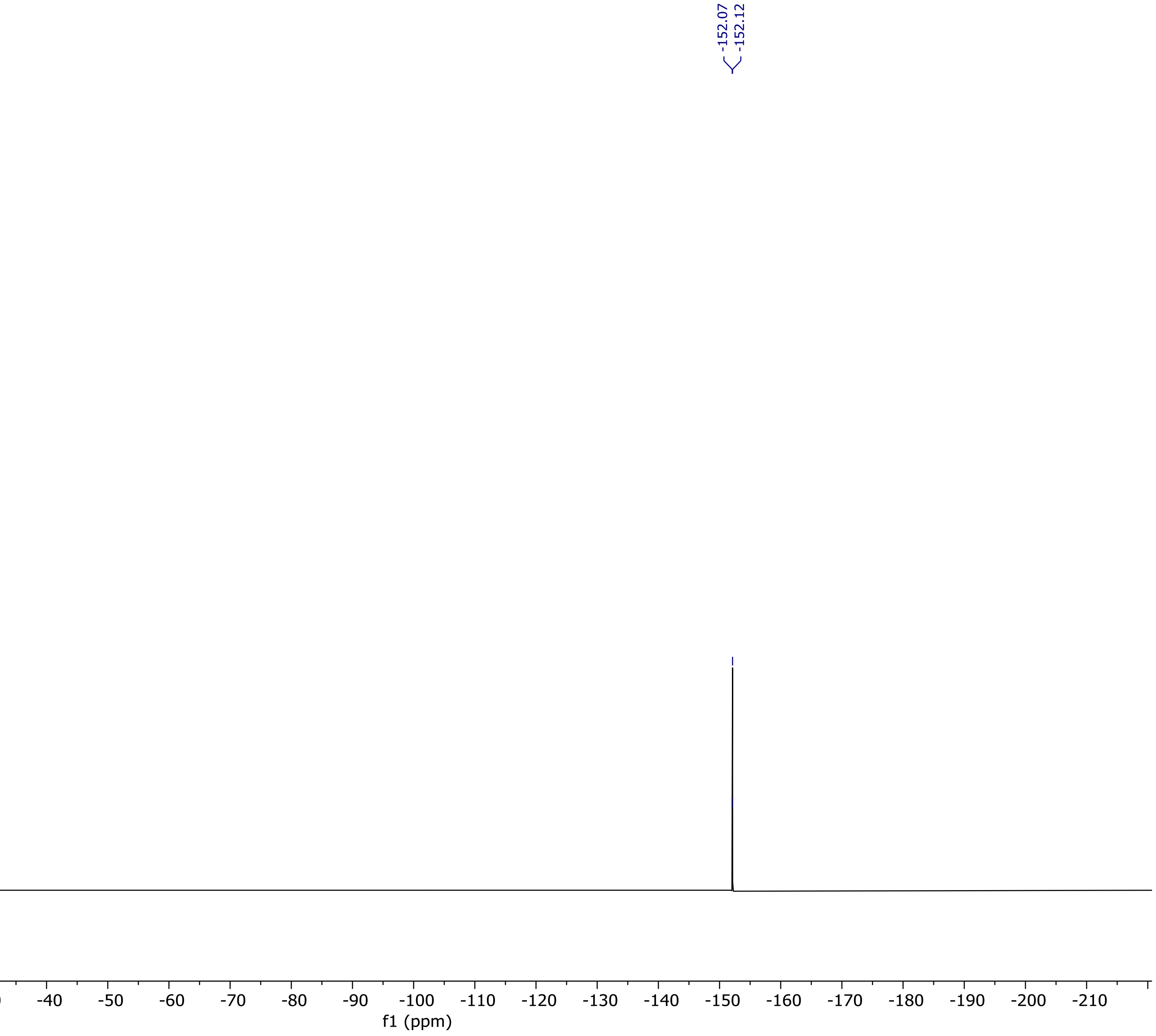

^{1}H NMR of (–)-*cis*-rose-oxide derived thianthrenium salt TT-16

$CDCl_3$, 23°C

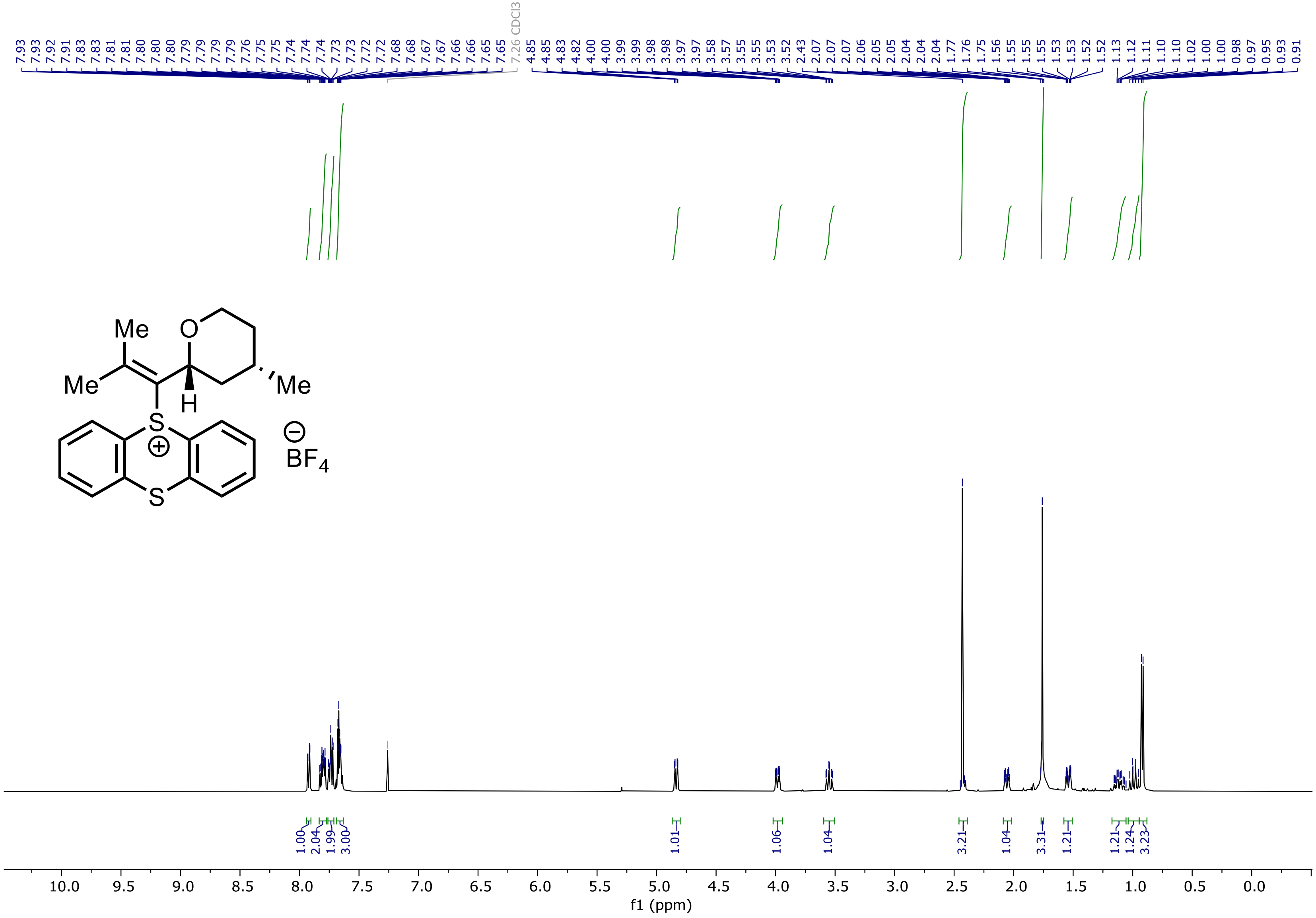

^{13}C NMR of (–)-*cis*-rose-oxide derived thianthrenium salt TT-16

$CDCl_3$, 23°C

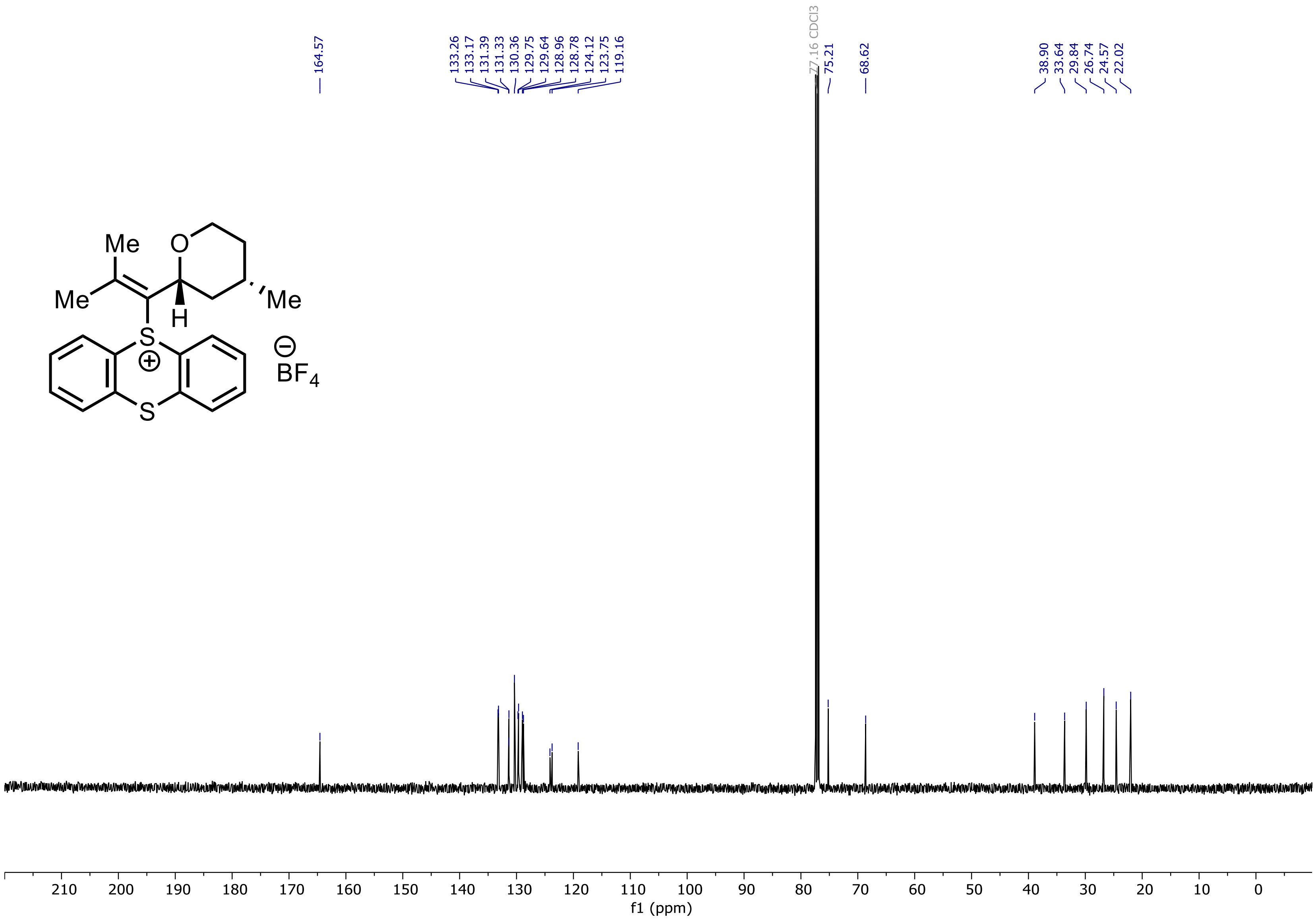

^{19}F NMR of (–)-*cis*-rose-oxide derived thianthrenium salt TT-16

$CDCl_3$, 23°C

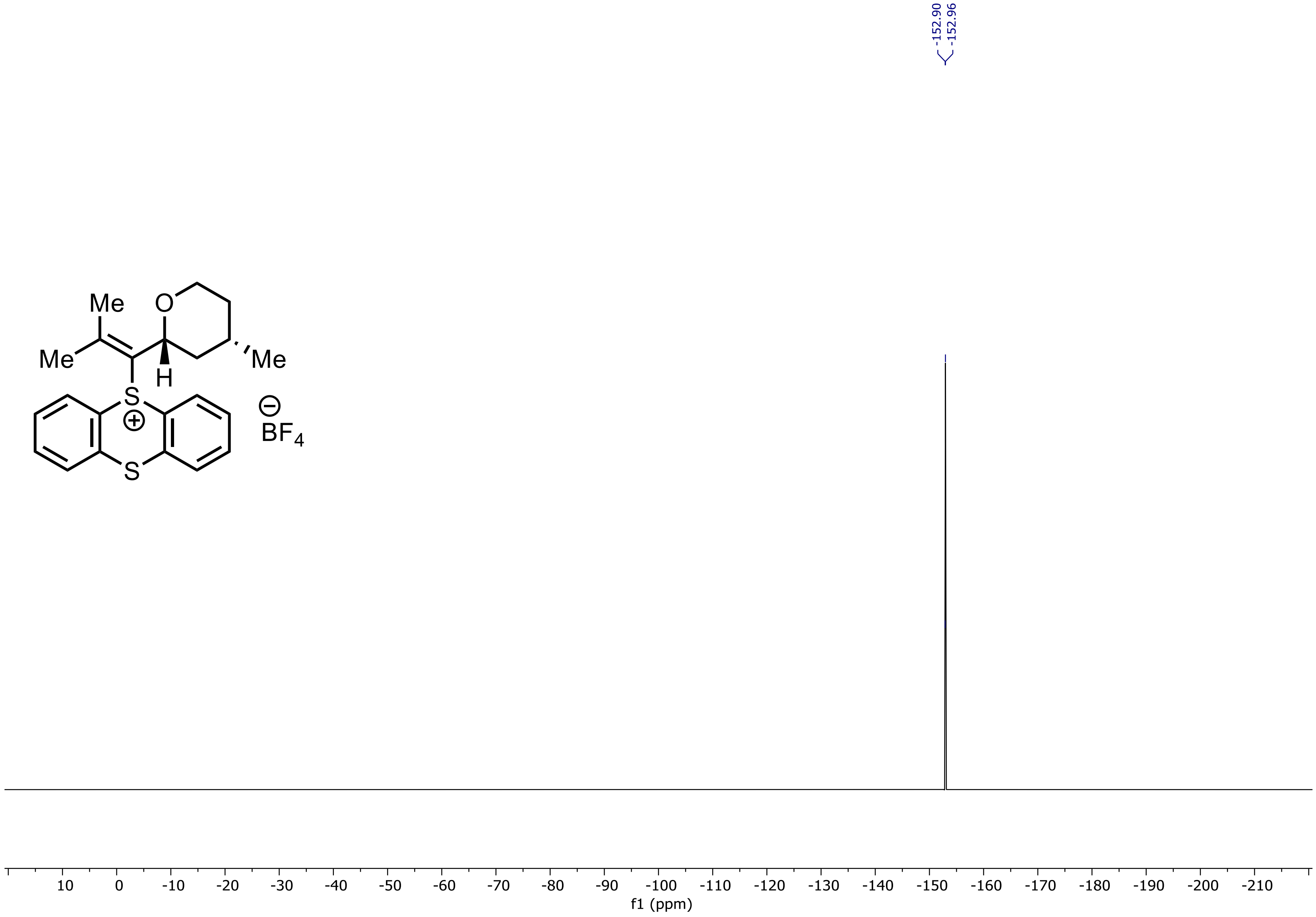

^{1}H NMR of (*S*)-(+)-carvone derived thianthrenium salt TT-17

$CDCl_3$, 23°C

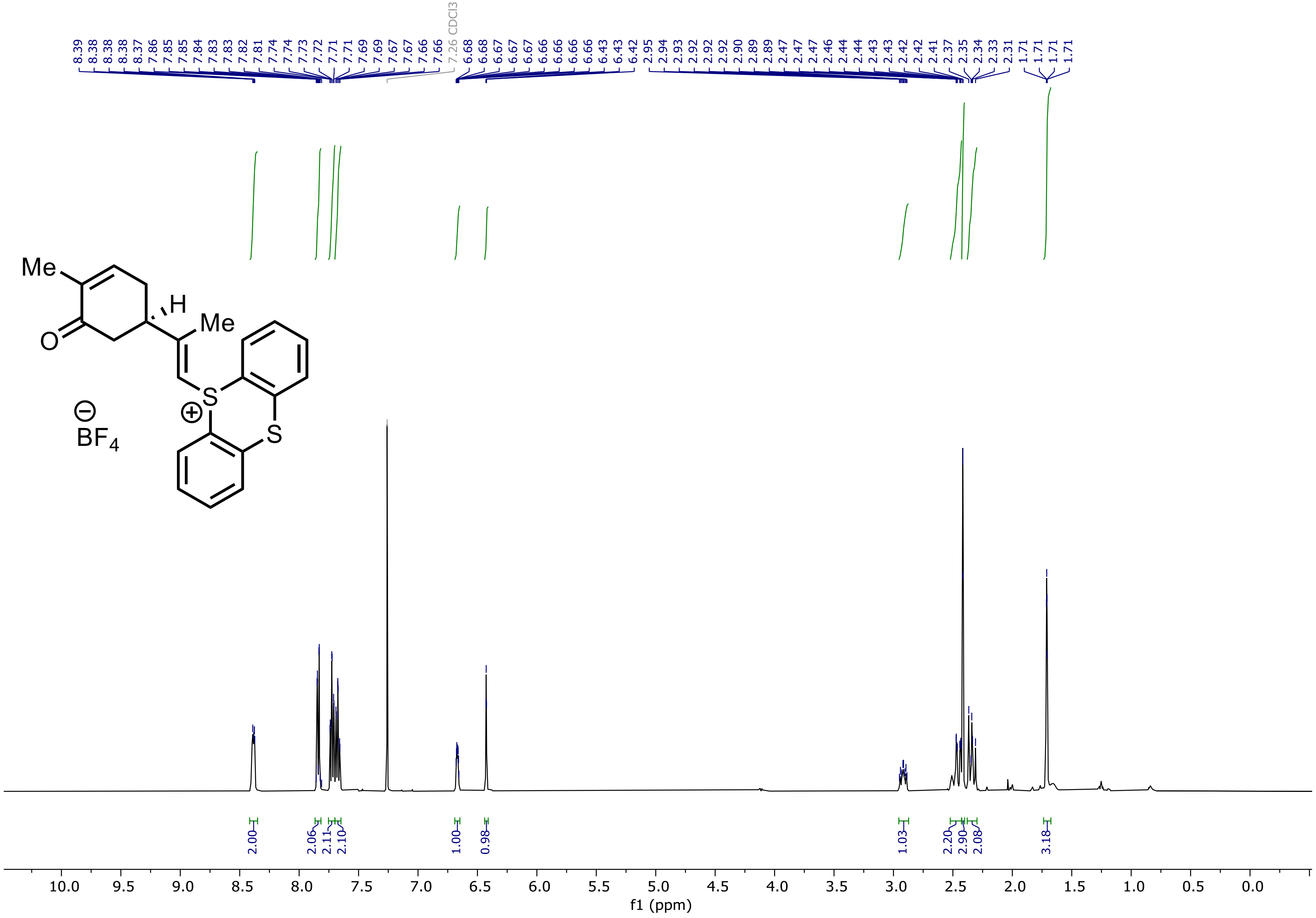

^{13}C NMR of (*S*)-(+)-carvone derived thianthrenium salt TT-17

$CDCl_3$, 23°C

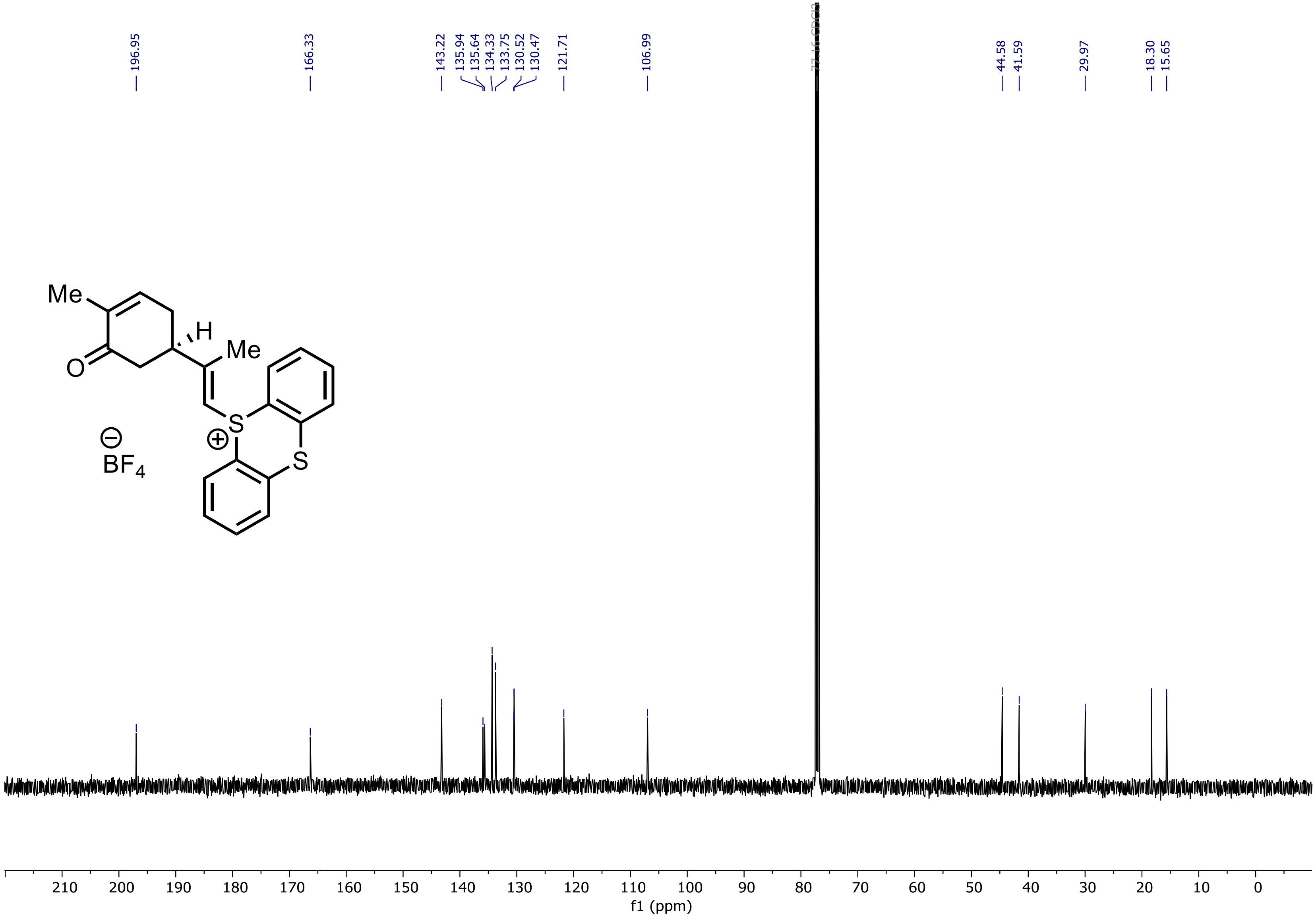

^{19}F NMR of (*S*)-(+)-carvone derived thianthrenium salt TT-17

$CDCl_3$, 23°C

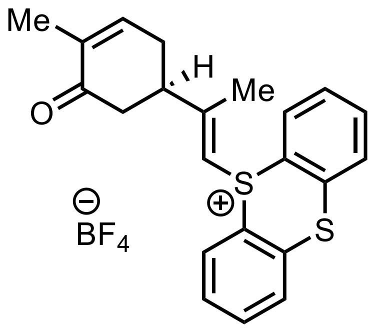

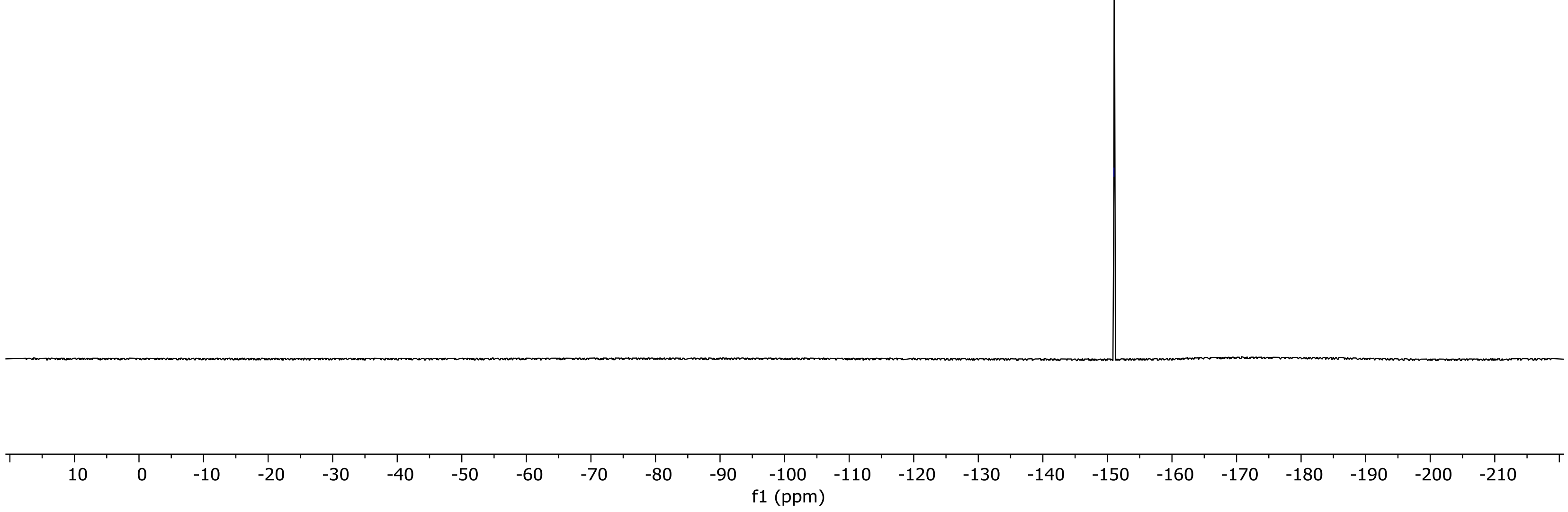

^{1}H NMR of (*S*)-(–)-*β*-citronellol derived thianthrenium salt TT-18

$CDCl_3$, 23°C

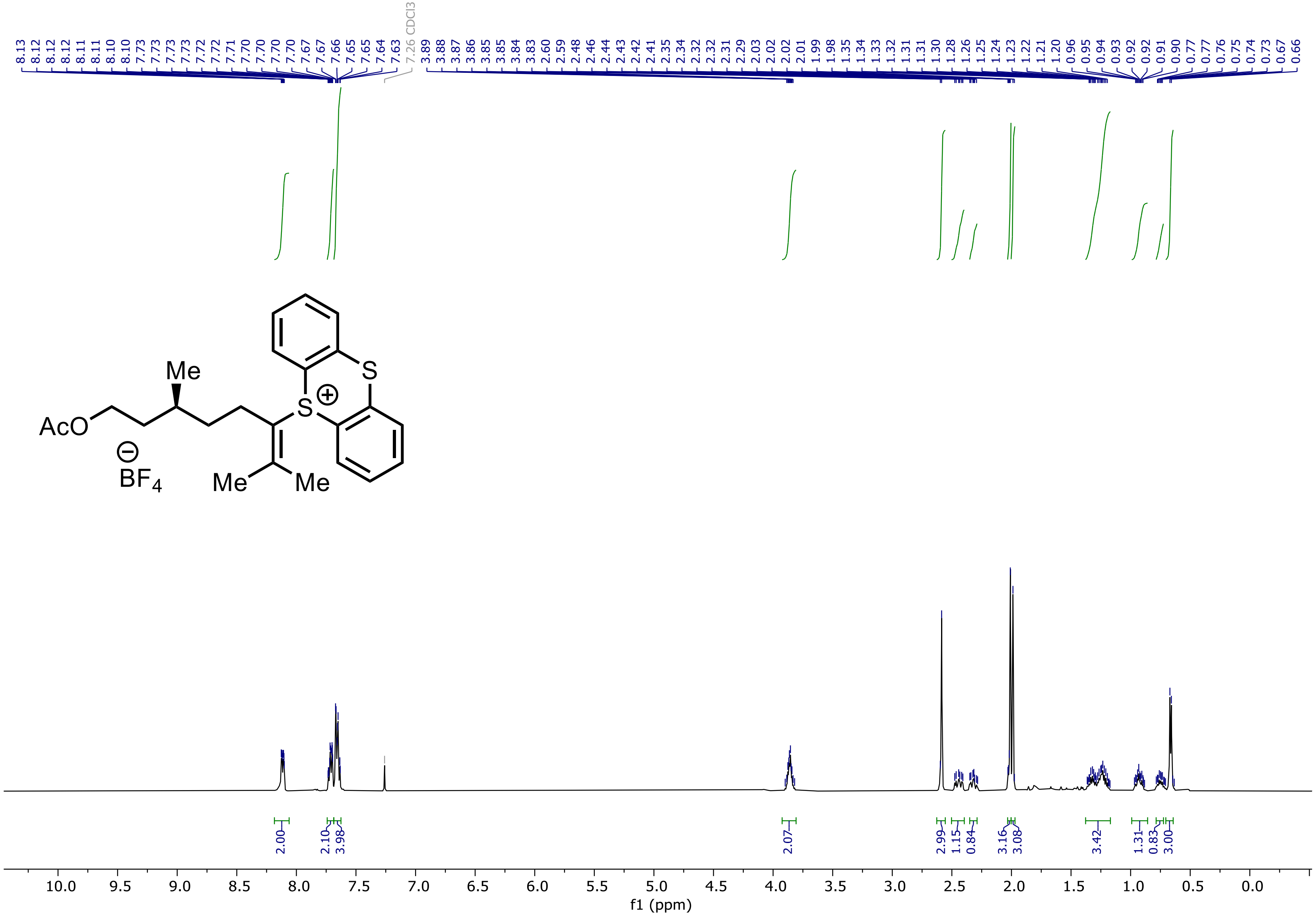

^{13}C NMR of (*S*)-(–)-*β*-citronellol derived thianthrenium salt TT-18

$CDCl_3$, 23°C

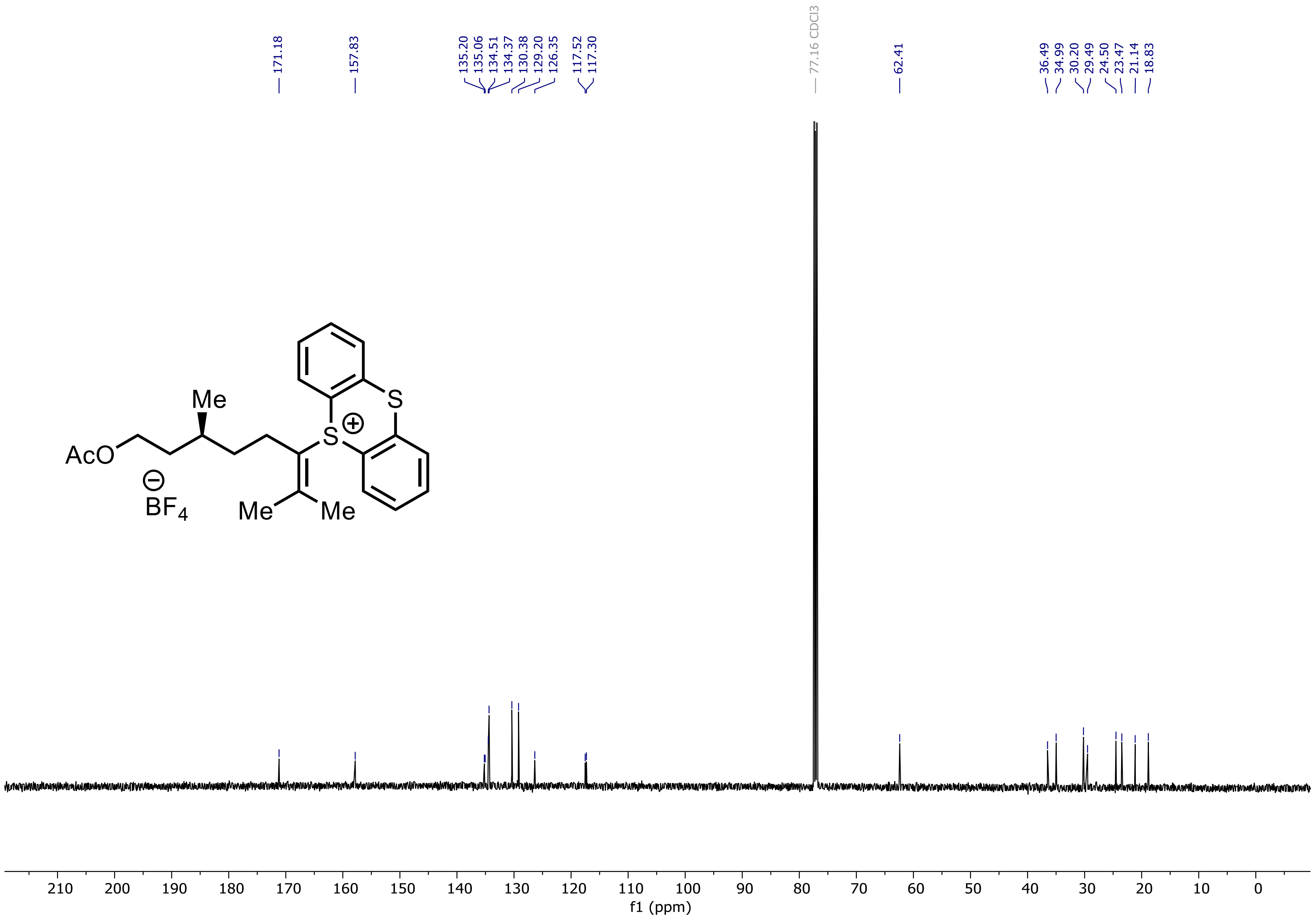

^{19}F NMR of (*S*)-(–)-*β*-citronellol derived thianthrenium salt TT-18

CDCl$_3$, 23°C

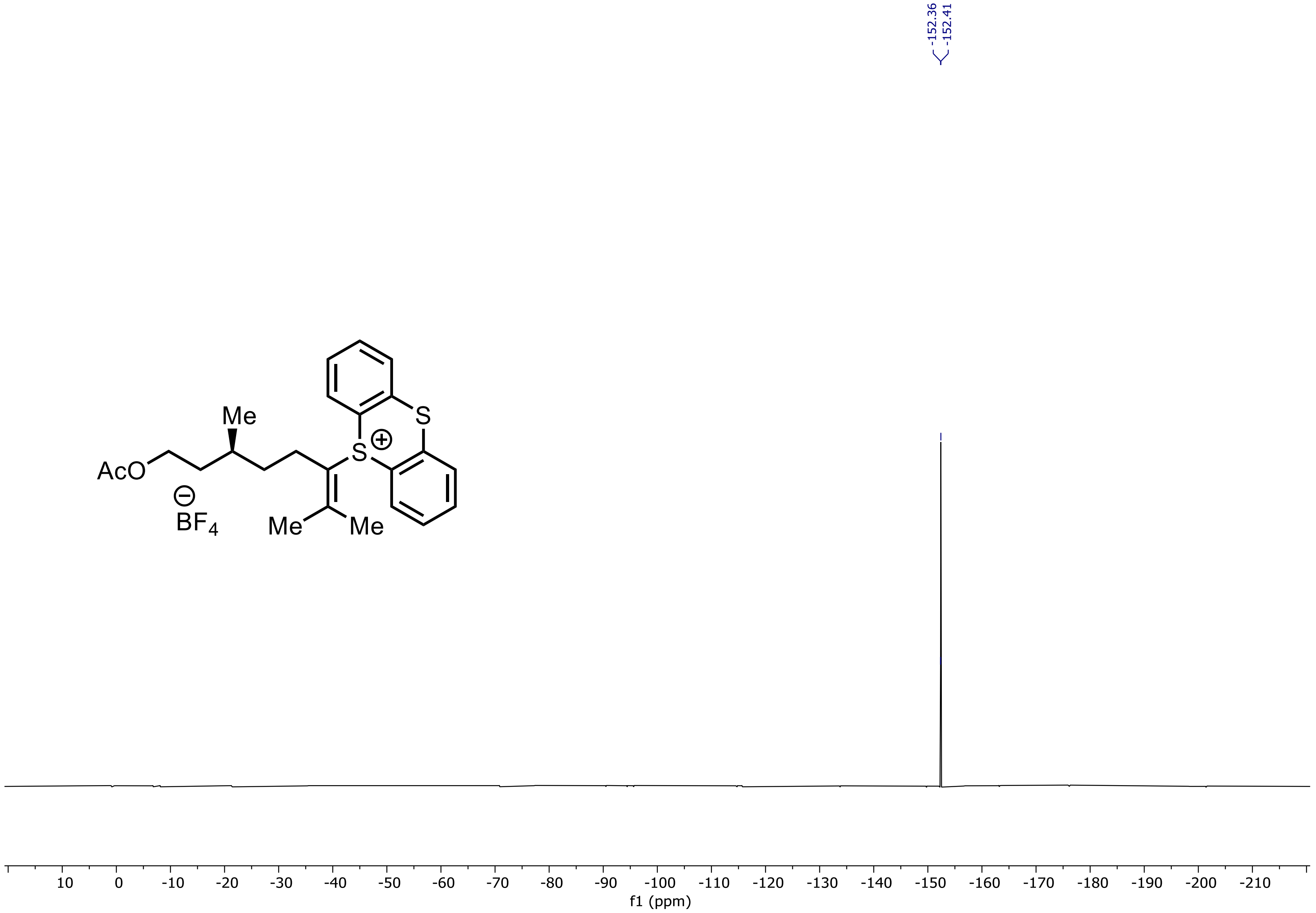

^{1}H NMR of (*S*)-(–)-perillaldehyde derived thianthrenium salt TT-19

$CDCl_3$, 23°C

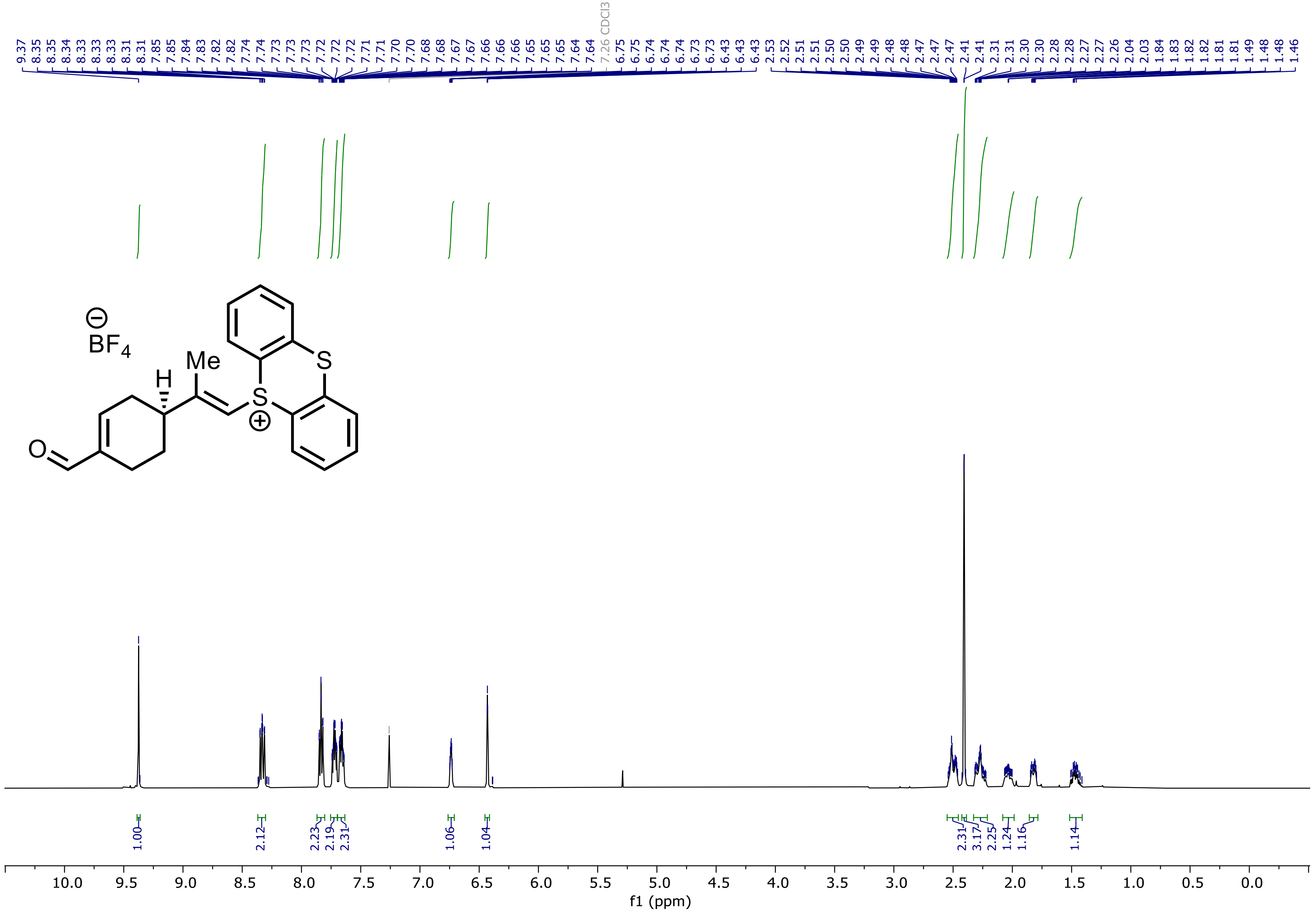

^{13}C NMR of (*S*)-(–)-perillaldehyde derived thianthrenium salt TT-19

CDCl$_3$, 23°C

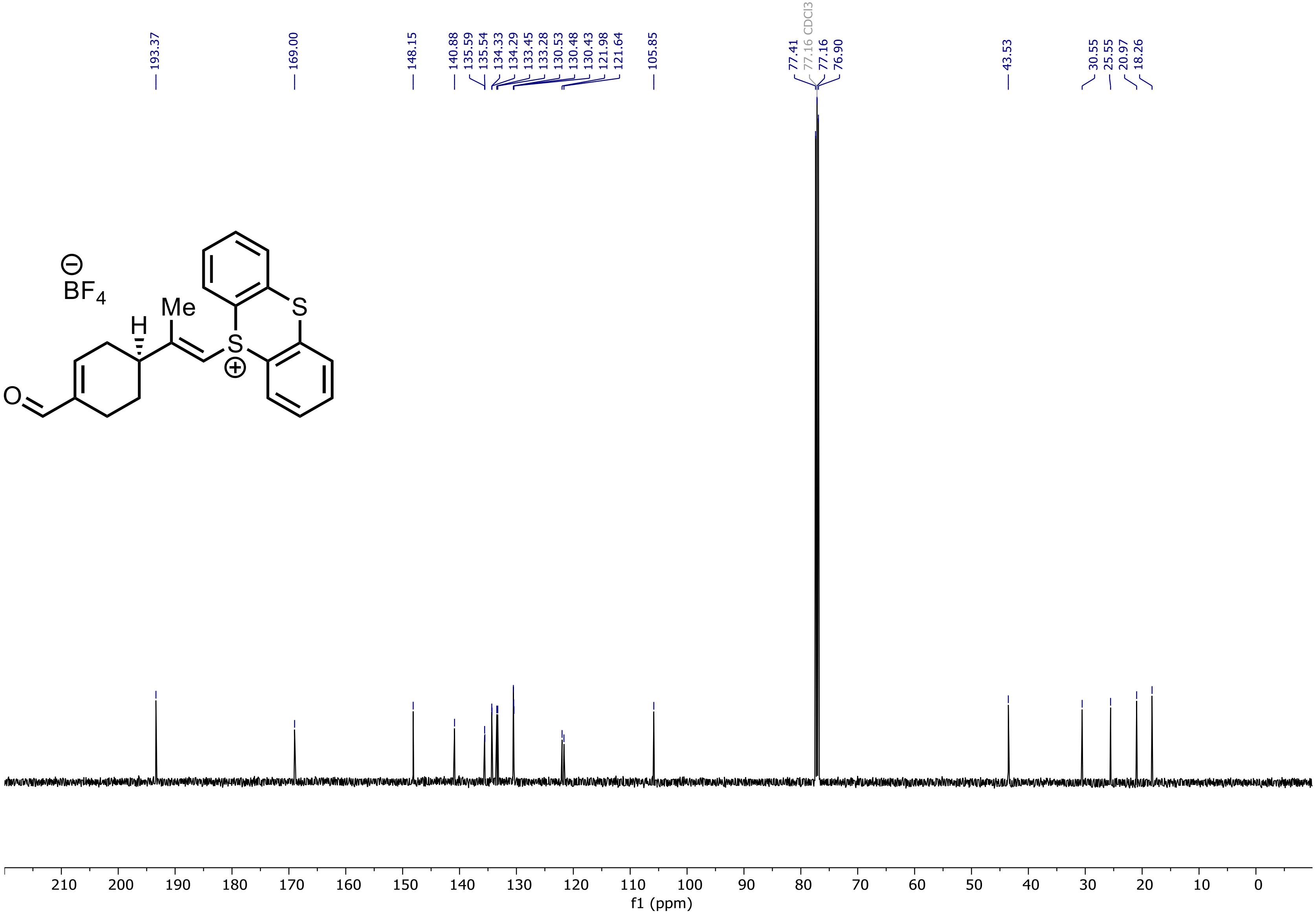

^{19}F NMR of (*S*)-(–)-perillaldehyde derived thianthrenium salt TT-19

$CDCl_3$, 23°C

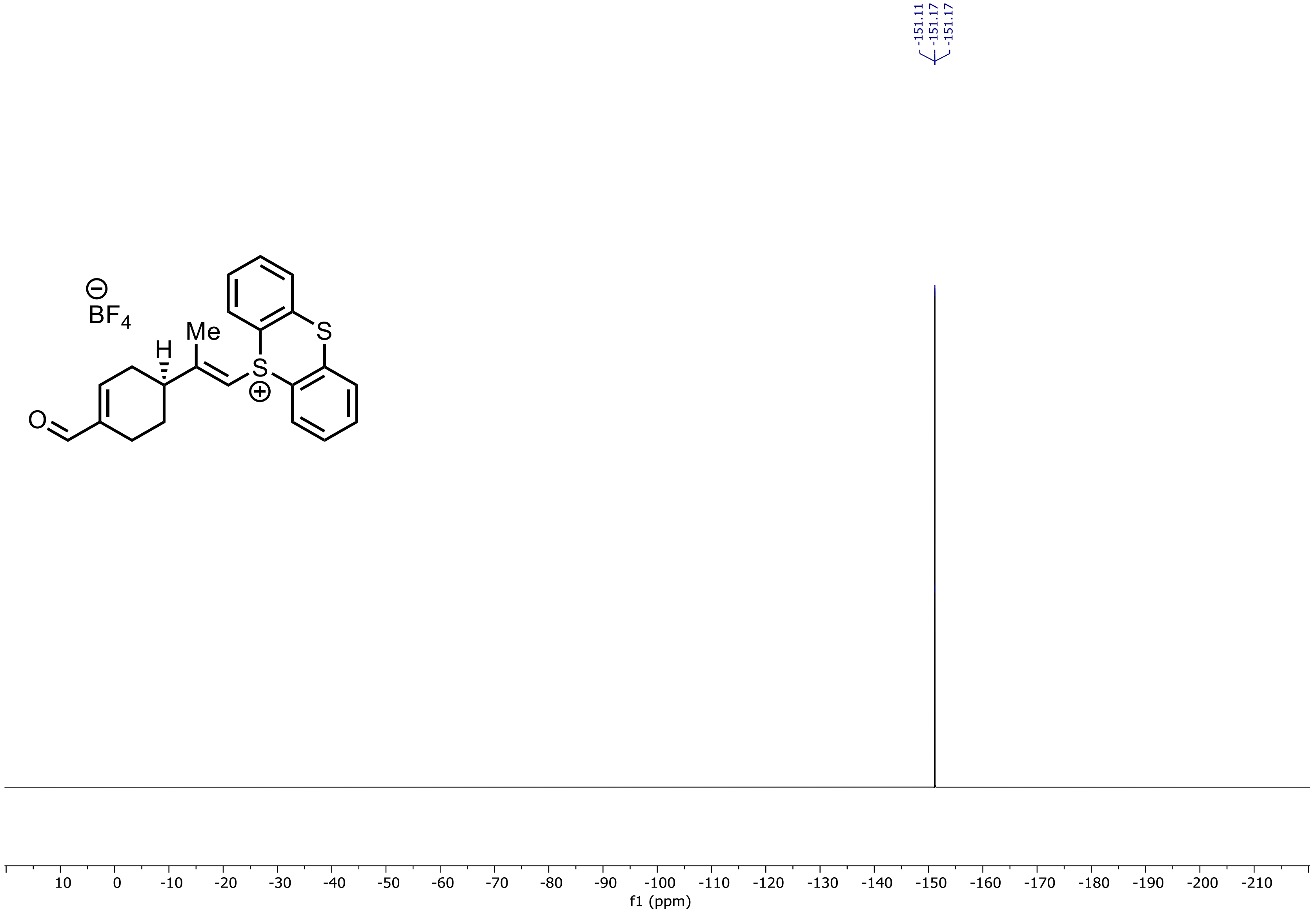

^{1}H NMR of citral derived thianthrenium salt TT-20

CD_3CN, 23°C

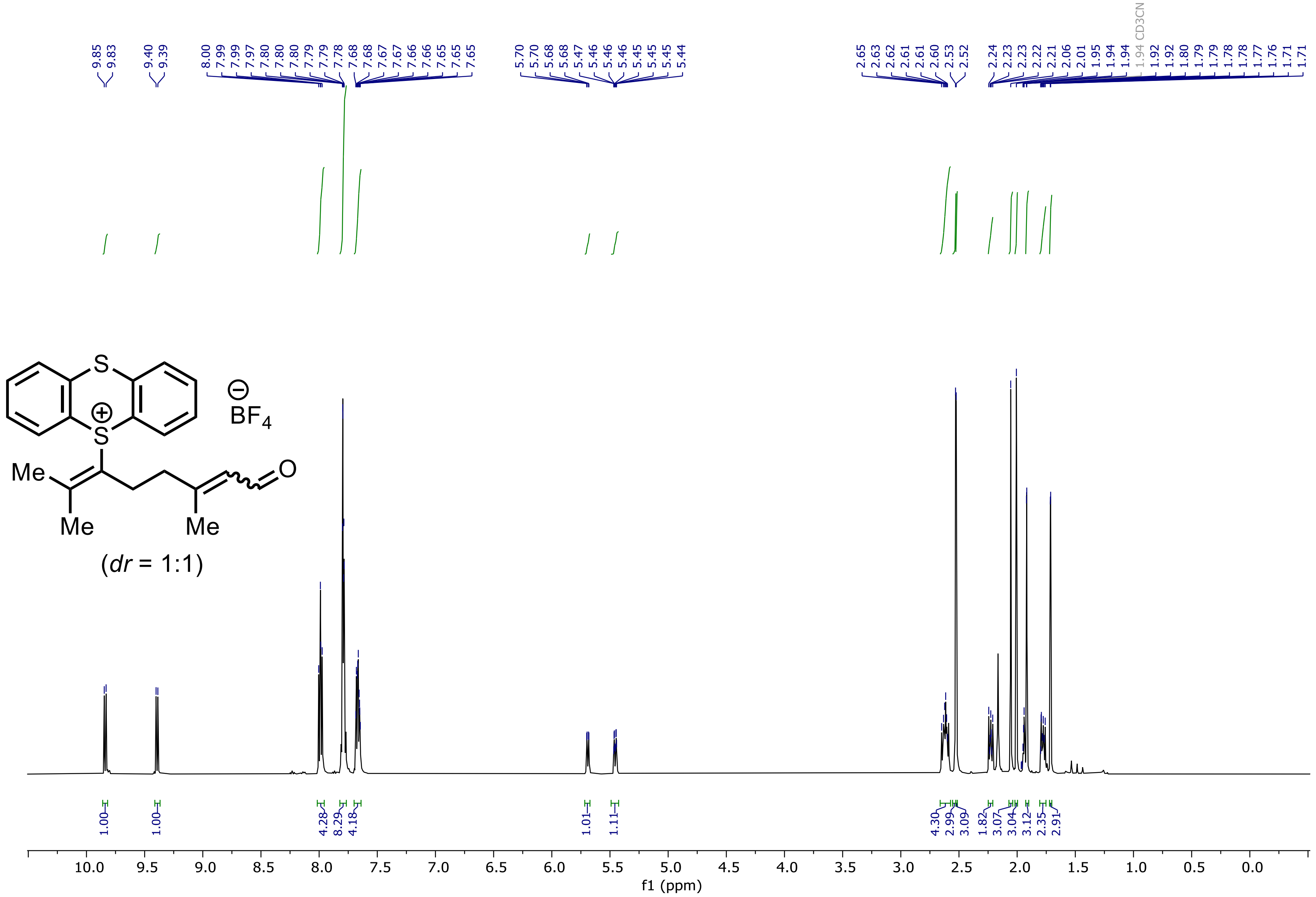

^{13}C NMR of citral derived thianthrenium salt TT-20

$CDCl_3$, 25°C

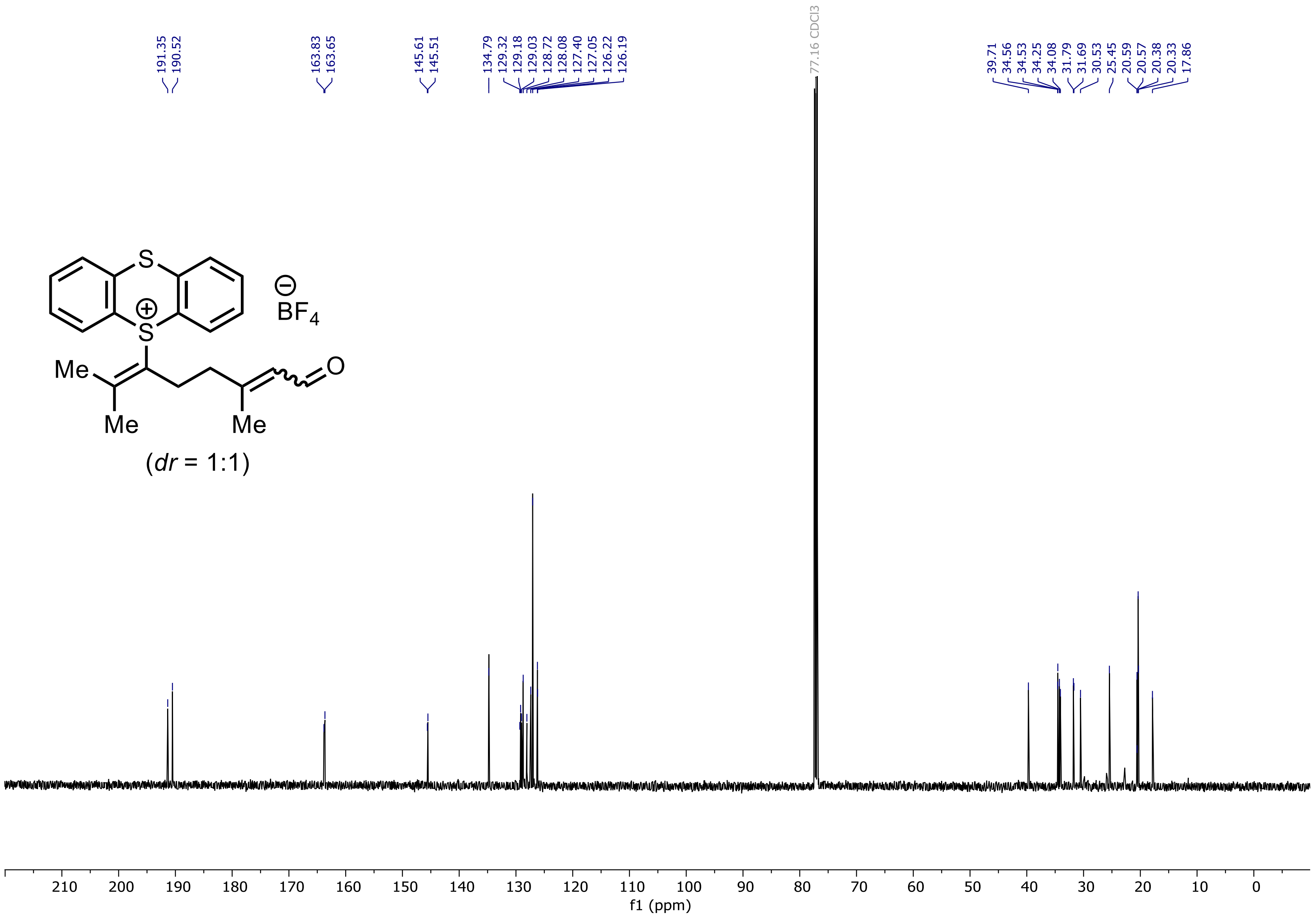

^{19}F NMR of citral derived thianthrenium salt TT-20

CD_3CN, 25°C

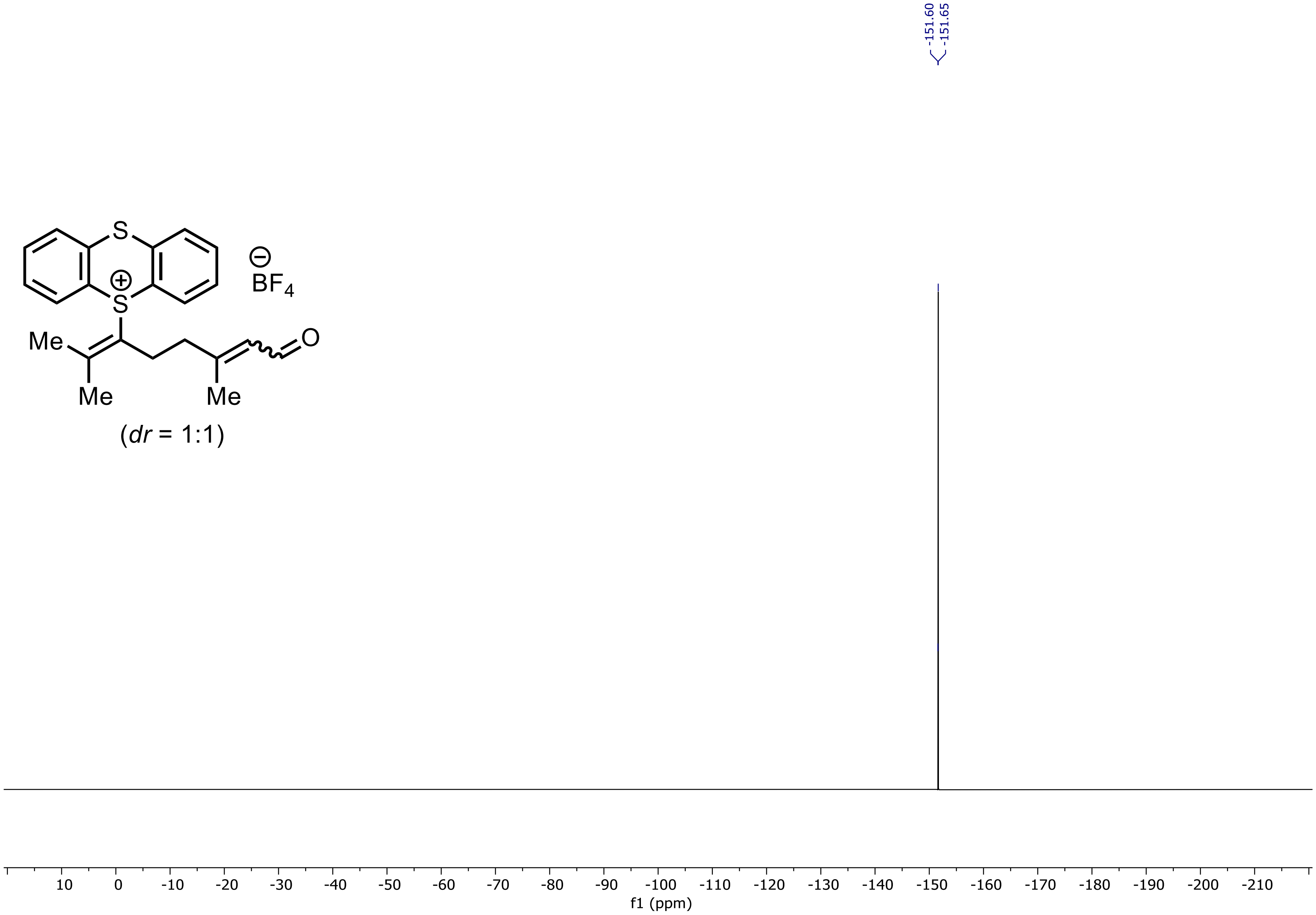

(*dr* = 1:1)

^{1}H NMR of 3-(3,5-dichlorophenyl)propanoic acid derived redox-active ester RAE-16

CD_2Cl_2, 23°C

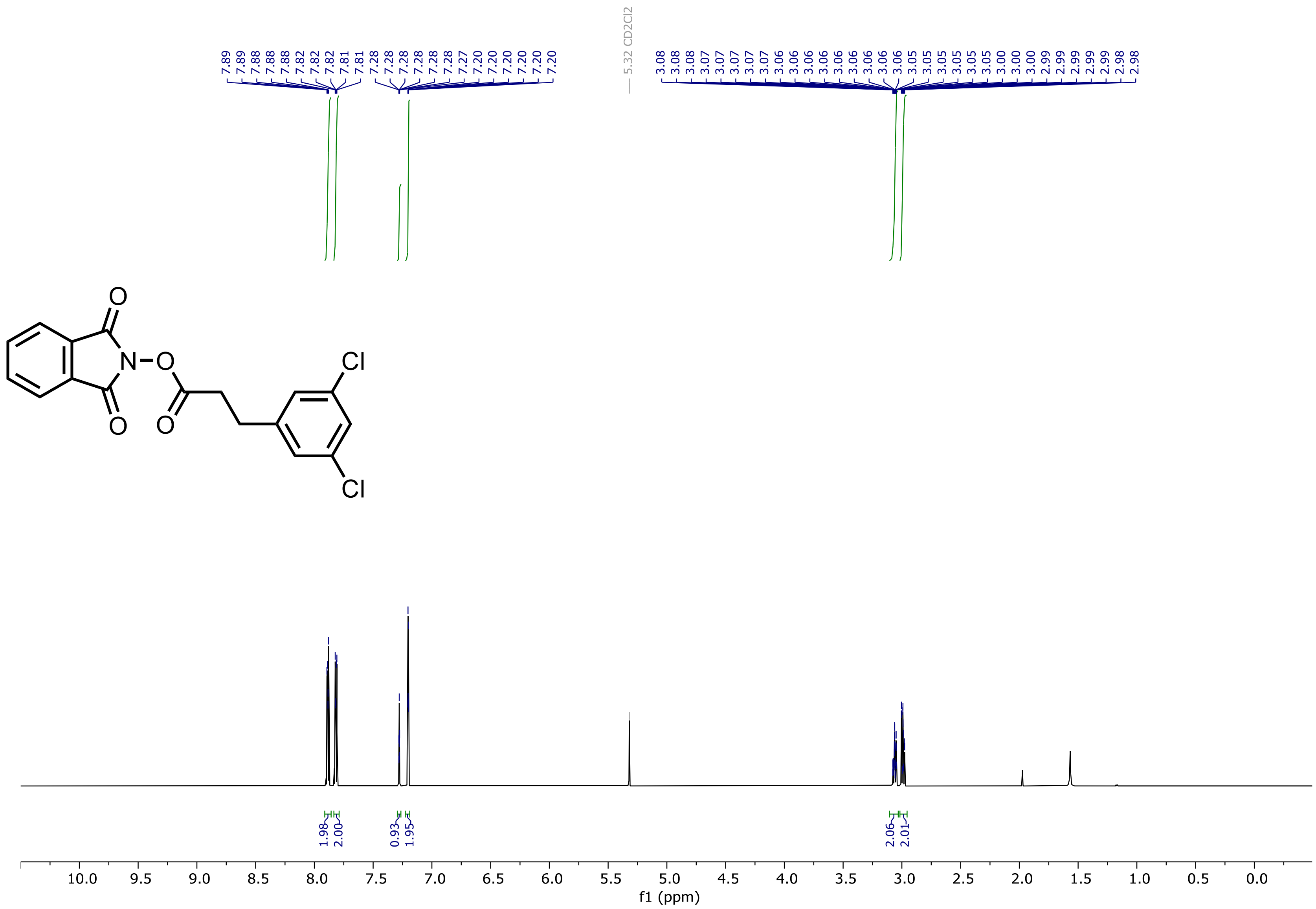

^{13}C NMR of 3-(3,5-dichlorophenyl)propanoic acid derived redox-active ester RAE-16

CD_2Cl_2, 23°C

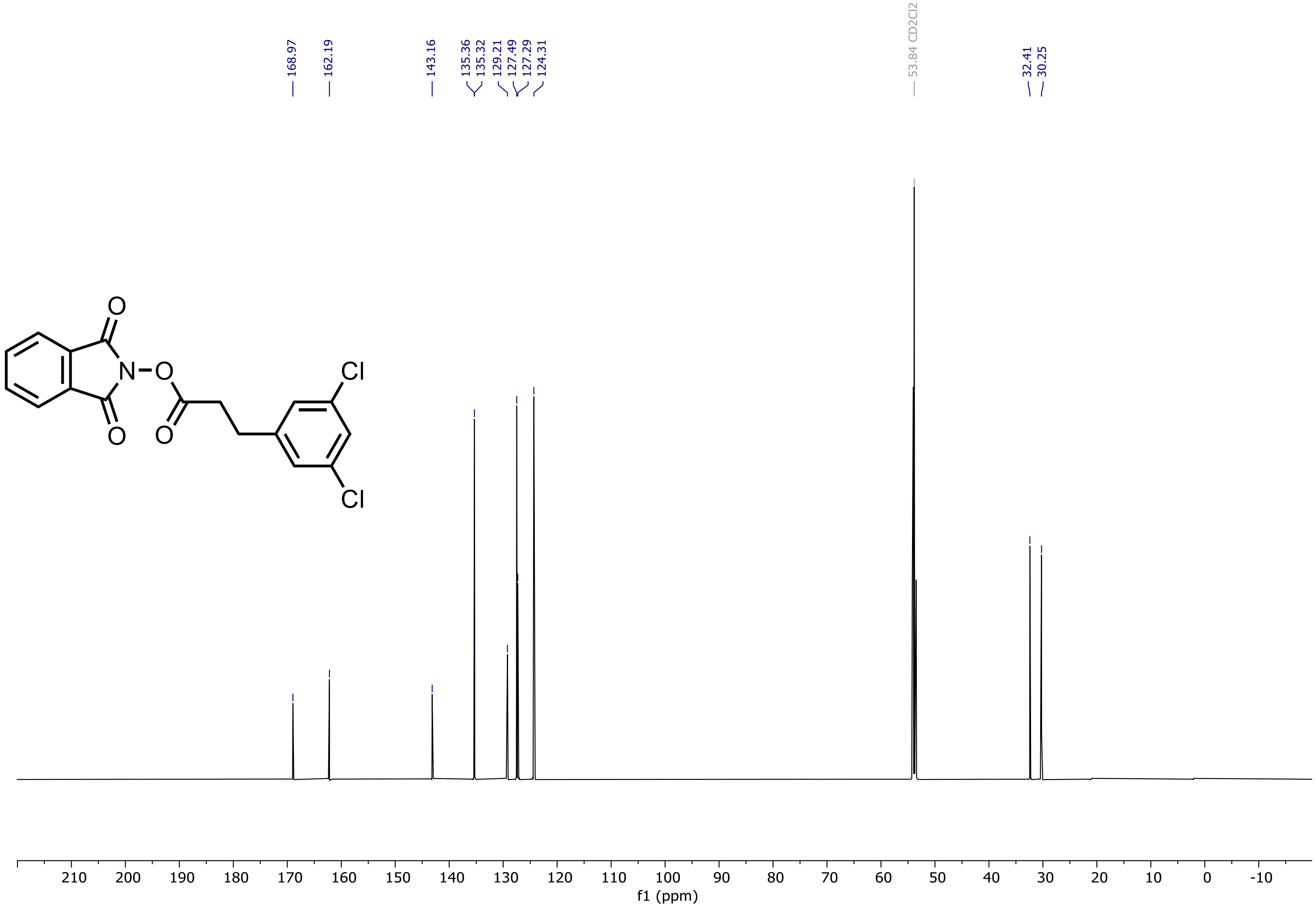

^{1}H NMR of 1-ethyl-3,5-dimethoxybenzene-derived alkylated alkene 1

$CDCl_3$, 23°C

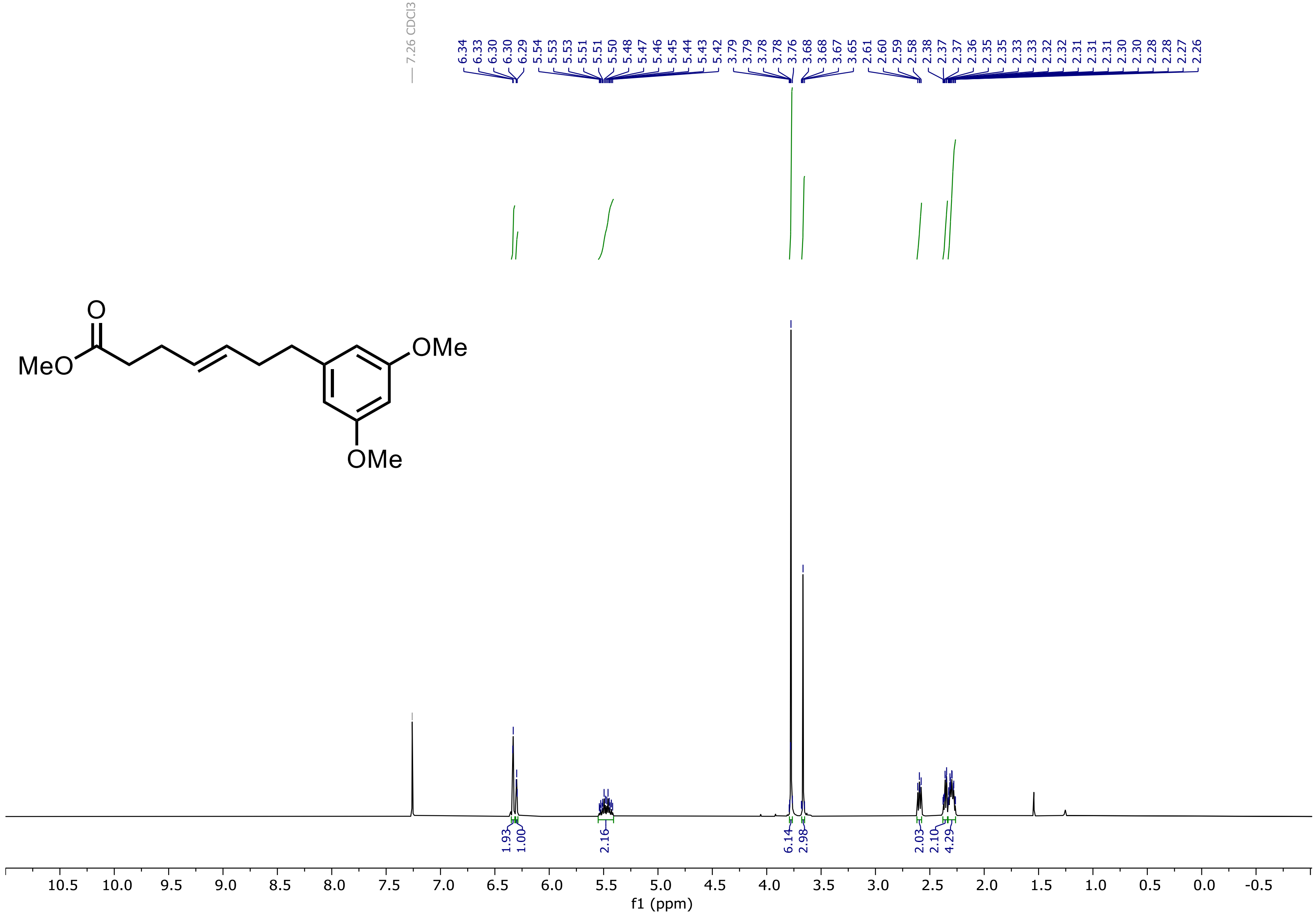

^{13}C NMR of 1-ethyl-3,5-dimethoxybenzene-derived alkylated alkene 1

$CDCl_3$, 23°C

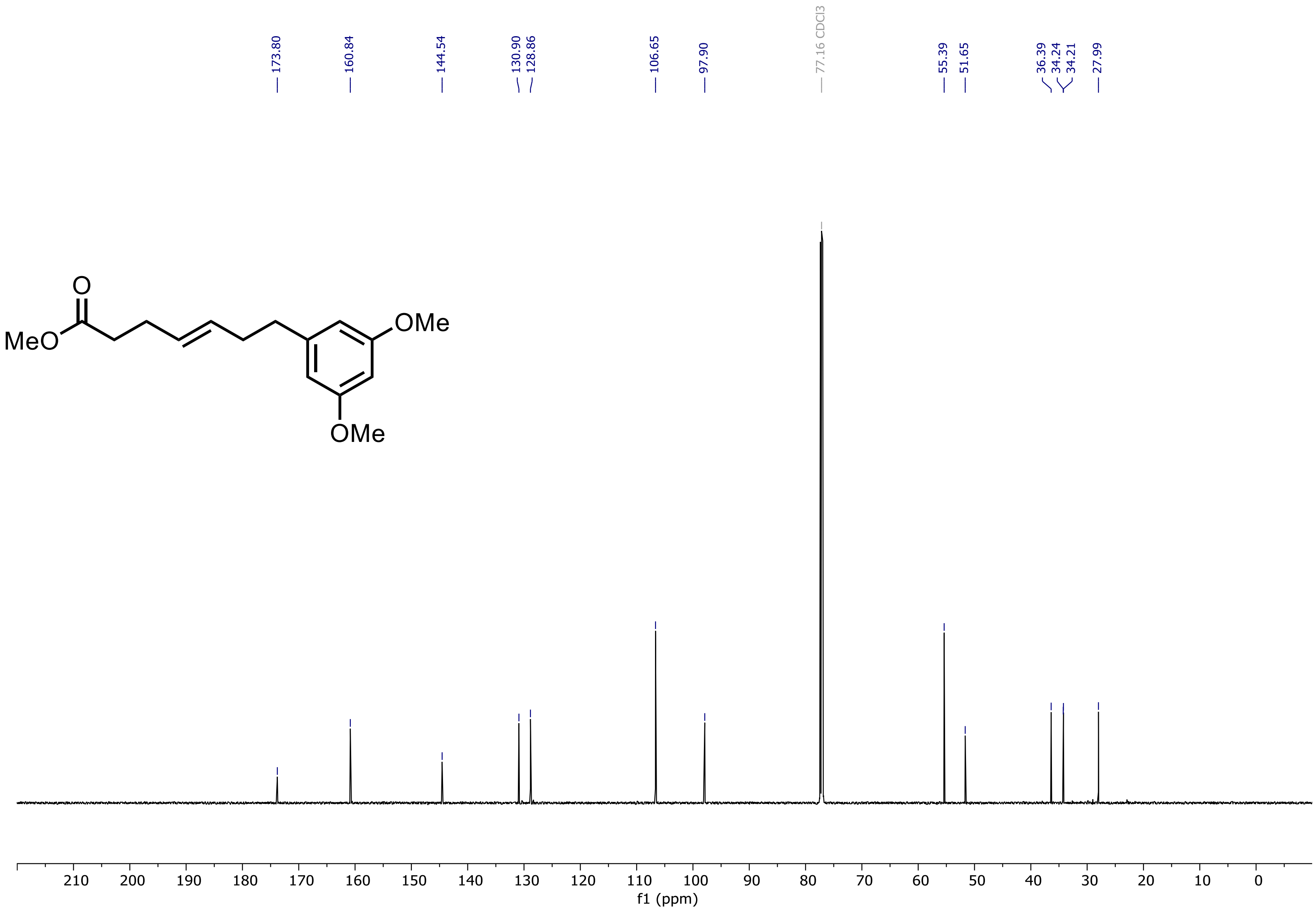

^{1}H NMR of γ-aminobutyric acid-derived alkylated alkene 2

$CDCl_3$, 23°C

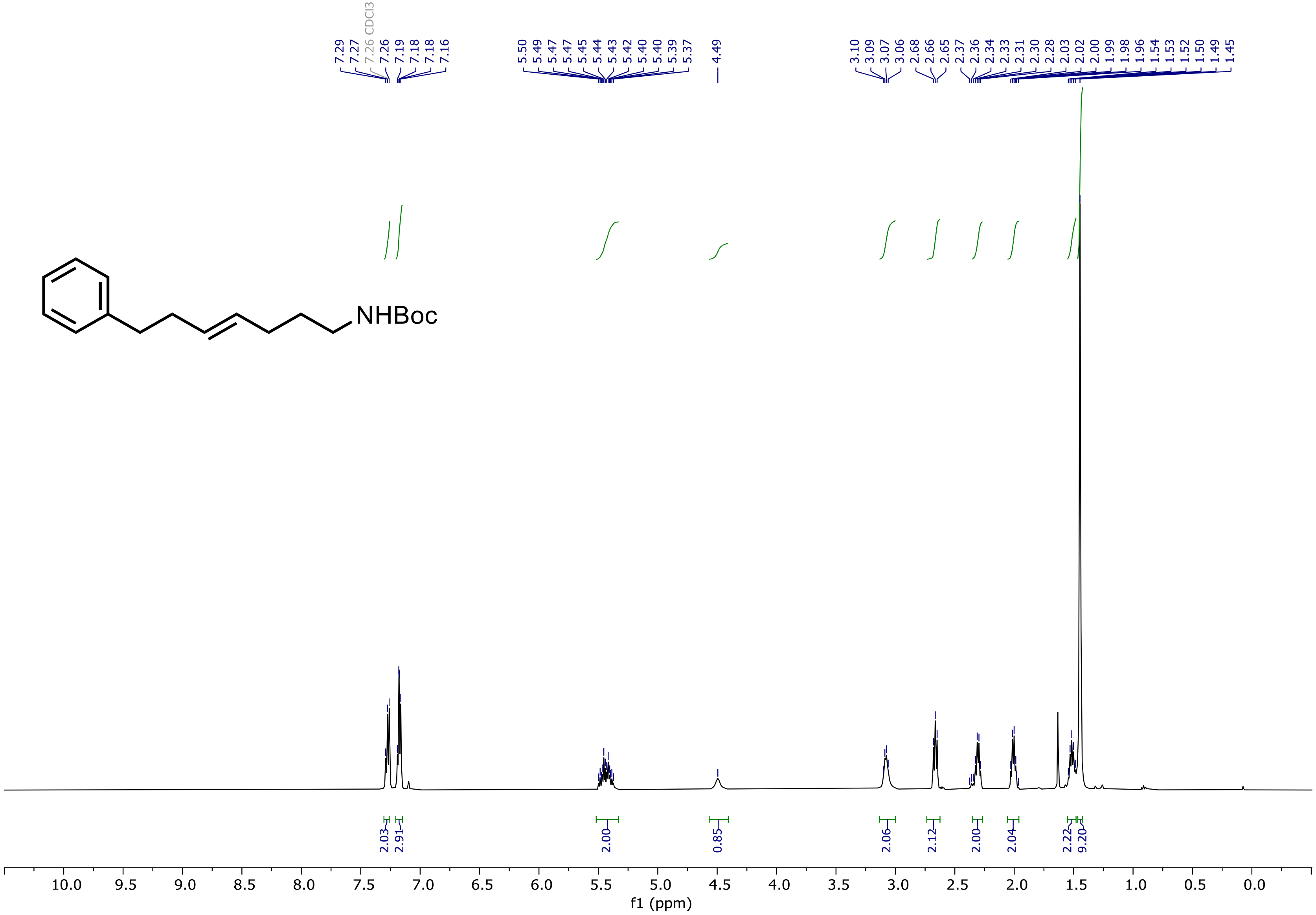

^{13}C NMR of γ-aminobutyric acid-derived alkylated alkene 2

$CDCl_3$, 23°C

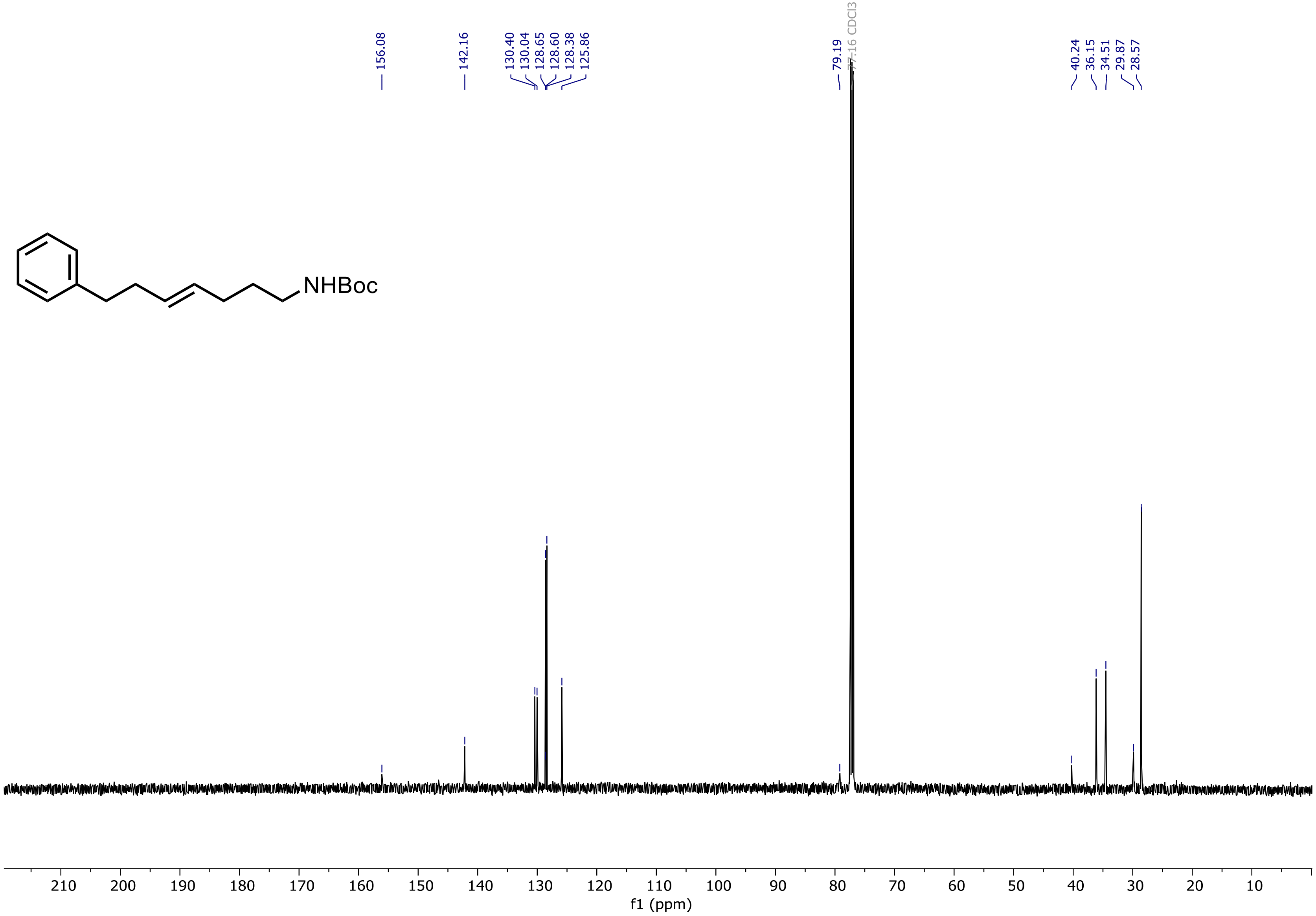

^{1}H NMR of tetrahydropyran-derived alkylated alkene 3

CDCl$_3$, 23°C

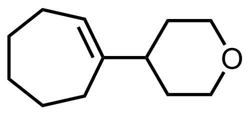

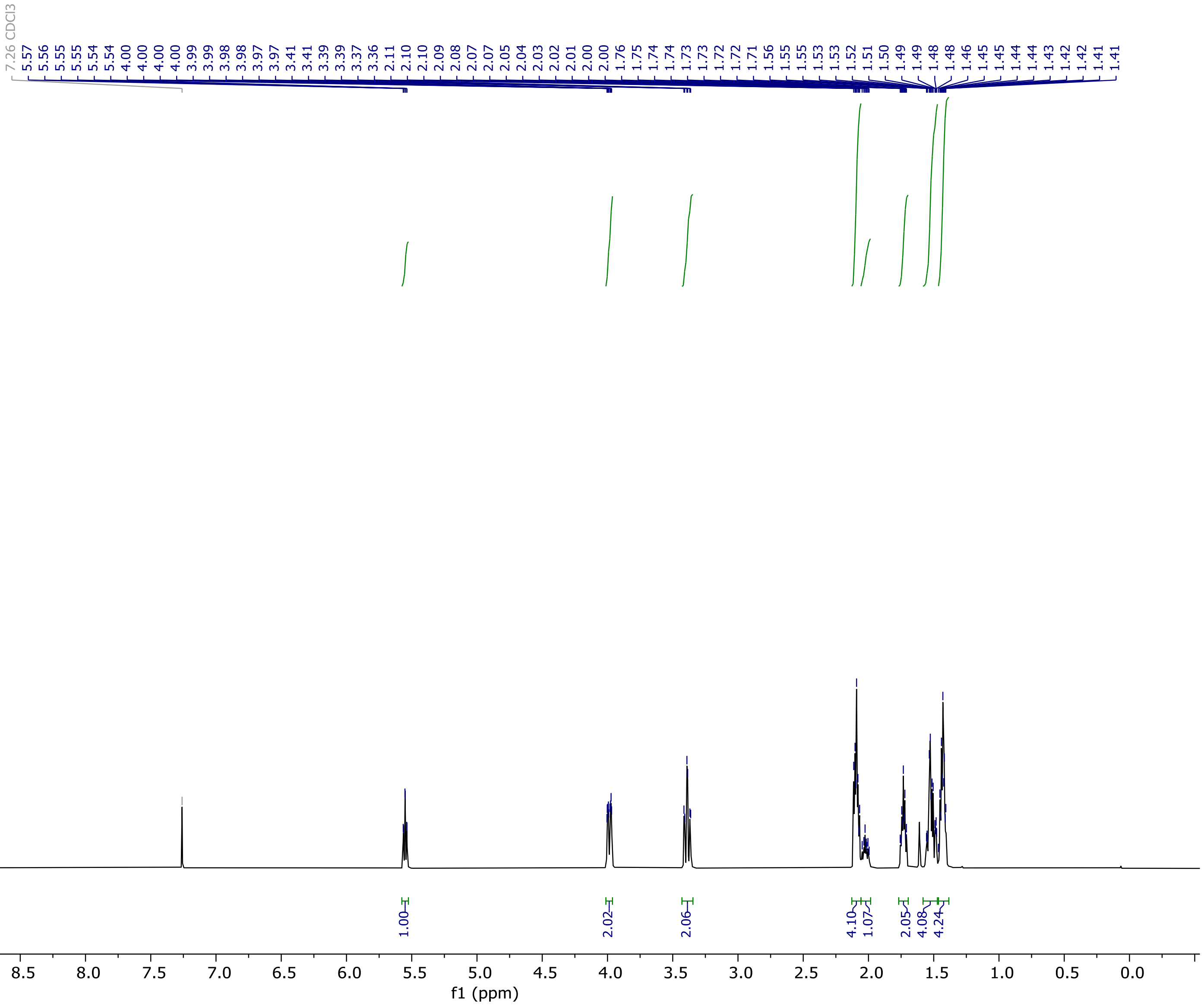

^{13}C NMR of tetrahydropyran-derived alkylated alkene 3

$CDCl_3$, 23°C

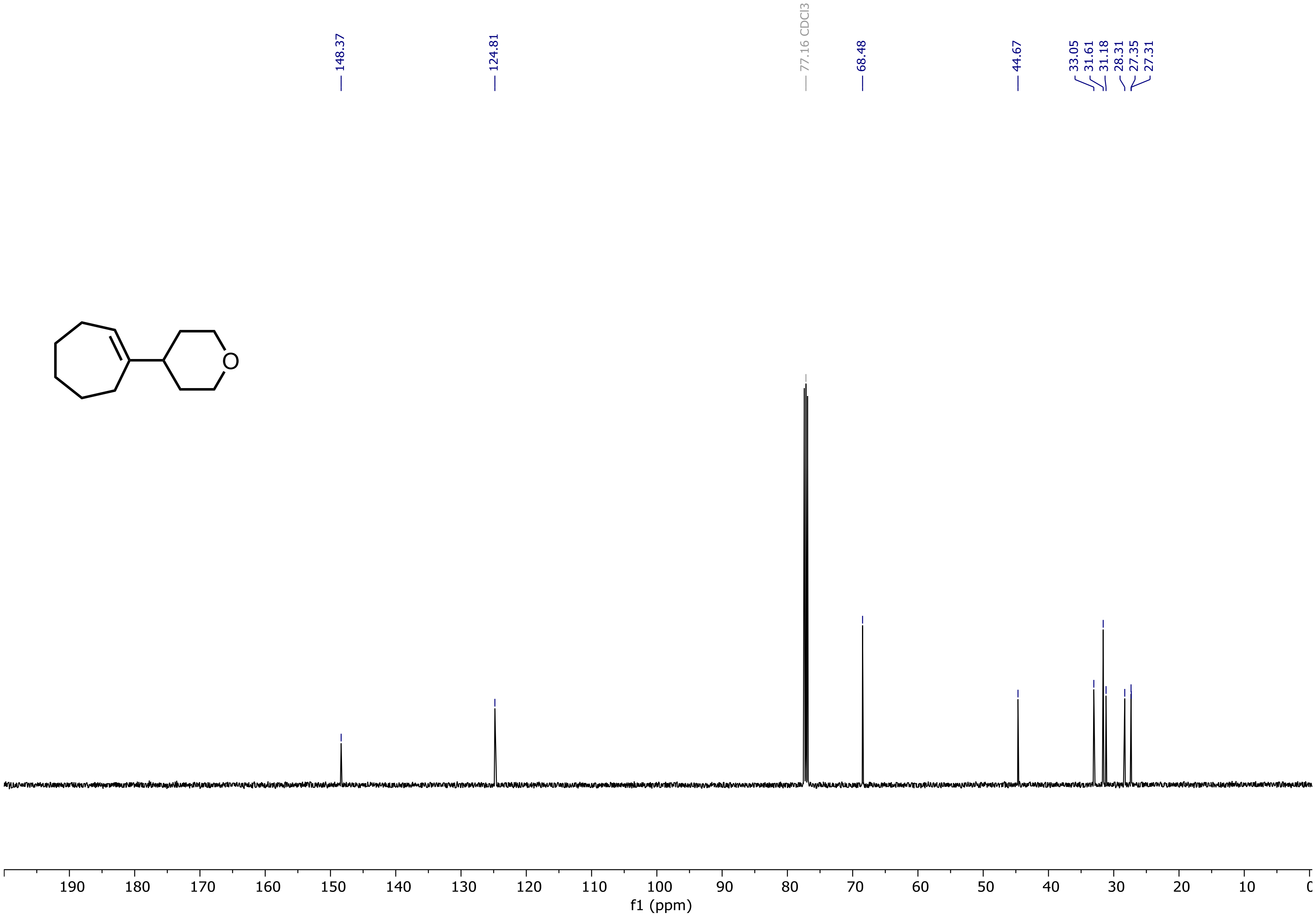

^{1}H NMR of 1-ethyl-3,5-dimethoxybenzene-derived alkylated alkene 4

$CDCl_3$, 23°C

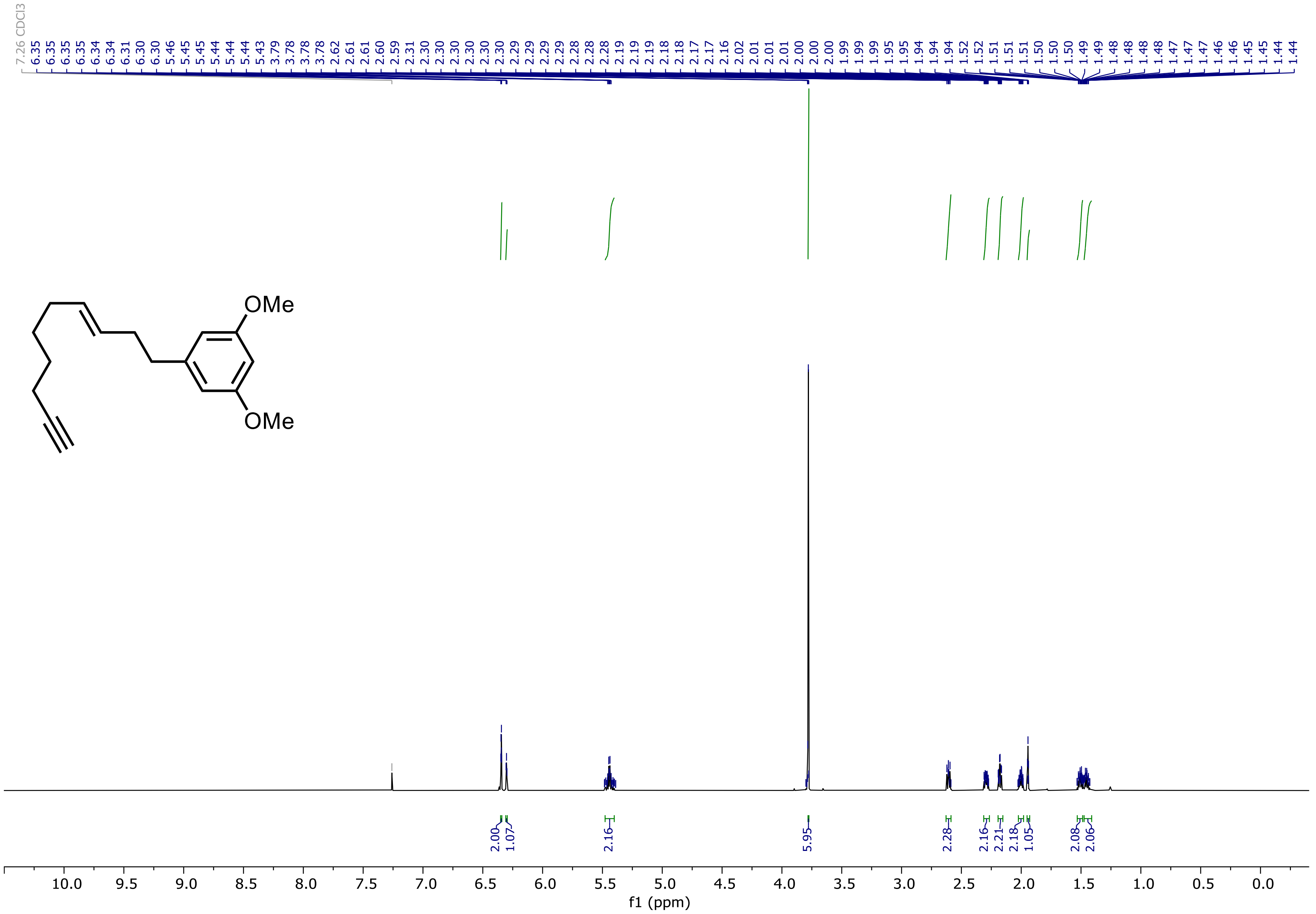

^{13}C NMR of 1-ethyl-3,5-dimethoxybenzene-derived alkylated alkene 4

$CDCl_3$, 23°C

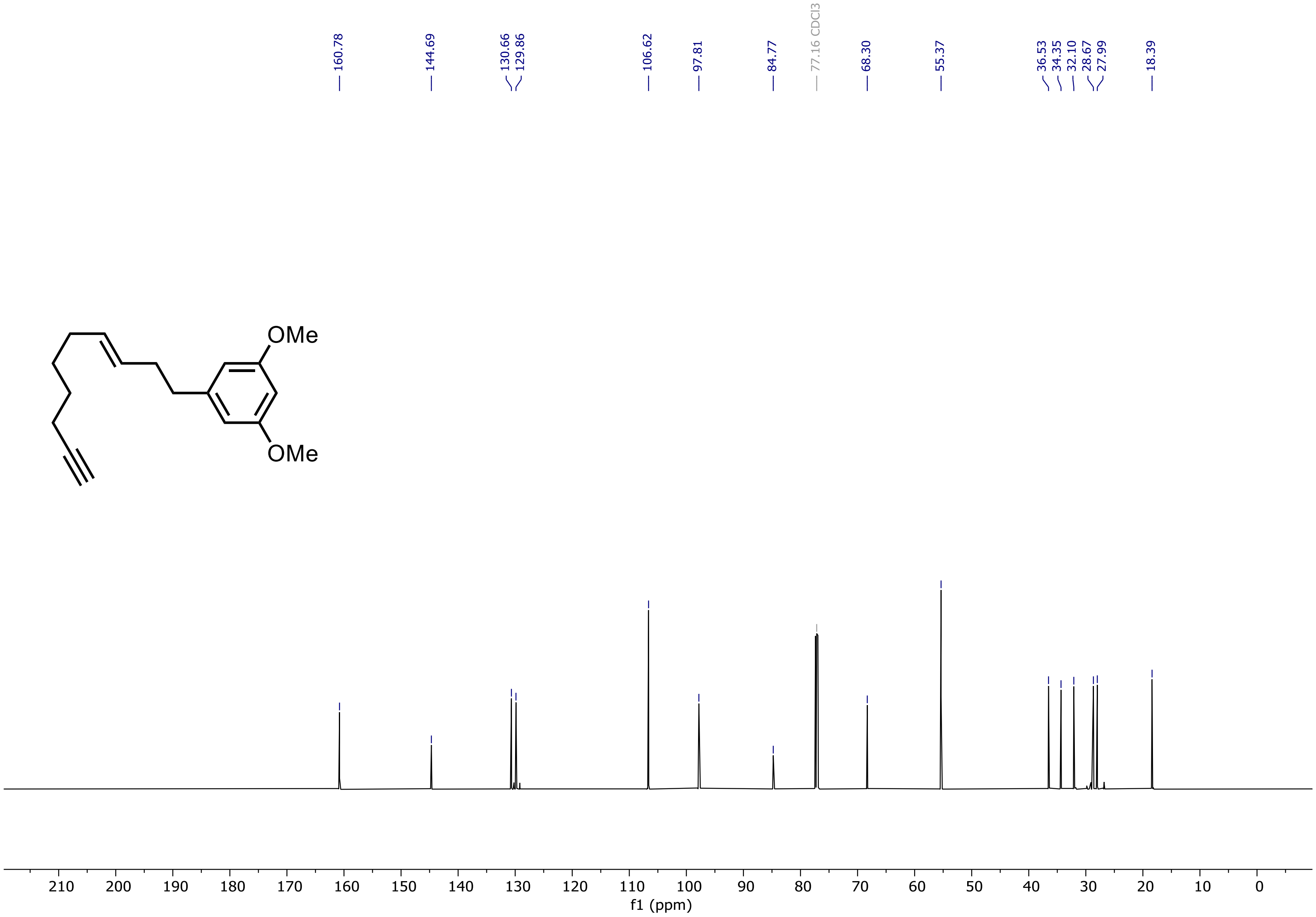

^{1}H NMR of 1,1-difluorocyclohexane-derived alkylated alkene 5

$CDCl_3$, 23°C

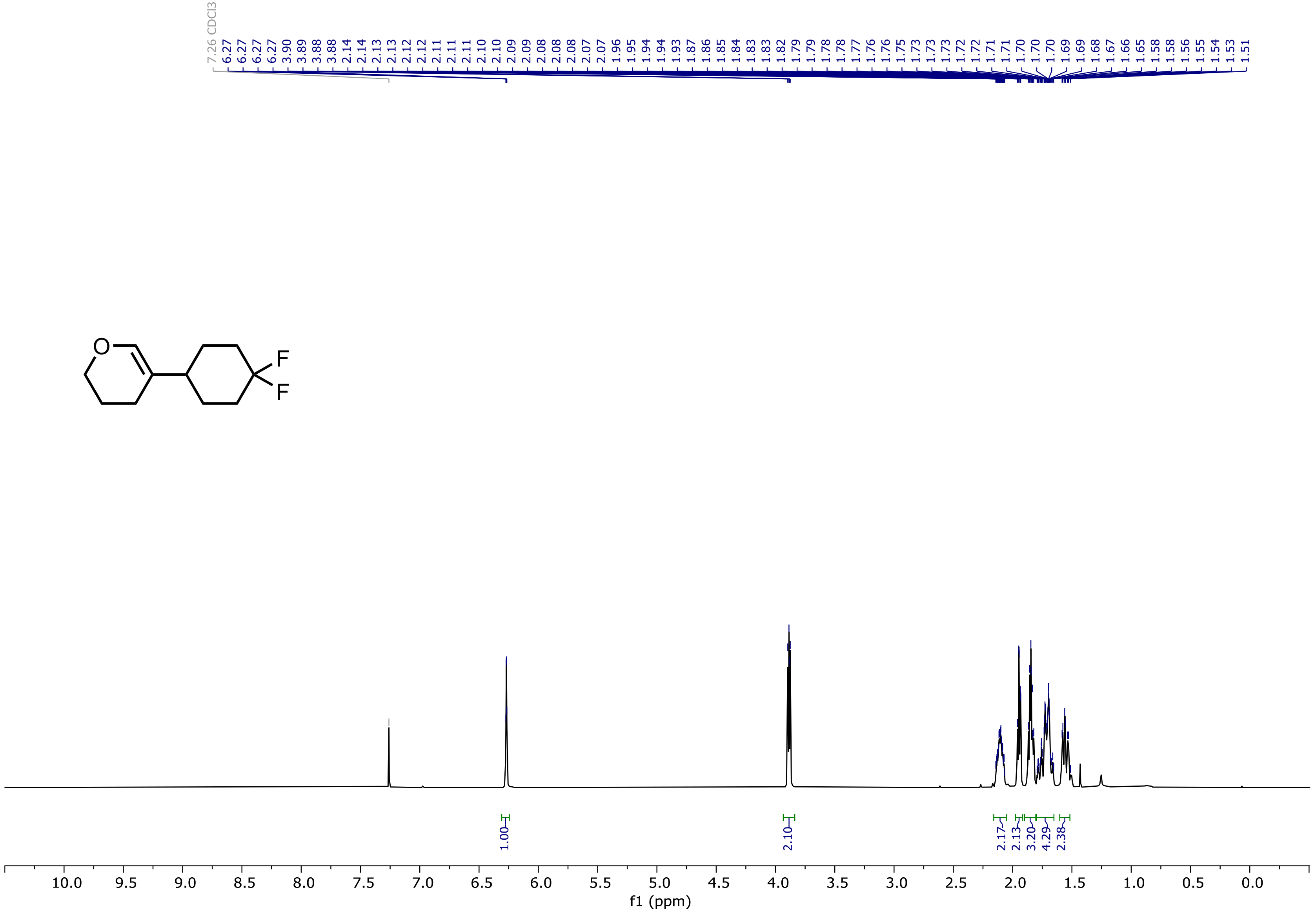

^{13}C NMR of 1,1-difluorocyclohexane-derived alkylated alkene 5

CDCl$_3$, 23°C

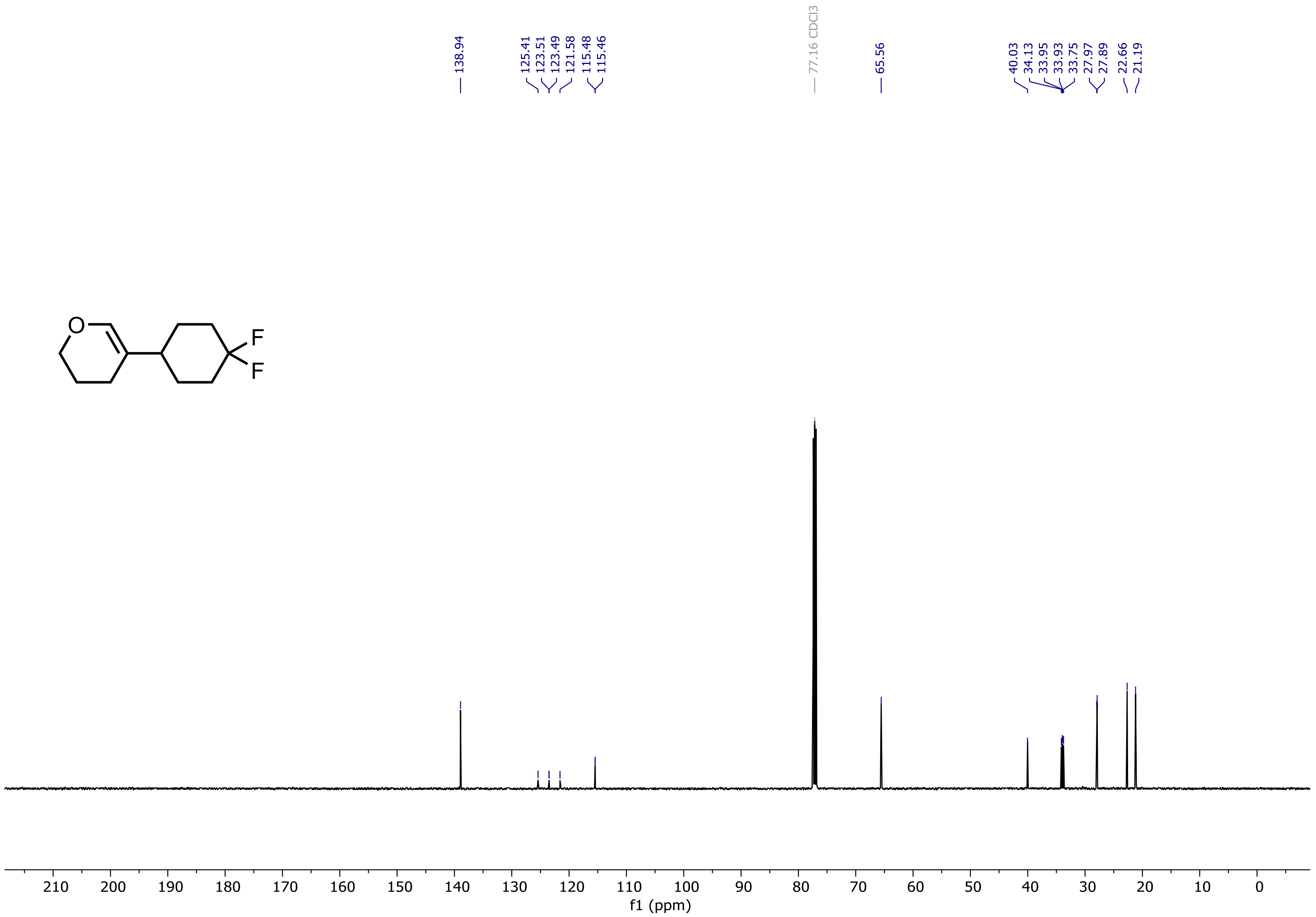

^{19}F NMR of 1,1-difluorocyclohexane-derived alkylated alkene 5

$CDCl_3$, 23°C

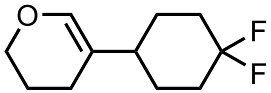

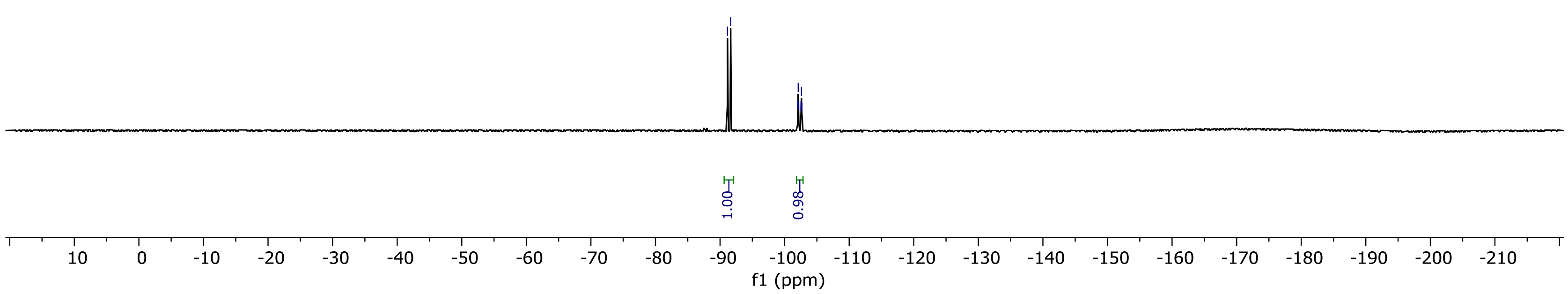

^{1}H NMR of 1-Benzoylpiperidine-derived alkylated alkene 6

$CDCl_3$, 23°C

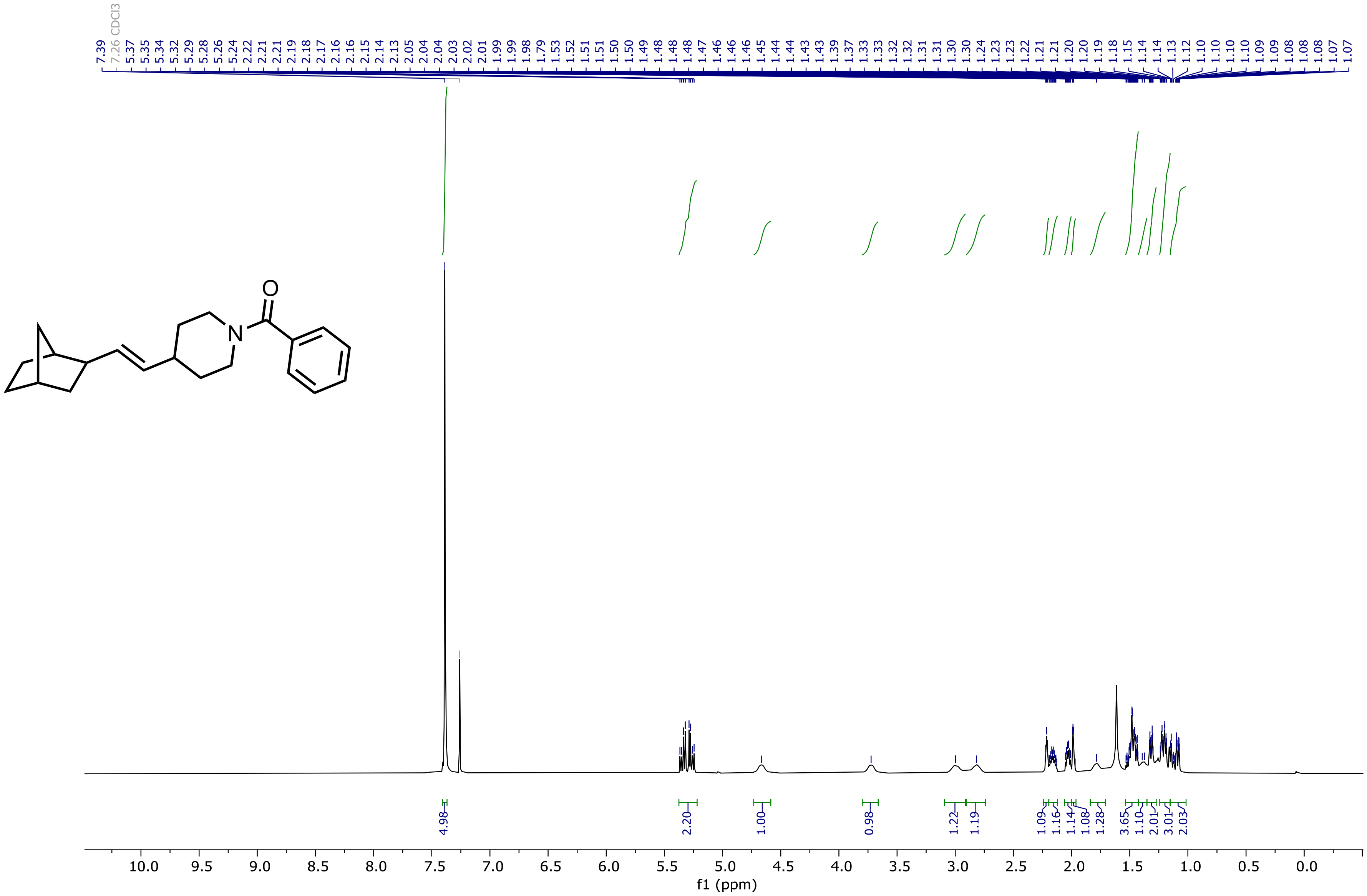

^{13}C NMR of 1-Benzoylpiperidine-derived alkylated alkene 6

$CDCl_3$, 23°C

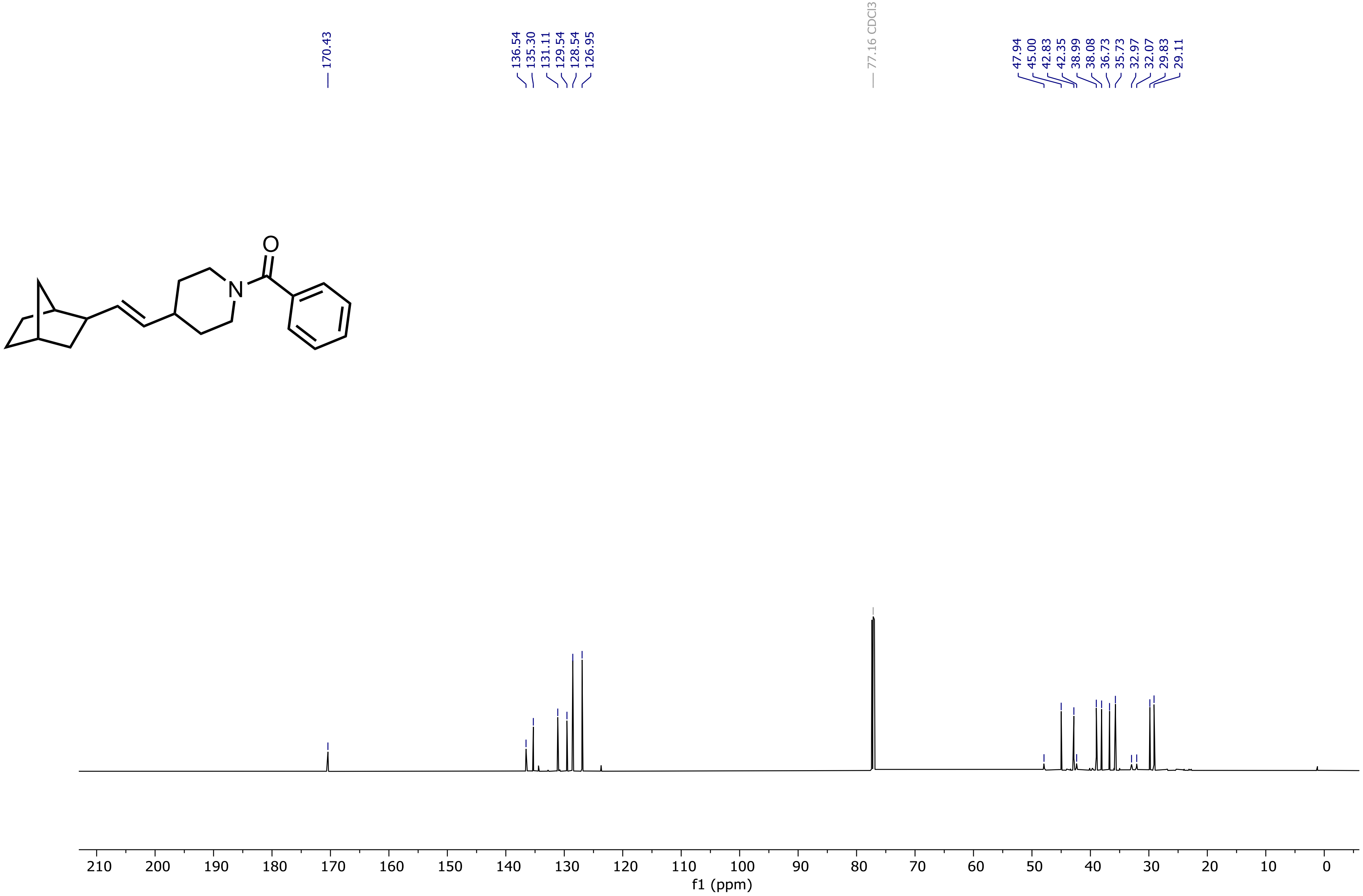

^{1}H NMR of *N-tert*-butyloxycarbonyl-azetidine-derived alkylated alkene 7

$CDCl_3$, 23°C

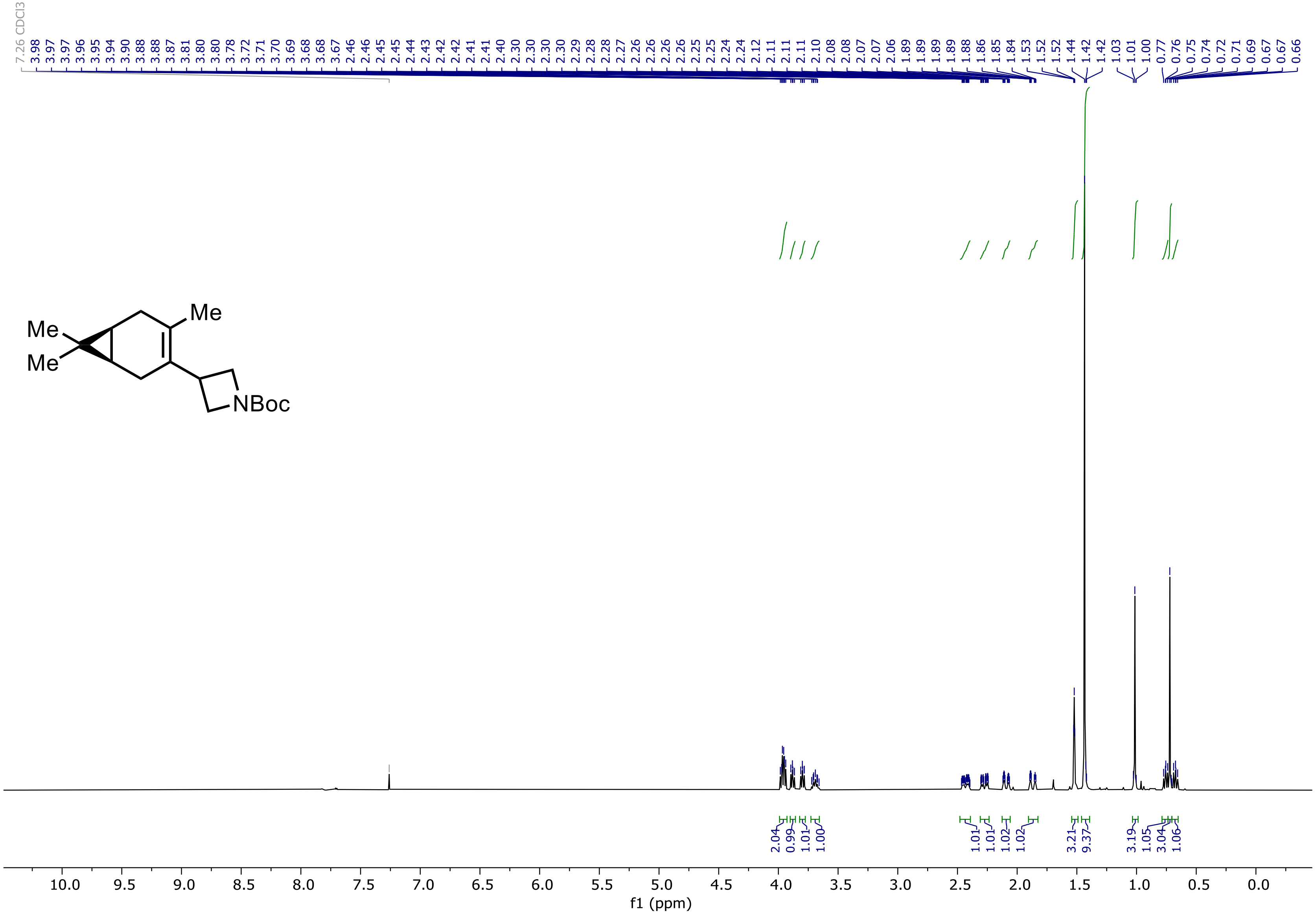

^{13}C NMR of *N-tert*-butyloxycarbonyl-azetidine-derived alkylated alkene 7

$CDCl_3$, 23°C

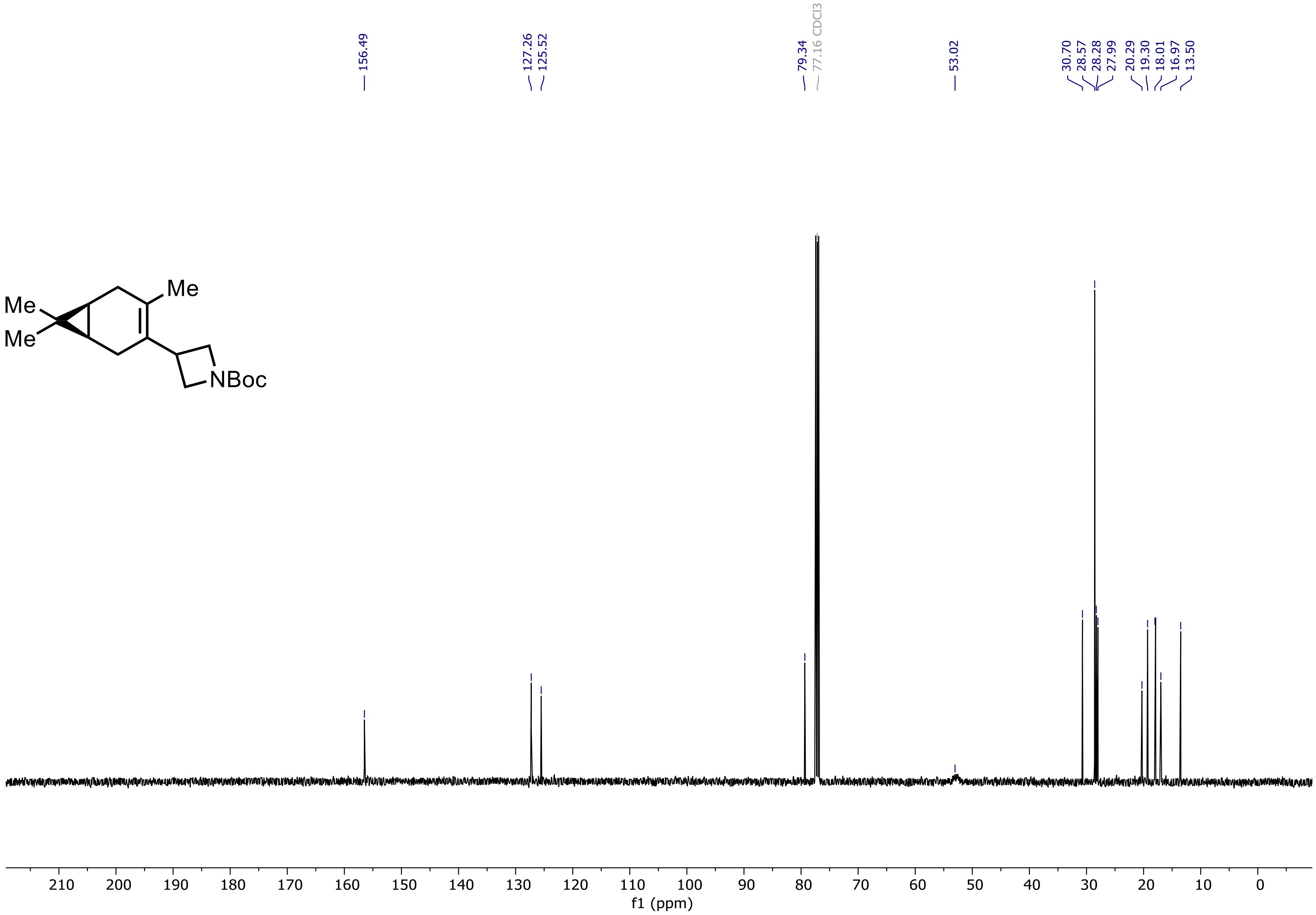

^{1}H NMR of butanone-derived alkylated alkene 8

$CDCl_3$, 23°C

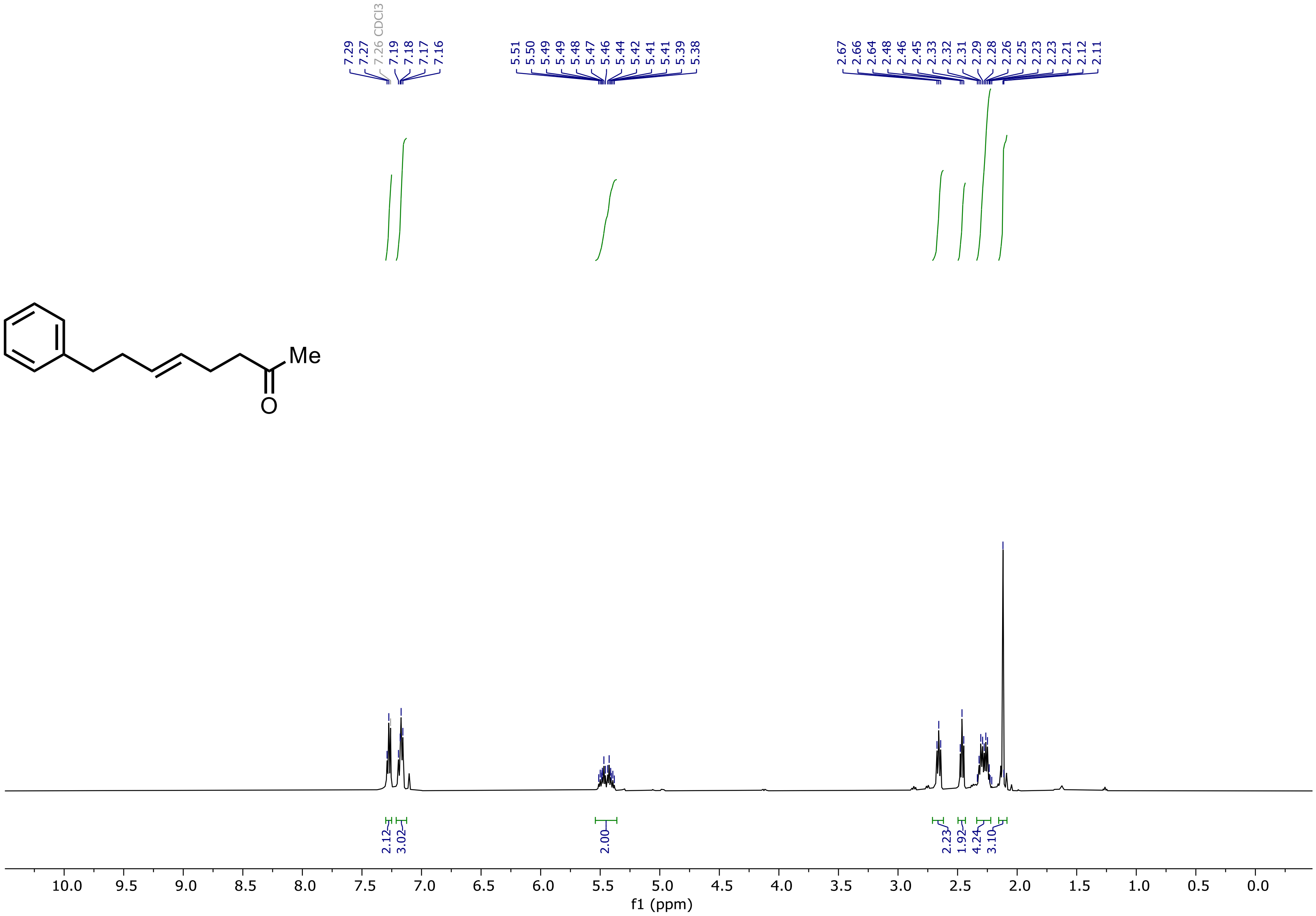

^{13}C NMR of butanone-derived alkylated alkene 8

$CDCl_3$, 23°C

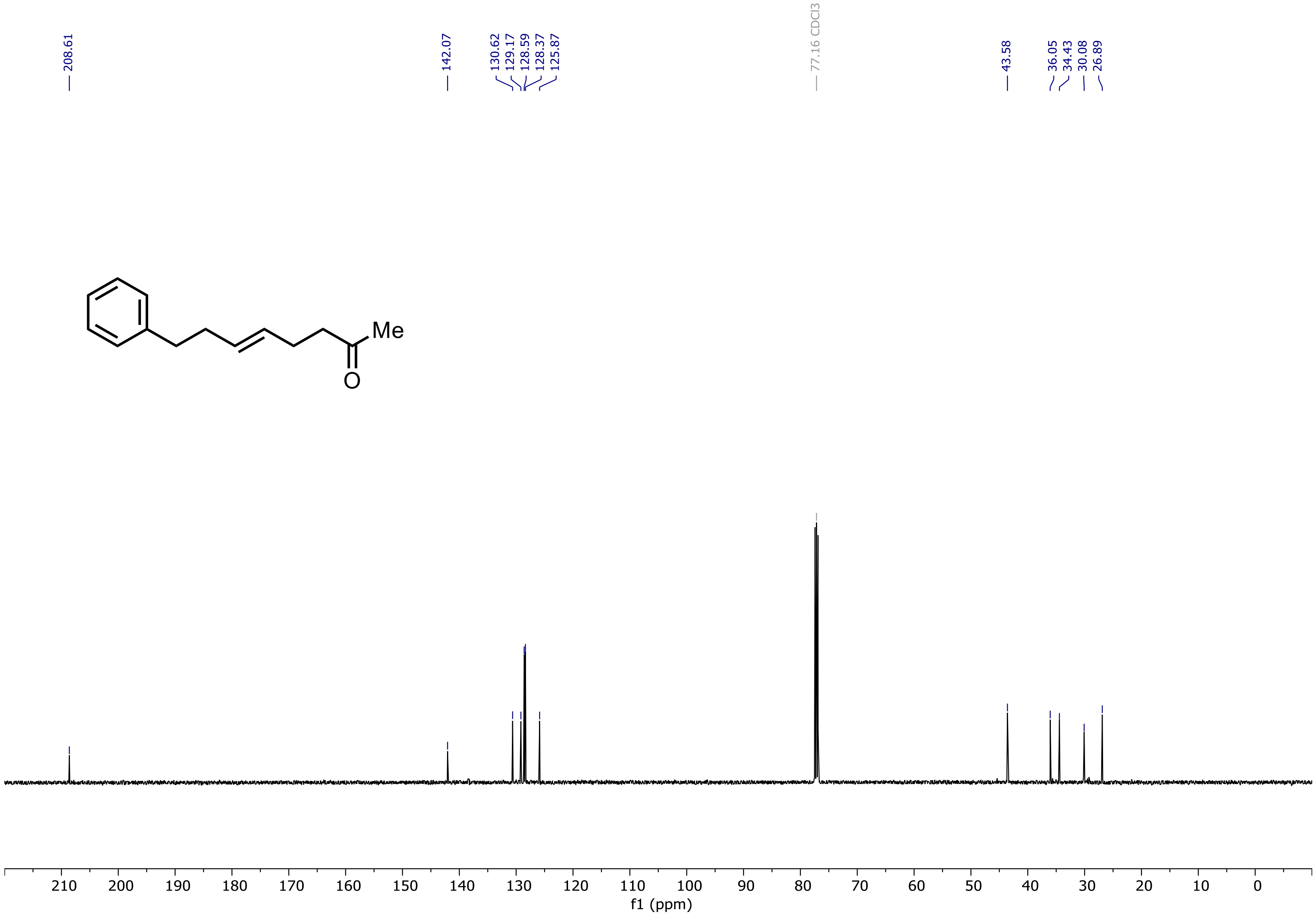

^{1}H NMR of cyclohexanone-derived alkylated alkene 9

$CDCl_3$, 23°C

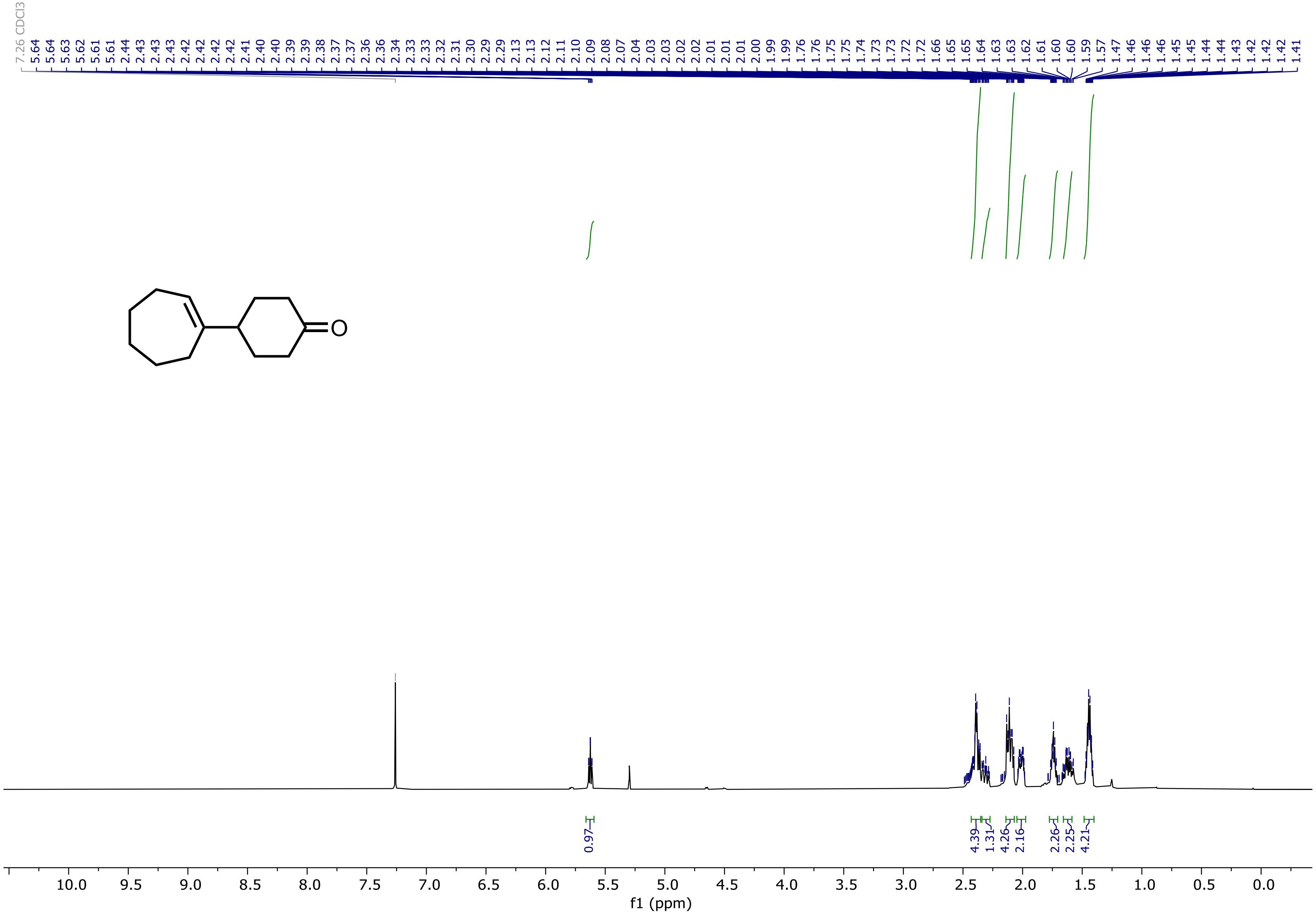

^{13}C NMR of cyclohexanone-derived alkylated alkene 9

CDCl3, 23°C

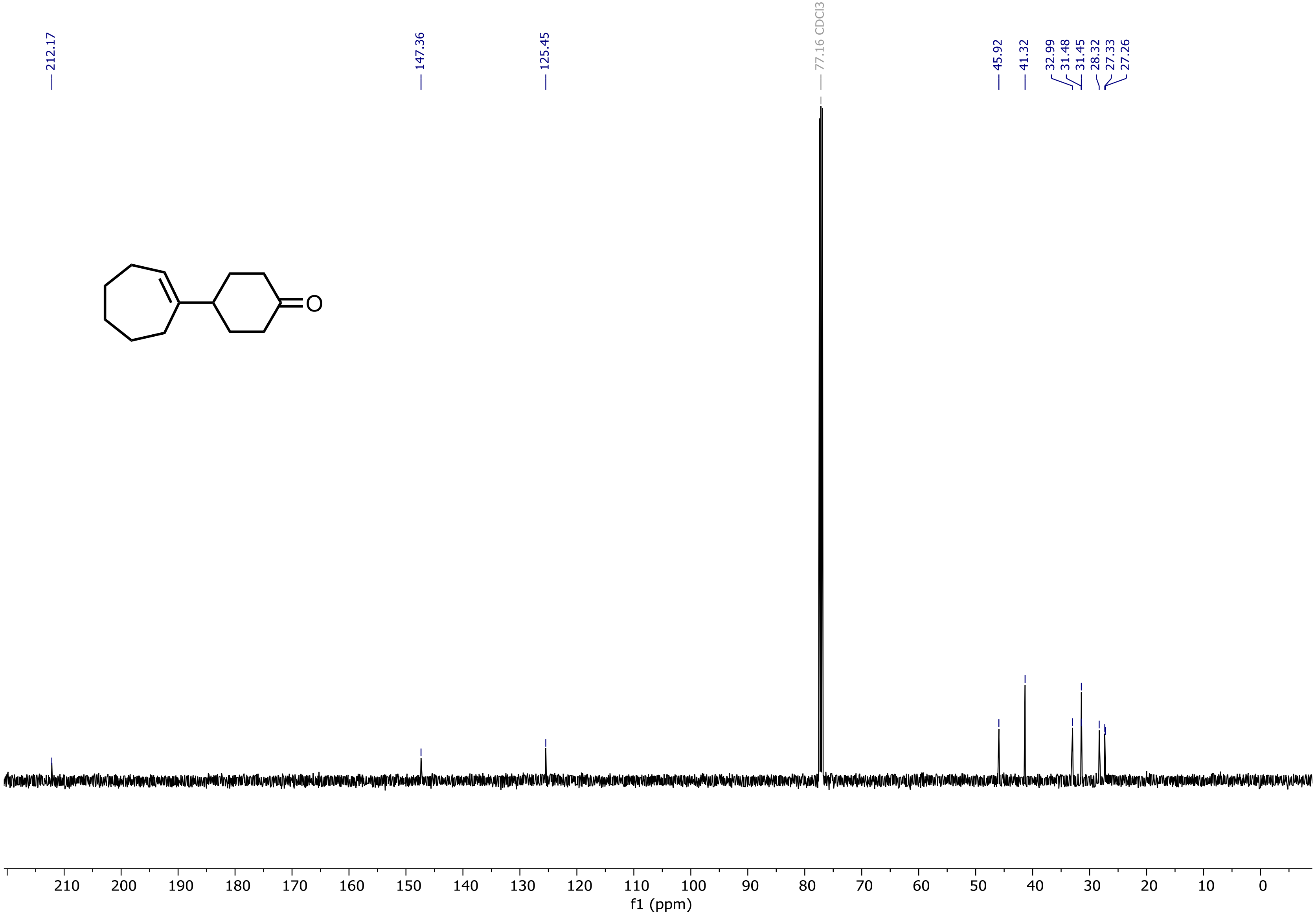

^{1}H NMR of *tert*-butyl-propionate-derived alkylated alkene 10

CDCl$_3$, 23°C

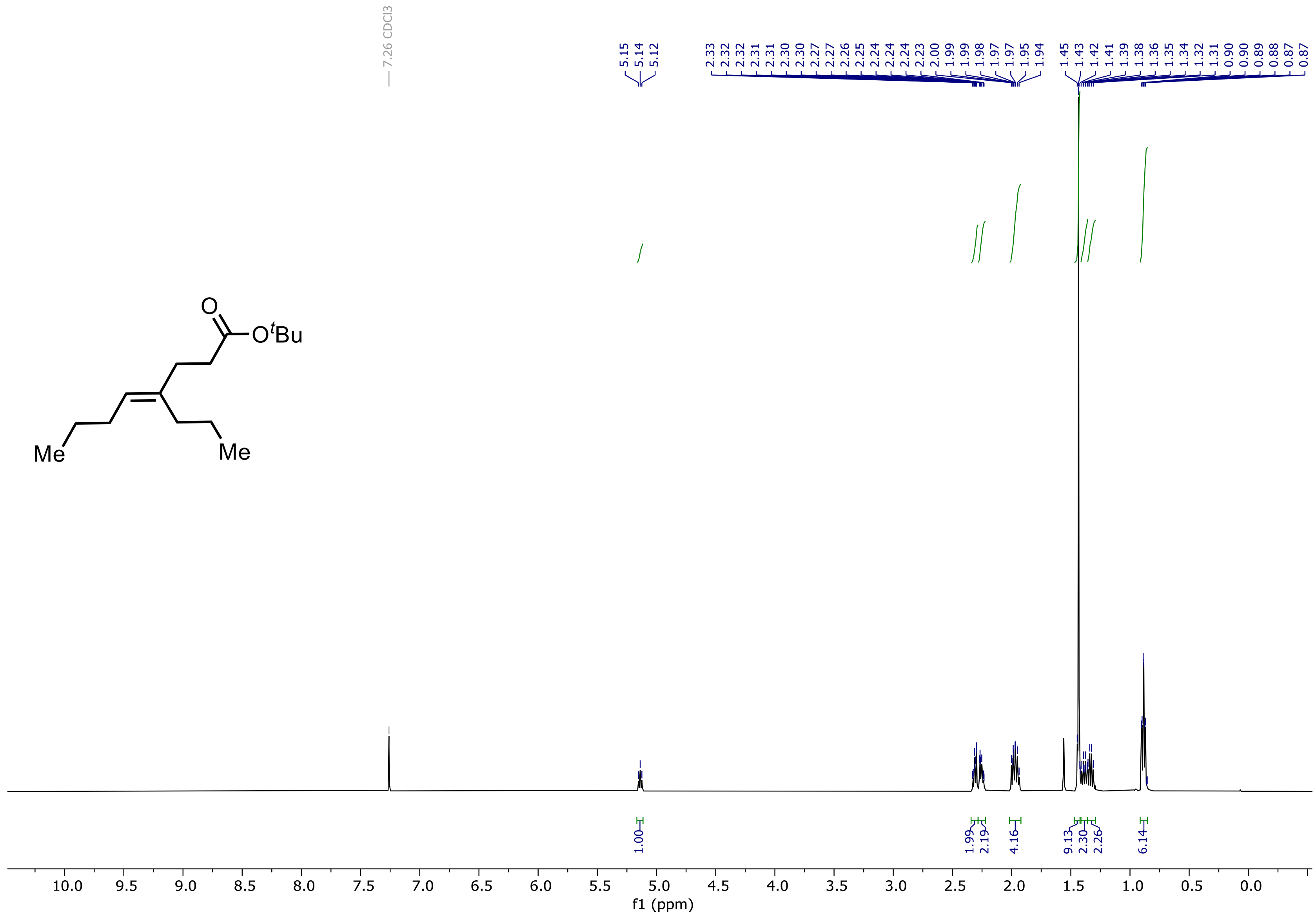

^{13}C NMR of *tert*-butyl-propionate-derived alkylated alkene 10

CDCl$_3$, 23°C

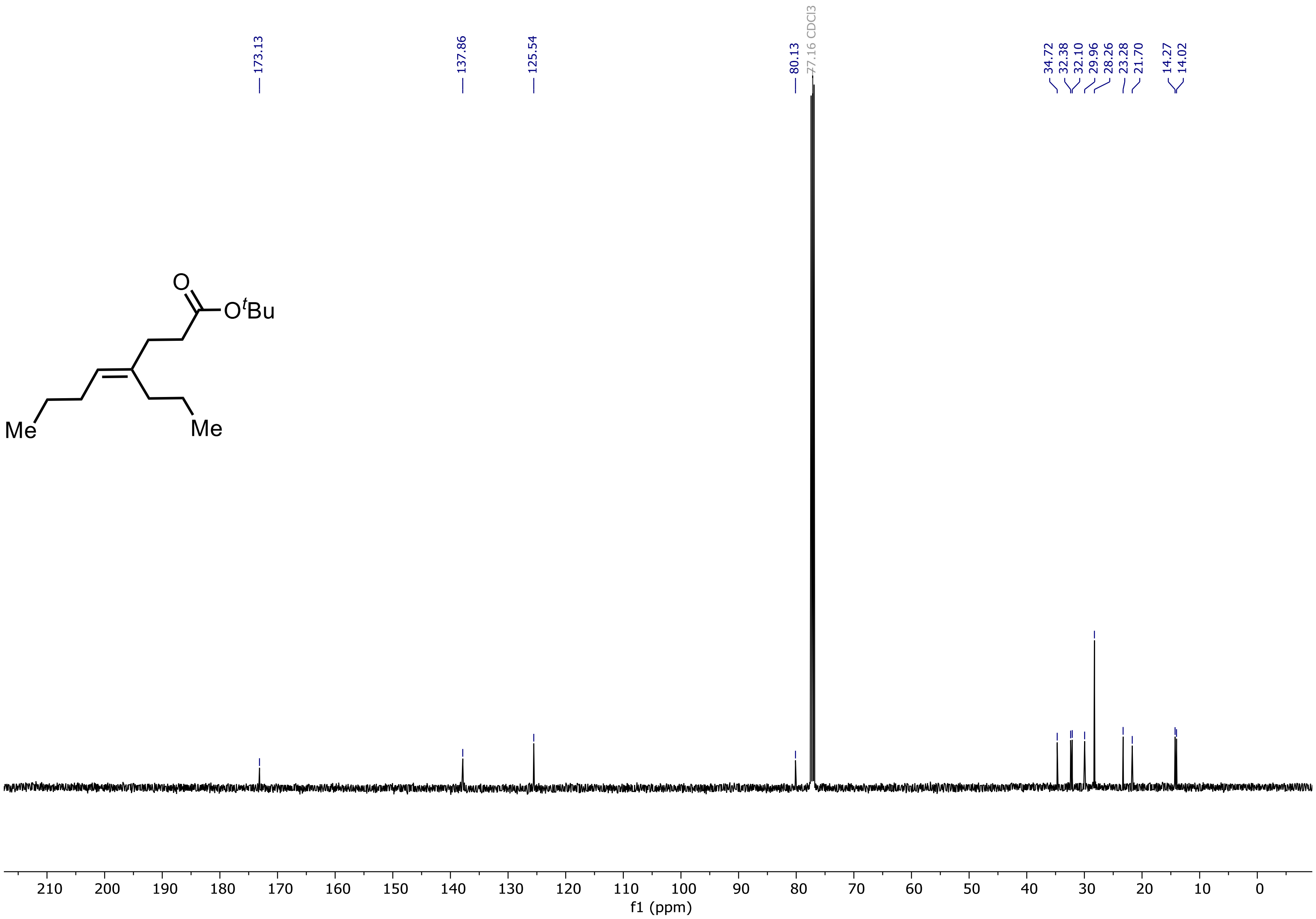

^{1}H NMR of indometacin-derived alkylated alkene 11

CDCl$_3$, 23°C

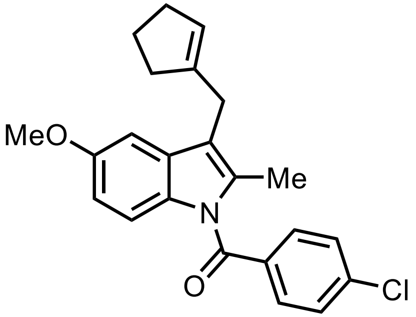

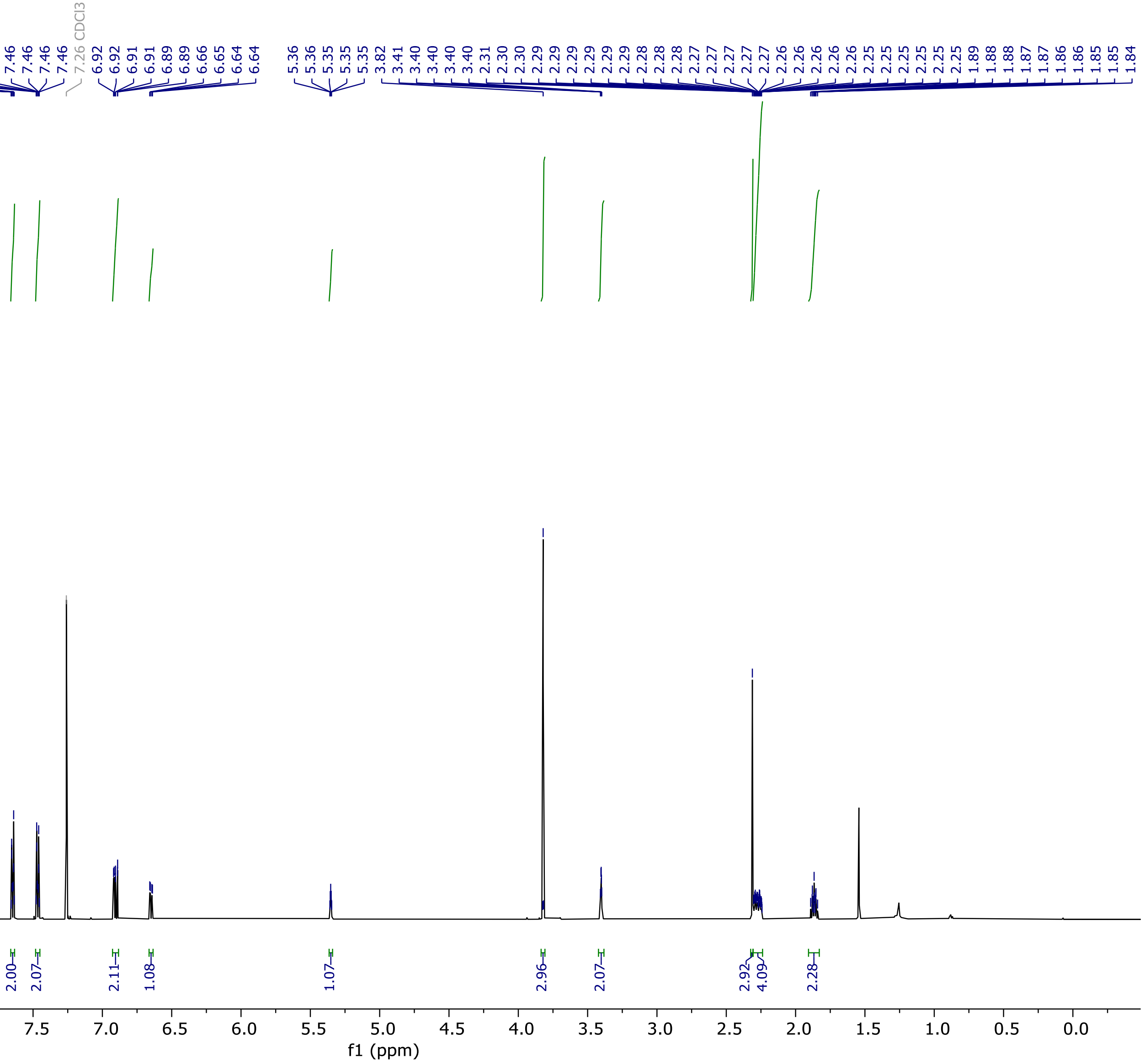

^{13}C NMR of indometacin-derived alkylated alkene 11

CDCl$_3$, 23°C

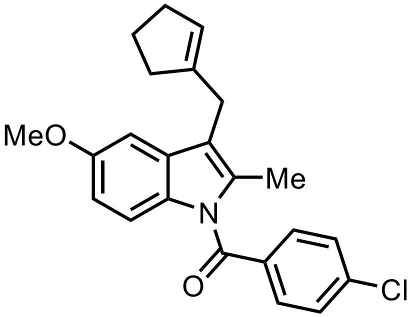

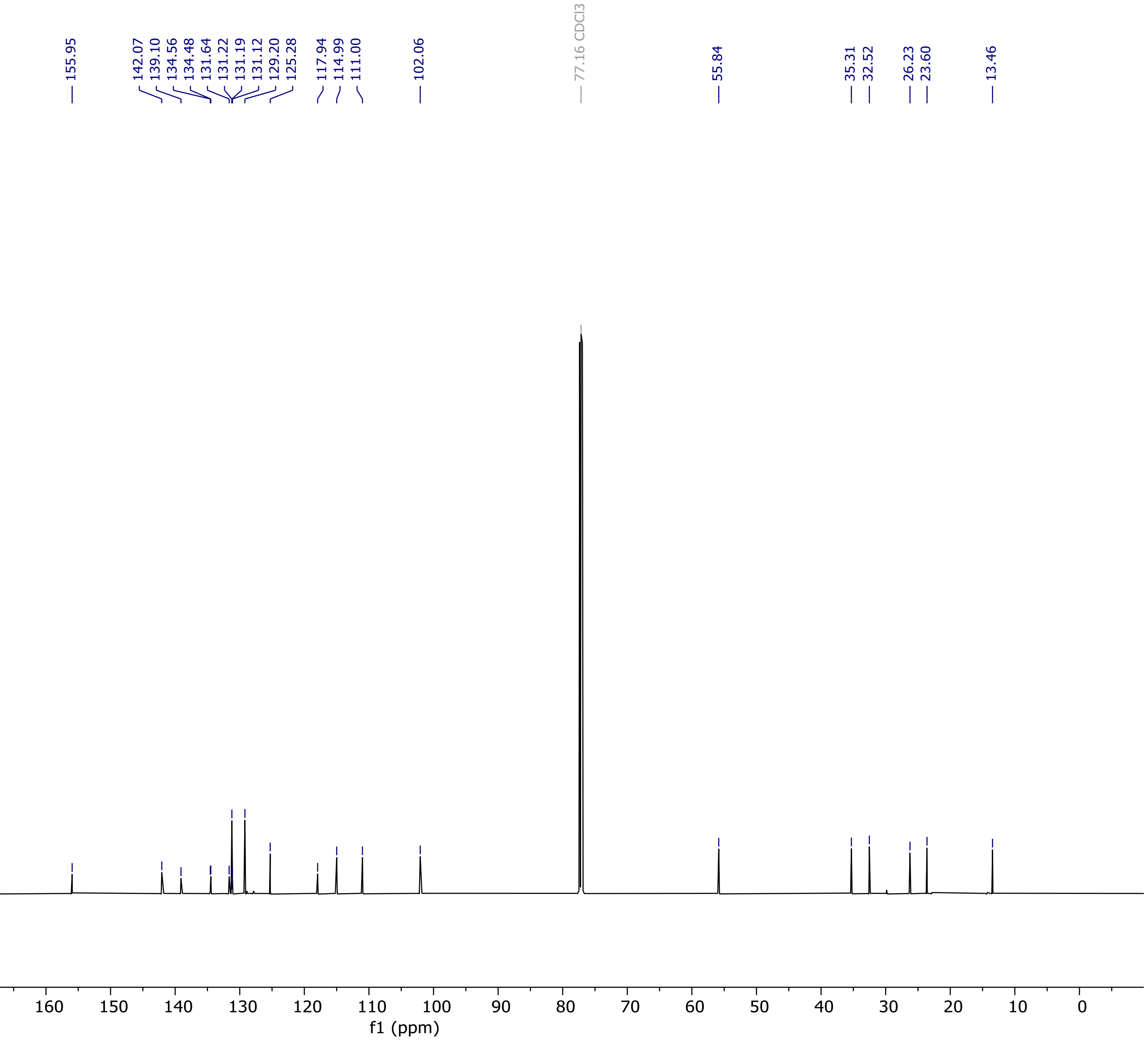

^{1}H NMR of methoxymethane-derived alkylated alkene 12

CDCl$_3$, 23°C

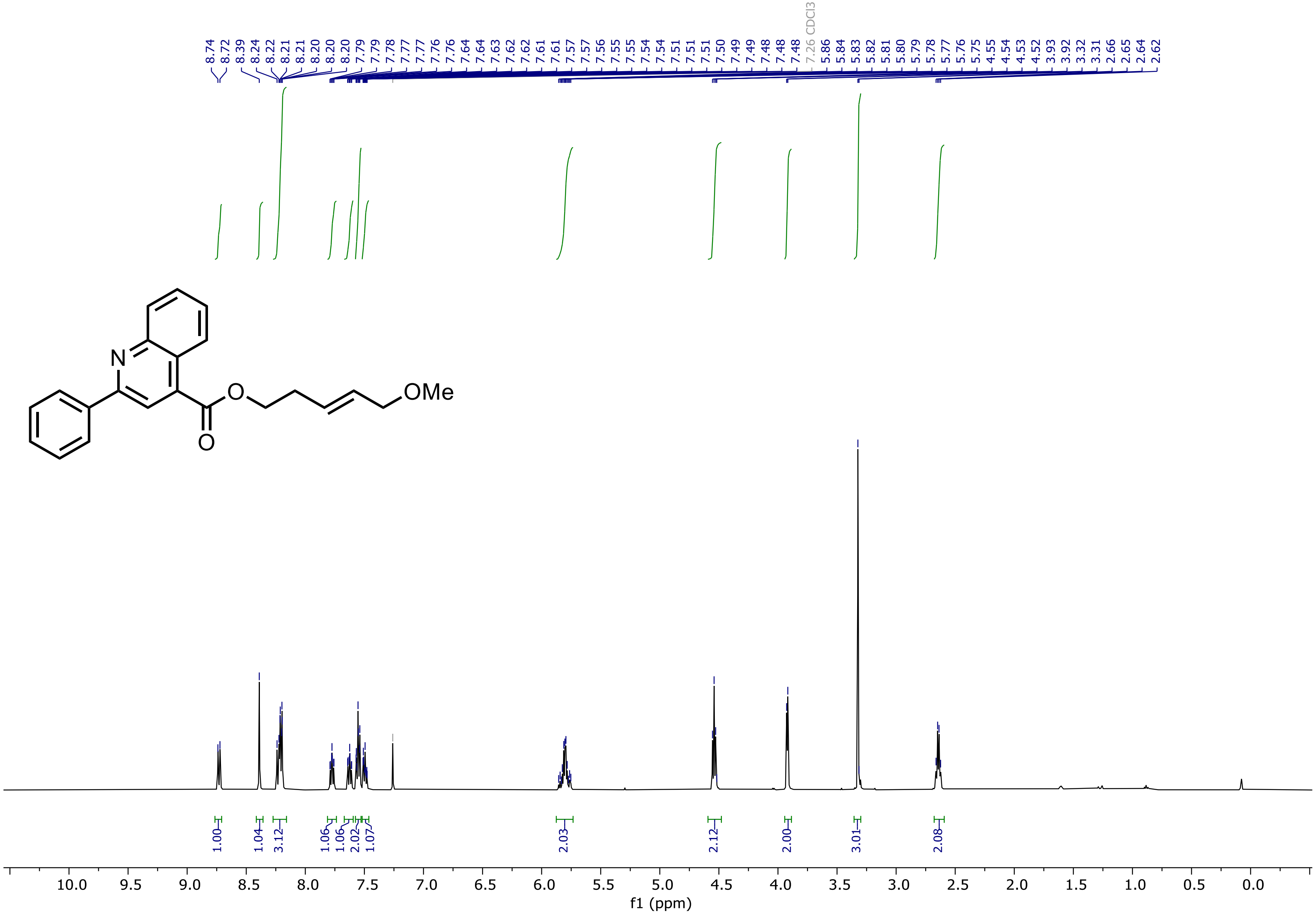

^{13}C NMR of methoxymethane-derived alkylated alkene 12

$CDCl_3$, 23°C

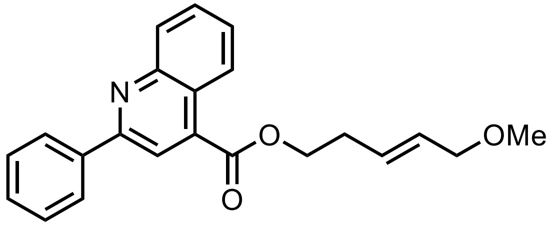

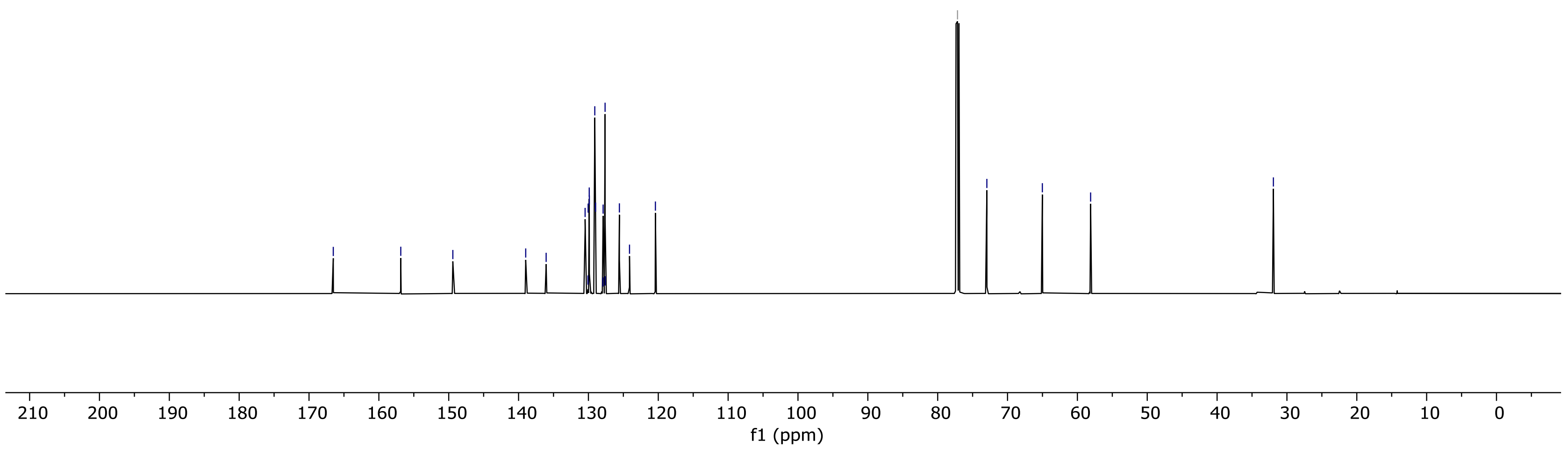

^{1}H NMR of 1-Benzoylpiperidine-derived alkylated alkene (±)-13

CDCl$_3$, 23°C

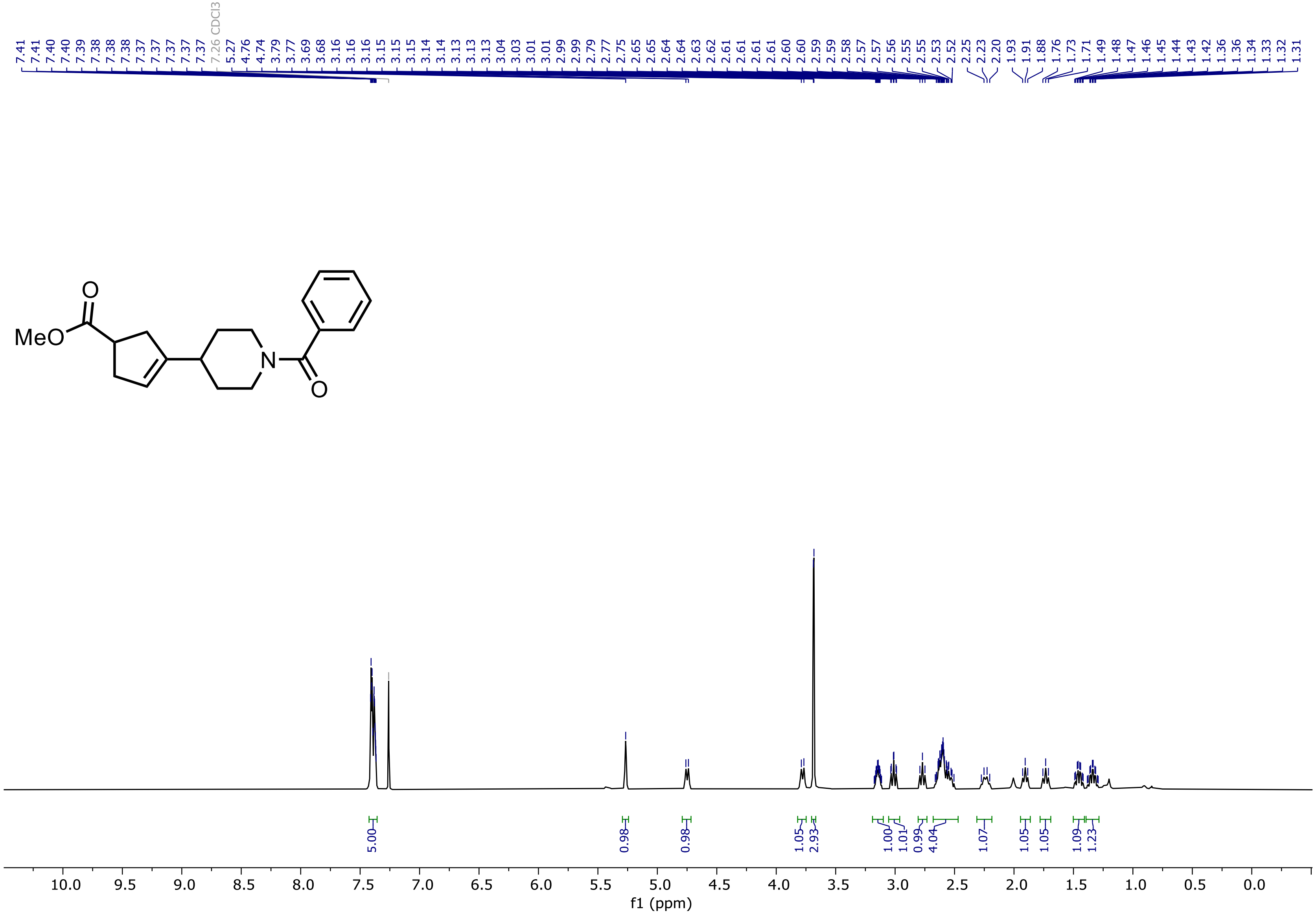

^{13}C NMR of 1-Benzoylpiperidine-derived alkylated alkene (±)-13

$CDCl_3$, 23°C

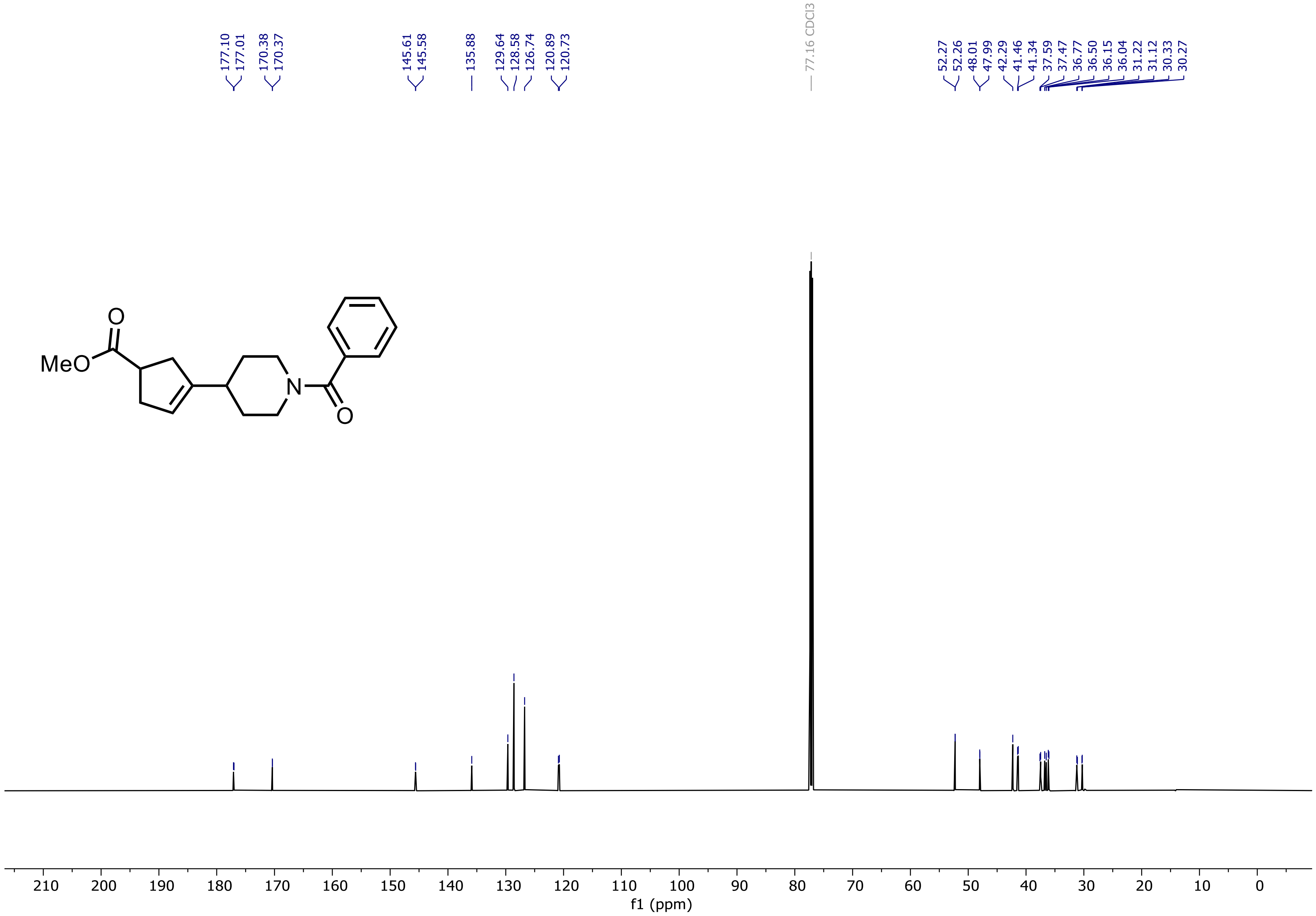

^{1}H NMR of *N-tert*-butyloxycarbonyl-azetidine-derived alkylated alkene 14

$CDCl_3$, 23°C

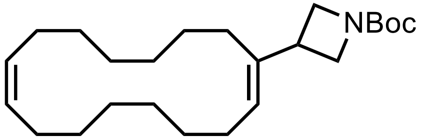

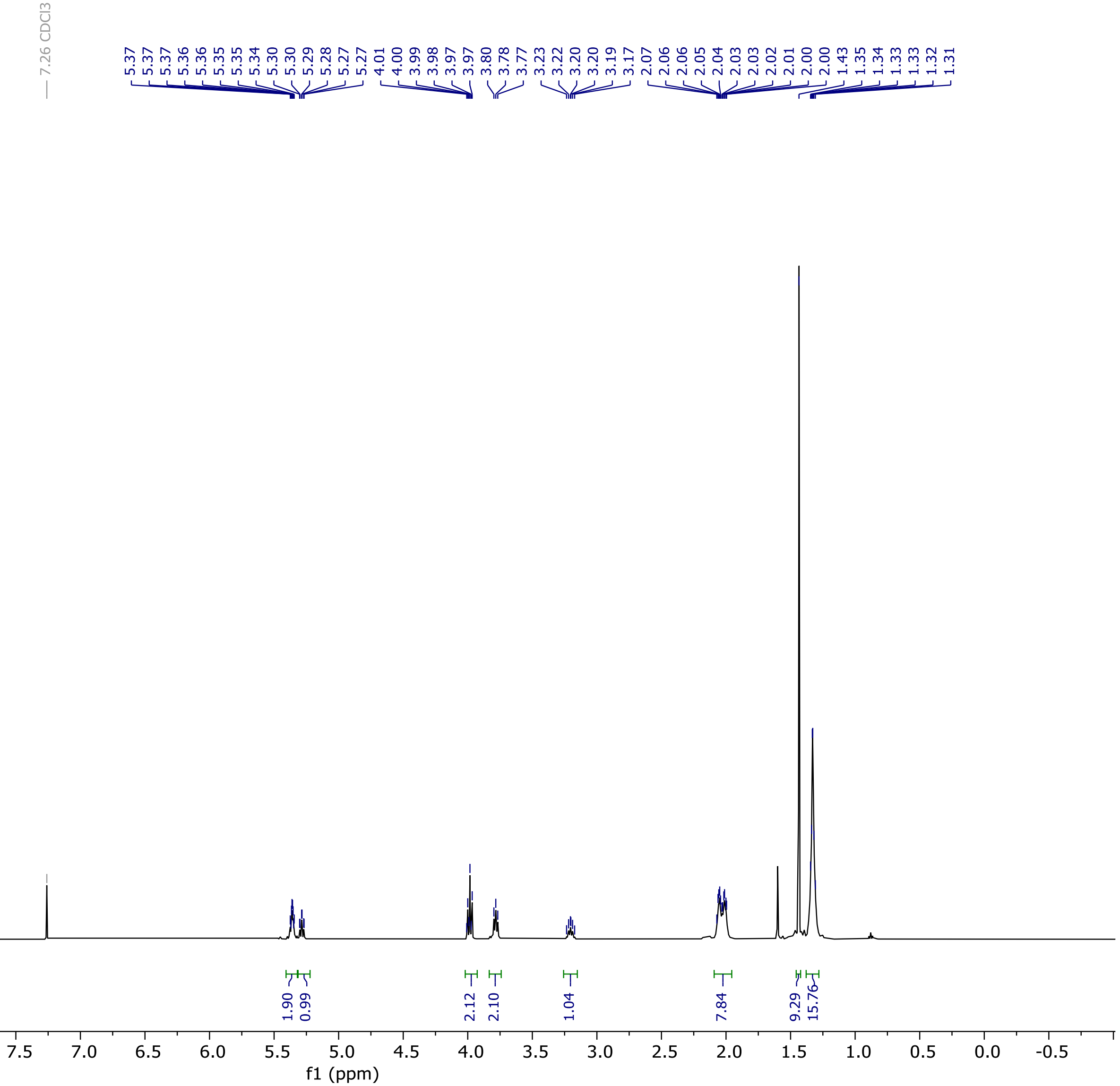

^{13}C NMR of *N-tert*-butyloxycarbonyl-azetidine-derived alkylated alkene 14

$CDCl_3$, 23°C

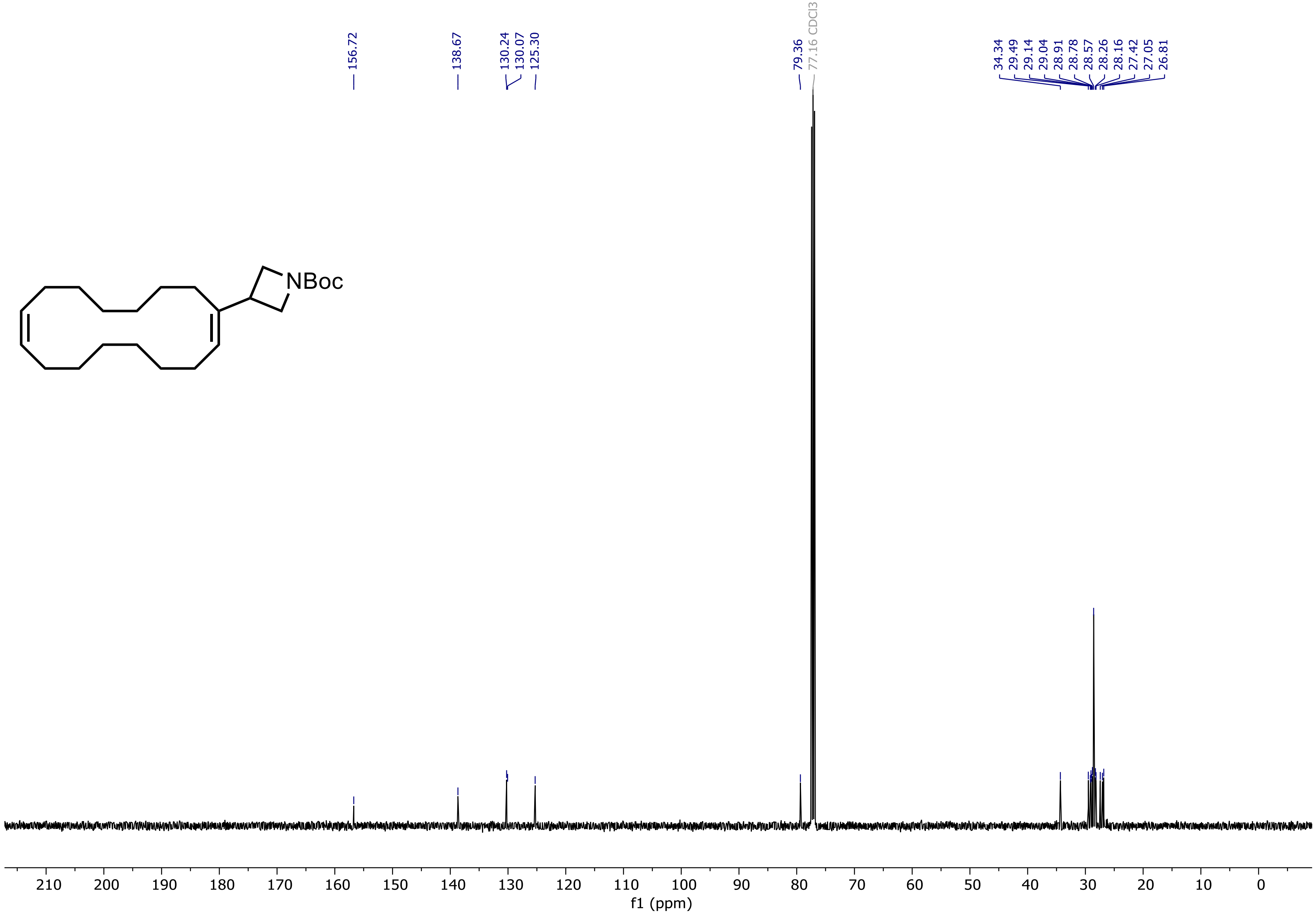

^{1}H NMR of tetrahydropyran-derived alkylated alkene 15

$CDCl_3$, 23°C

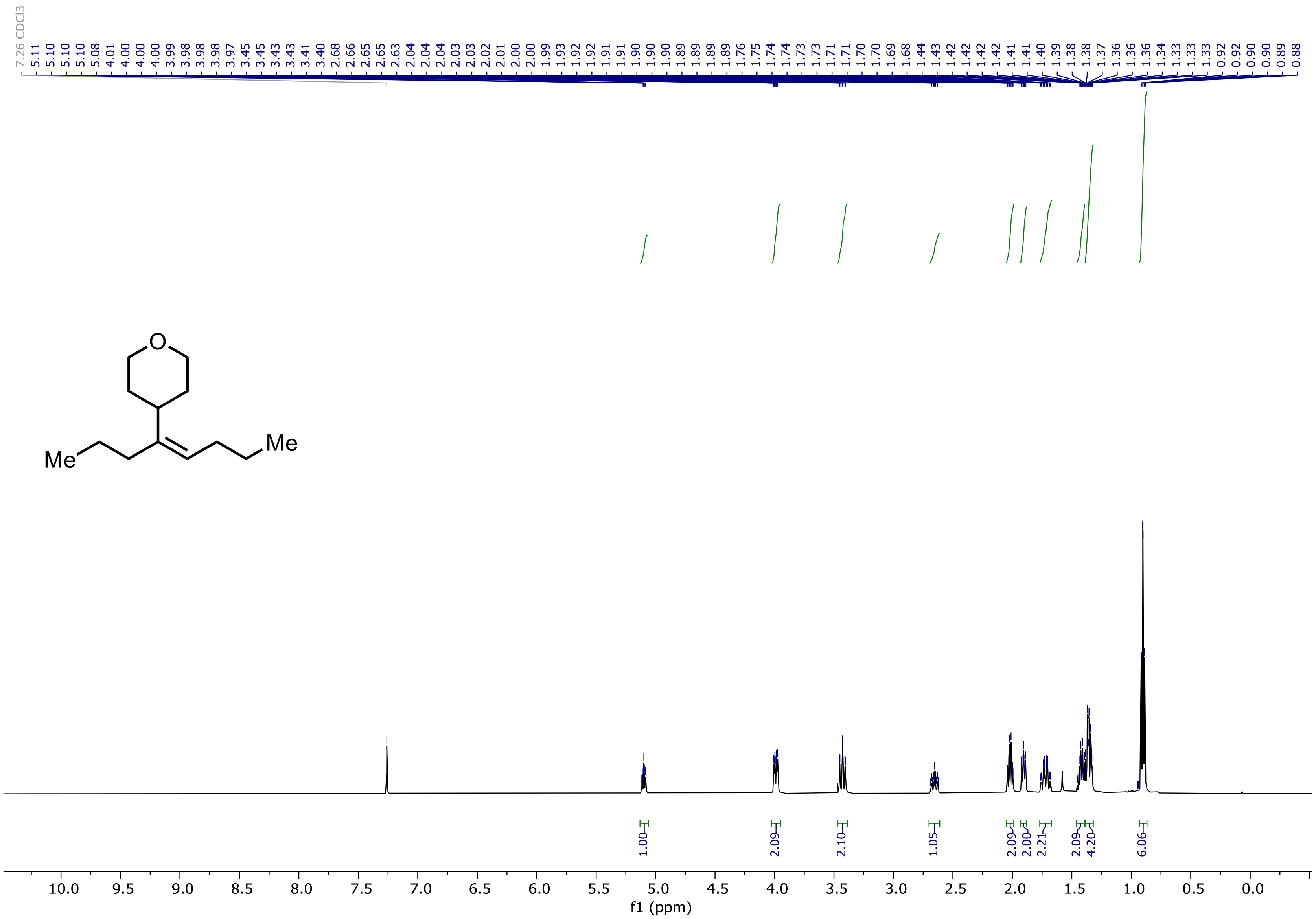

^{13}C NMR of tetrahydropyran-derived alkylated alkene 15

CDCl$_3$, 23°C

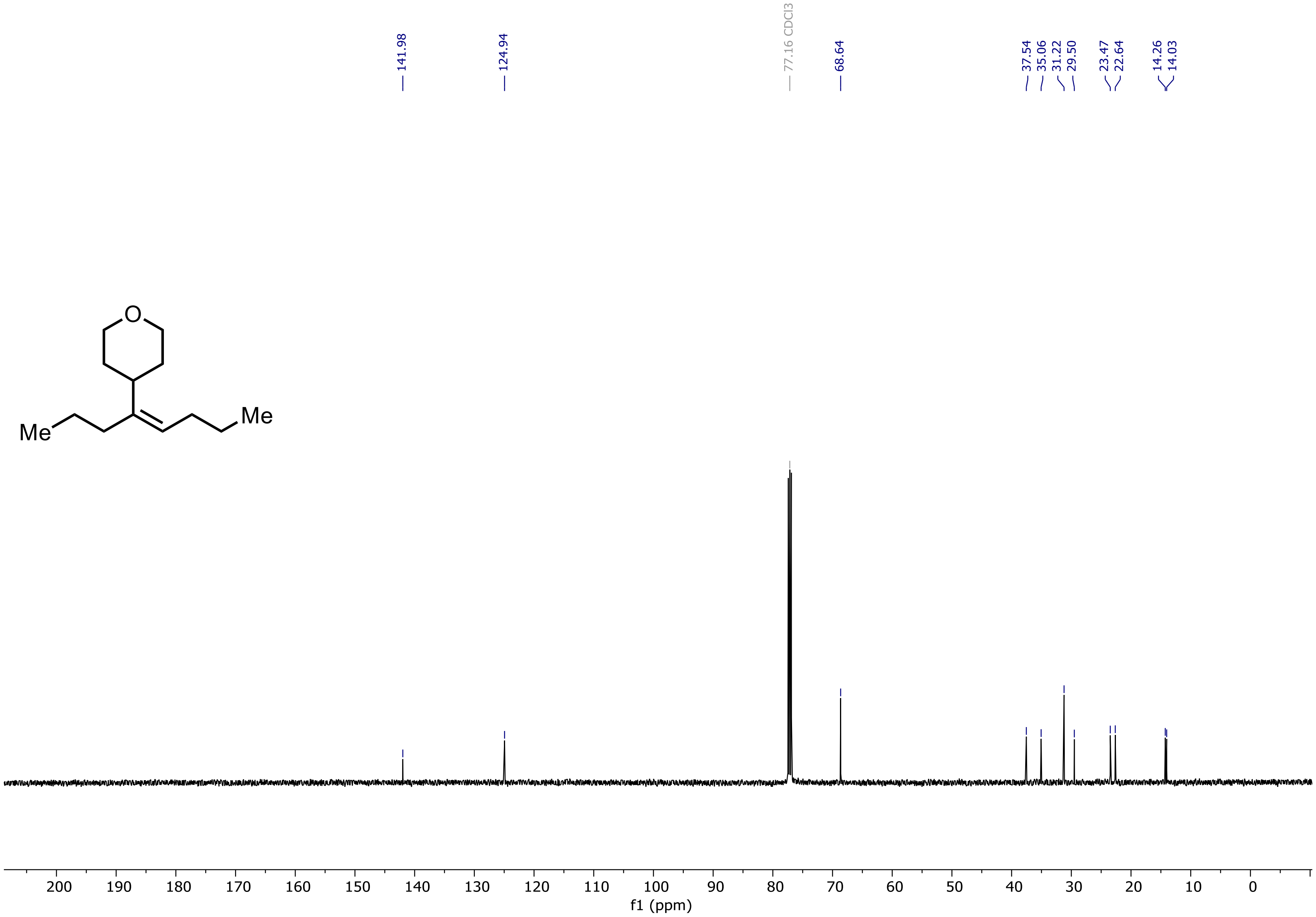

^{1}H NMR of 1,1-difluorocyclohexane-derived alkylated alkene 16

$CDCl_3$, 23°C

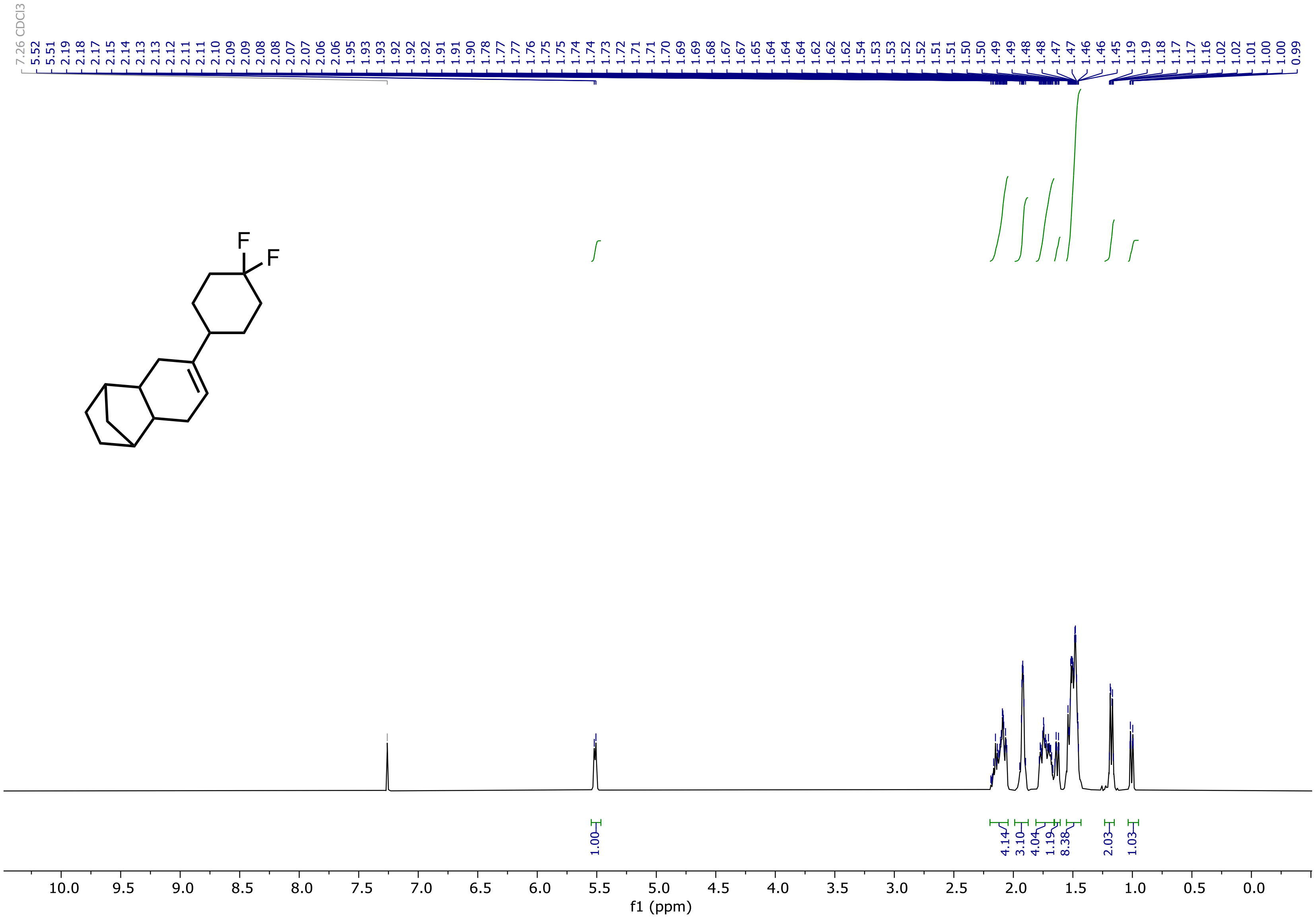

^{13}C NMR of 1,1-difluorocyclohexane-derived alkylated alkene 16

$CDCl_3$, 23°C

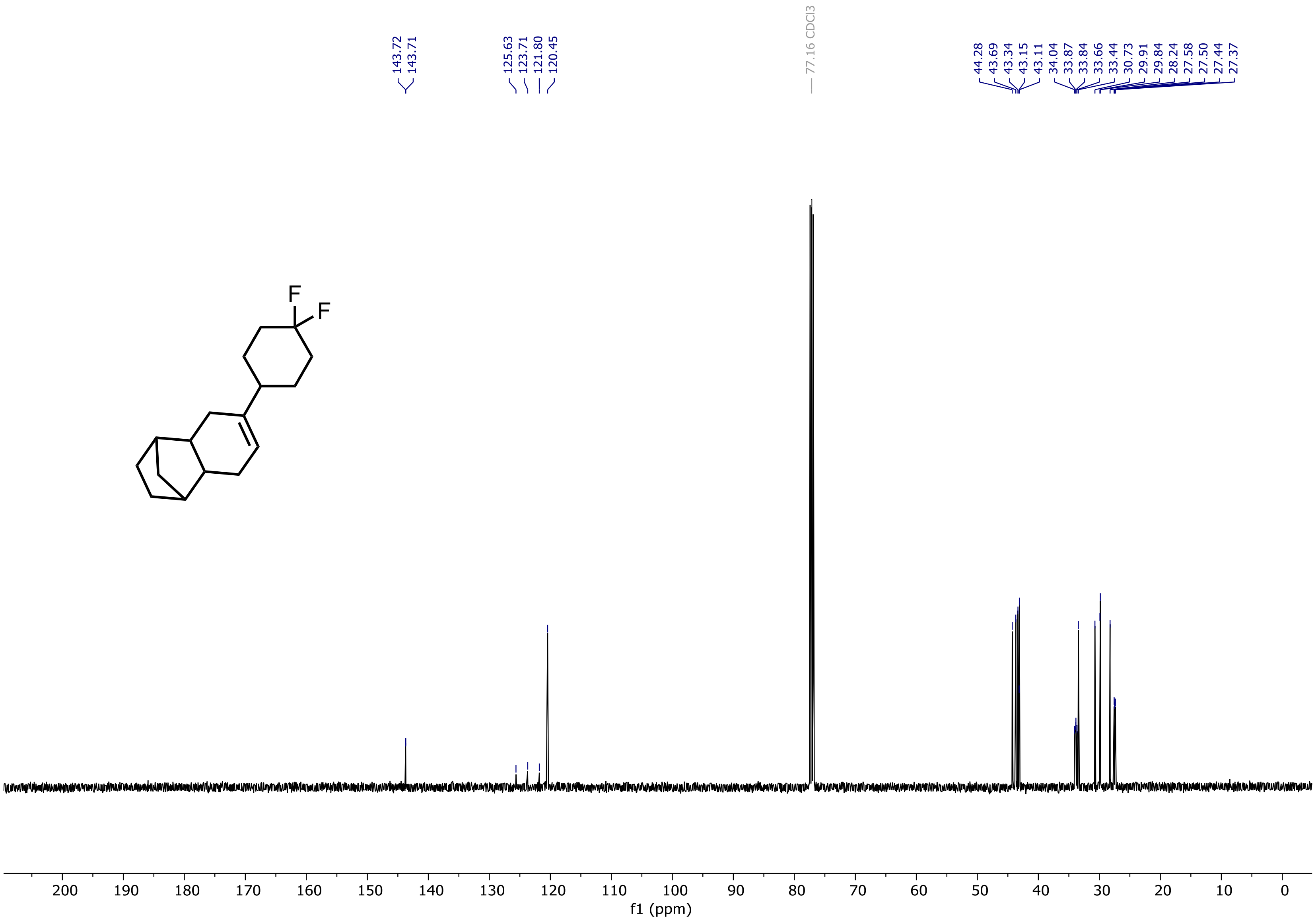

^{19}F NMR of 1,1-difluorocyclohexane-derived alkylated alkene 16

CDCl$_3$, 23°C

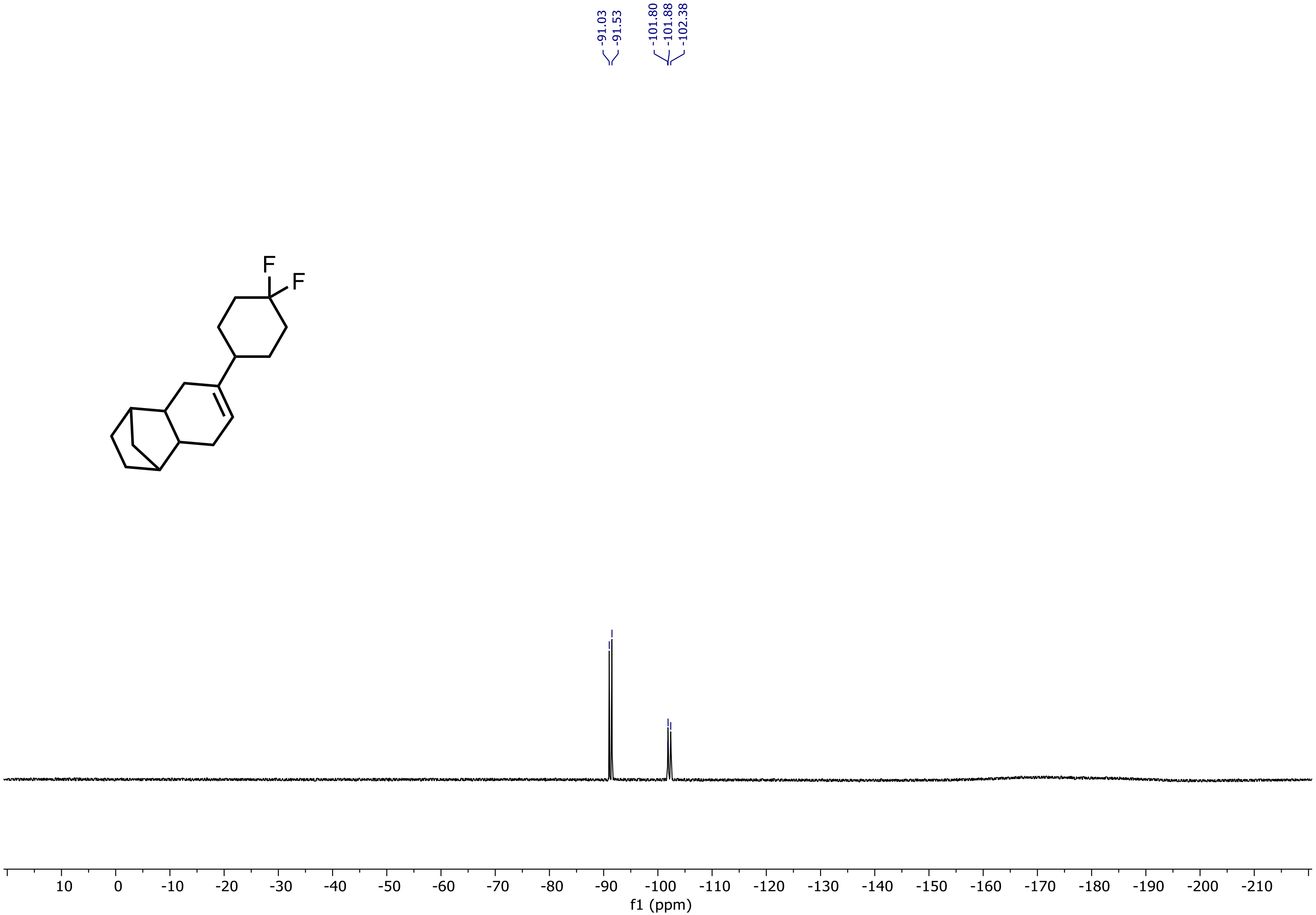

^{1}H NMR of 2,4-D-derived alkylated alkene 17

$CDCl_3$, 23°C

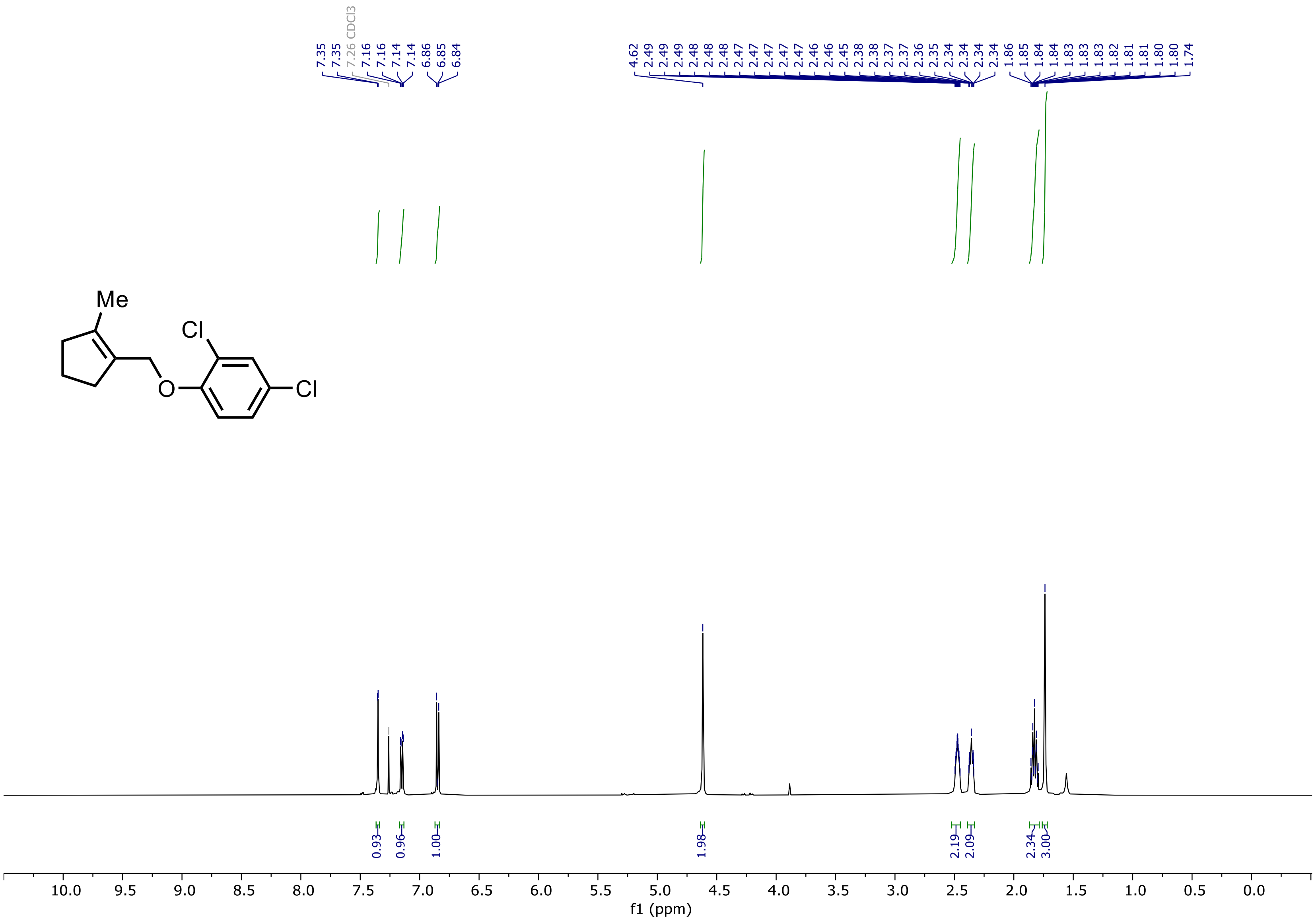

^{13}C NMR of 2,4-D-derived alkylated alkene 17

$CDCl_3$, 23°C

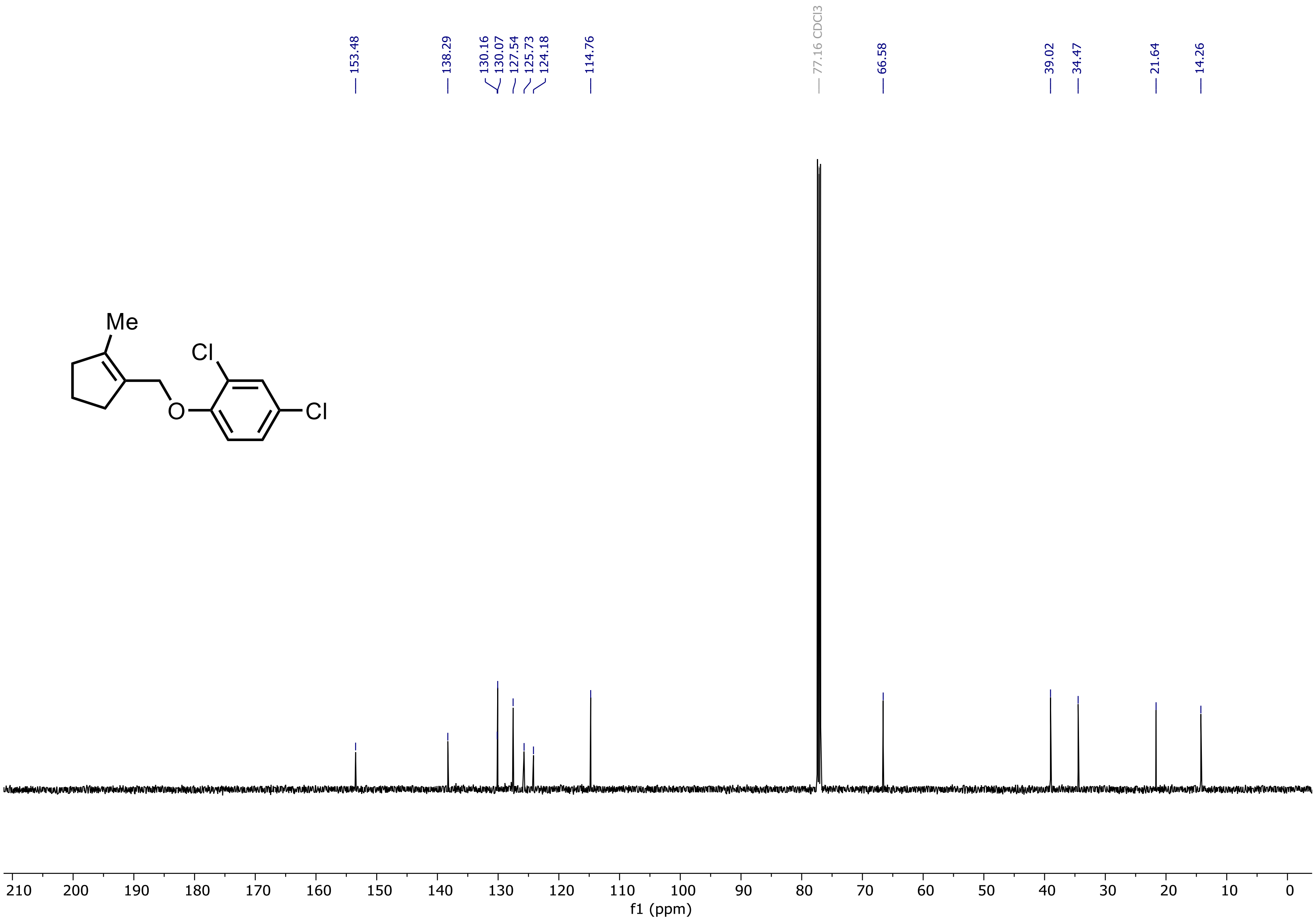

^{1}H NMR of methoxymethane-derived alkylated alkene 18

CDCl$_3$, 23°C

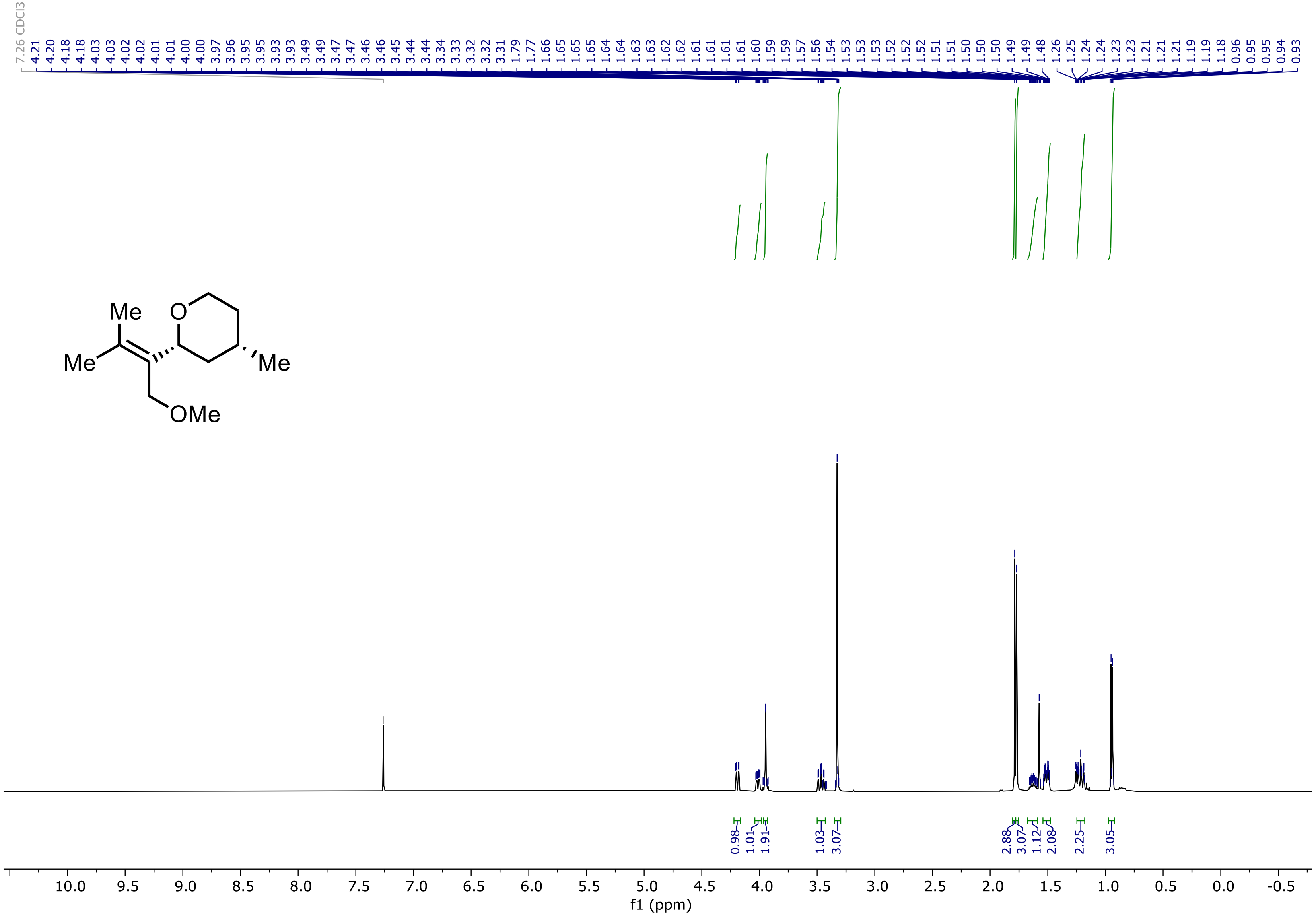

^{13}C NMR of methoxymethane-derived alkylated alkene 18

$CDCl_3$, 23°C

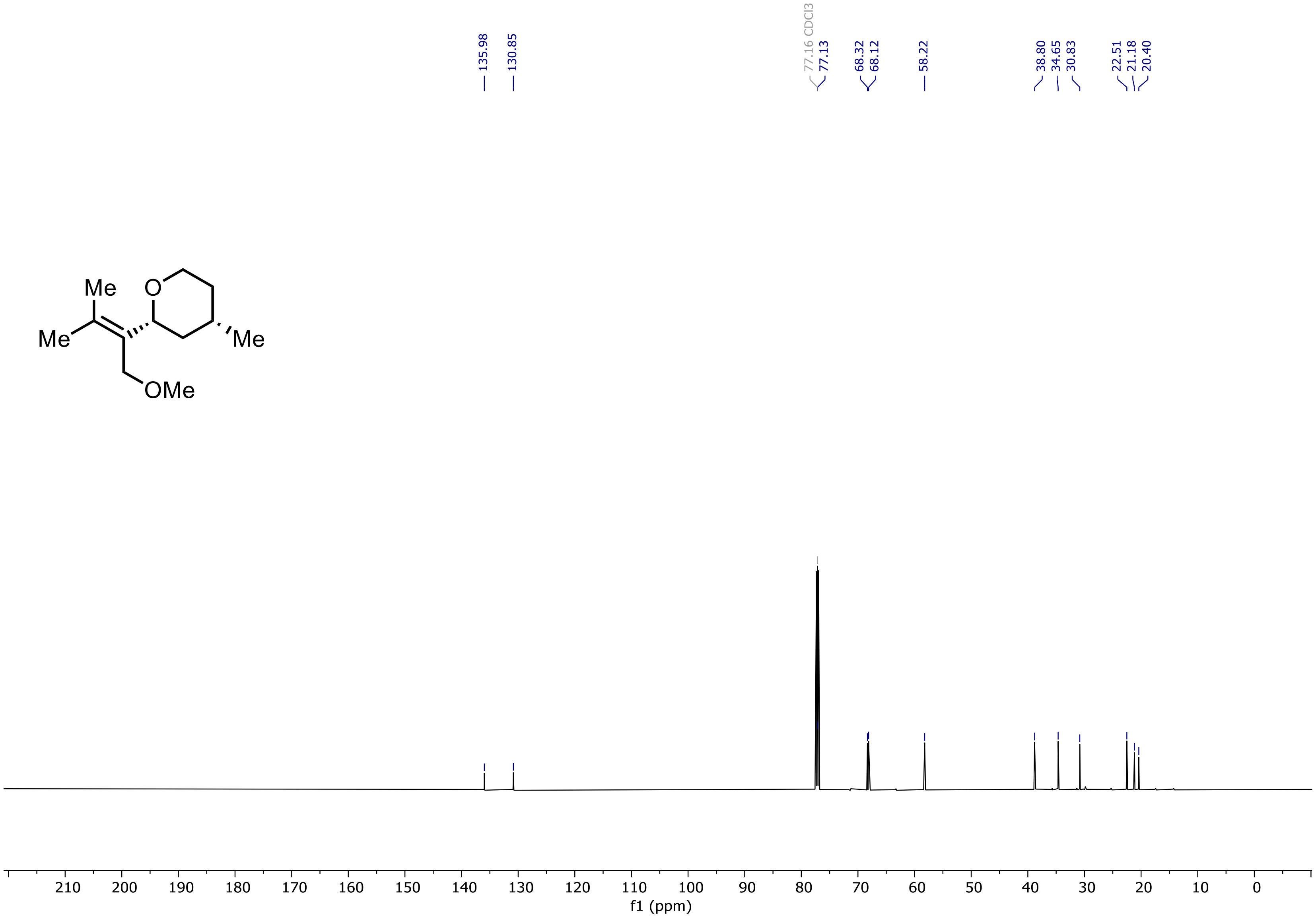

^{1}H NMR of (−)-Menthyloxyacetic acid derived alkylated alkene 19

$CDCl_3$, 23°C

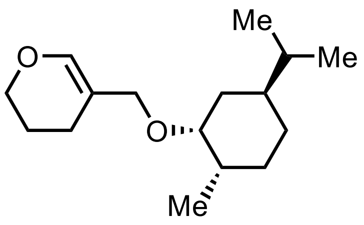

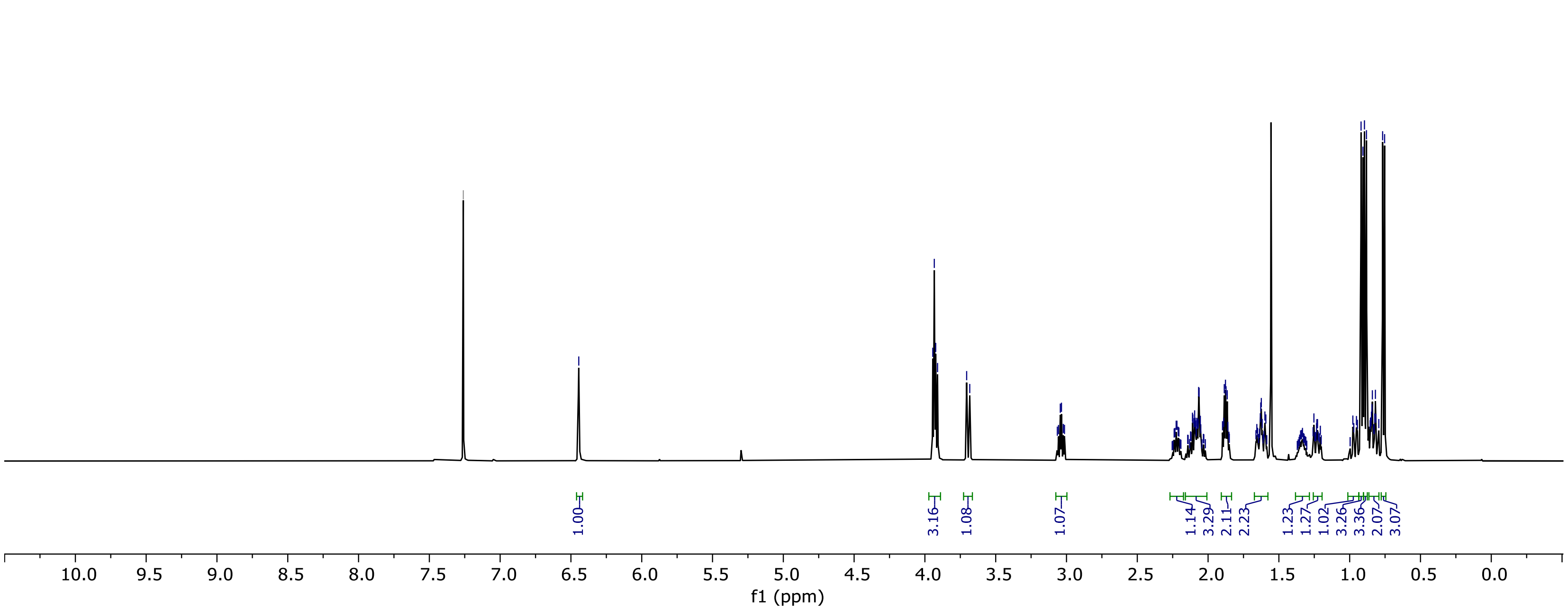

^{13}C NMR of (−)-Menthyloxyacetic acid derived alkylated alkene 19

$CDCl_3$, 23°C

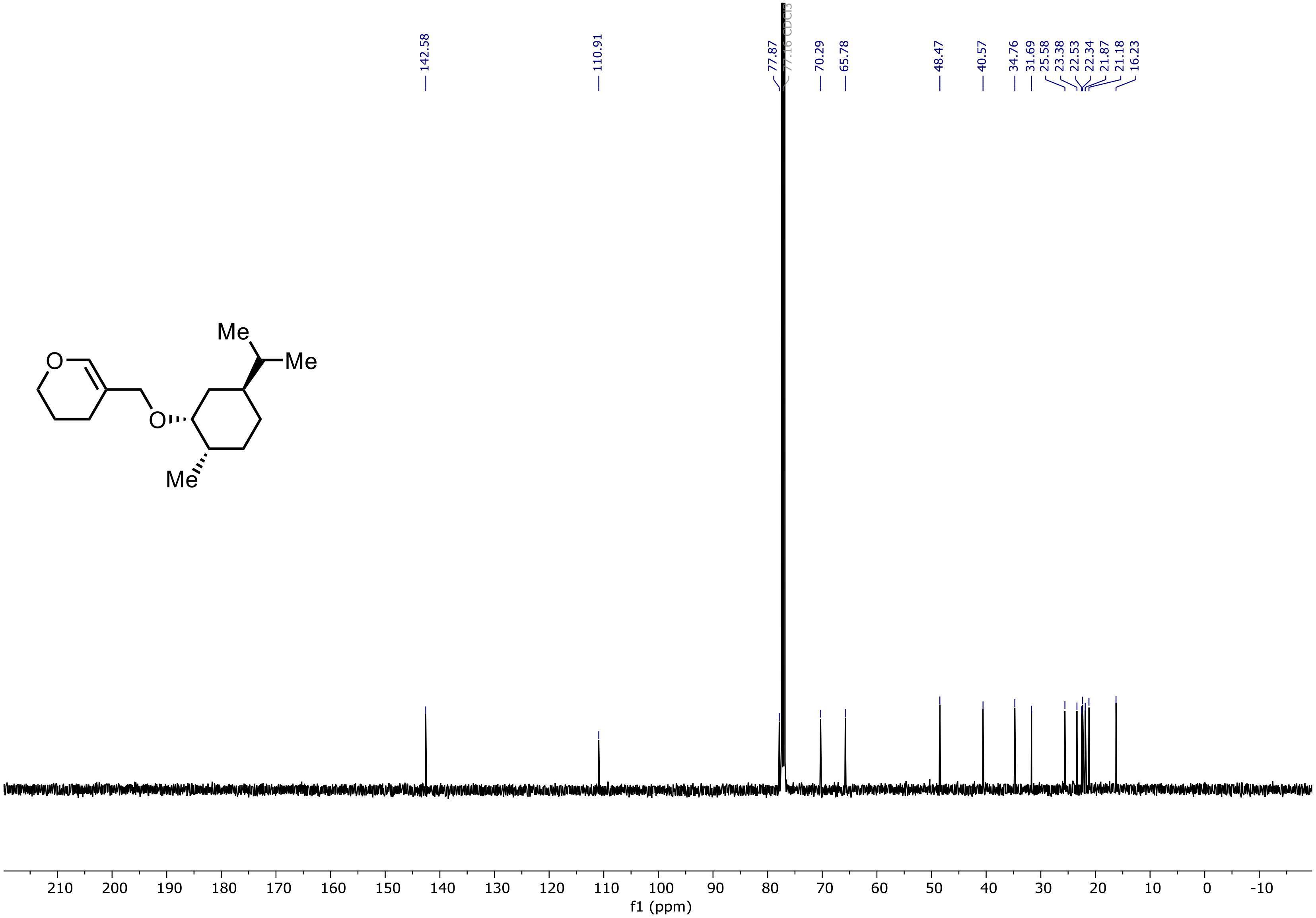

^{1}H NMR of methoxyethane derived alkylated alkene 20

$CDCl_3$, 23°C

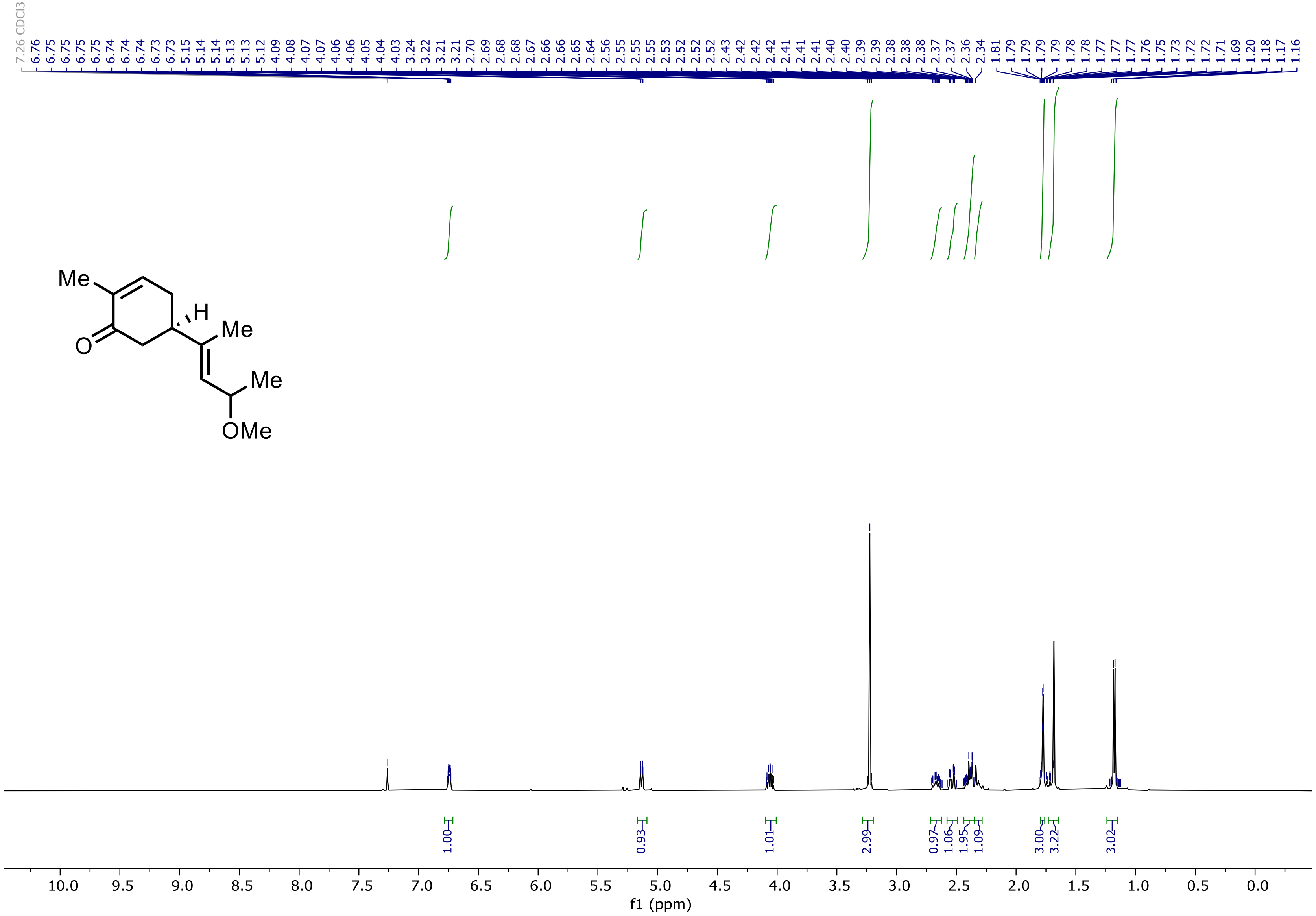

^{13}C NMR of methoxyethane derived alkylated alkene 20

$CDCl_3$, 23°C

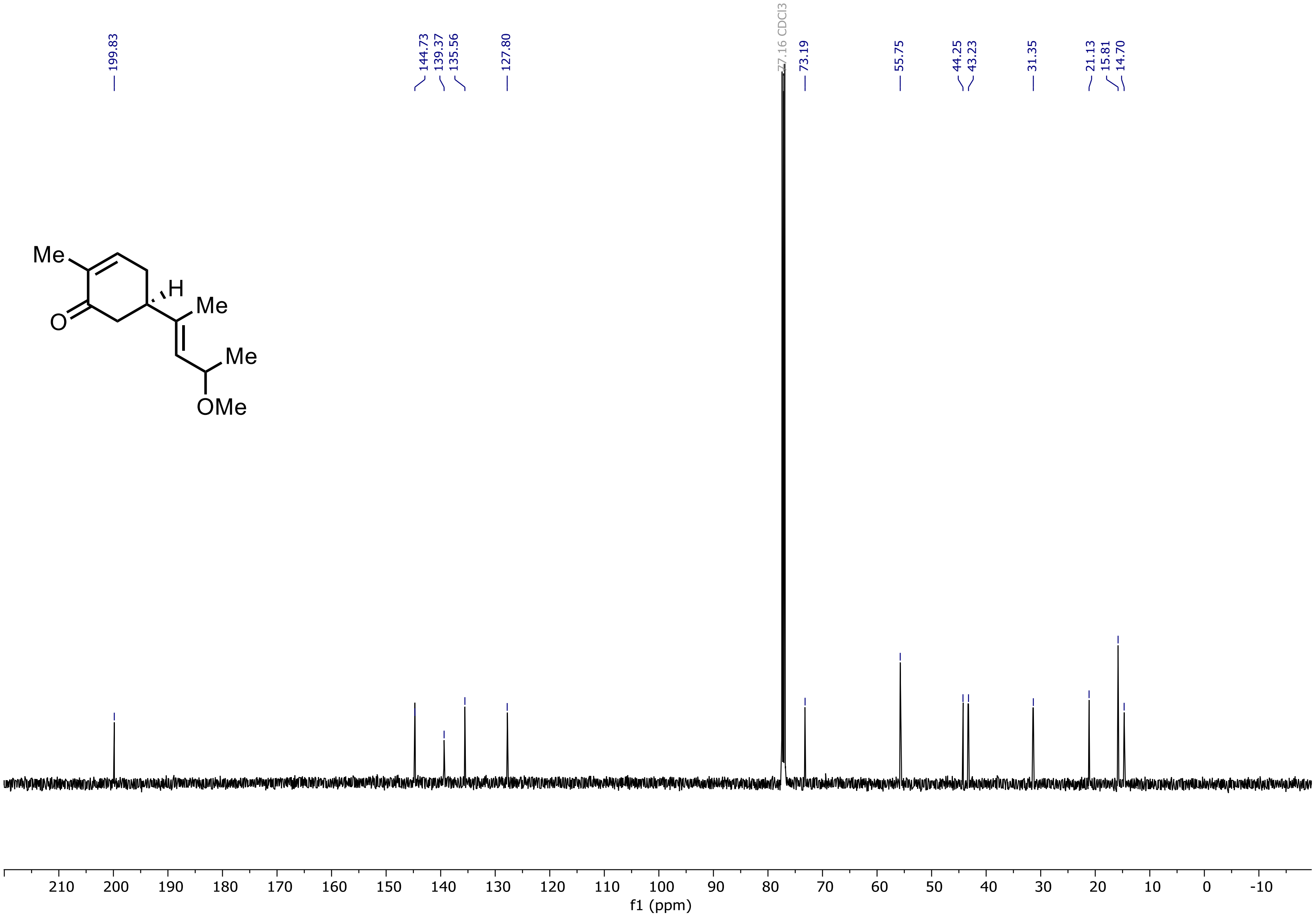

^{1}H NMR of *tert*-butyl-propionate-derived alkylated alkene 21

CDCl$_3$, 23°C

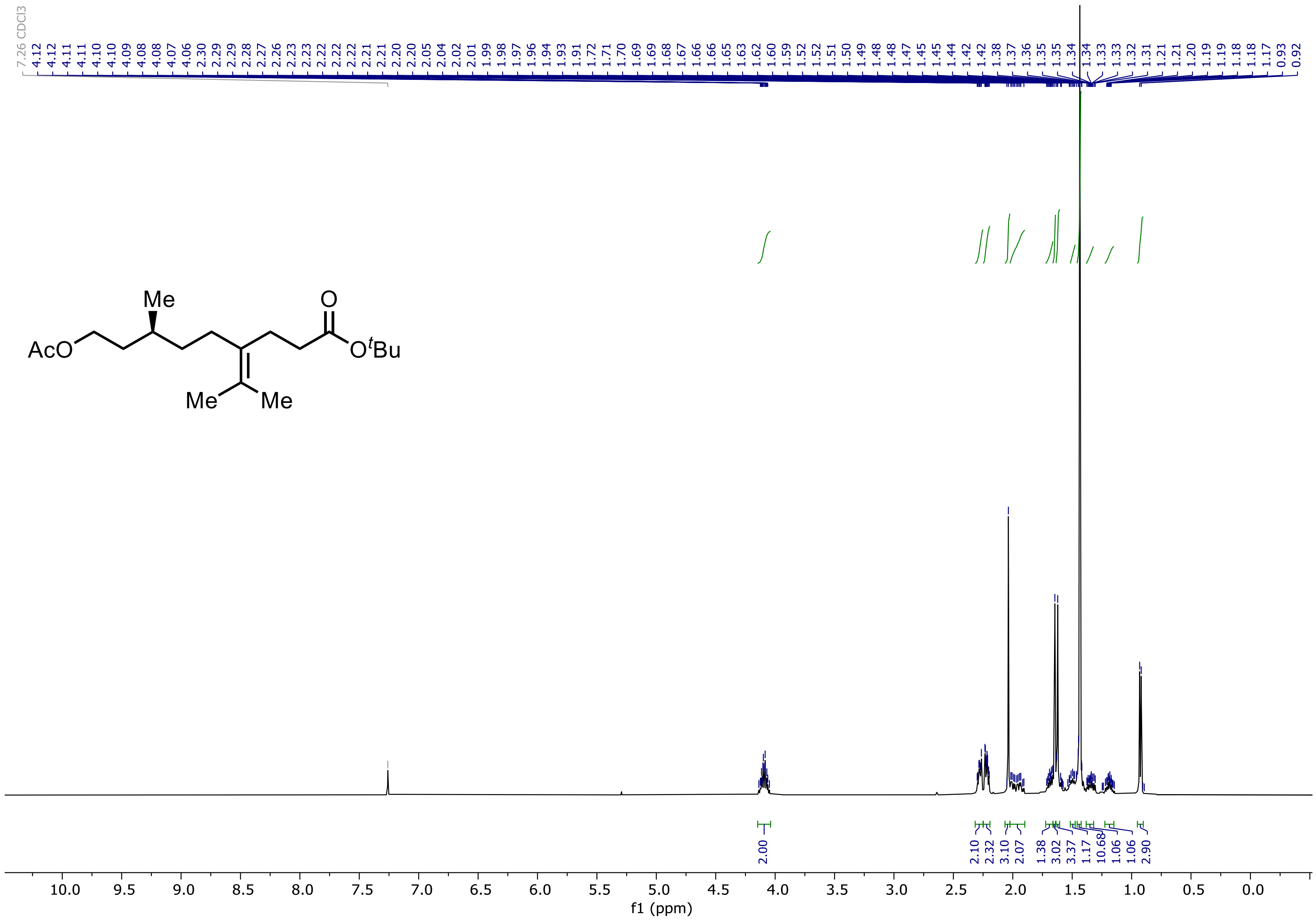

^{13}C NMR *tert*-butyl-propionate-derived of alkylated alkene 21

CDCl$_3$, 23°C

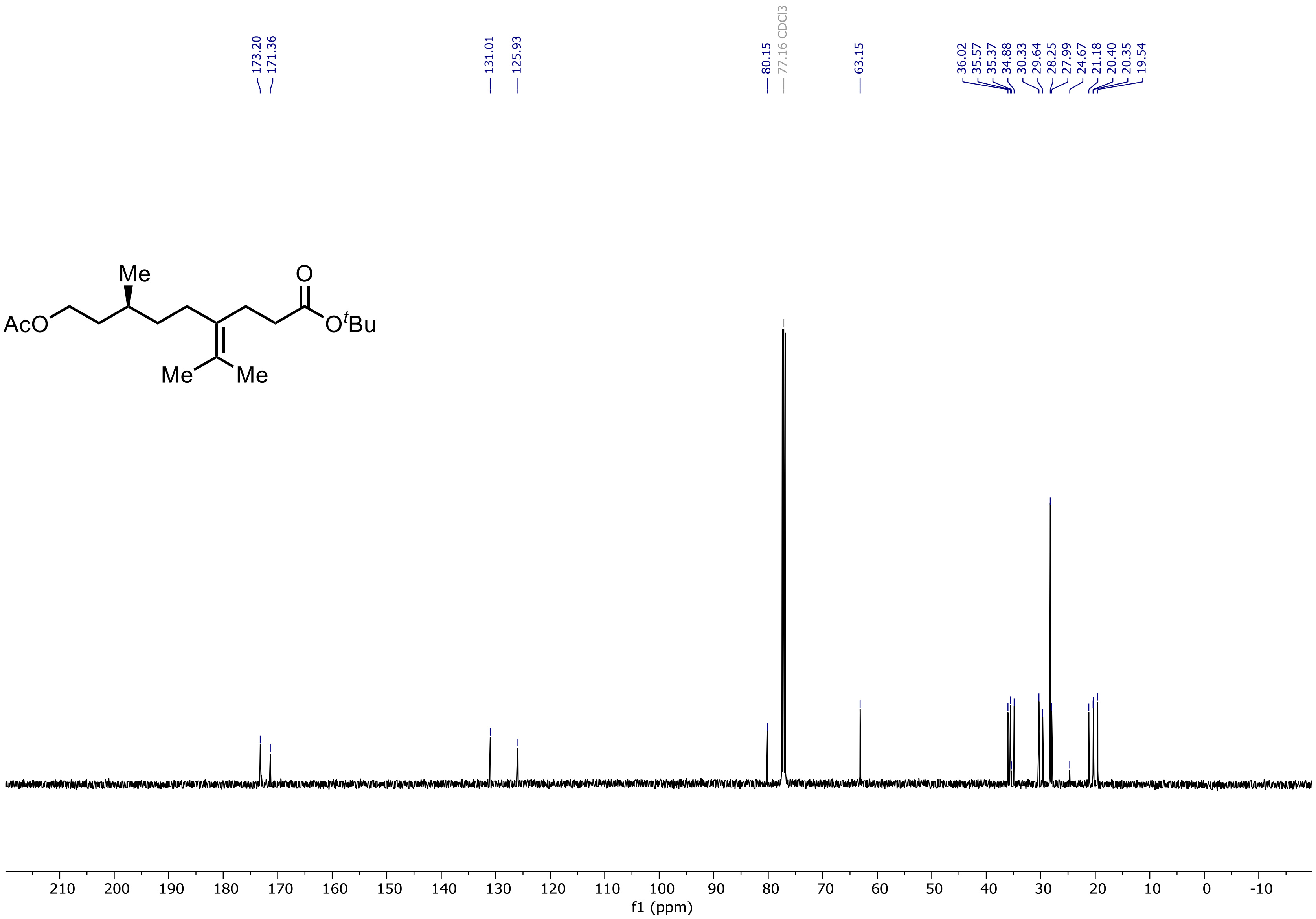

^{1}H NMR of γ-aminobutyric acid-derived alkylated alkene 22

CDCl$_3$, 23°C

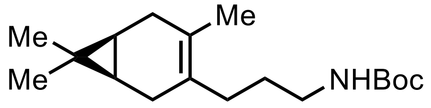

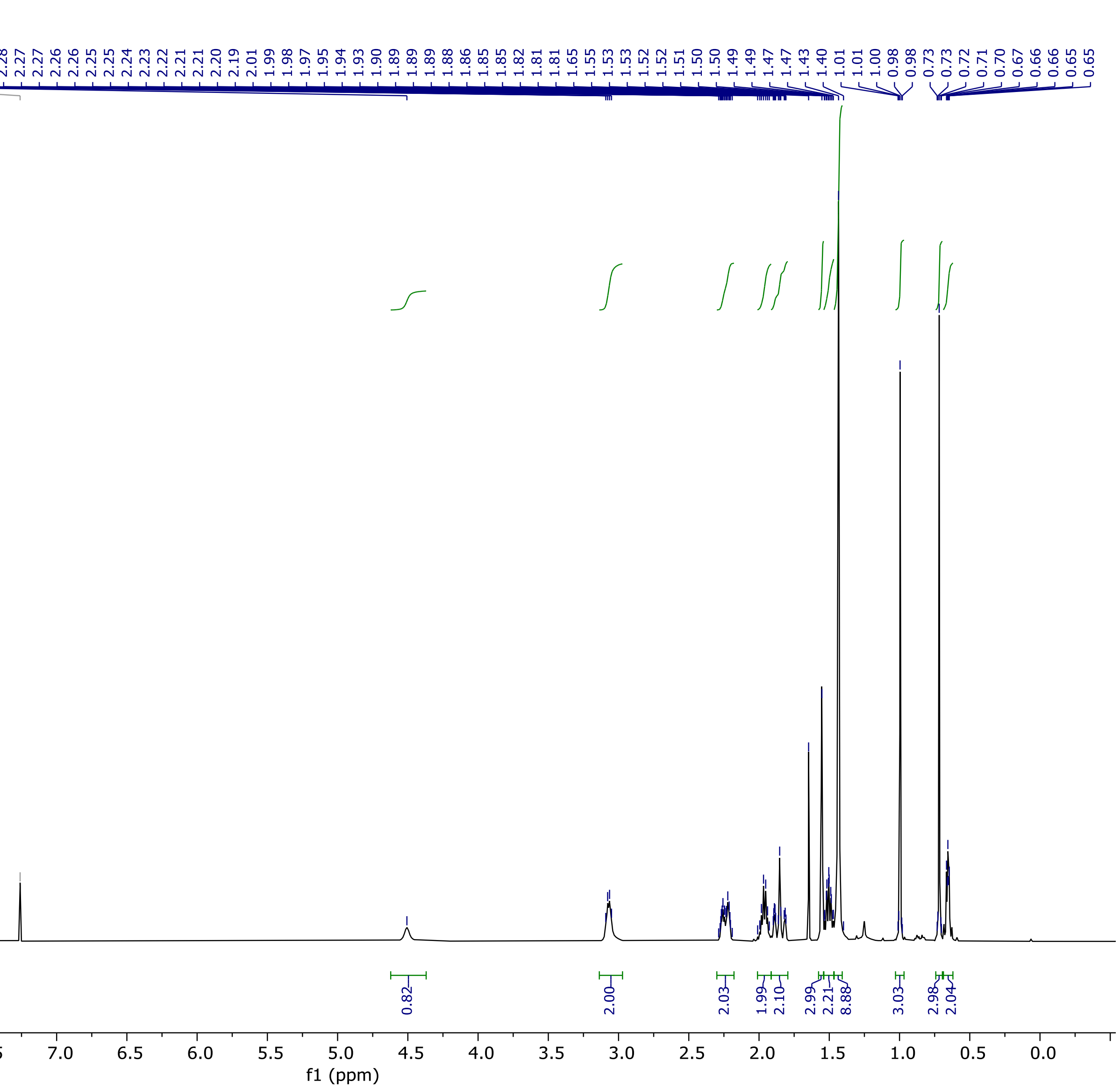

^{13}C NMR of γ-aminobutyric acid-derived alkylated alkene 22

CDCl3, 23°C

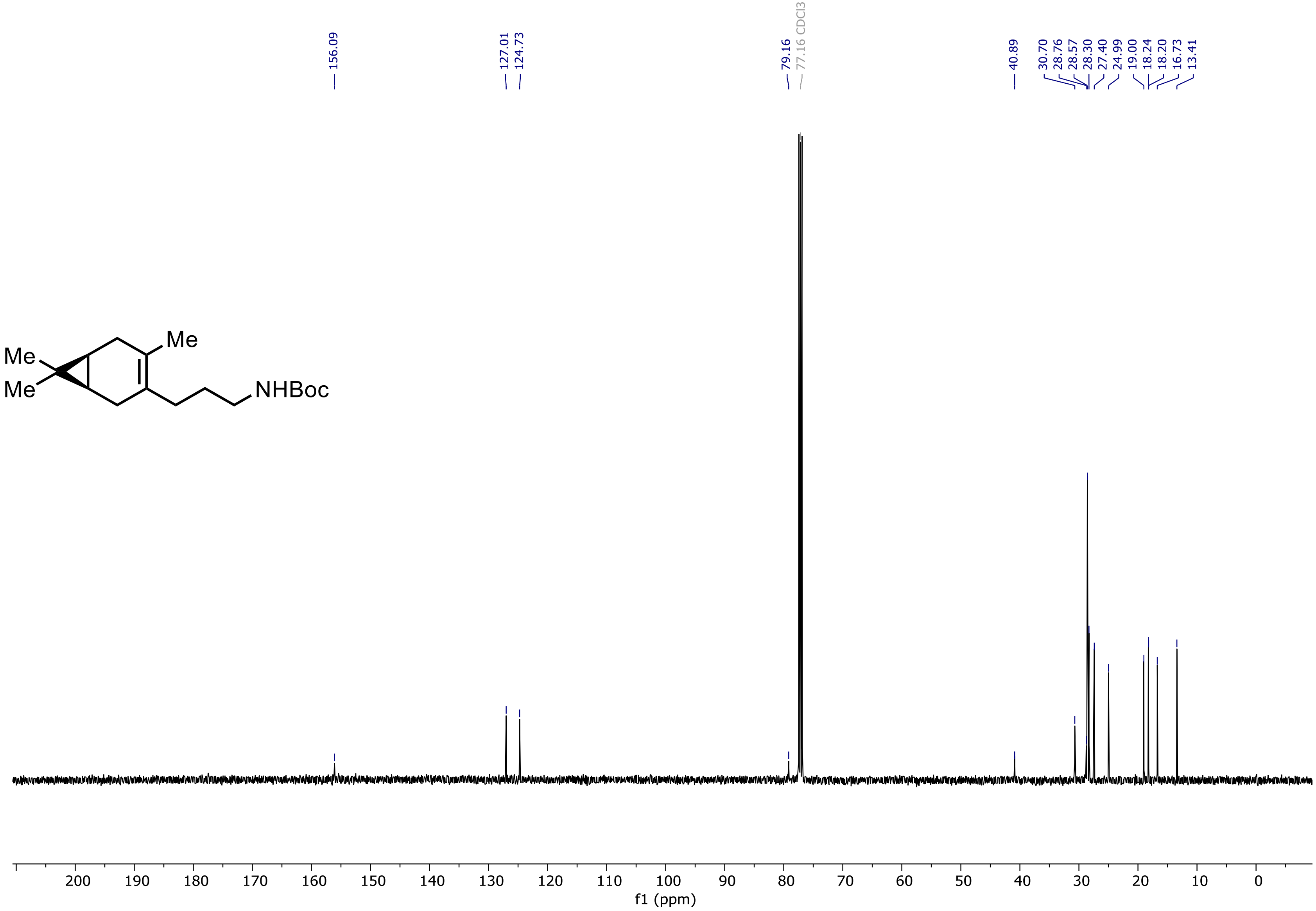

^{1}H NMR of 1,1-difluorocyclobutane derived alkylated alkene 23

CDCl$_3$, 23°C

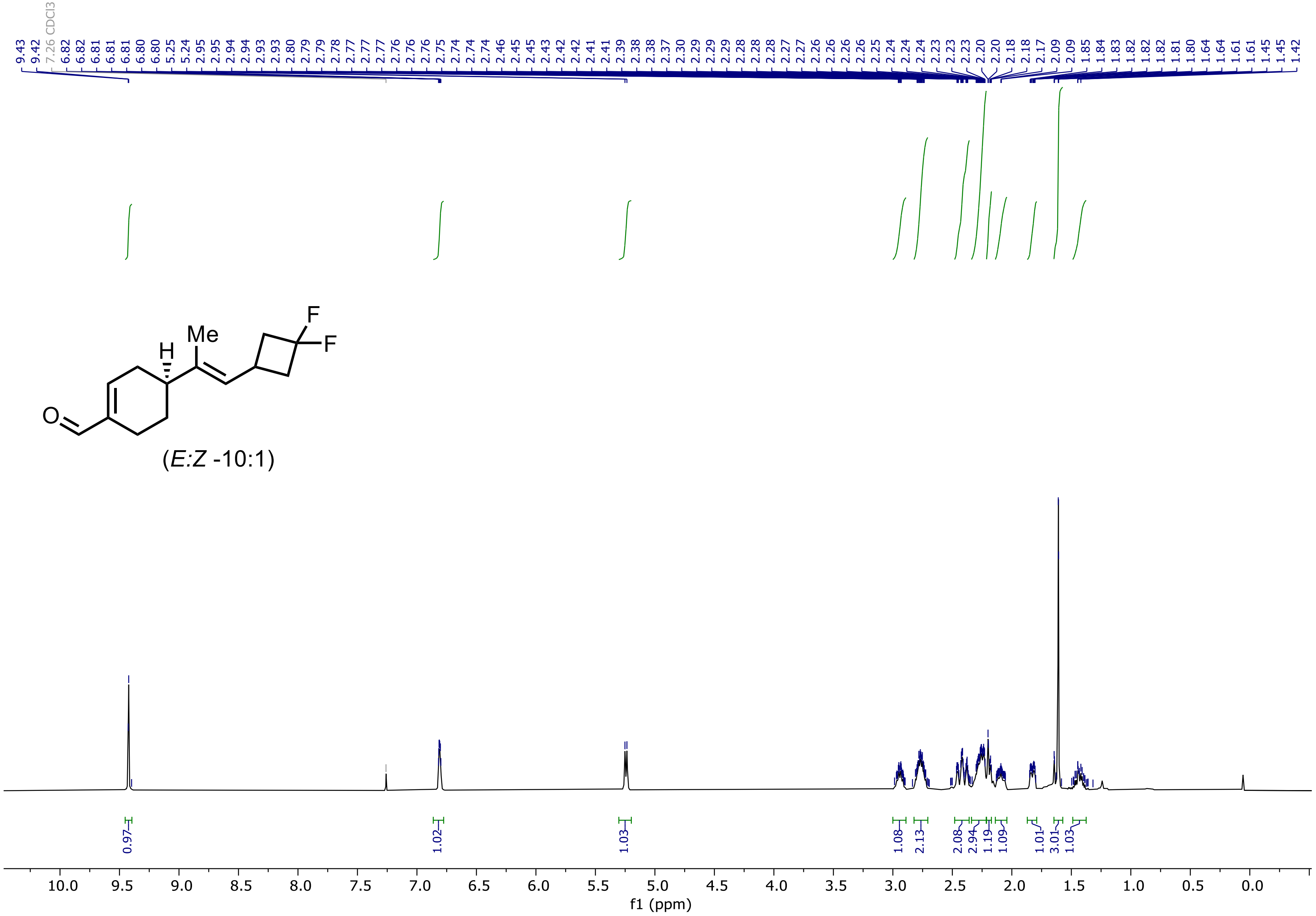

^{13}C NMR of 1,1-difluorocyclobutane derived alkylated alkene 23

$CDCl_3$, 23°C

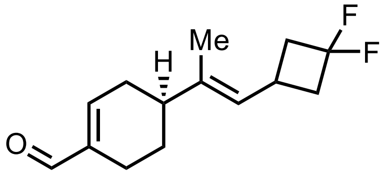

(*E*:*Z* -10:1)

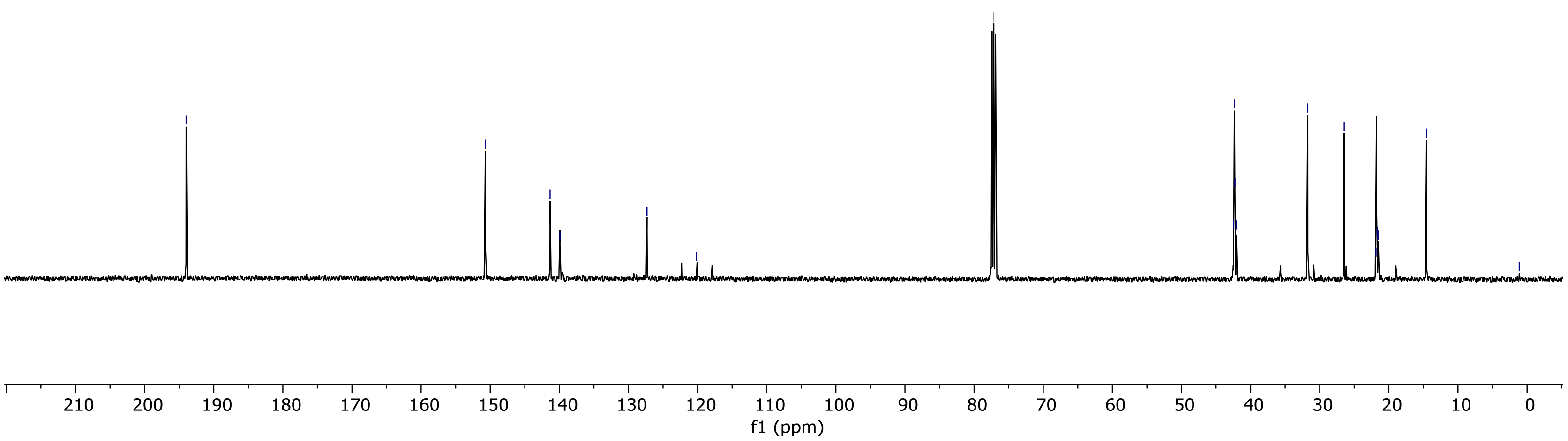

^{19}F NMR of 1,1-difluorocyclobutane derived alkylated alkene 23

$CDCl_3$, 23°C

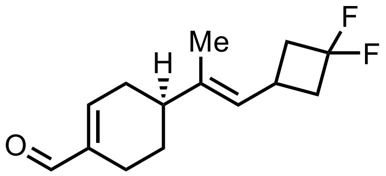

(*E*:*Z* -10:1)

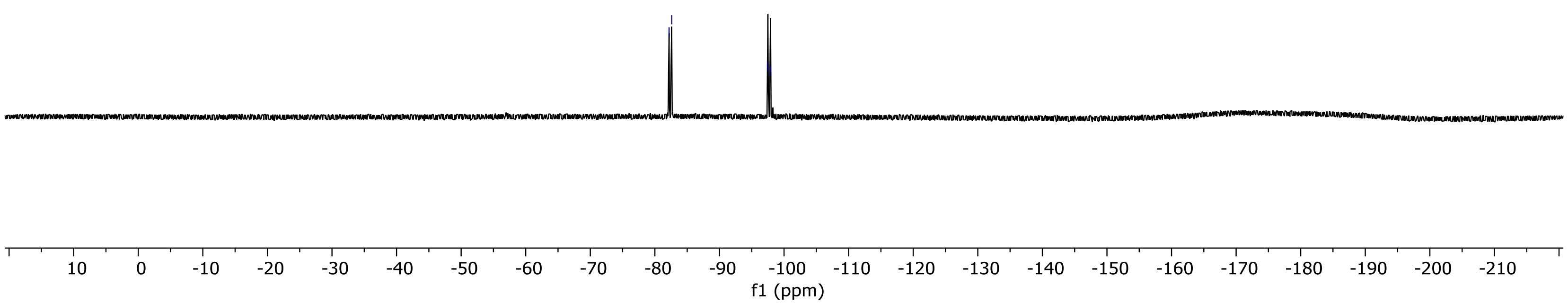

^{1}H NMR of 1-ethyl-3,5-dichlorobenzene derived alkylated alkene 24

$CDCl_3$, 23°C

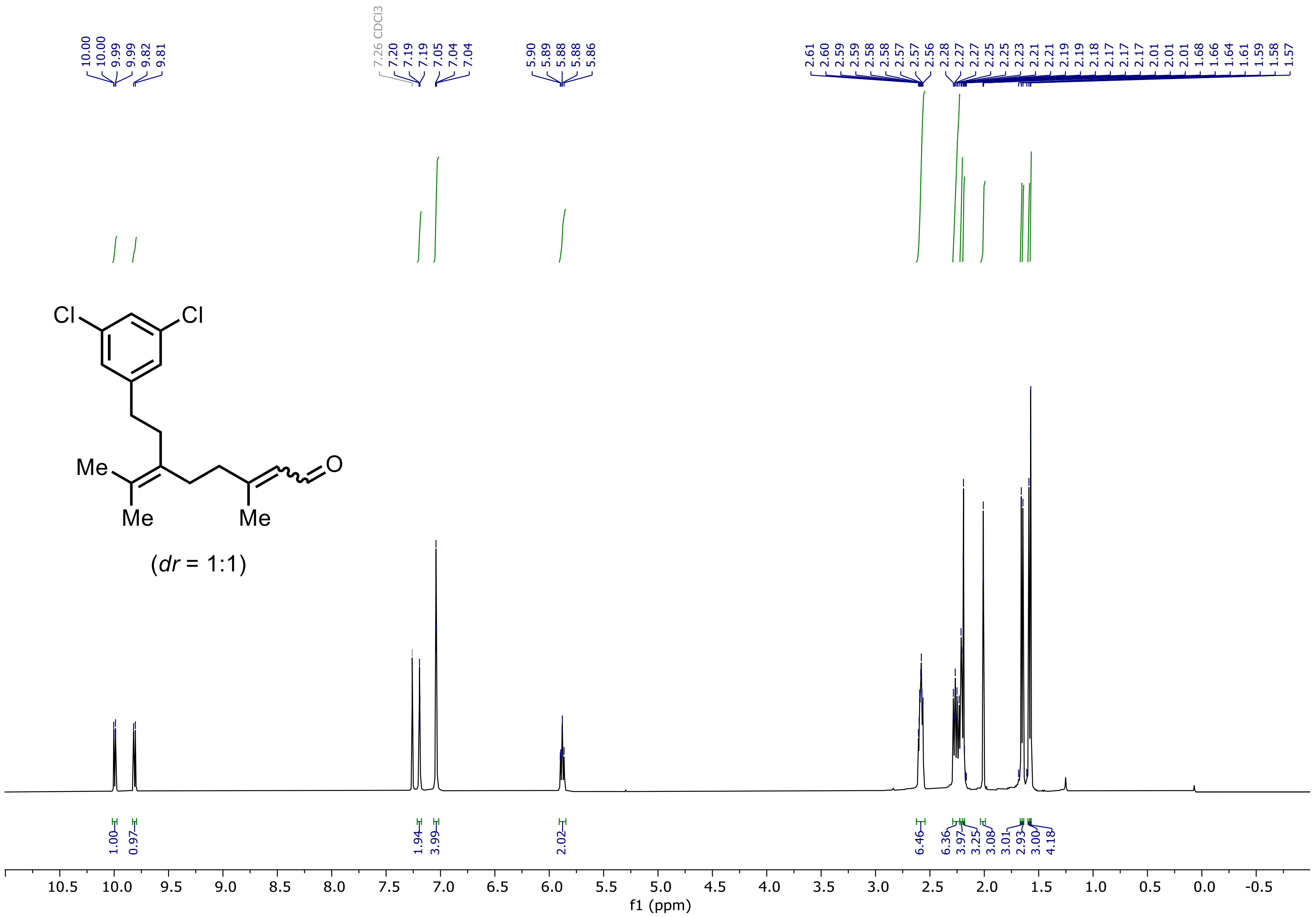

^{13}C NMR of 1-ethyl-3,5-dichlorobenzene derived alkylated alkene 24

$CDCl_3$, 23°C

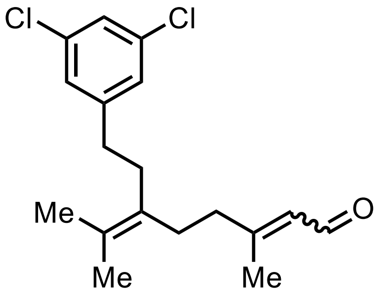

(*dr* = 1:1)

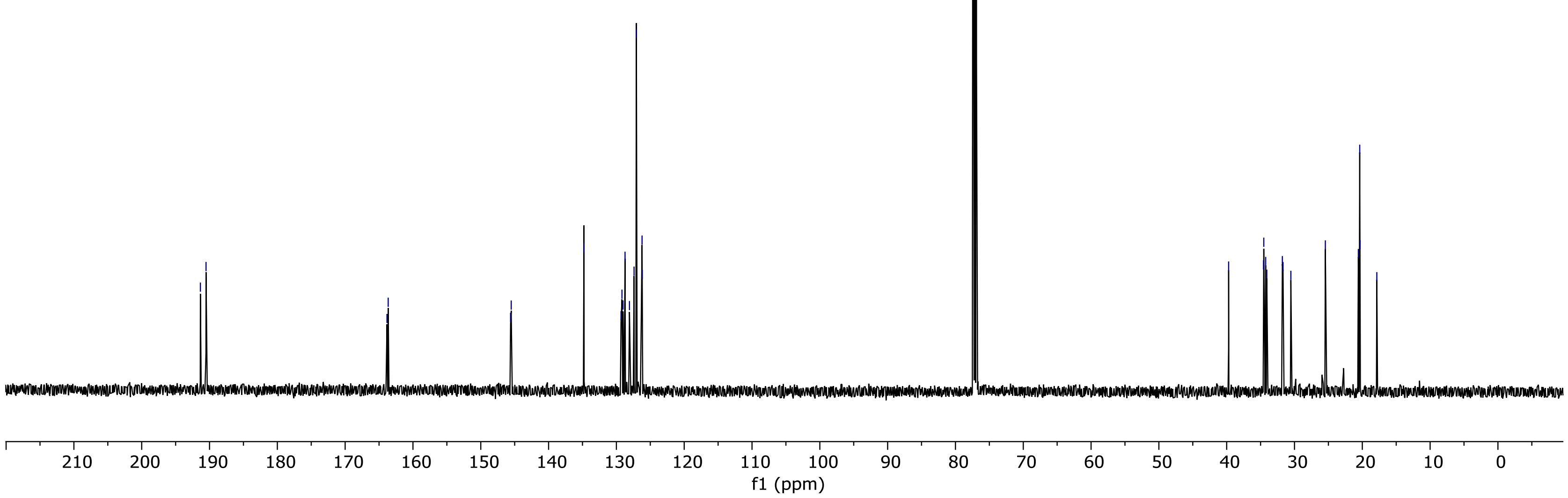

^{1}H NMR of baclofen derived alkylated alkene (±)-25

$CDCl_3$, 23°C

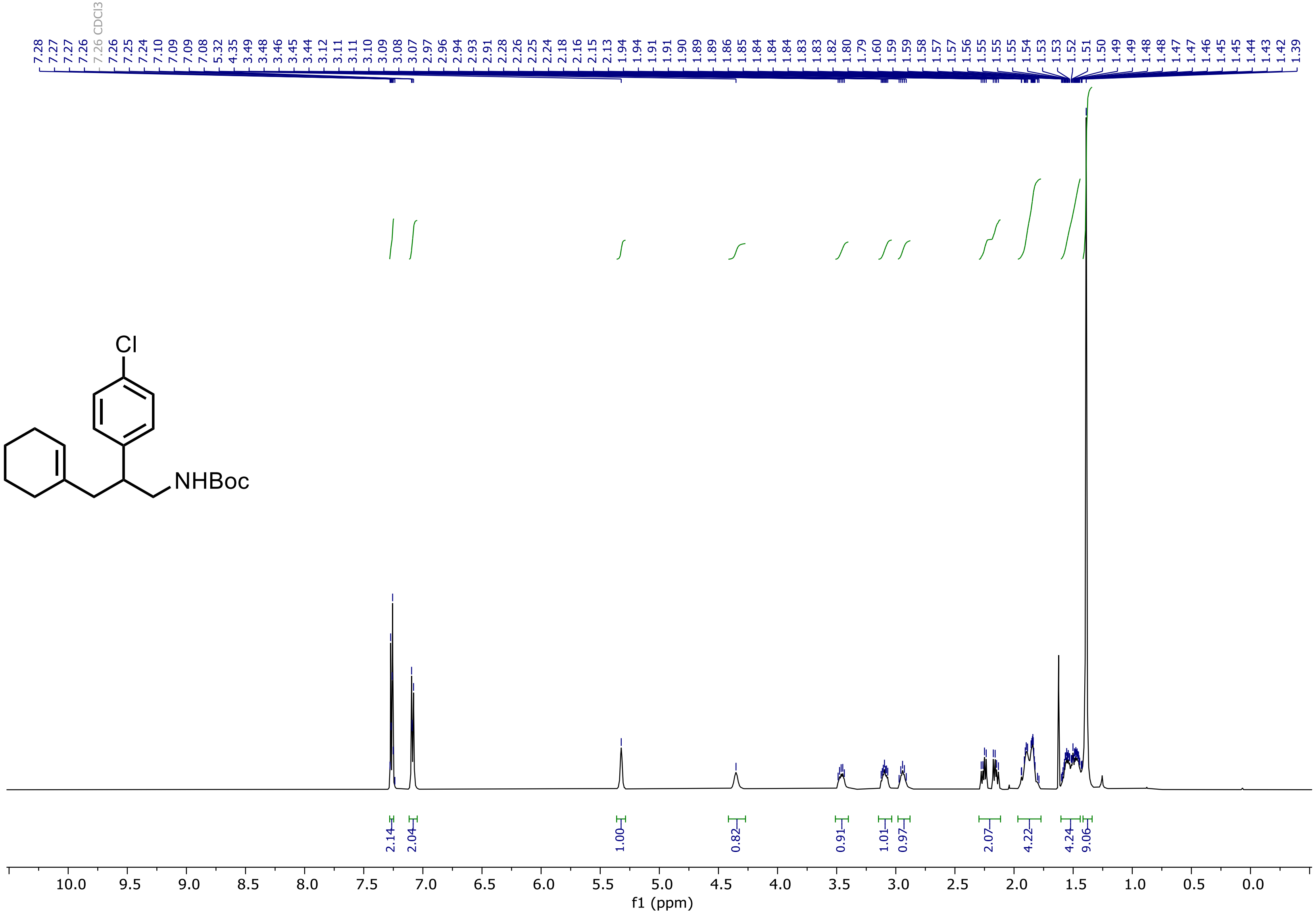

^{13}C NMR of baclofen derived alkylated alkene (±)-25

$CDCl_3$, 23°C

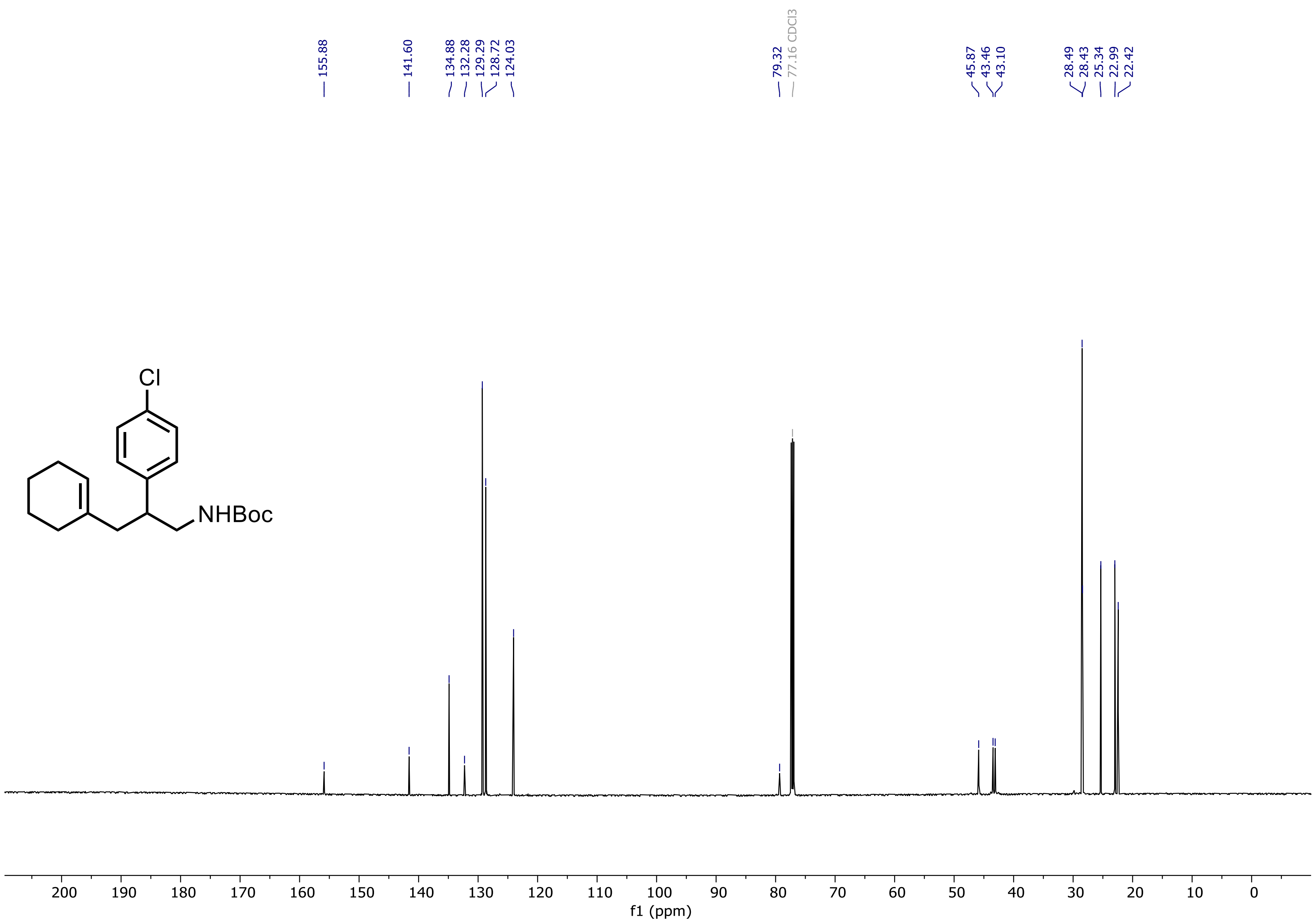

^{1}H NMR of iodide compound 26

CDCl₃, 23°C

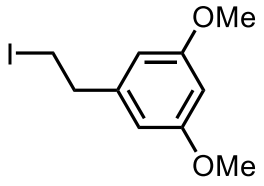

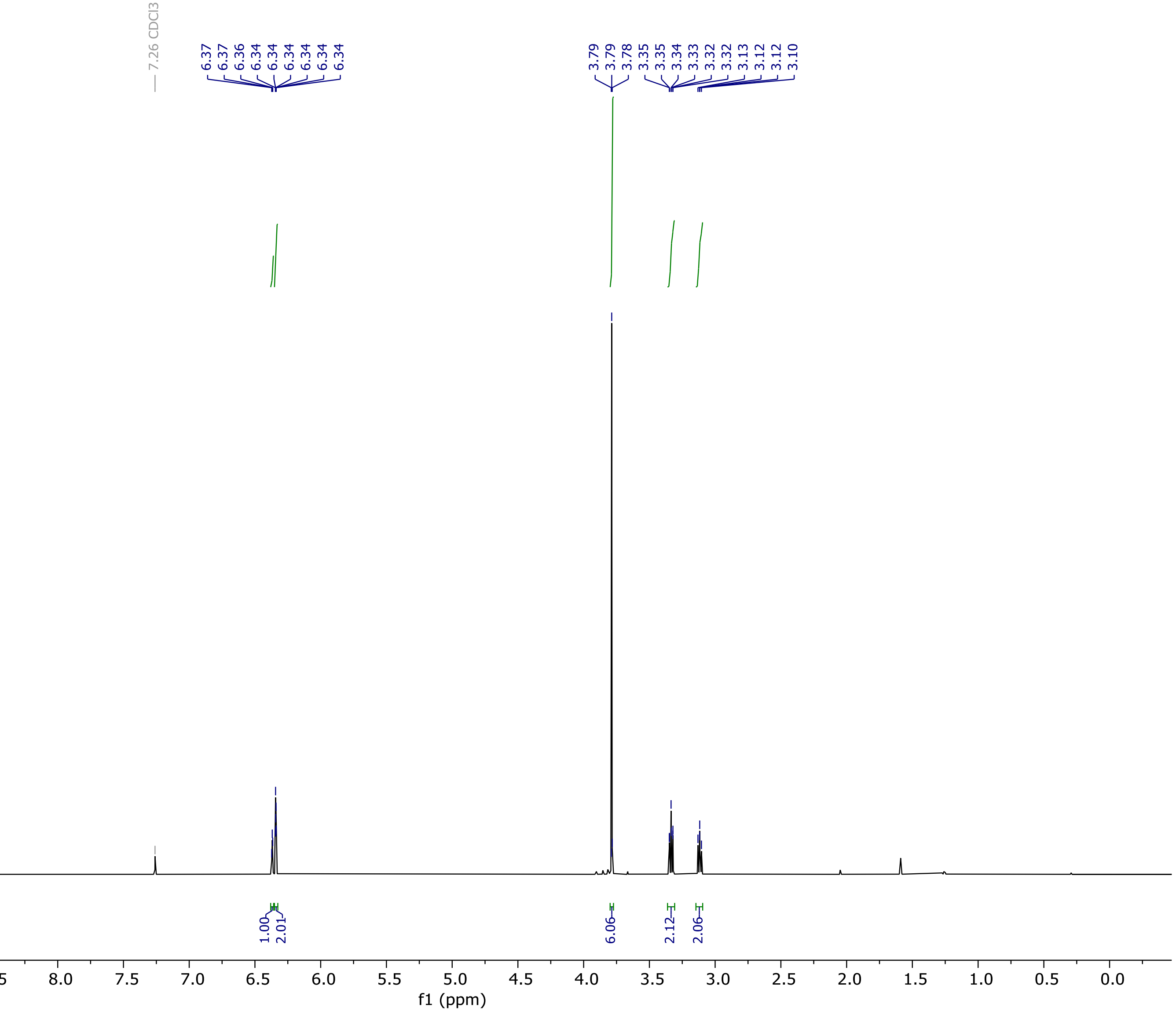

^{13}C NMR of iodide compound 26

$CDCl_3$, 23°C

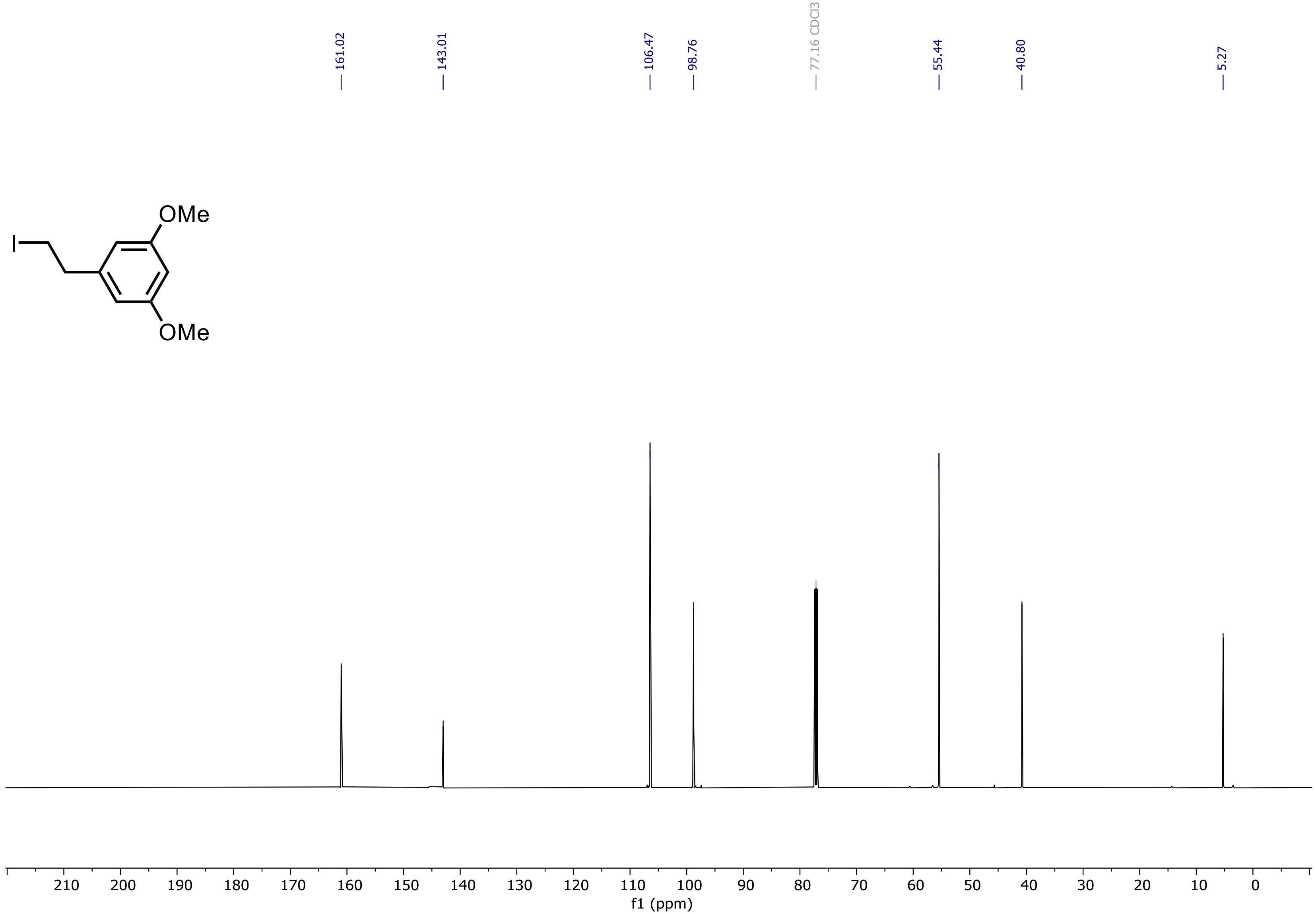

^{1}H NMR of *N-tert*-butyloxycarbonyl-azetidine derived alkylated alkene 27

$CDCl_3$, 23°C

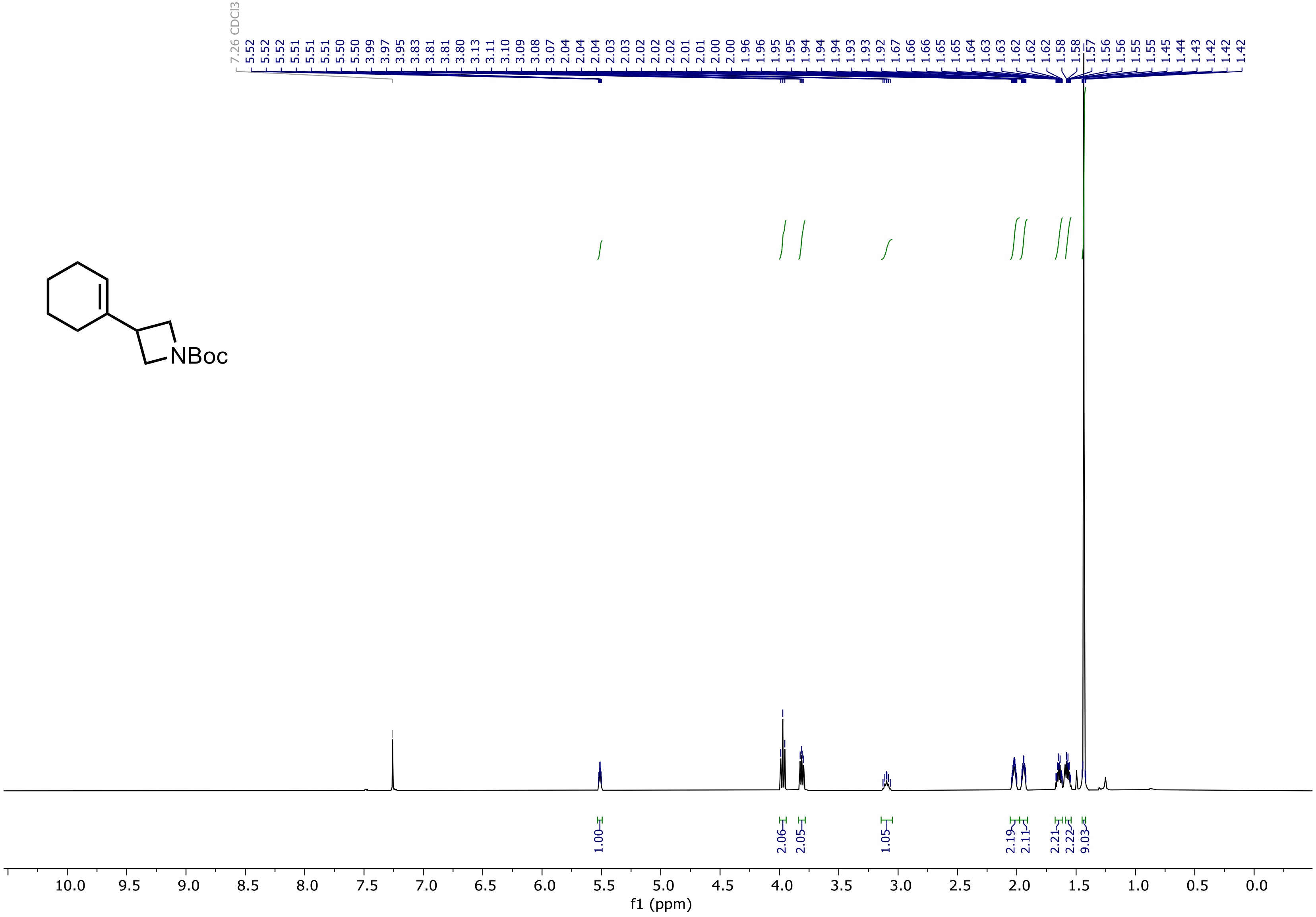

^{13}C NMR of *N-tert*-butyloxycarbonyl-azetidine derived alkylated alkene 27

CDCl$_3$, 23°C

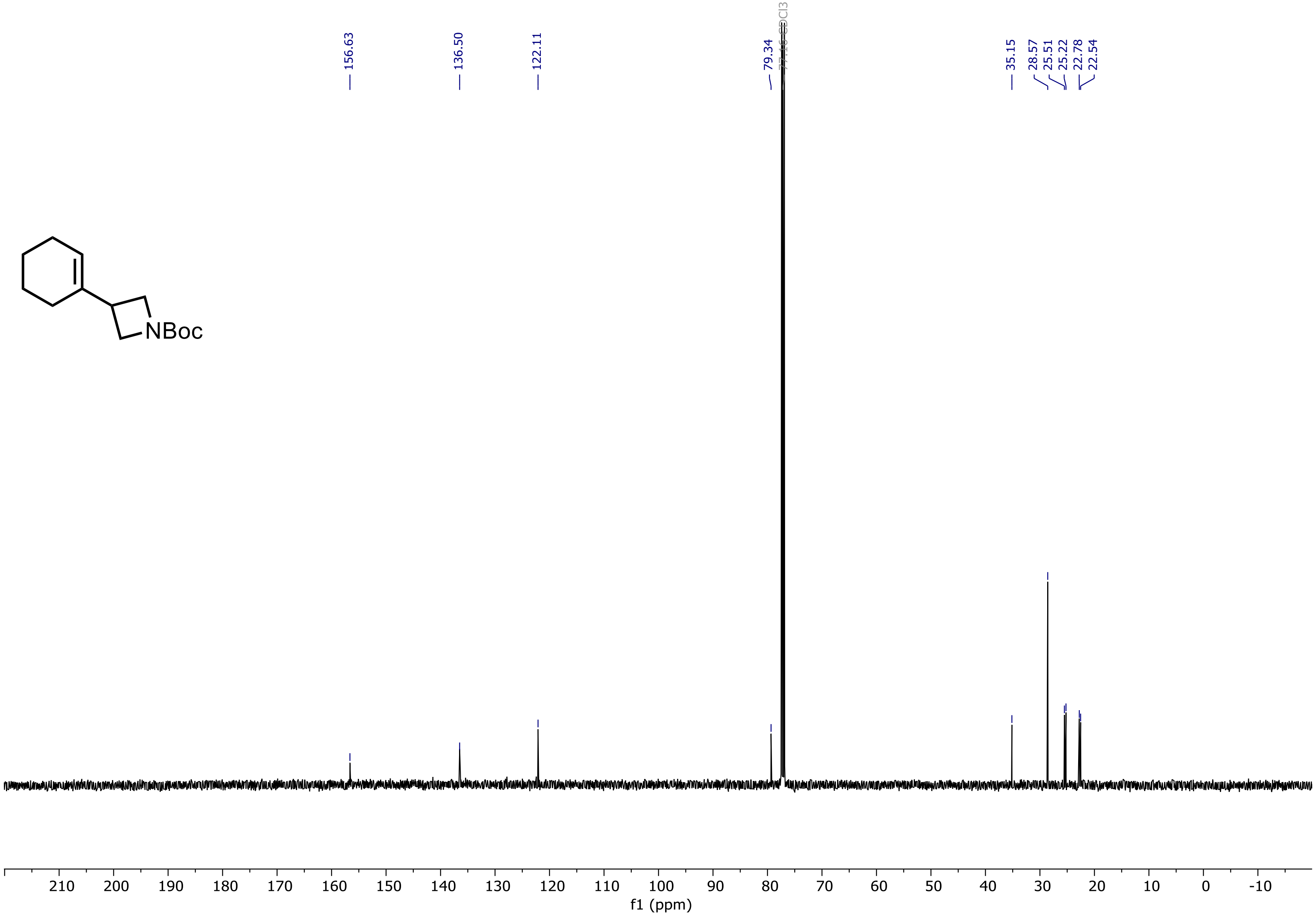

^{1}H NMR of side product 28

$CDCl_3$, 23°C

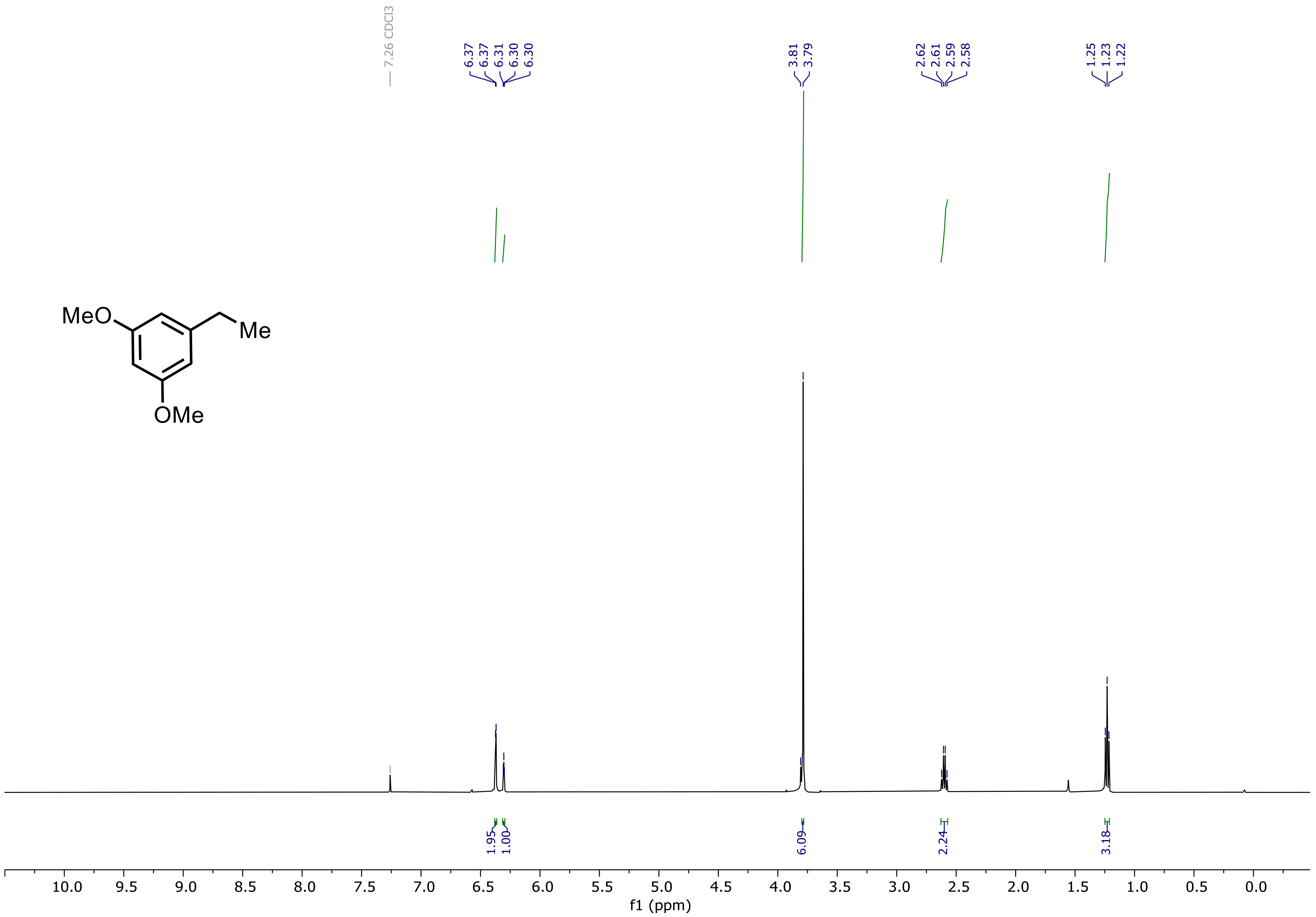

^{13}C NMR of side product 28

$CDCl_3$, 23°C

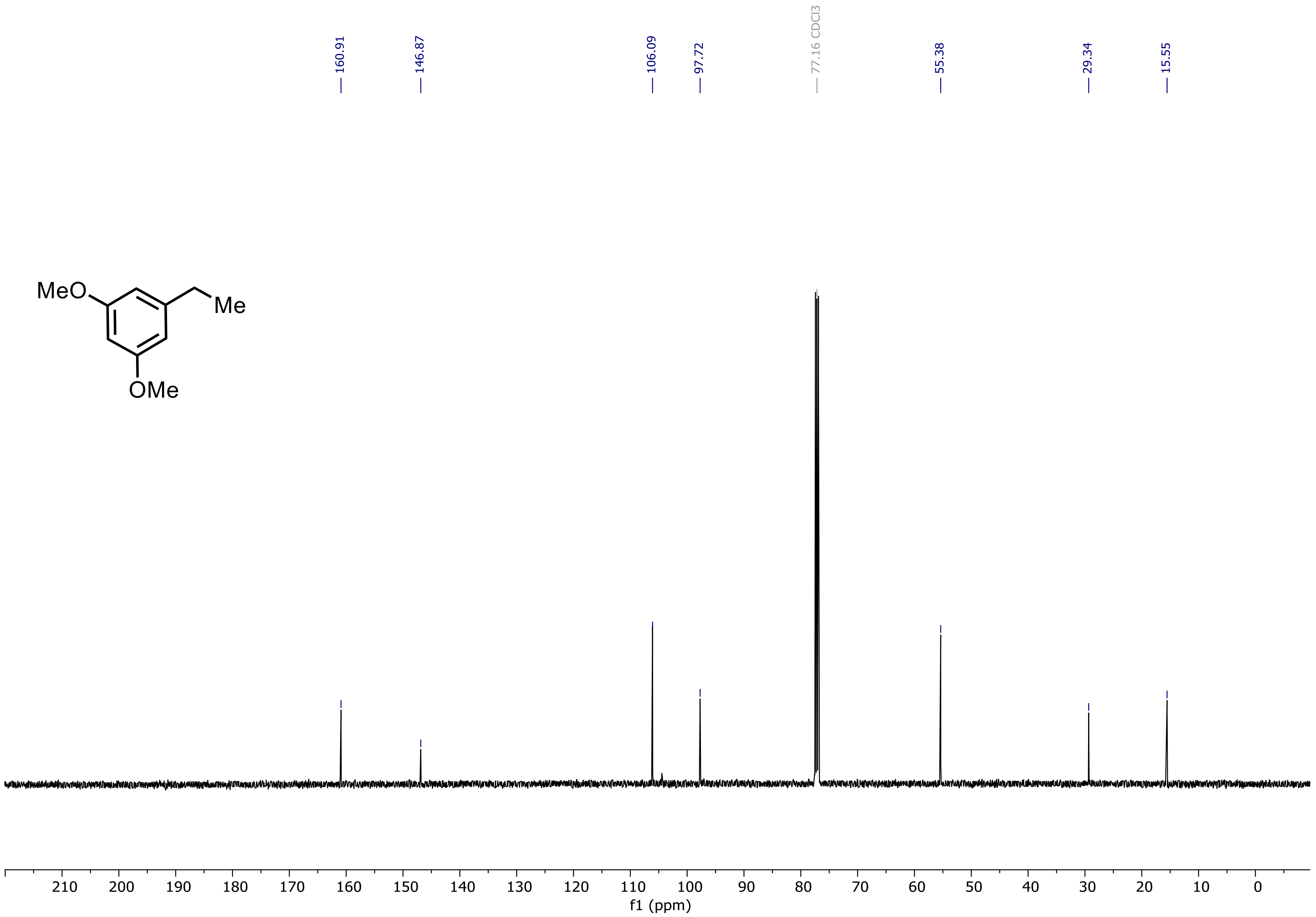

¹H NMR of side product 29

CDCl₃, 23°C

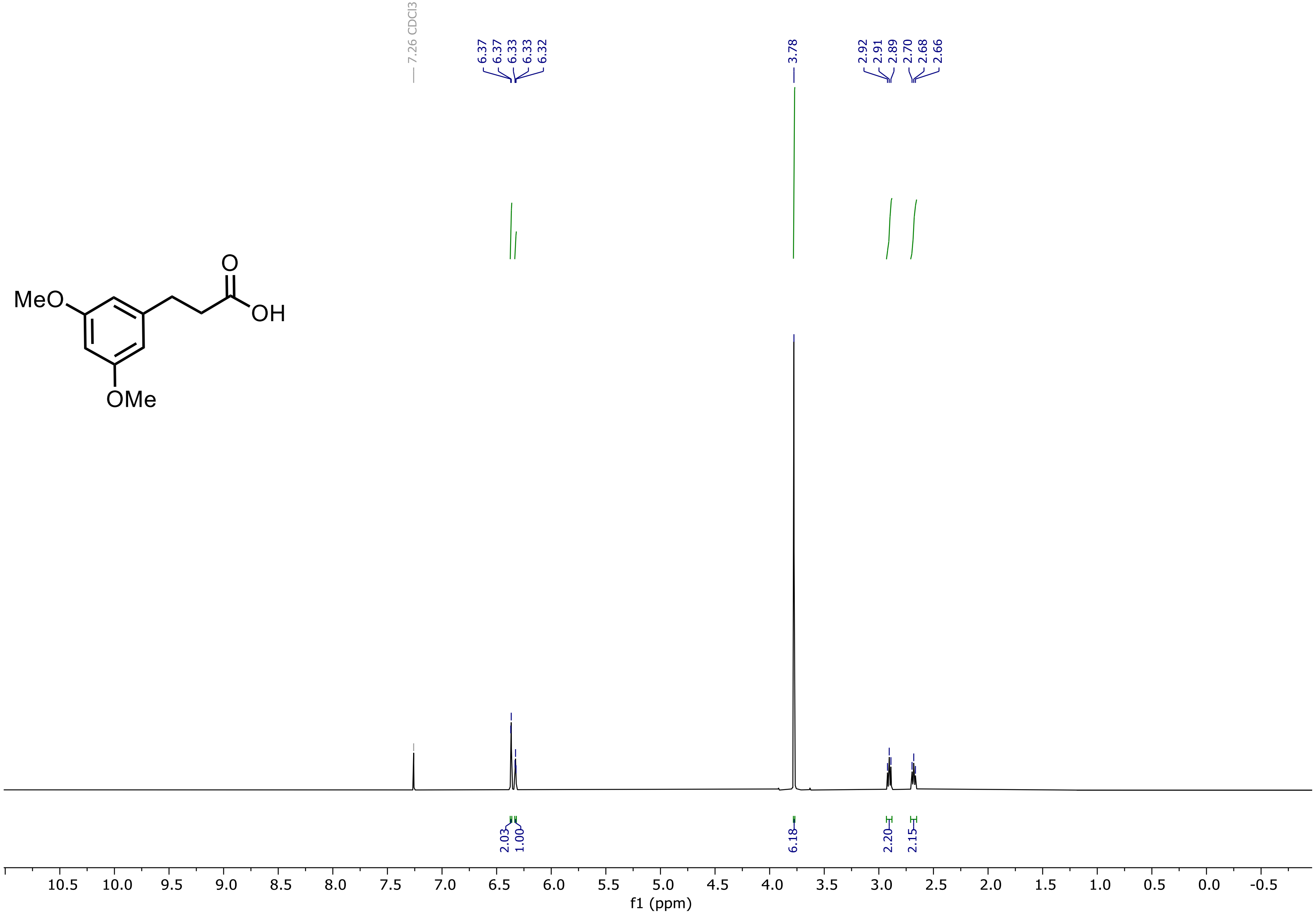

^{13}C NMR of side product 29

$CDCl_3$, 23°C

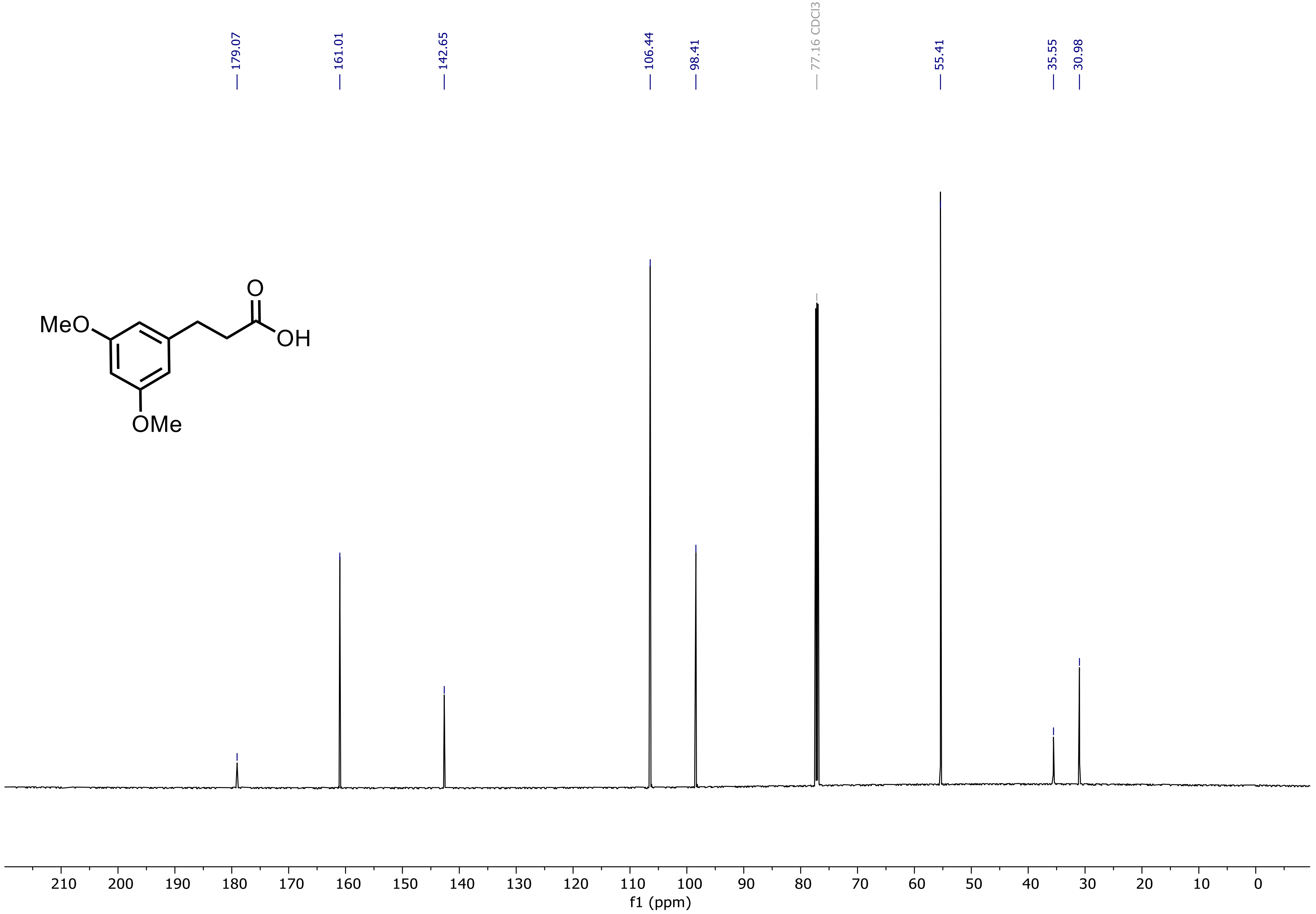

^{1}H NMR of side product 30

CDCl₃, 23°C

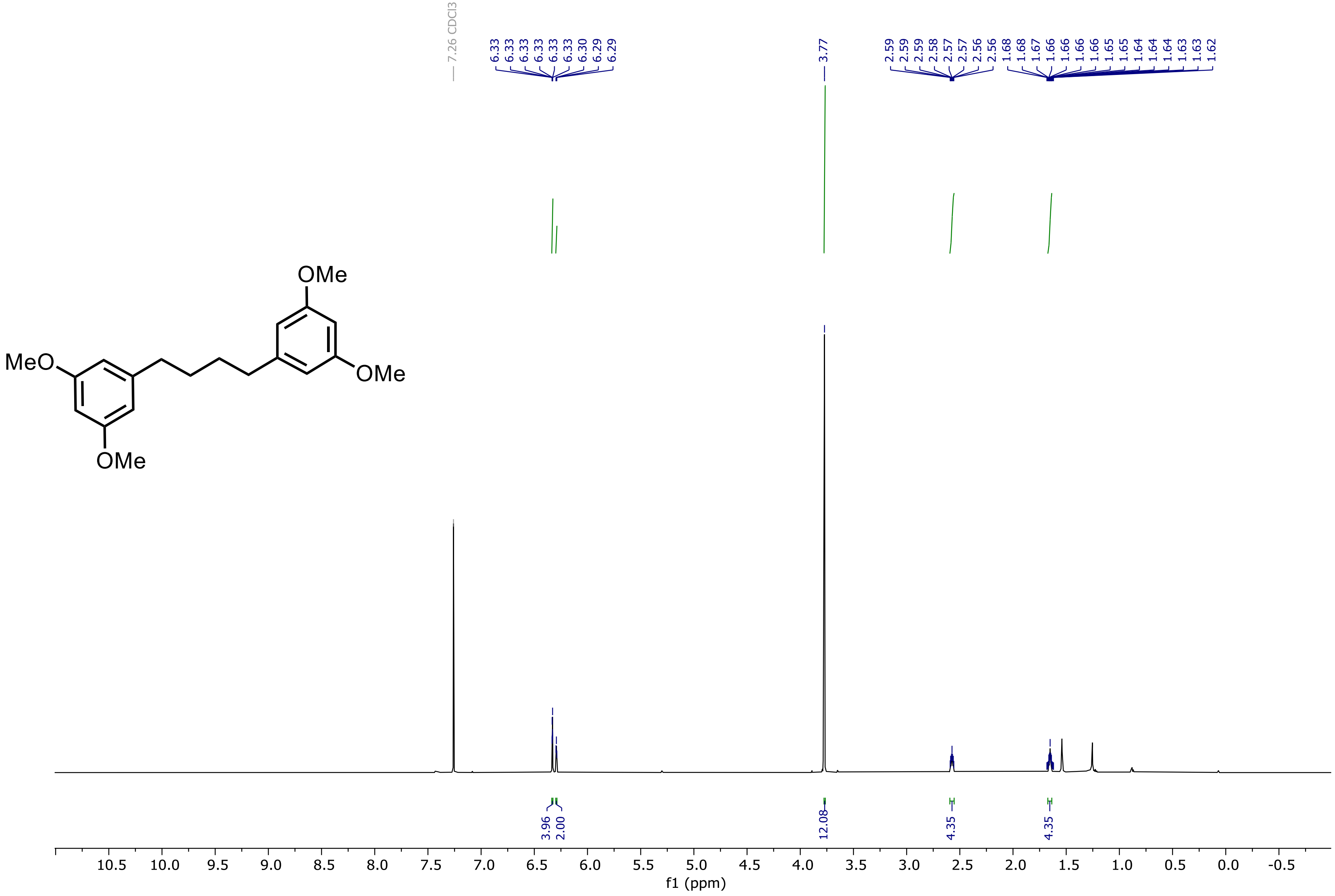

^{13}C NMR of side product 30

CDCl$_3$, 23°C

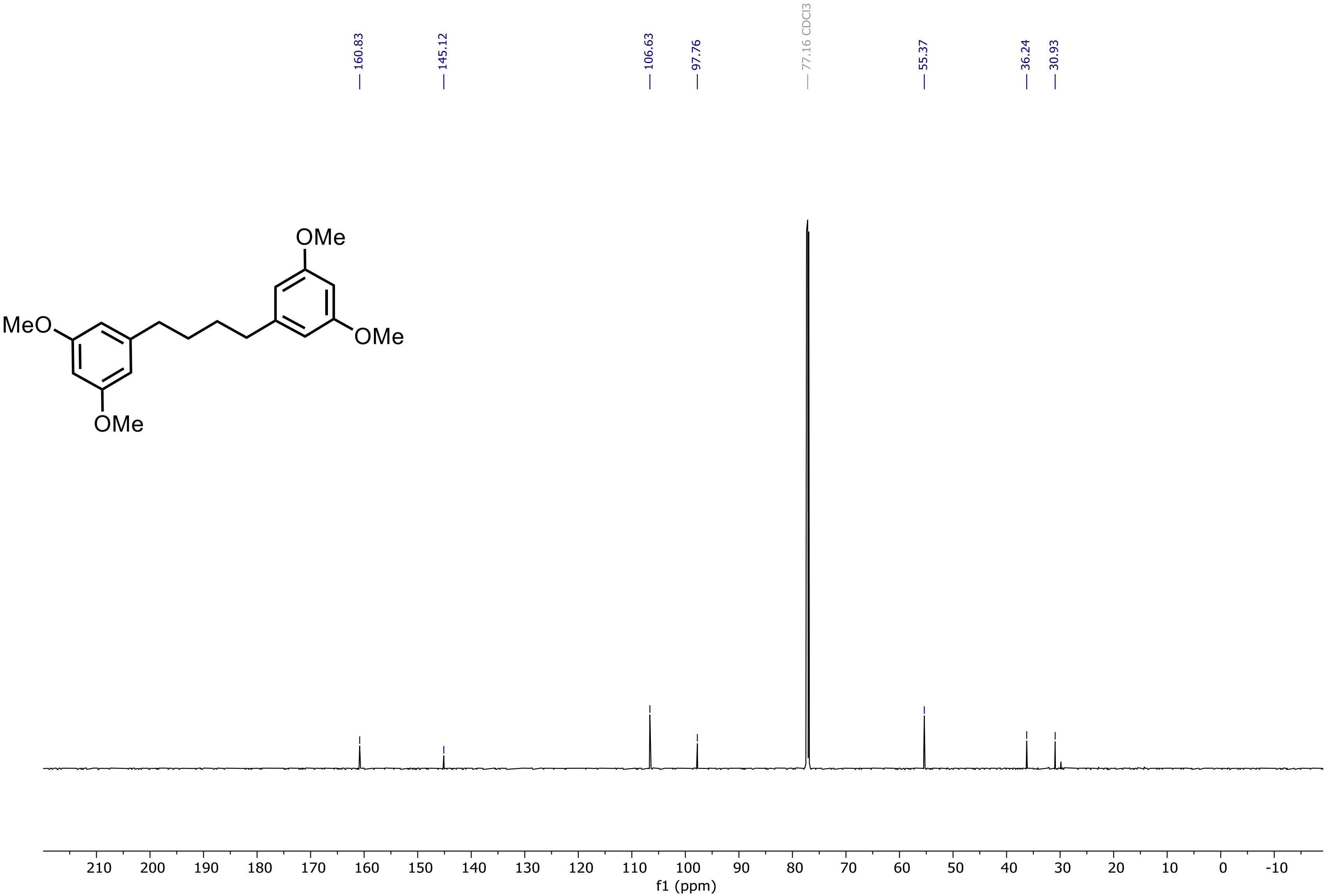

REFERENCES

1. Fulmer, G. R.; Miller, A. J. M.; Sherden, N. H.; Gottlieb, H. E.; Nudelman, A.; Stoltz, B. M.; Bercaw, J. E.; Goldberg, K. I.; NMR Chemical Shifts of Trace Impurities: Common Laboratory Solvents, Organics, and Gases in Deuterated Solvents Relevant to the Organometallic Chemist. *Organometallics* **2010,** *29* (9), 2176-2179.

2. Guerrero, A.; Hughes, D. L.; Bochmann, M.; Synthesis and crystal structure of ethyl zinc chloride. *Organometallics* **2006**, *25* (6), 1525–1527.

3. Posa, L.; Tomek, P.; Lamba, S.; Sarojini, V; Barker D.; Development of Truncated Battacin Antimicrobials Featuring Novel N-terminal Fatty Acids with an Excellent Safety Profile. *Bioorg. Med. Chem. Lett.* **2023**, *96*, 129535.

4. Chen, J.; Li, J.; Plutschack, M. B.; Berger, F.; Ritter, T.; Regio- and Stereoselective Thianthrenation of Olefins to Access Versatile Alkenyl Electrophiles. *Angew. Chem. Int. Ed.* **2020**, *59* (14), 5616 – 5620.

5. Williams, D. R.; Okha, F. M.; Ward, S. A., Studies Toward the Synthesis of Leiodolide A. *Org. Lett.* **2025**, *27* (10), 2279 – 2283.

6. Okanishi, Y.; Takemoto, O.; Kawahara, S.; Hayashi, S.; Takanami, T.; Yoshimitsu, T.; Red-Light-Promoted Radical Cascade Reaction to Access Tetralins and Dialins Enabled by Zinc(II)porphyrin, A Light-Flexible Catalyst. *Org. Lett.* **2024**, *26* (18), 3929 – 3934.

7. Ling, B.; Yao, S.; Ouyang, S.; Bai, H.; Zhai, X.; Zhu, C.; Li, W.; Xie, J.; Nickel-Catalyzed Highly Selective Radical C−C Coupling from Carboxylic Acids with Photoredox Catalysis. *Angew. Chem. Int. Ed.* **2024**, *63* (32), e202405866.

8. Yang, T.; Jiang, Y.; Luo, Y.; Lim, J. J. H.; Lan, Y.; Koh, M. J.; Chemoselective Union of Olefins, Organohalides and Redox-Active Esters Enables Regioselective Alkene Dialkylation. *J. Am. Chem. Soc.* **2020**, *142* (51), 21410 – 21419.

9. Lu, X.; Xiao, B.; Liu, L.; Fu Y.; Formation of C(sp^3)–C(sp^3) Bonds through Nickel-Catalyzed Decarboxylative Olefin Hydroalkylation Reactions. *Chem. Eur. J.* **2016**, *22* (32), 11161 – 11164.

10. Yu, L.; Tang, M.-L.; Si, C.-M.; Meng, Z.; Liang, Y.; Han, J.; Sun, X., Zinc-Mediated Decarboxylative Alkylation of *Gem*-difluoroalkenes. *Org. Lett.* **2018**, *20* (15), 4579 – 4583.

11. Brauer, J.; Quraishi, E.; Kammer, L. M.; Opatz T., Nickel-Mediated Photoreductive Cross Coupling of Carboxylic Acid Derivatives for Ketone Synthesis. *Chem. Eur. J.* **2021**, *27* (72), 18168 – 18174.

12. Nagy, B.; Gonda, Z.; Földesi, T.; Fehér, P. P.; Stirling, A.; Tolnai, G. L.; Novák, Z., Photoinduced Decarboxylative Borylation of *N*-Hydroxyphthalimide Esters with Hypoboric Acid. *Org. Lett.* **2024**, *26* (11), 2292 – 2296.

13. Le, L.; Zeng, H.; Zhou, W.; Tang, N.; Yin, S.-F.; Kambe, N; Qiu, R., Catalyst-Free, Zn-Mediated Decarboxylative Coupling of Chlorostibines to Access Alkylstibines with Stable C(sp^3)–Sb Bonds. *Org. Lett.* **2024**, *26* (28), 6018 – 6023.

14. Serafino, A.; Pierre, H.; Le Vaillant, F.; Boutet, J.; Guillamot, G.; Neuville, L.; Masson, G., Visible-Light-Driven Decarboxylative Borylation: Rapid Access to α- and β-Amino-boronamides. *Org. Lett.* **2023**, *25* (51), 9249 – 9254.

15. Brunetti, A.; Garbini, M.; Gino Kub, N.; Monari, M.; Pedrazzani, R.; Zanardi, C.; Bertuzzi, G.; Bandini, M., Electrochemical Site-Selective Alkylation of Tropones via Formal $C(sp^3)-C(sp^2)$ Coupling Reaction. *Adv. Synth. Catal.* **2024**, *366* (9), 1965 – 1971.

16. Chandrachud, P. P.; Wojtas, L.; Lopchuk, J. M., Decarboxylative Amination: Diazirines as Single and Double Electrophilic Nitrogen Transfer Reagents. *J. Am. Chem. Soc.* **2020**, *142* (52), 21743 – 21750.

17. Ma, J.; Lin, J.; Zhao, L.; Harms, K.; Marsch, M.; Xie, X.; Meggers, E., Synthesis of β-Substituted γ-Aminobutyric Acid Derivatives through Enantioselective Photoredox Catalysis. *Chem. Int. Ed.* **2018**, *57* (35), 11193 – 11197.

18. Pagire, S. K.; Shu, C.; Reich, D.; Noble, A.; Aggarwal, V. K., Convergent Deboronative and Decarboxylative Phosphonylation Enabled by the Phosphite Radical Trap "BecaP". *J. Am. Chem. Soc.* **2023**, *145* (33), 18649 – 18657.
